# Supplementary material for: Robust and efficient hydrogenation of carbonyl compounds catalysed by mixed donor Mn(I) pincer complexes
Source: Nat Commun. 2021 Jan 4;12:12. doi: 10.1038/s41467-020-20168-2 (PMC7782525; doi:10.1038/s41467-020-20168-2)
Supplement: Supplementary file 1 — Supplementary Information [file 41467_2020_20168_MOESM1_ESM.pdf]

Supplementary Information

Robust and Efficient Hydrogenation of Carbonyl Compounds Catalysed by Mixed Donor Mn(I) Pincer Complexes

Wenjun Yang,[1] Ivan Yu. Chernyshov,[2] Robin K. A. van Schendel,[1] Manuela Weber,[3] Christian Müller,[3] Georgy A. Filonenko, *[1] and Evgeny A. Pidko*[1]

[1] Inorganic Systems Engineering group, Department of Chemical Engineering, Faculty of Applied Sciences, Delft University of Technology, Van der Maasweg 9, 2629 HZ, Delft, The Netherlands

[2] TheoMAT Group, ChemBio cluster, ITMO University, Lomonosova 9, St. Petersburg, 191002, Russia

[3] Institute of Chemistry and Biochemistry, Freie Universität Berlin, Fabeckstraße 34/36, Berlin, D-14195, Germany

Corresponding authors: Georgy A. Filonenko (G.A.Filonenko@tudelft.nl)

Evgeny A. Pidko (E.A.Pidko@tudelft.nl)

Supplementary Methods

All manipulations were, unless stated otherwise, performed under inert atmosphere in an argon filled glovebox (INERT) or using standard Schlenk techniques. Anhydrous solvents were either dispensed from an Inert PureSolv solvent purification system or dried using 3/4 Å molecular sieves and were degassed before use. Chemicals were purchased from Sigma-Aldrich, Strem, abcr, or TCI. Liquid hydrogenation substrates were degassed and dried by molecular sieves before use. Air and/or moisture sensitive materials were stored in the glovebox. Deuterated solvents were purchased from Eurisotop, dried using molecular sieves, degassed and stored in the glovebox.

NMR spectra were recorded on an Agilent 400-MR DD2 400 MHz spectrometer equipped with a 5 mm ONE NMR probe. All ^{13}C and ^{31}P NMR spectra were recorded with ^{1}H decoupling. All chemical shifts were referenced to residual solvent peaks [$CDCl_3$: 7.26 ppm (^{1}H), 77.2 ppm (^{13}C); CD_2Cl_2: 5.32 ppm (^{1}H), 53.5 ppm (^{13}C); THF-*d8*: 1.72/3.58 ppm (^{1}H), 67.6/25.4 ppm) (^{13}C)]. Proton and carbon assignments were made on basis of combined gCOSY and gHSQC spectra. FTIR (ATR and transmittance modes) was measured on a Bruker Alpha II spectrometer. The solutions of the complexes in THF (0.05 M) were filled in an IR-cell with CaF_2 windows and an optical path length of 0.1 mm which was purged with nitrogen prior to use. The cell was stabilized at 25 °C. The spectra were taken with a resolution of 2 cm^{-1}. Elemental analyses were performed by Mikroanalytisches Laboratorium Kolbe, Oberhausen, Germany.

Synthesis of 1:

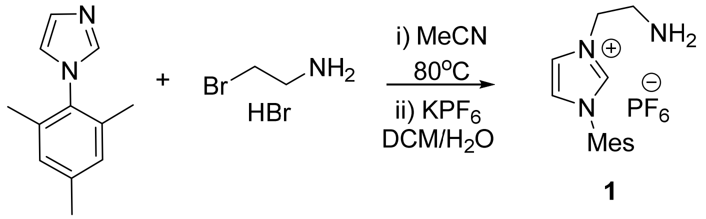

1

A suspension of 2-bromoethylamine hydrobromide (2.05 g, 10 mmol) was prepared in a Schlenk tube in dry MeCN (15 mL). The mixture was heated to 80°C, and 4.65 gram of mesityl imidazole (25 mmol, prepared according to literature procedure[1] and sublimed under vacuum before use) was added in three portions in three hours. After heating overnight, solution was cooled down the solvent was removed on rotary evaporator. The oily residue was dissolved in water, neutralized with Na_2CO_3 until pH 9 (white solid precipitated out) and extracted with diethyl ether for three times to recover unreacted imidazole. The aqueous phase was stirred vigorously with KPF_6 (20 mmol, 2 eq.) for 1 h to form a turbid emulsion followed by extraction with dichloromethane. Combined organic phases were dried over anhydrous Na_2SO_4 and evaporated to dryness to produce viscous oil. Removal of residual water from the product was done by co-evaporating water with acetonitrile three times on the rotary evaporation. Resulting oil was used without further purification.

^{1}H NMR (400 MHz, $CDCl_3$, 297 K) δ 8.65 (s, 1H), 7.66 (s, 1H), 7.15 (t, J = 1.8 Hz, 1H), 7.02 (s, 2H), 4.44 – 4.34 (m, 2H), 3.23 – 3.13 (m, 2H), 2.35 (s, 3H), 2.04 (s, 6H).

Synthesis of 2:

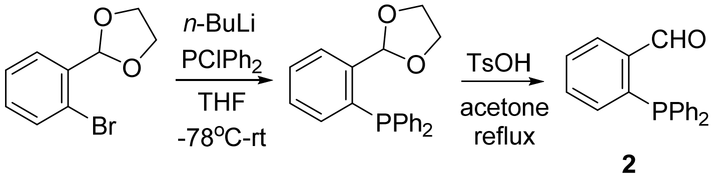

2

Under an inert atmosphere, a solution of 2-(o-bromophenyl)-1,3-dioxolane (0.57 g, 2.5 mmol) in 10 mL THF was cooled down to -78 °C. *n*-BuLi in hexane (1.3 mL, 3.25 mmol, 2.5 M) was slowly added via syringe. After stirring at -78 °C for 2 h, diphenylphosphine chloride (0.55 g, 2.5 mmol) was added slowly via syringe. The solution was warmed up to rt and stirred overnight before quench with H_2O (20 mL). The organic phase was extracted with Et_2O (20 mL*3), dried over anhydrous Na_2SO_4 and concentrated. The resulting oily liquid was purified by recrystallization from hot EtOH and cooled to -25 °C, to afford the known title compound as a waxy white solid to provide the 2-(o-Diphenylphosphinophenyl)-1,3-dioxolane as a white solid in 73 % yield (0.61 g).

To the solution of obtained compound above (0.61g, 1.8 mmol) in acetone (15 mL) was added *para*-toluenesulfonic acid (15.48 mg, 0.09 mmol) and refluxed for 8 h. The resulting solution was

concentrated and purified by column chromatography to afford compound **2** as yellow solid. The spectroscopic data for this product match the literature data.[2]

Synthesis of ligand L1:

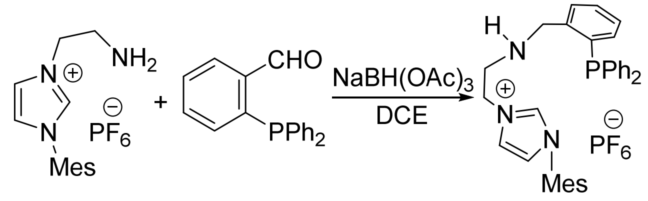

1 **2** **L1**

Under an inert atmosphere, compound **1** (3 g, 8 mmol) and **2** (2.32 g, 8 mmol) were dissolved in DCE (20 mL) and stirred overnight. Sodium triacetoxyborohydride (4.24 g, 20 mmol) was then slowly added as solid to the resulting solution. After stirring for another night, the solution was quenched by water and extracted with DCM, The combined organic phases were dried over anhydrous Na_2SO_4, concentrated and purified by column chromatography to provide the compound **L1** as white foam solid in 81% yield (4.2 g). The compound was used for pre-catalyst **3** preparation without further purification.

^{1}H NMR (400 MHz, $CDCl_3$, 297 K) δ 8.51 (s, 1H), 7.60 (s, 1H), 7.37 – 7.27 (m, 6H), 7.20 – 7.18 (m, 7H), 7.05 (s, 1H), 6.98 (s, 2H), 6.90 – 6.87 (m, 1H), 4.29 – 4.22 (m, 2H), 3.94 (s, 2H), 3.02 – 2.90 (m, 2H), 2.34 (s, 3H), 1.97 (s, 5H); ^{31}P{^{1}H}NMR (162 MHz, $CDCl_3$, 297 K) δ -16.1 (s); ^{13}C {^{1}H}NMR (101 MHz, $CDCl_3$, 297 K) δ 143.7, 143.4, 141.3, 136.6, 136.4, 136.3, 135.7, 135.6, 134.5, 133.9, 133.8, 133.6, 130.6, 129.8, 129.4, 129.3, 129.0, 128.9, 128.7, 128.6, 128.2, 127.8, 125.3, 123.8, 122.8, 52.0, 50.0, 47.8, 21.5, 21.1, 17.1; HRMS (m/z): $[M-PF_6]^+$ Calcd. for $C_{33}H_{35}N_3P_1^+$, 504.2563; found 504.2544.

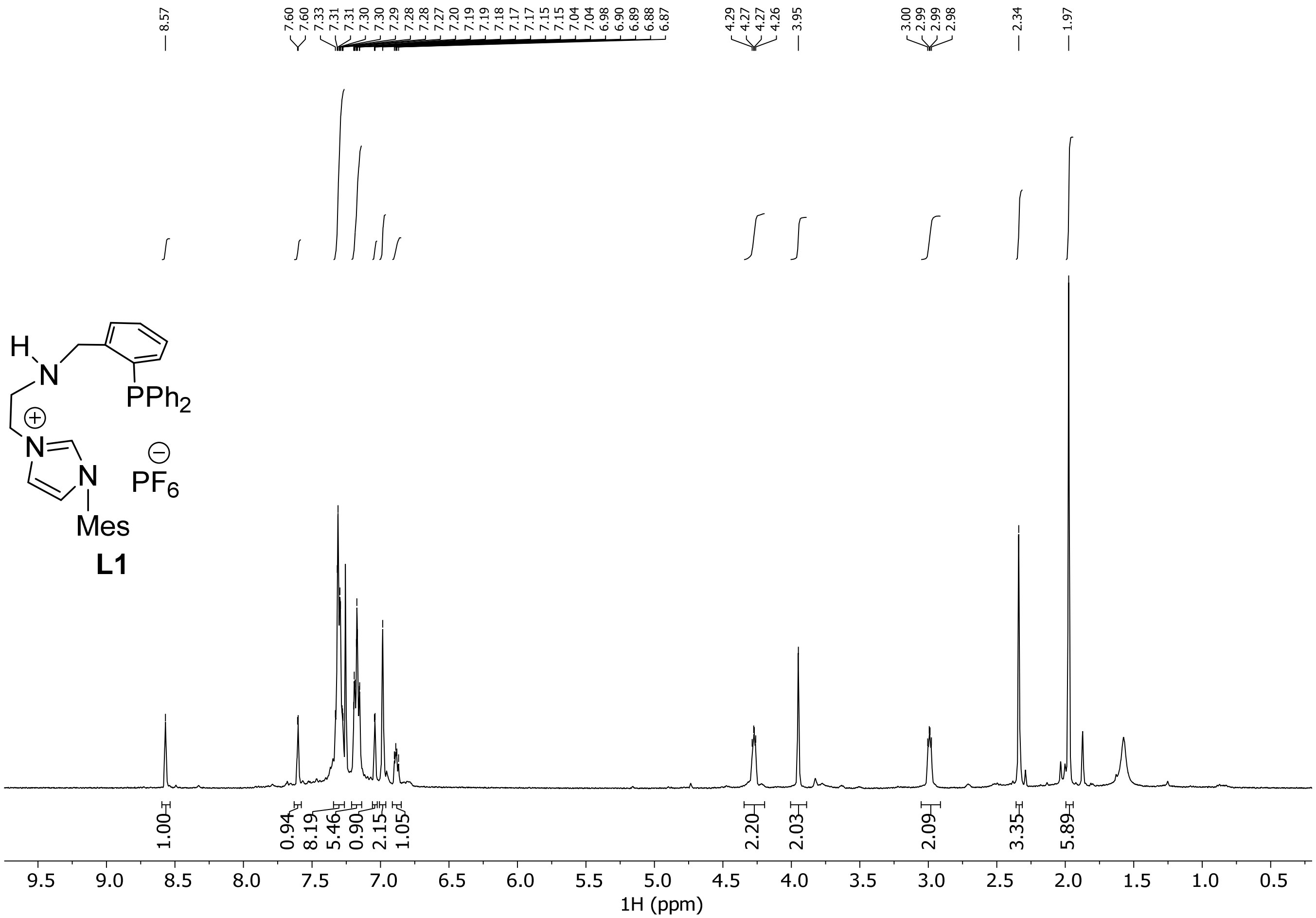

Supplementary Figure 1. ^{1}H-NMR spectrum of ligand **L1** in CD_3Cl (400 MHz).

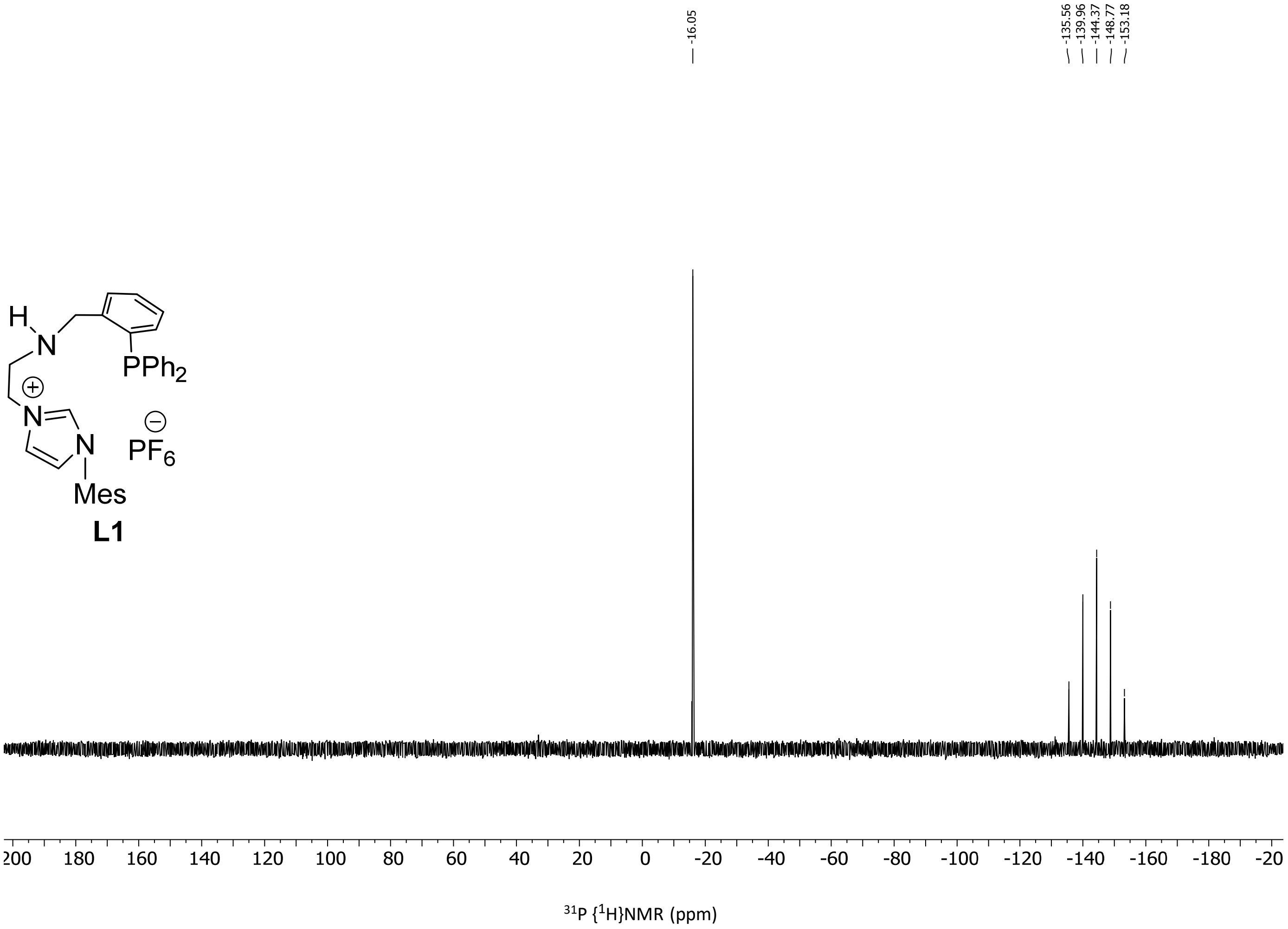

Supplementary Figure 2. ^{31}P {^{1}H}NMR spectrum of ligand **L1** in CD_3Cl (400 MHz).

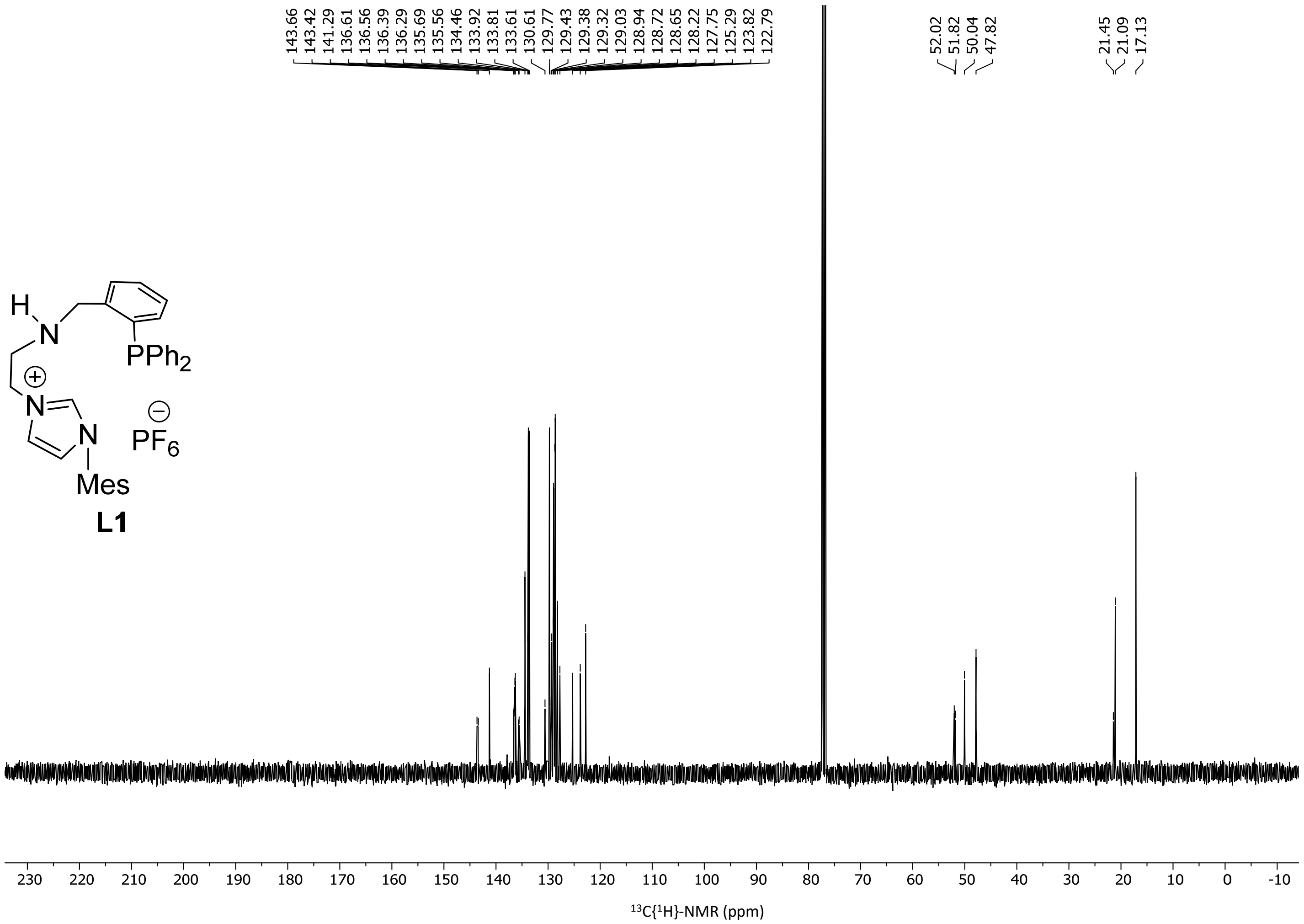

Supplementary Figure 3. ^{13}C {^{1}H}NMR spectrum of ligand **L1** in CD_3Cl (400 MHz)

Synthesis of complex 3:

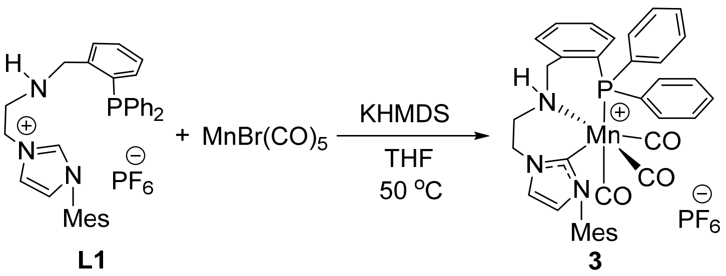

To the orange suspension of [MnBr(CO)$_5$] (137.5 mg, 0.5 mmol) in THF (5 mL) was added ligand **L1** (325 mg, 0.5 mmol) and stirred for 3 h at 50 °C. The resulting solution was then cooled down to room temperature, potassium bis(trimethylsilyl)amide (110 mg, 0.55 mmol) in THF (2 mL) was added dropwise, stirred for another 3 h and evaporated to dryness. Residual oil was washed with pentane, re-dissolved in DCM, filtered through a Celite plug to remove inorganic salts and evaporated to dryness. The crude product was further purified by crystallization (diethyl ether vapor diffusion into solution in THF) to afford **3** as yellow solid in 51% yield (200.1 mg).

^{1}H NMR (400 MHz, CD_2Cl_2, 297 K) δ 7.60 – 7.52 (m, 4H, C*H*-c,c',f,g-phenyl), 7.47 – 7.41 (m, 3H, C*H*-b',e-phenyl) 7.37 – 7.33 (m, 2H, C*H*-b-phenyl), 7.29 (s, 1H, *CH*-imidazole), 7.25 – 7.14 (m, 2H, C*H*-a'-phenyl), 7.11 (s, 1H, *CH*-imidazole), 7.00 – 6.96 (m, 2H, one from C*H*-Mes, one from C*H*-d-phenyl), 6.76 – 6.72 (m, 2H, C*H*-a-phenyl), 6.68 (s, 1H, C*H*-Mes), 5.24 (s, 1H, *NH*), 3.89 – 3.86 (m, 2H, one from CH_2-A_2, one from CH_2-B_2), 3.68 (s, 1H, CH_2-A_1), 3.34 (s, 1H, CH_2-C_2) 3.23 – 3.21 (m, 2H, one from CH_2-B_1, one from CH_2-C_1), 2.25 (s, 3H, CH_3-Mes), 1.88 (s, 3H, CH_3-Mes), 1.55 (s, 3H, CH_3-Mes); ^{31}P{^{1}H}NMR (162 MHz, CD_2Cl_2, 297 K) δ 37.6 (s), -144.4 (hep, $^1J_{FP}$ 712.8 Hz); ^{13}C {^{1}H}NMR (101 MHz, CD_2Cl_2, 297 K) δ 217.7 (Mn-*C*O), 215.5 (Mn-*C*O), 213.9 (Mn-*C*O), 187.0 (NHC Mn-*C*), 140.2 (C*C*CH-j), 139.4 (C*C*CH-i, $^1J_{PC}$ 16.2 Hz), 136.6 (CH*C*CH-h'), 135.9 (CH*C*CH-h), 133.3, 133.2 (C*C*HCH-a'), 133.1 (CH*C*HCH-c), 133.0 (CH*C*HCH-c'), 132.9, 132.8 (C*C*HCH-a), 132.1 (CH*C*HCH-e), 131.7 (C*C*HCH-g), 131.4 (C*C*HCH-d), 131.3 (CH*C*HCH-f), 130.1 (CH*C*CH-m), 130.0 (CH*C*HCH-b), 129.6 (C*C*C-l), 129.5 (C*C*HC-Mes), 129.3 (C*C*HCH-k), 129.1, 129.0 (CH*C*HCH-b'), 126.5 (*C*H-imidazole), 125.4 (*C*H-imidazole), 56.3 (*C*H_2-A), 53.1 (*C*H_2-C), 45.8 (*C*H_2-B), 20.7, 18.5 and 17.6 (*C*H_3-Mes). FTIR-ATR (solid): ῡ [cm^{-1}] 2021 (s, ῡ CO), 1943 (s, ῡ CO), 1919 (s, ῡ CO); Elemental analysis: (calcd., found for $C_{36}H_{34}F_6MnN_3O_3P_2$): C (54.90, 54.80), H (4.35, 4.41), N (5.34, 5.33).

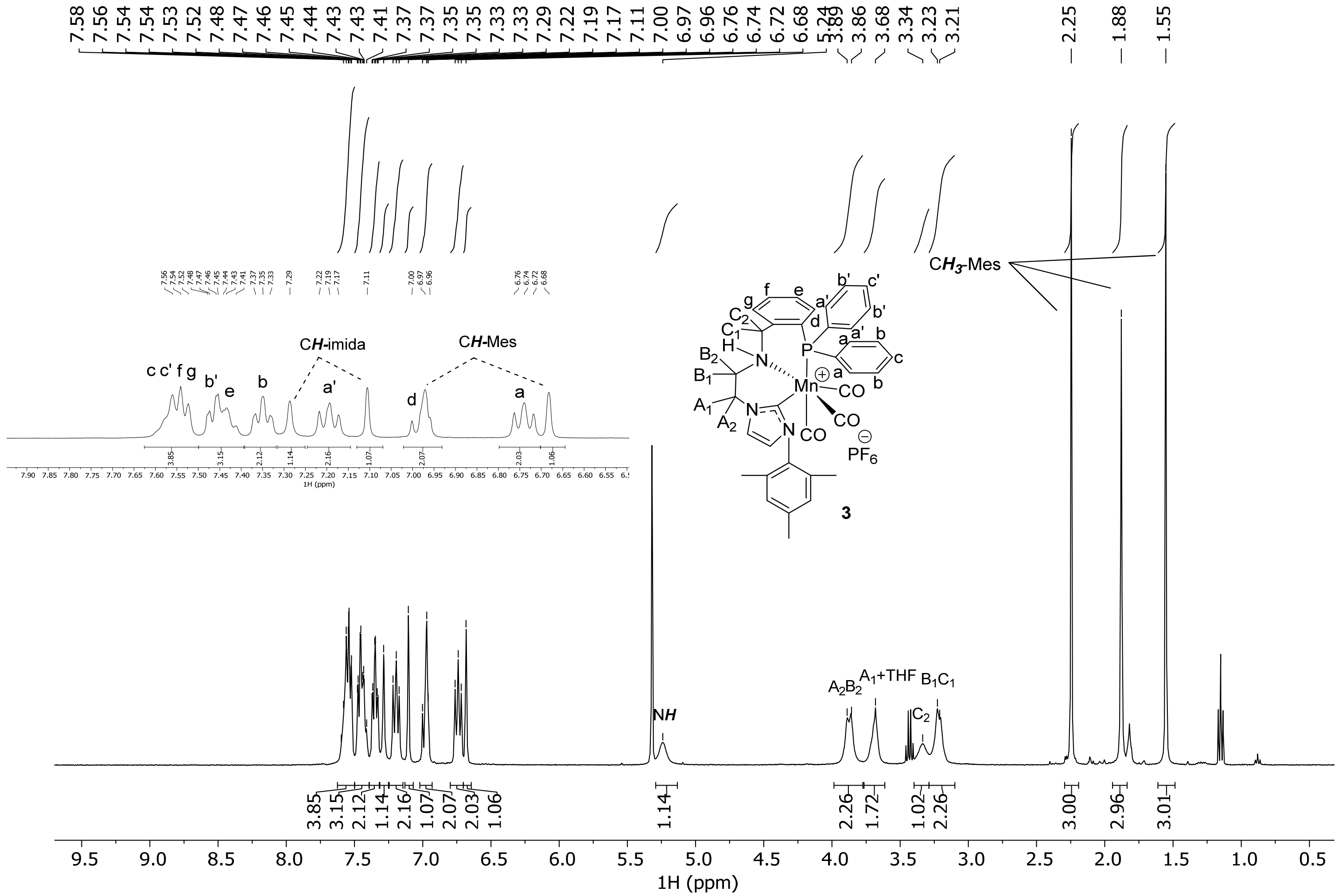

Supplementary Figure 4. ^{1}H-NMR spectrum of complex **3** in CD_2Cl_2 (400 MHz).

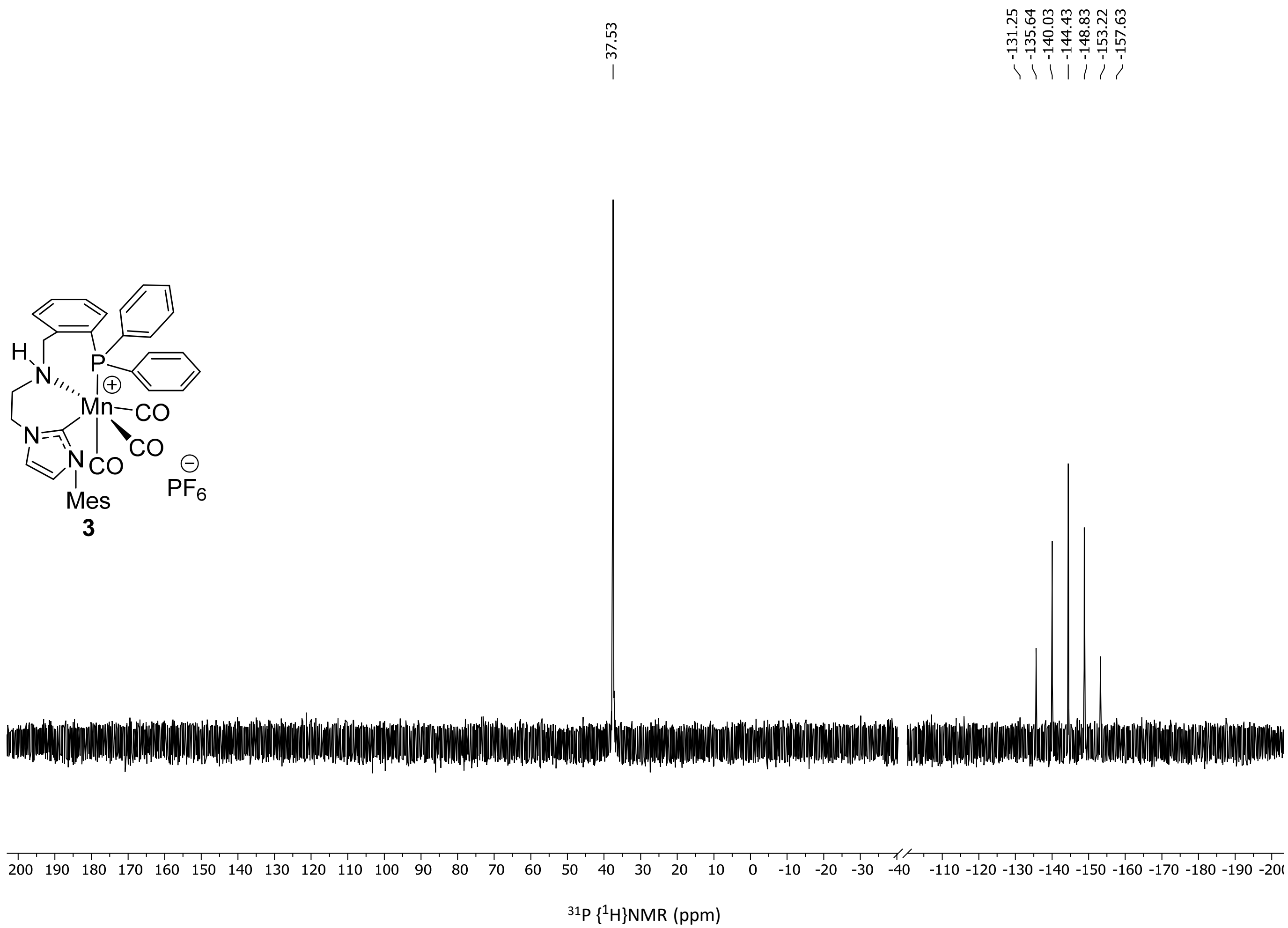

Supplementary Figure 5. ^{31}P $\{^{1}H\}$NMR spectrum of complex **3** in CD_2Cl_2 (162 MHz).

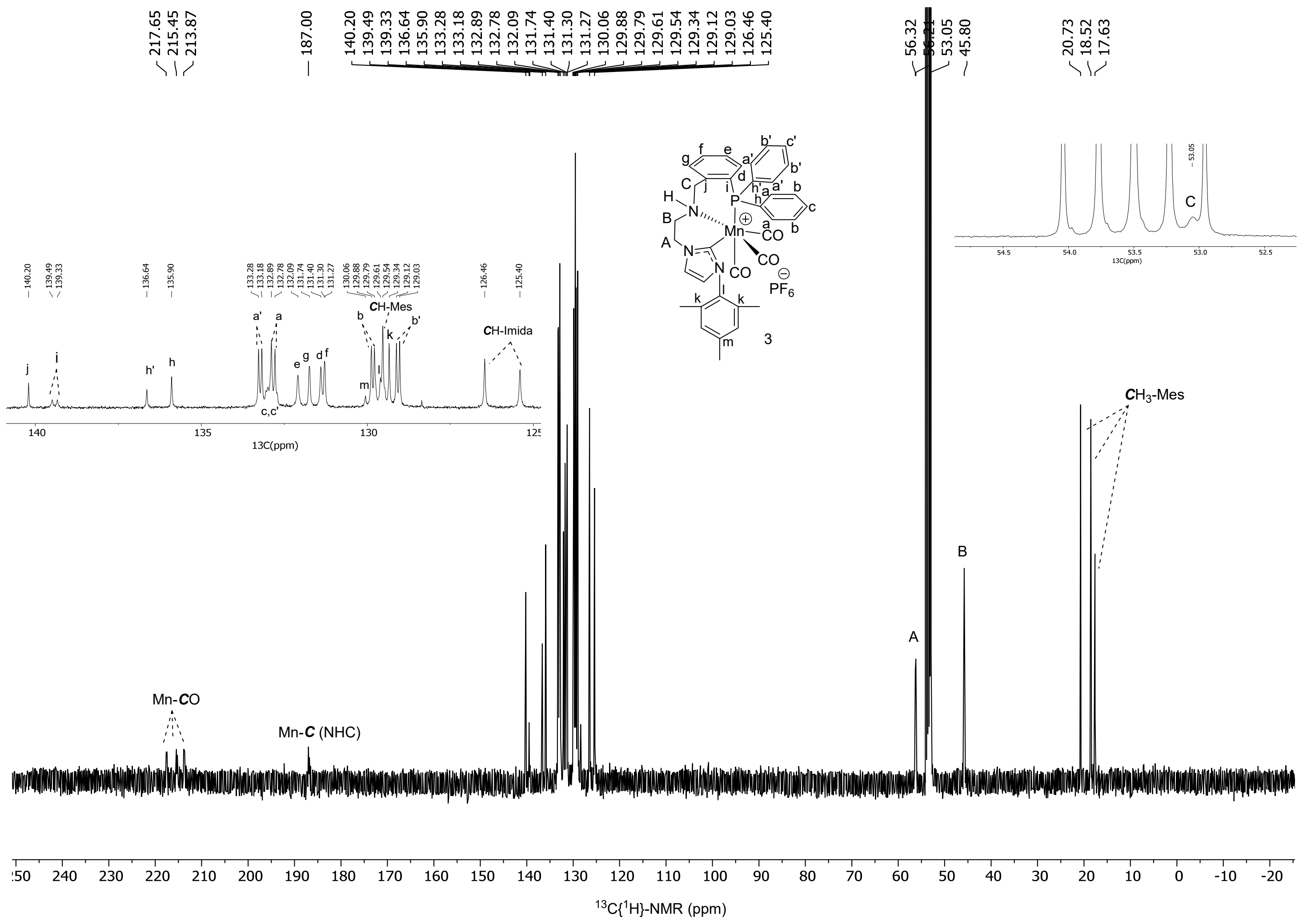

Supplementary Figure 6. $^{13}C\ \{^{1}H\}$NMR spectrum of complex **3** in CD_2Cl_2 (100 MHz).

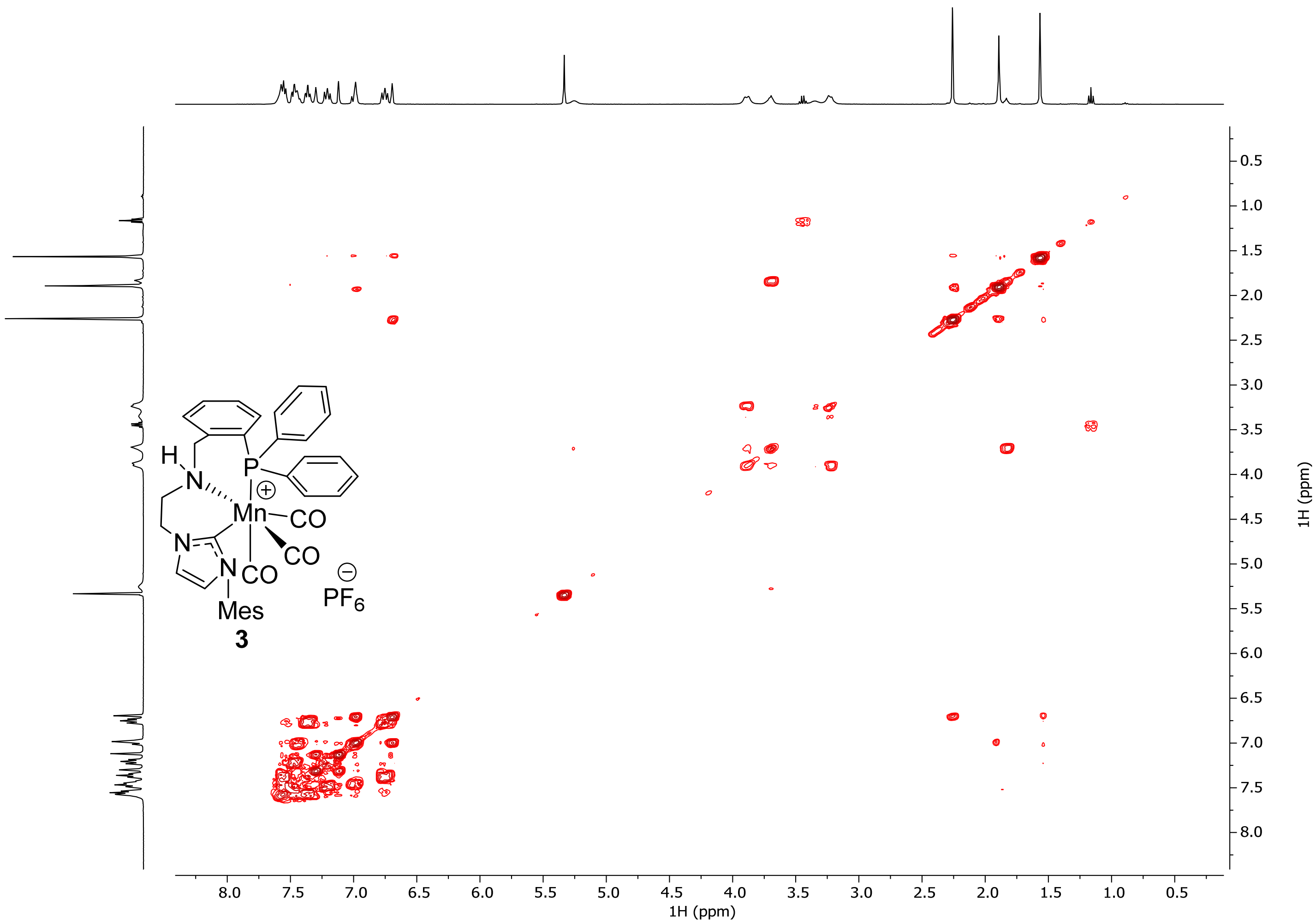

Supplementary Figure 7. gCOSY spectrum of complex **3** in CD_2Cl_2.

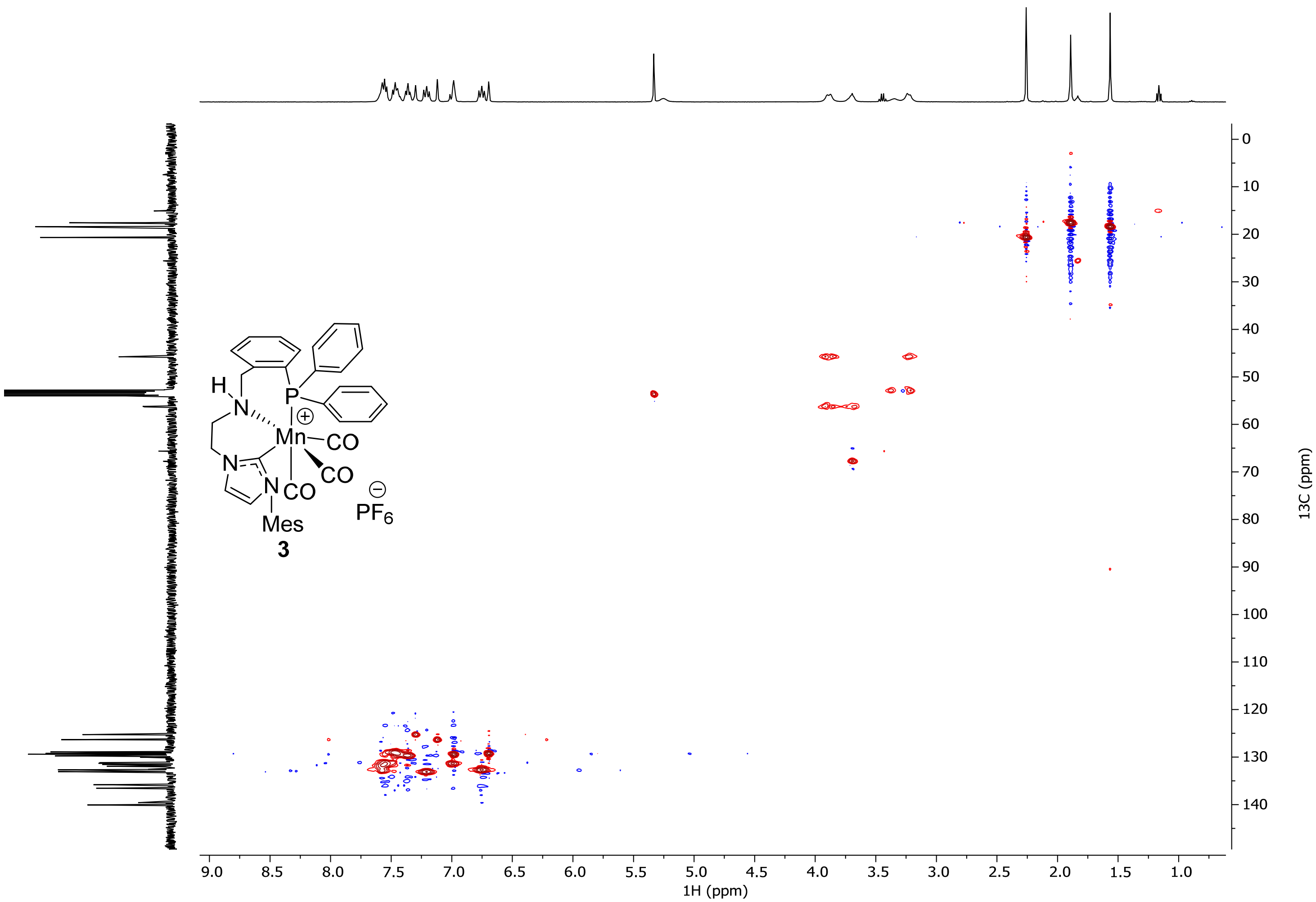

Supplementary Figure 8. gHMQC spectrum of complex **3** in CD_2Cl_2.

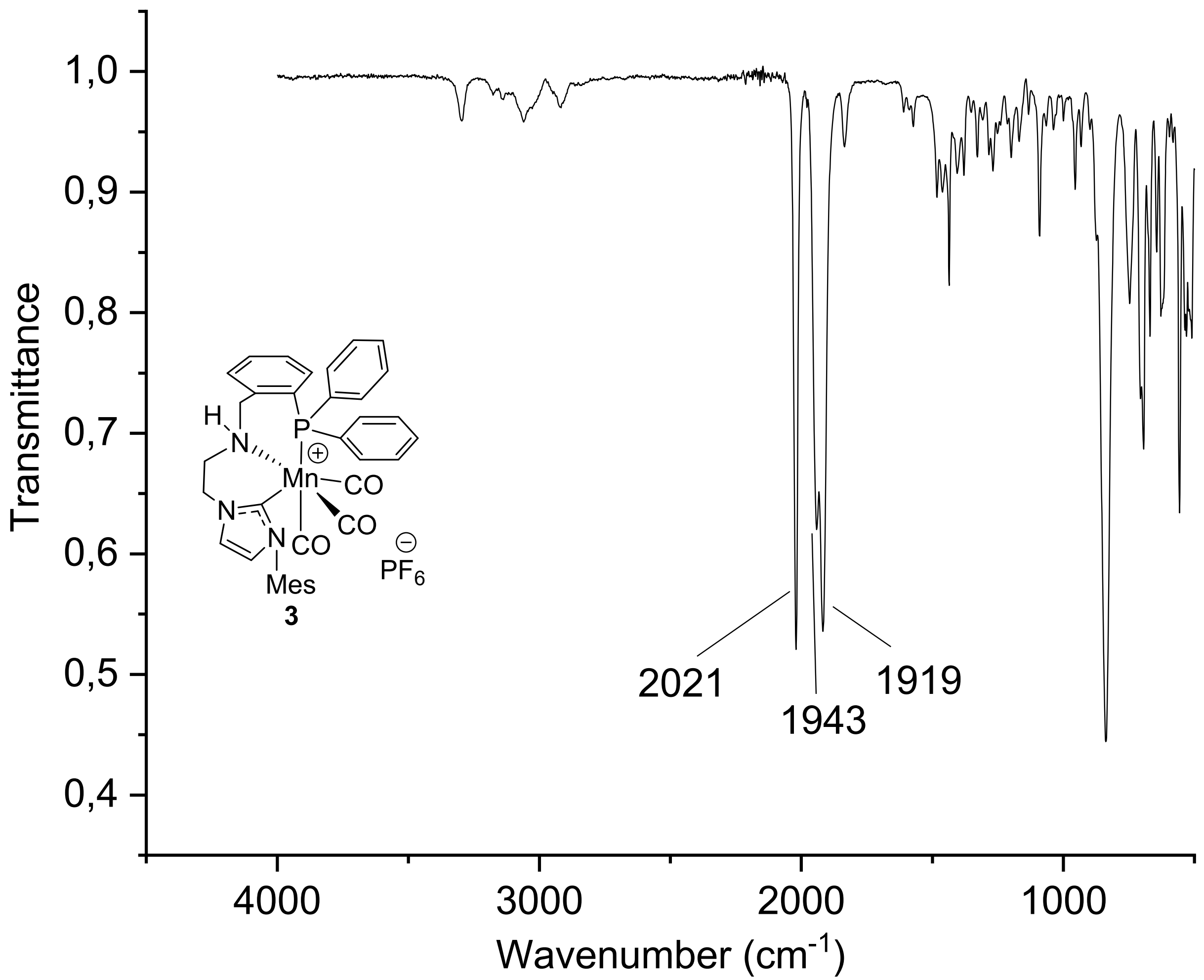

Supplementary Figure 9. IR spectrum of solid complex **3**. Solution spectrum given in Supplementary Figure 18

Synthesis of complex 4:

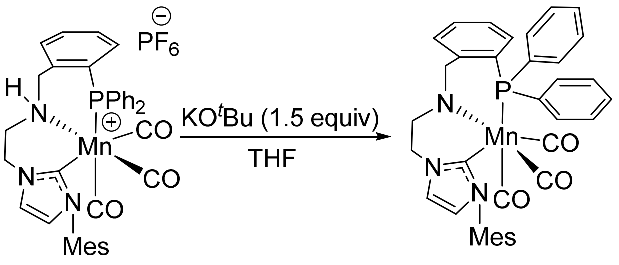

3 **4**

To the solution of complex **3** (52.5 mg, 0.067 mmol) in THF (2 mL) was dropwise added KO^{t}Bu (9.4 mg, 0.08 mmol) in 0.5 mL THF. After stirring for 1 h, the resulting mixture was evaporated to dryness. Residual oil was re-dissolved in DCM, filtered through a Celite plug to remove inorganic salt and evaporated to dryness. The crude was further purified by slow diffusion of pentane into its solution in THF to afford **6** as red crystals in 70 % yield (30.2 mg).

^{1}H NMR (400 MHz, THF-d$_8$, 297 K) δ 7.34 – 7.19 (m, 8H, C*H*-c,c',a',b',f,g-phenyl), 7.12 (t, J = 6.9 Hz, 2H, C*H*-b-phenyl), 7.06 (s, 1H, *CH*-imidazole), 6.94 – 6.82 (m, 5H, one from *CH*-imidazole, one from *CH*-Mes, three from C*H*-e,a-phenyl), 6.70 (s, 1H, *CH*-Mes), 6.54 (t, J = 8.6 Hz, 1H, C*H*-d-phenyl), 4.82 – 4.78 (m, 1H, C*H$_2$*-A$_1$), 4.10 – 4.04 (m, 1H, C*H$_2$*-A$_2$), 3.64 – 3.63 (m, 1H, C*H$_2$*-C$_1$), 3.39 – 3.35 (m, 1H, C*H$_2$*-C$_2$), 2.79 – 2.75 (m, 1H, C*H$_2$*-B$_1$), 2.23 (s, 3H, *CH$_3$*-Mes), 1.94 – 1.87 (s, 4H, three from *CH$_3$*-Mes, one from C*H$_2$*-B$_2$), 1.74 (s, 3H, *CH$_3$*-Mes); ^{31}P{^{1}H}NMR (162 MHz, THF-d$_8$, 297 K) δ 33.6 (s); ^{13}C {^{1}H}NMR (101 MHz, THF-d$_8$, 297 K) 223.0, 220.4, and 216.4 (Mn-*C*O), 192.2 (NHC Mn-*C*), 150.5 (C*C*CH-i, $^1J_{PC}$ 6.1 Hz), 142.0 (C*C*CH-j), 139.9 (CH*C*CH-m), 139.5 (C*C*HCH-k), 138.9 (C*C*HCH-k), 138.6 (C*C*C-l), 137.8 (CH*C*CH-h), 137.1(CH*C*CH-h'), 135.1 (CH*C*HCH-f), 134.1 (CH*C*HCH-e), 134.0 (C*C*HCH-d), 130.2 (C*C*HC-Mes), 130.1 (C*C*HC-Mes), 130.0 (C*C*HCH-g), 129.7 (CH*C*HCH-c), 129.2 (CH*C*HCH-c'), 128.8 (CH*C*HCH-b'), 128.2 (CH*C*HCH-b), 128.1 (CH*C*HCH-b), 127.7 (C*C*HCH-a'), 127.6 (C*C*HCH-a'), 125.5 (*C*H-imidazole), 125.4 (*C*H-imidazole), 125.3 (C*C*HCH-a), 125.2 (C*C*HCH-a), 72.3 (*C*H$_2$-A), 62.6 (*C*H$_2$-C), 51.3 (*C*H$_2$-B), 21.2, 19.5, and 18.1 (*C*H$_3$-Mes). IR (solution in THF): ῡ [cm^{-1}] 1989 (s, ῡ CO), 1901 (s, ῡ CO), 1885 (s, ῡ CO); Elemental analysis: (calcd., found for $C_{36}H_{33}MnN_3O_3P_1 \cdot H_2O$): C (65.55, 65.79), H (5.35, 5.21), N (6.37, 6.37).

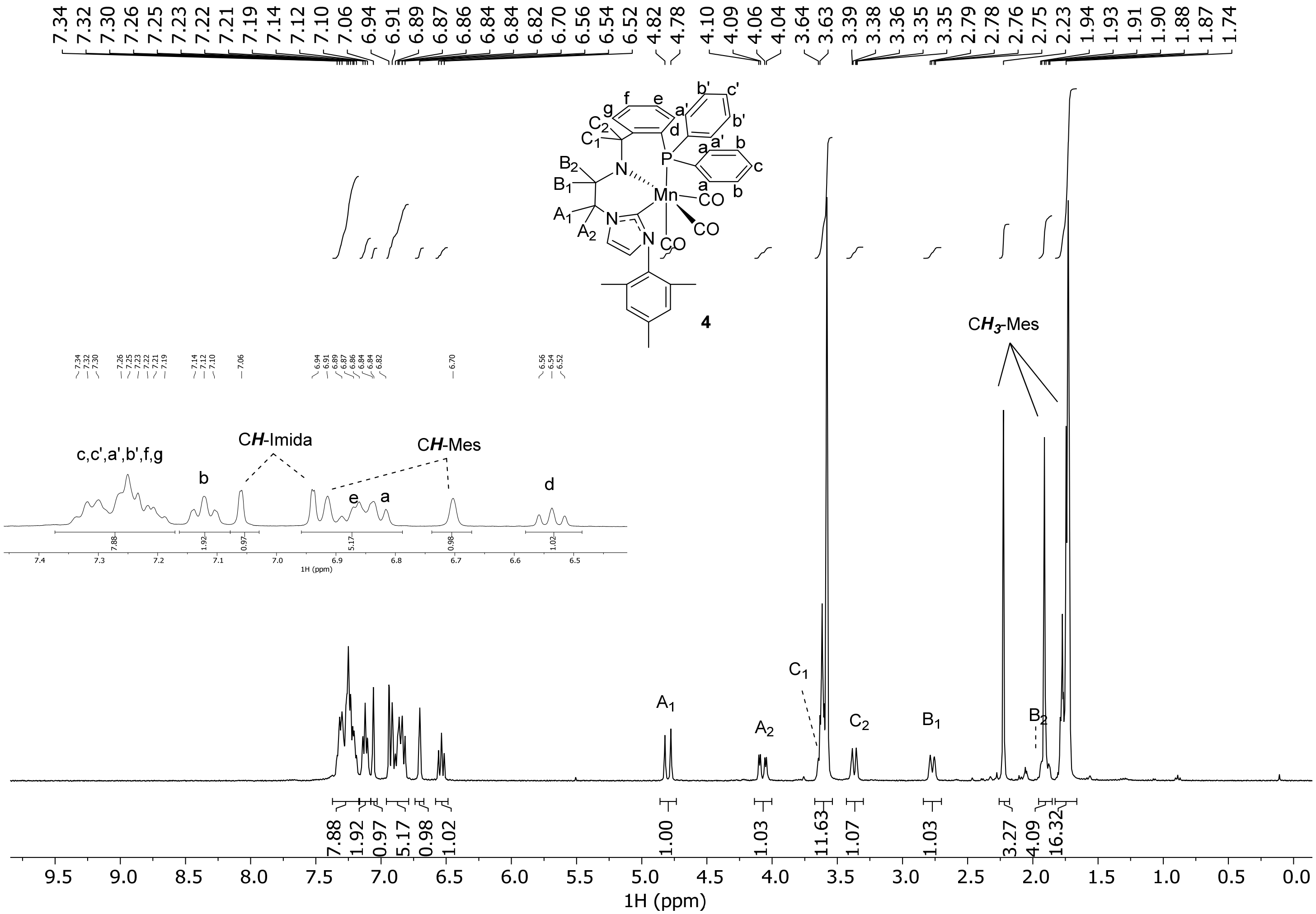

Supplementary Figure 10. ^{1}H-NMR spectrum of complex **4** in THF-d_8 (400 MHz).

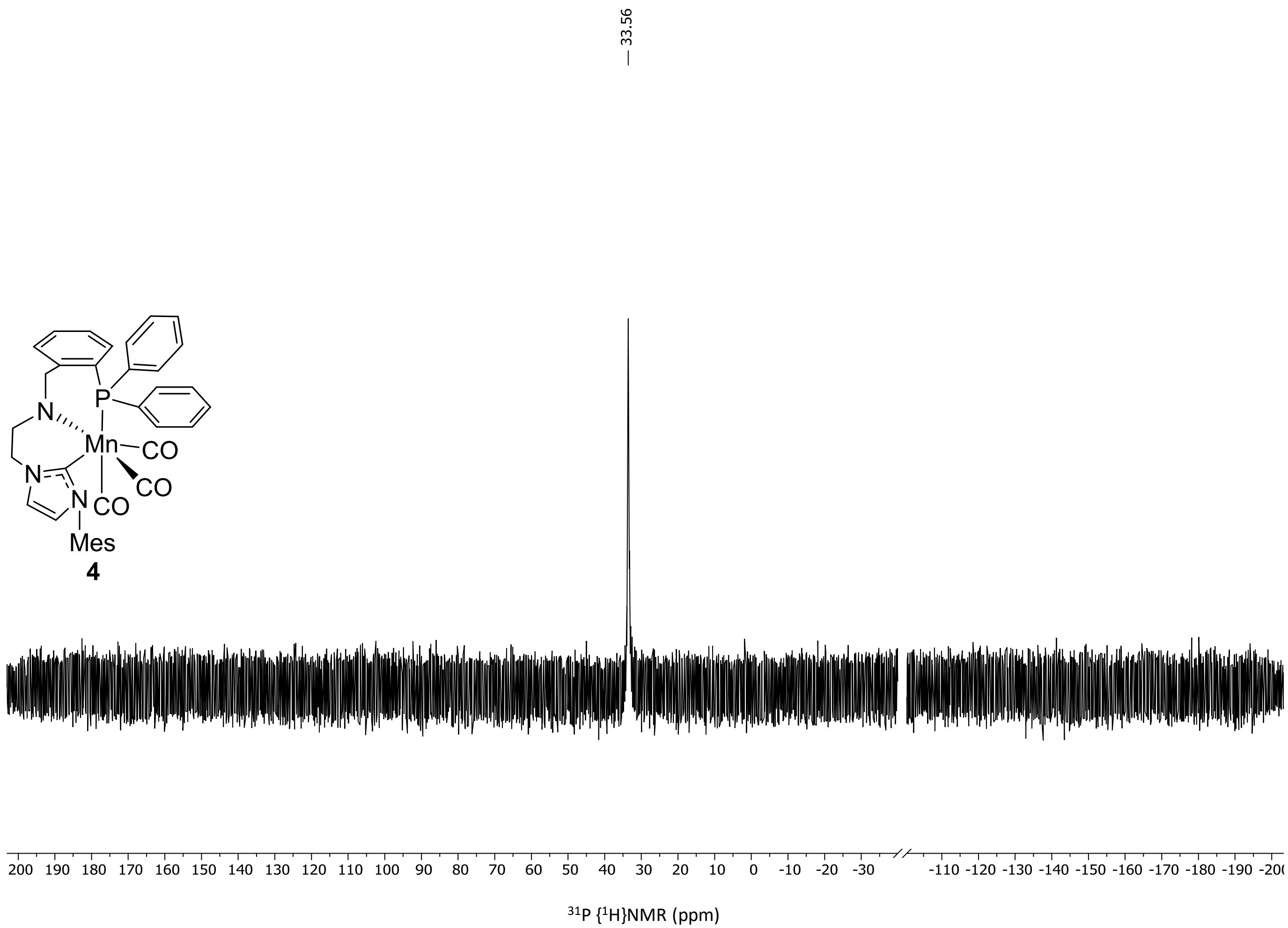

Supplementary Figure 11. ^{31}P {^{1}H}NMR spectrum of complex **4** in THF-d_8 (162 MHz).

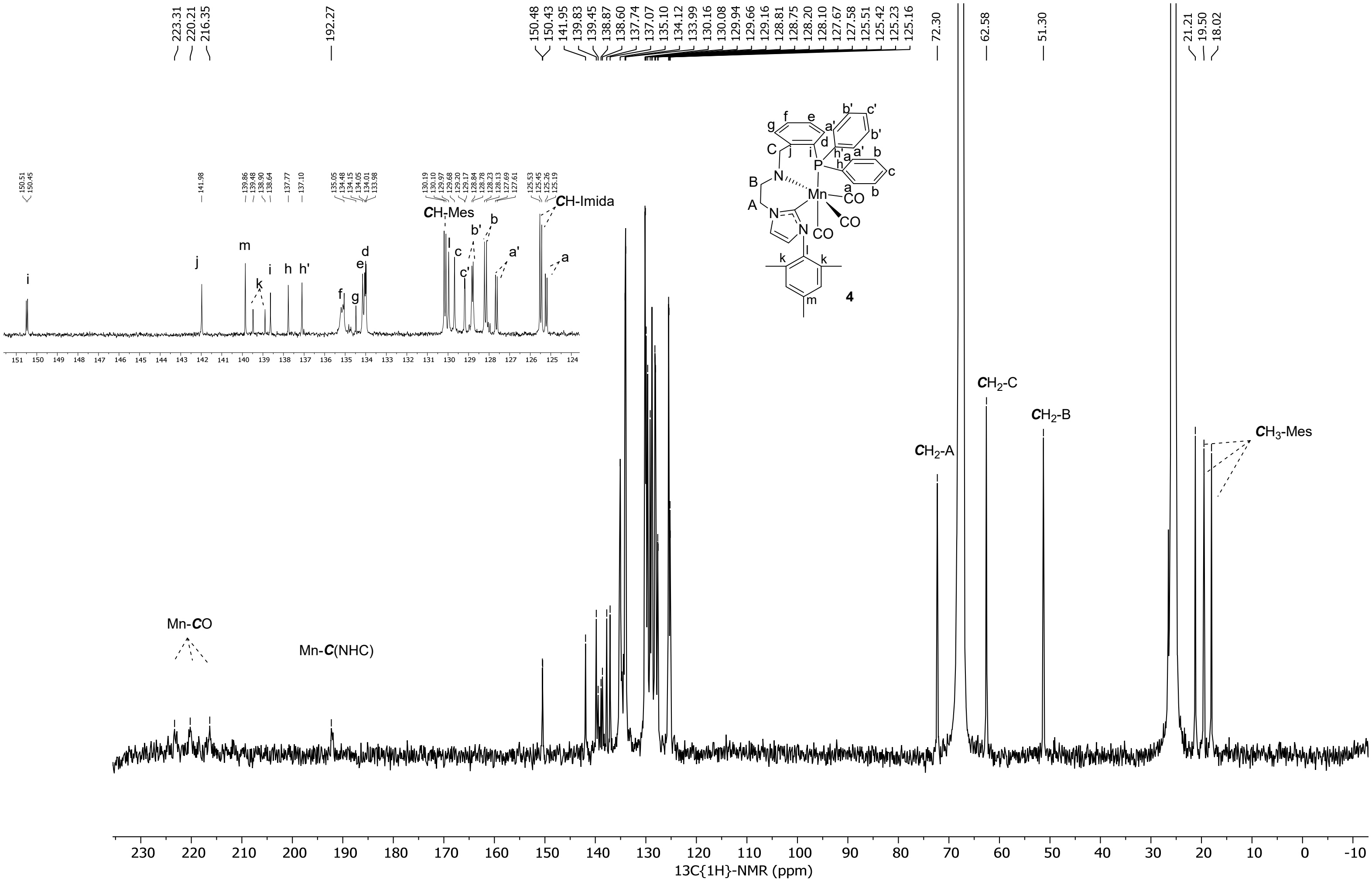

Supplementary Figure 12. ^{13}C $\{^1H\}$NMR spectrum of complex **4** in THF-d_8 (100 MHz).

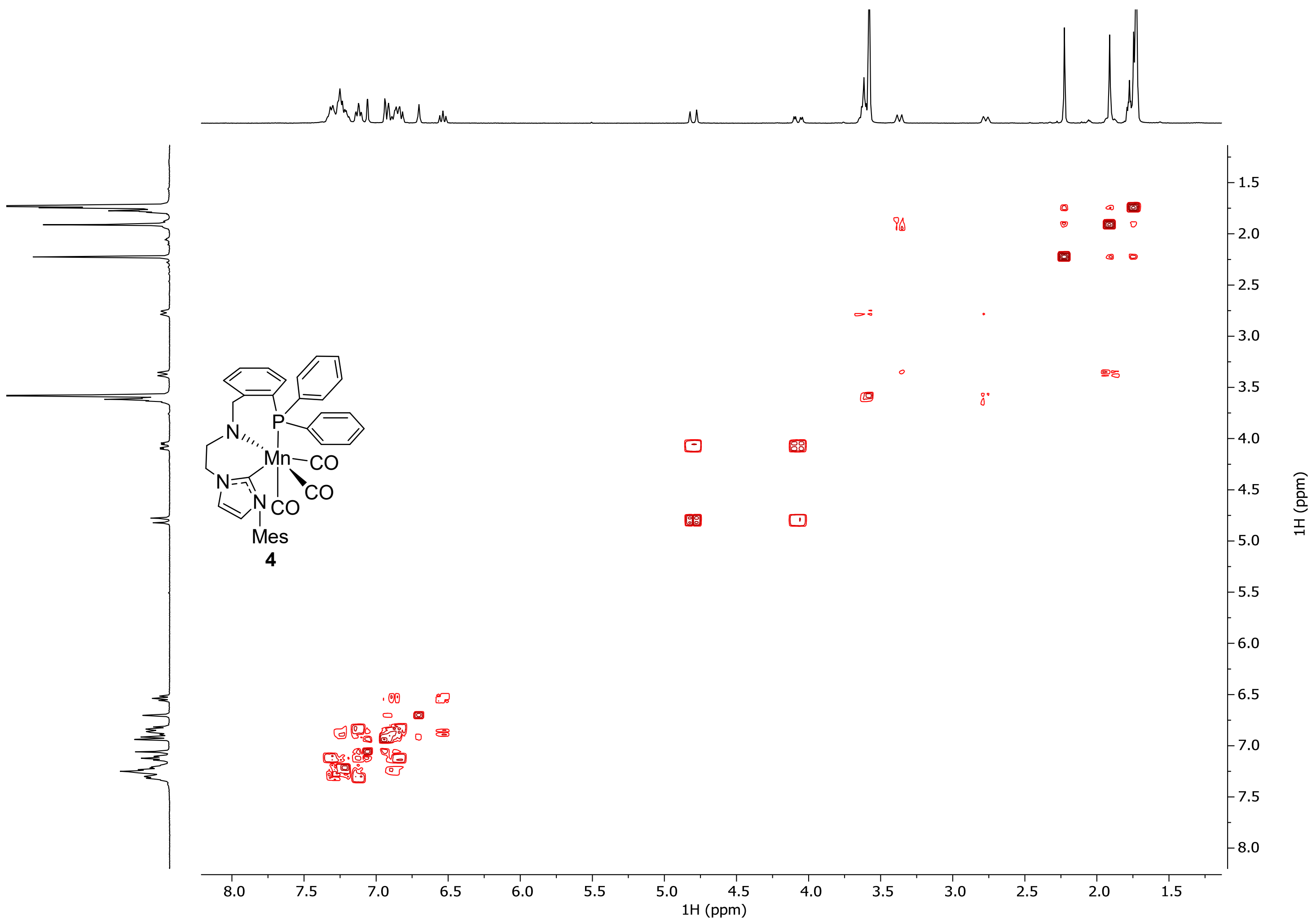

Supplementary Figure 13. gCOSY spectrum of complex **4** in THF-d_8.

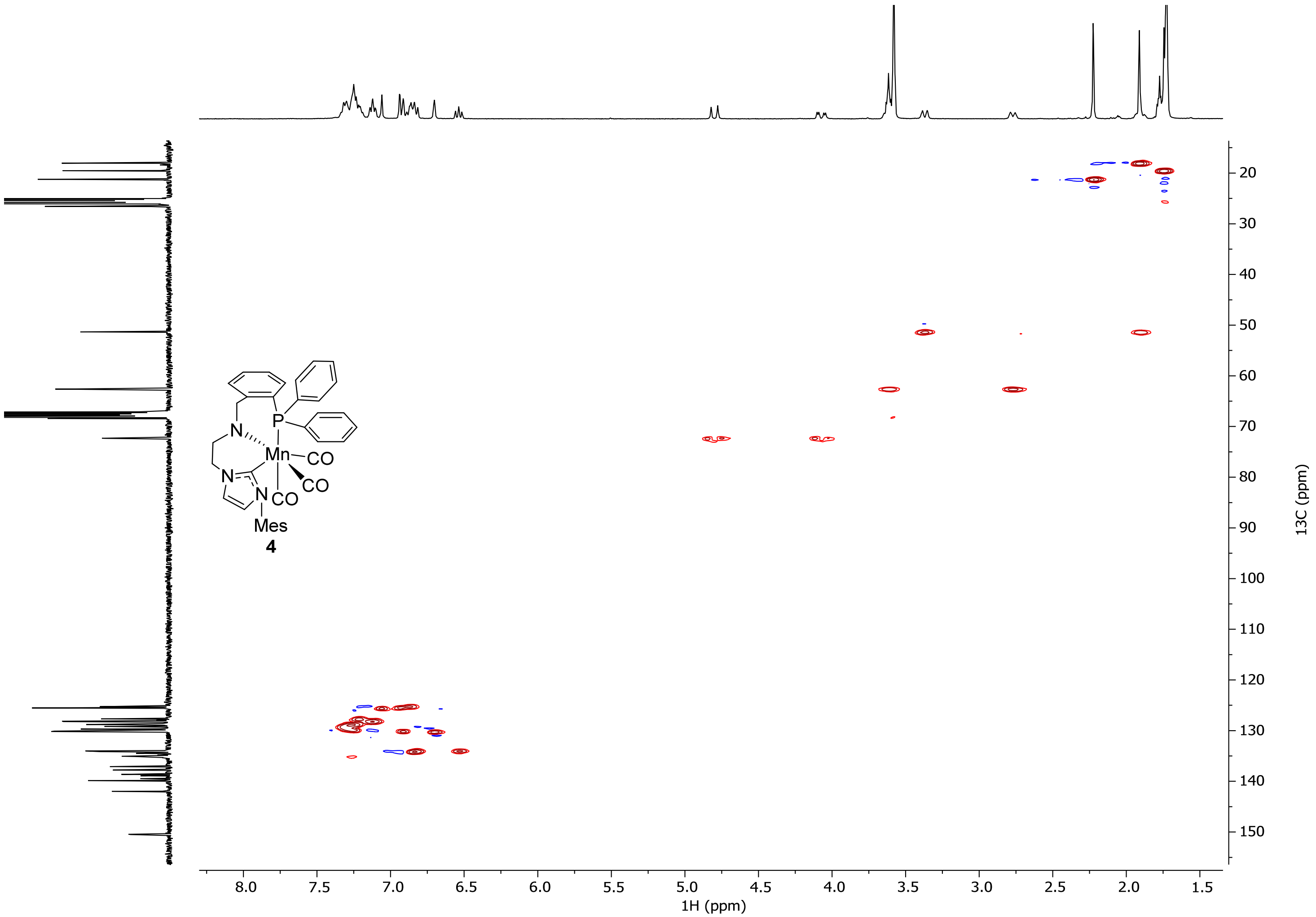

Supplementary Figure 14. HMQC spectrum of complex **4** in THF-d_8.

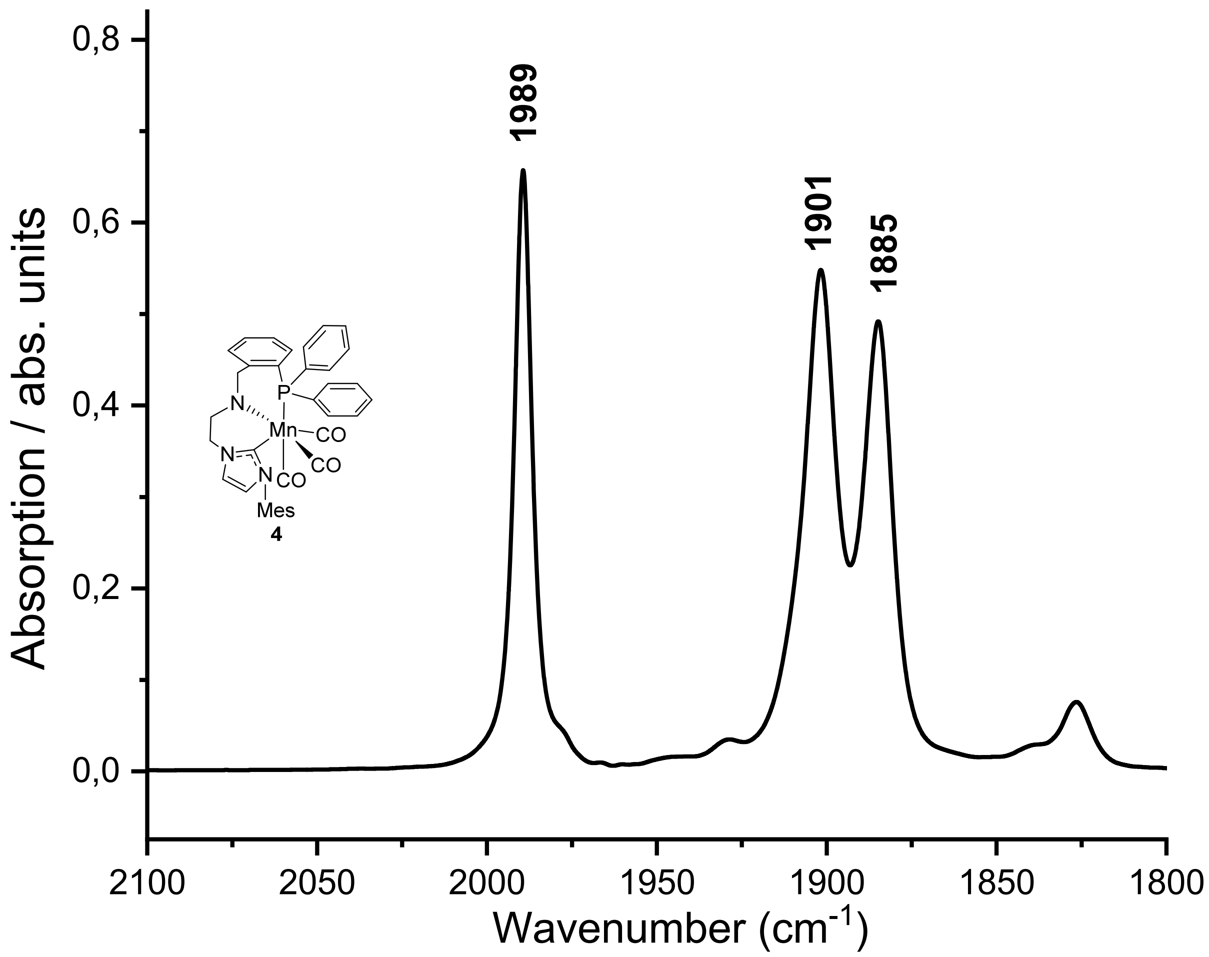

Supplementary Figure 15. IR spectrum of THF solution of complex **4.**

***In situ*-generation of Complex 4:**

Complex **4** was generated upon treatment of complex **3** (7.9 mg, 1 equiv.) with KO^{t}Bu (1,7 mg, 1.5 equiv.) in THF-d_8 (0.6 mL). An orange-red solution was formed immediately followed by NMR measurements. IR analysis was performed through same procedure with protic THF as solvent. **4** (*in situ*): $^{31}P\{^1H\}$NMR (162 MHz, THF-d_8, 297 K): δ = 33.6 (s). IR (solution in THF, 297 K): ῡ [cm^{-1}] 1989 (s, ῡ CO), 1901 (s, ῡ CO), 1885 (s, ῡ CO).

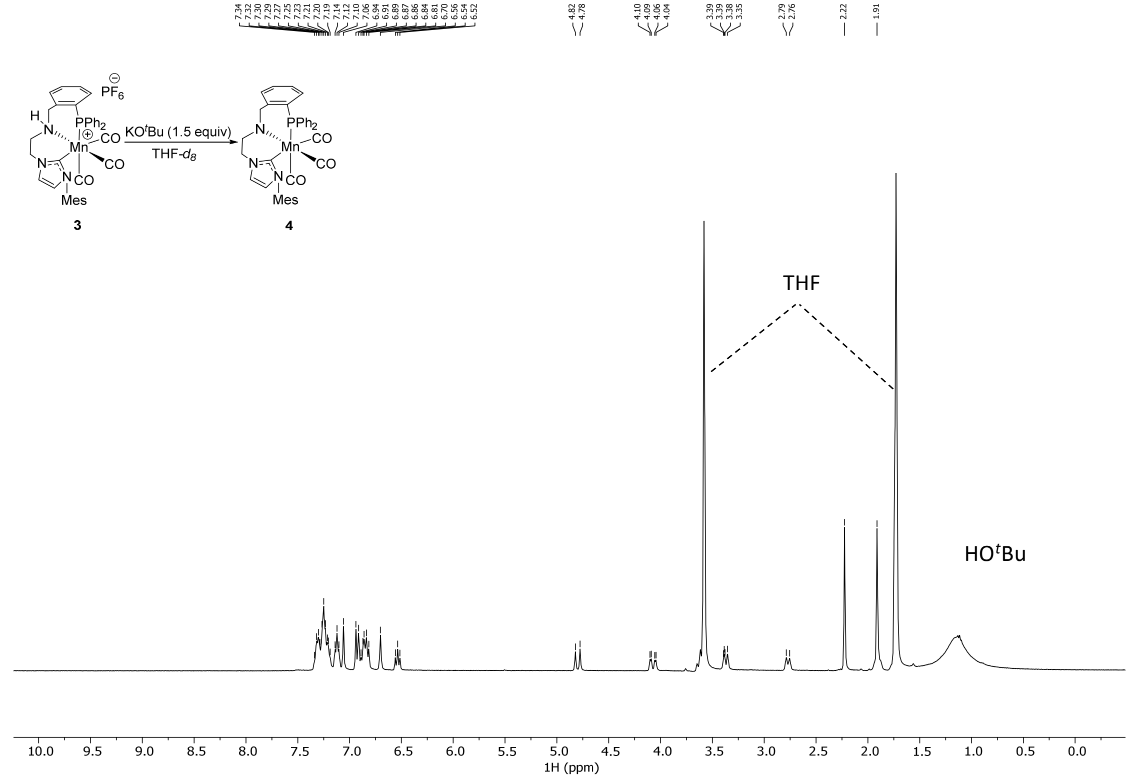

Supplementary Figure 16. ^{1}H-NMR spectrum for activation of **3** with KO^{t}Bu in THF-d_8. Complex **3** was transformed to complex **4** with nearly full conversion.

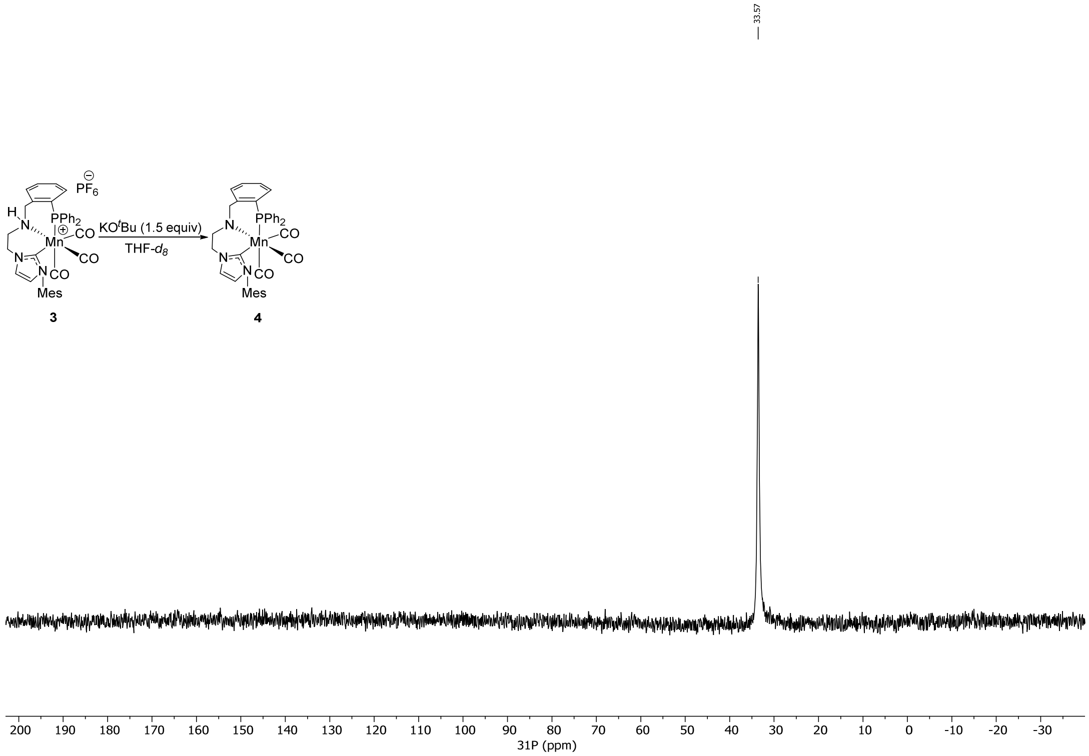

Supplementary Figure 17. ^{31}P {^{1}H}NMR spectrum for activation of **3** with KO^{t}Bu in THF-d$_8$.

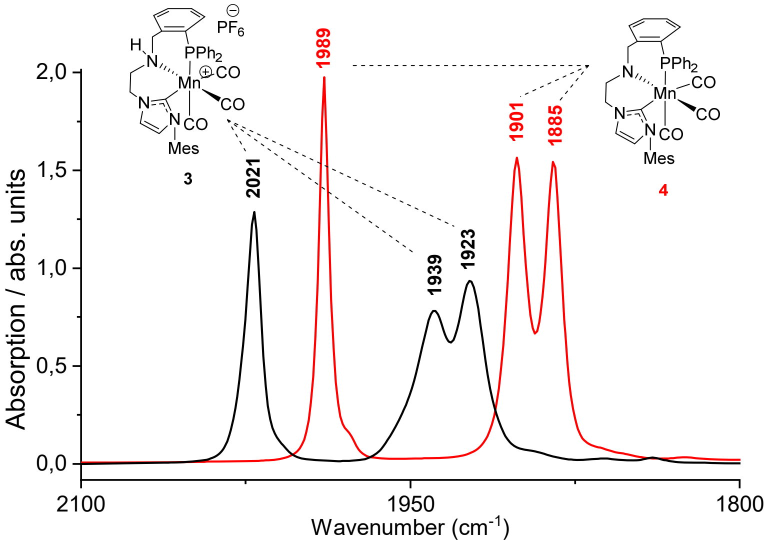

Supplementary Figure 18. IR spectrum of *in situ*-formed **4** (red) and complex **3** (black) in THF. Complex **3** was transformed to complex **4** with nearly full conversion.

***In situ*-generation of Mn-H complex 5:**

The solution of **4** obtained as above was pressurized with 3 bar H_2 and dwelled at 25°C for 12 h. The NMR spectra were collected and the sample was heated at 50 °C for another 12 h to form species **5**. Spectra given in Supplementary Figures 19-20. Full assignment was not possible due to incomplete conversion of **4** into **5**. The assignments were done with support of DFT calculations (Supplementary Figures 41, 42, 44) and IR spectroscopy.

5: ^{1}H NMR (400 MHz, THF-d_8, 297 K, hydride resonances): δ -3.46 (d, $^{2}J_{PH}$ 60.0 Hz, Mn-H), -3.49 (d, $^{2}J_{PH}$ 68.0 Hz, Mn-H); ^{31}P{^{1}H}NMR (162 MHz, THF-d_8, 297 K) δ = 81.1 (s). IR: (solution in THF, 297 K): ῡ [cm^{-1}] 1897 (s, ῡ CO, DFT estimated, overlaps with band of **4**), 1814 (s, ῡ CO).

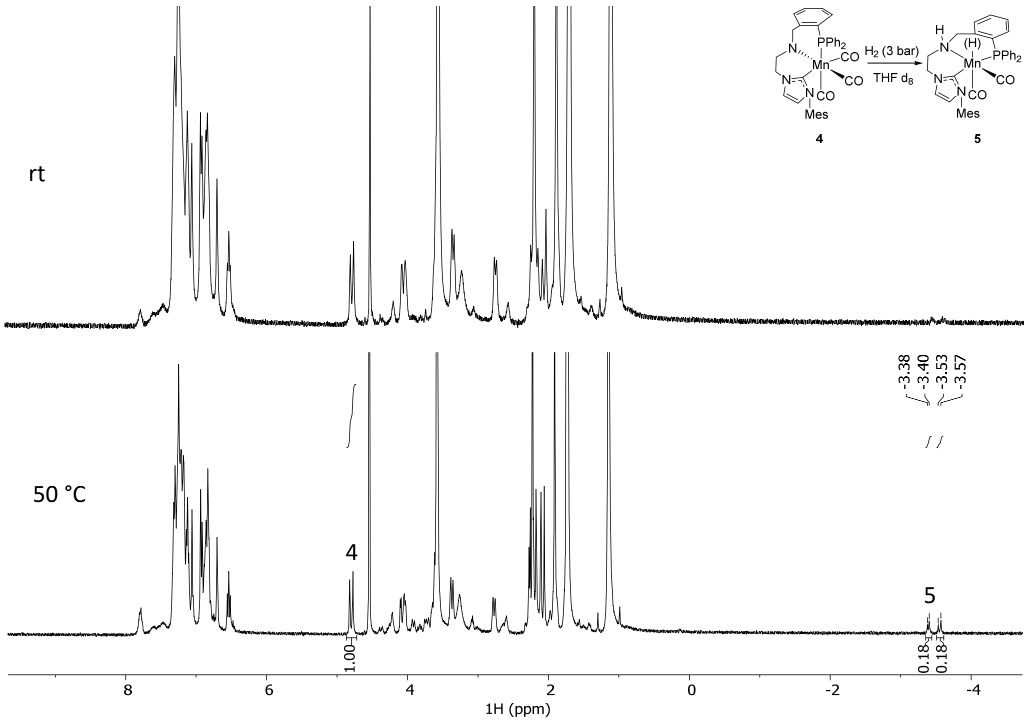

Supplementary Figure 19. ^{1}H-NMR spectrum of reaction mixture of in situ formed **4** with H_2 in THF-d_8 at room temperature (top, 12 h incubation time) and 50 °C (bottom, 12h incubation time). Reaction with H_2 at room temperature led to formation of trace amounts of hydride and heating at 50 $^{\circ}$C resulted in ca. 24% conversion. The conversion of **5** was derived by comparing integration of hydride peaks (-3.46, -3.49 ppm) with the methylene resonance (4.80 ppm) of complex **4**.

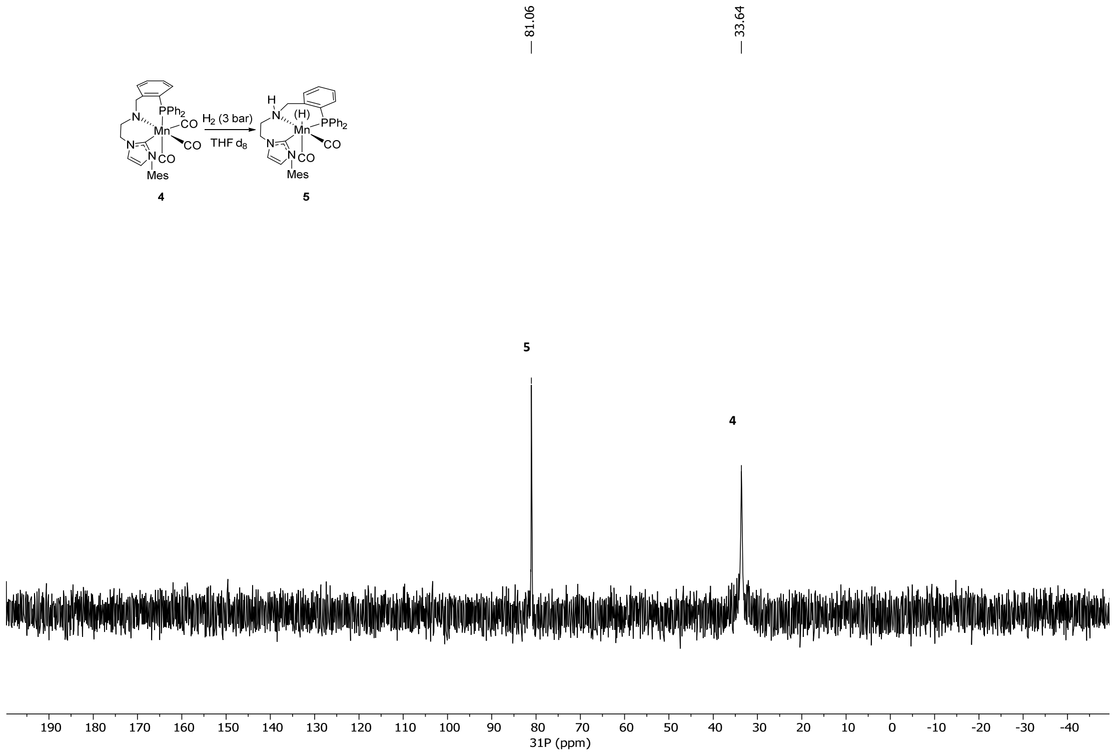

Supplementary Figure 20. $^{31}P\ \{^{1}H\}$NMR spectrum of reaction mixture of in situ formed **5** with H_2 in THF-d_8 at 50 °C for 12 h.

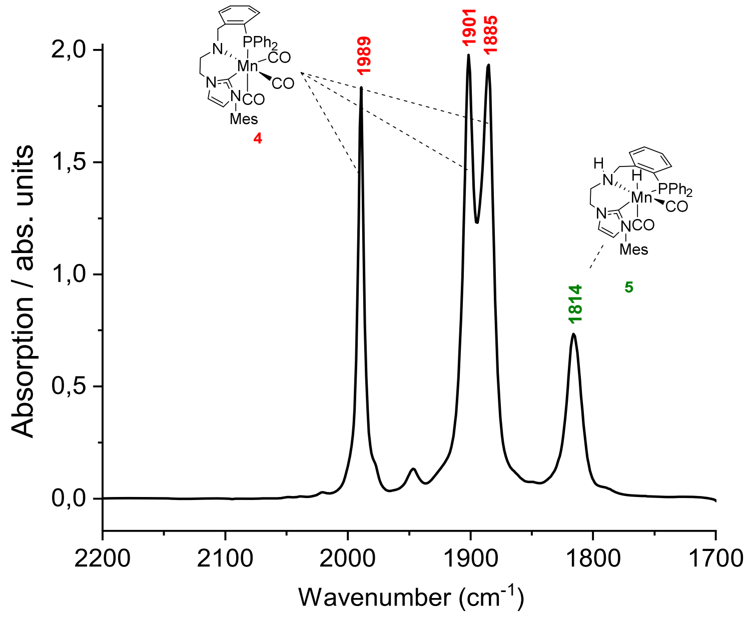

Supplementary Figure 21. IR spectrum of reaction mixture of in situ formed **4** with H_2 in THF at 50 °C for 12 h. Complex **4** was partially converted to hydride **5**. The other CO band of **5** was overlapped with that of complex **4**.

***In-situ* generation of hydride complex via activation with $KHBEt_3$:**

Upon treatment of complex **3** (7.9 mg, 1 equiv.) with $KBHEt_3$ (25 µl of 1M THF solution, 2.5 equiv.) in THF-d_8 (0.6 mL) an orange solution was formed immediately producing the mixture of complex **4** and hydride complexes **5** and **6**. The resulting solution was monitored by NMR immediately after borohydride addition and at 10, 15, 20, 25, 30 min and 12 h time.

Subsequently, three measurements after addition of 1, 0.1, and 1 equiv. of acetophenone respectively were then added sequentially to probe the reactivity of hydride species. Corresponding IR analysis was performed through same procedure with protic THF as solvent.

6: ^1H NMR (400 MHz, THF-d_8, 297 K, partial, hydride resonances only): δ -3.92 (s, Mn-H, **6a**), -4.40 (s, Mn-H, **6b**); FTIR-ATR (solution in THF, 297 K): ῡ [cm^{-1}] 1981 (s, ῡ CO), 1891 (s, ῡ CO, estimated), 1868 (s, ῡ CO). Assignment made on the basis of DFT calculations (Supplementary Figures 41, 42, 45).

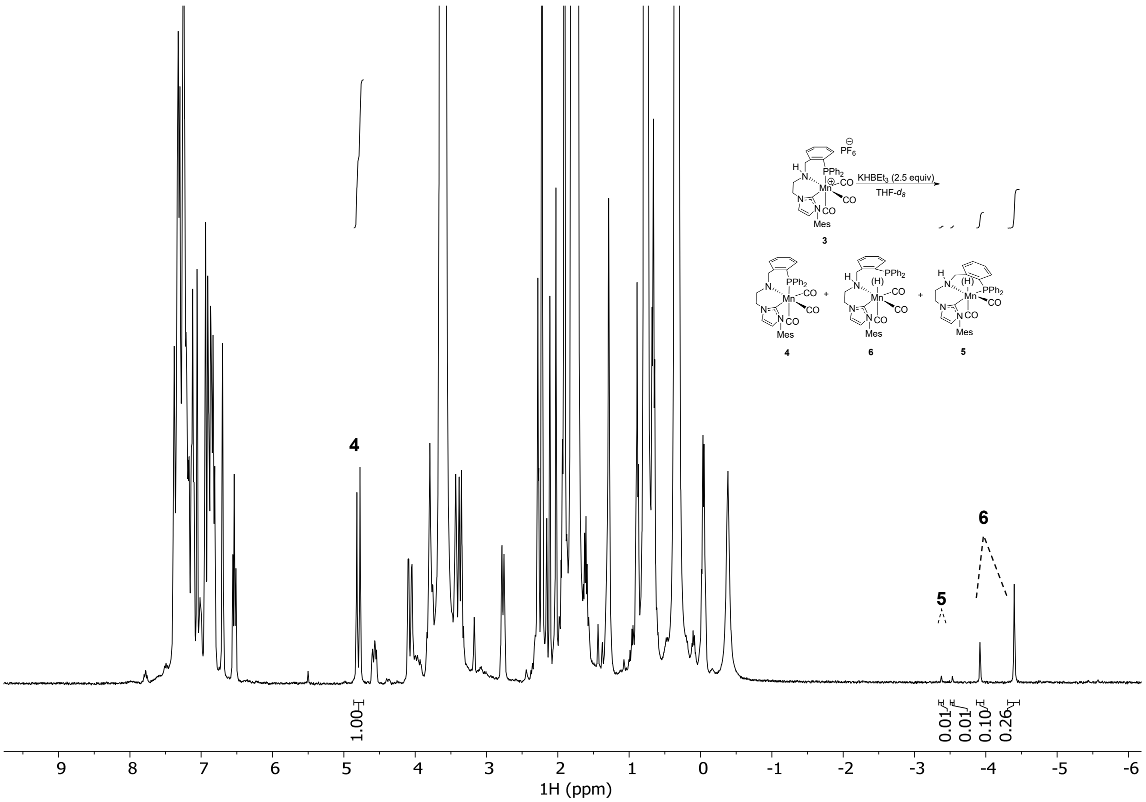

Supplementary Figure 22. ^1H-NMR spectrum of $KBHEt_3$-activated **3** in THF-d_8 immediately after reaction. Deprotonated complex **4**, bidentate Mn-H complex **6** with free phosphine were formed as major products. Additionally, hydride from complex **5** was also formed in trace amount. Integration shown in Supplementary Figure 22 estimates the fraction of hydride species at ca. 31% with the rest being complex **4**.

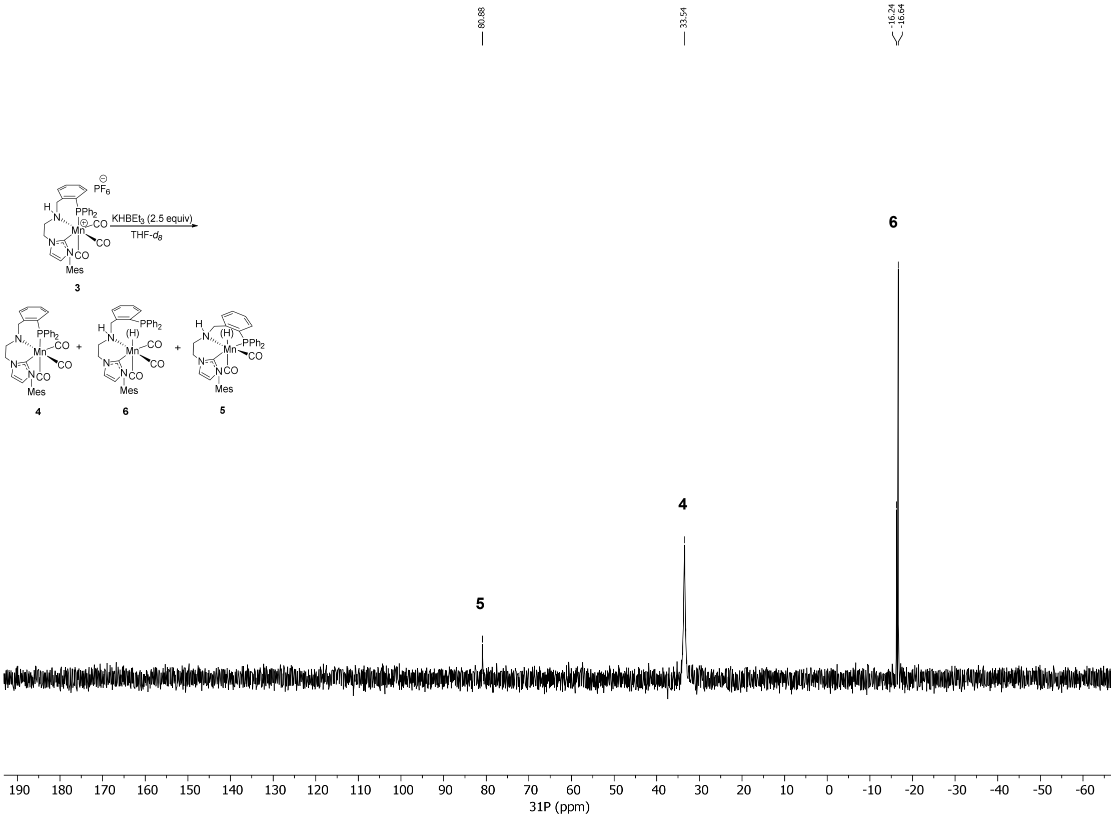

Supplementary Figure 23. $^{31}P\ \{^{1}H\}$NMR spectrum of $KBHEt_3$-activated **3** in THF-d_8 immediately after reaction.

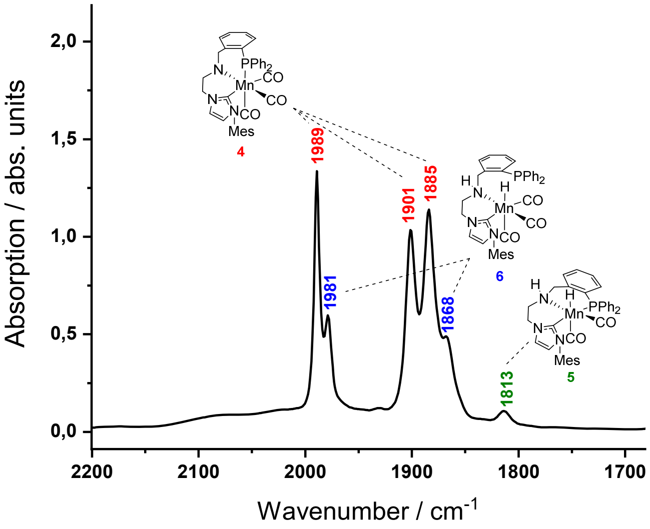

Supplementary Figure 24. IR spectrum of $KBHEt_3$-activated **3** in THF immediately after reaction.

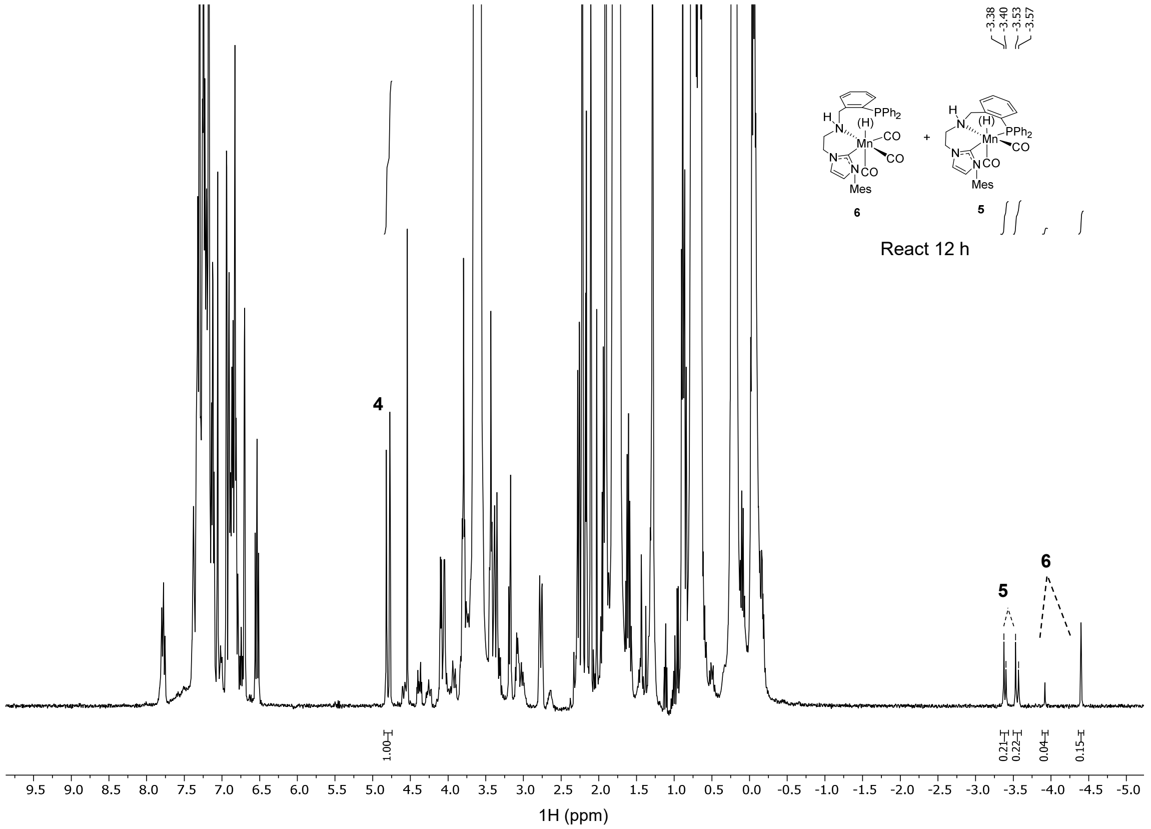

Supplementary Figure 25. ^{1}H-NMR spectrum of KBHEt$_3$-activated **3** (12 h) in THF-d_8. After 12 h, complex **6** was partially consumed, and more complex **5** was formed. The total content of hydride species was ca. 37% with the rest being complex **4**.

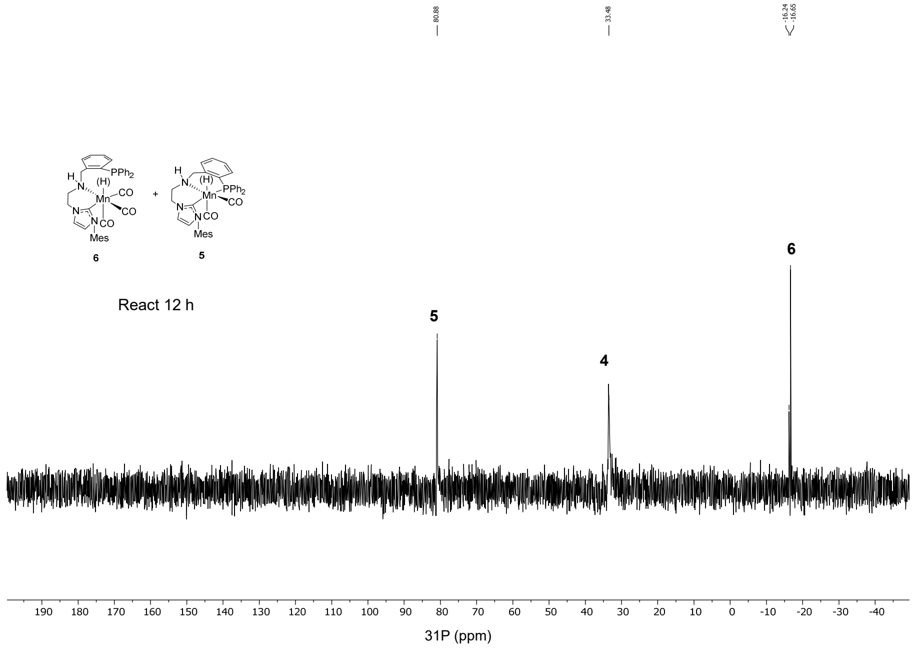

Supplementary Figure 26. ^{31}P {^{1}H}NMR spectrum of KBHEt$_3$-activated **3** (12 h) in THF-d_8.

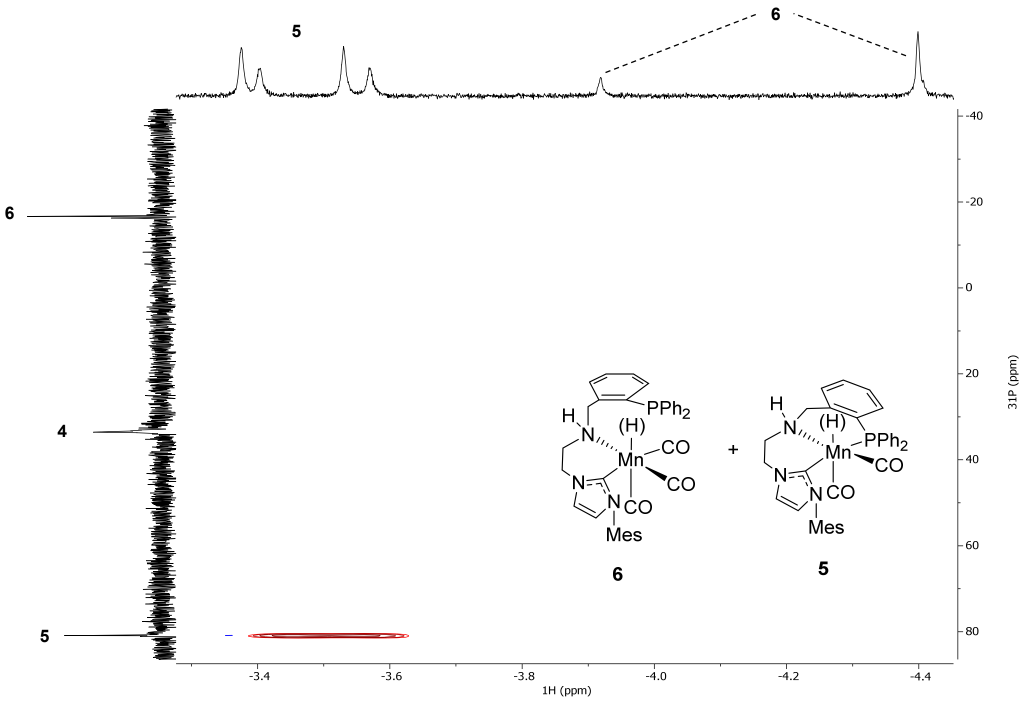

Supplementary Figure 27. ^{1}H-^{31}P heteronuclear HMQC spectrum of $KBHEt_3$-activated **3** (12 h) in THF-d_8. Note the presence of P-hydride cross-peak for **5** and the absence of one for **6** consistent with unbound nature of P-arm in **6**.

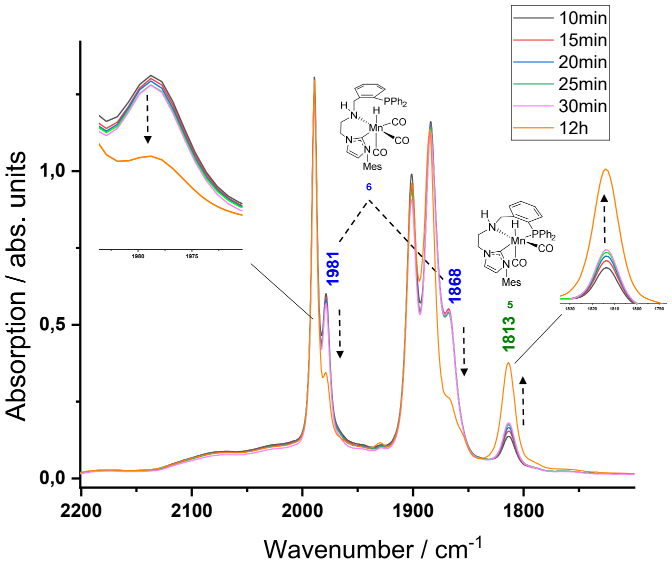

Supplementary Figure 28. Evolution of IR spectrum of **3** in THF after $KBHEt_3$ activation for 10 min, 15 min, 20 min, 25 min, 30 min and 12 h reaction times. Note the decrease of IR bands of complex **6** along with the increase of bands of complex **5** indicating conversion of **6** to **5**.

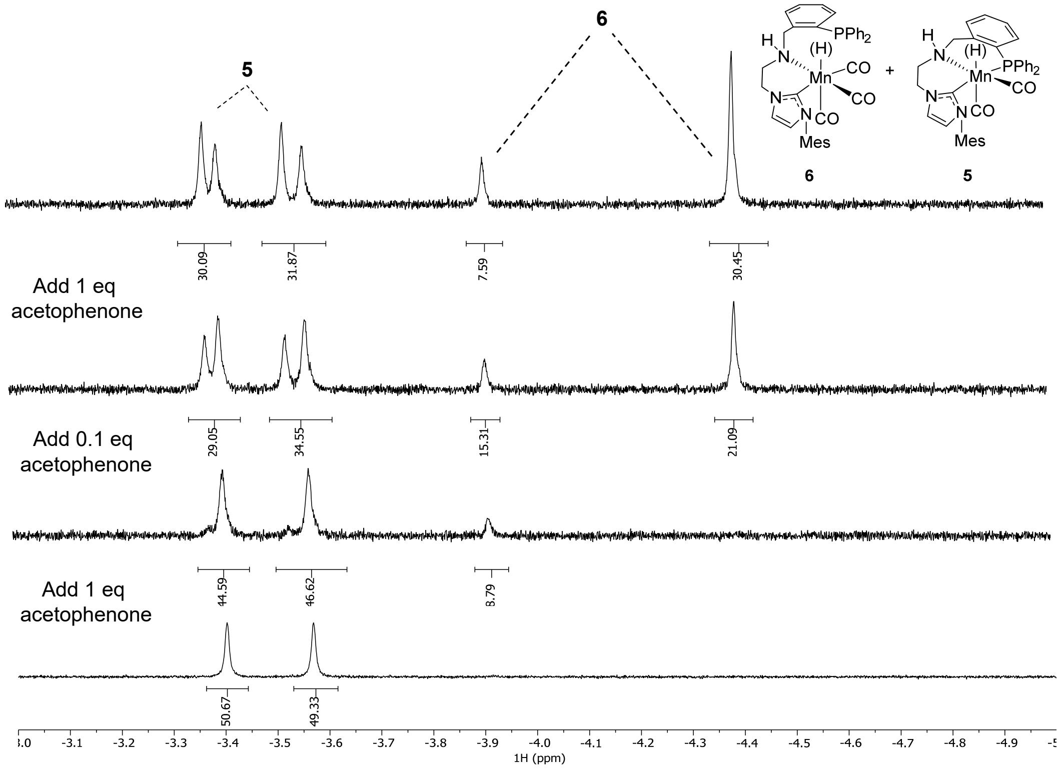

Supplementary Figure 29. ^{1}H-NMR spectrum (hydride region) of in situ-formed mixture of **5** and **6** (12 h) and its reaction with acetophenone in THF-d_8. The order of hydride consumption is indicating higher reactivity of **6** towards ketone substrates.

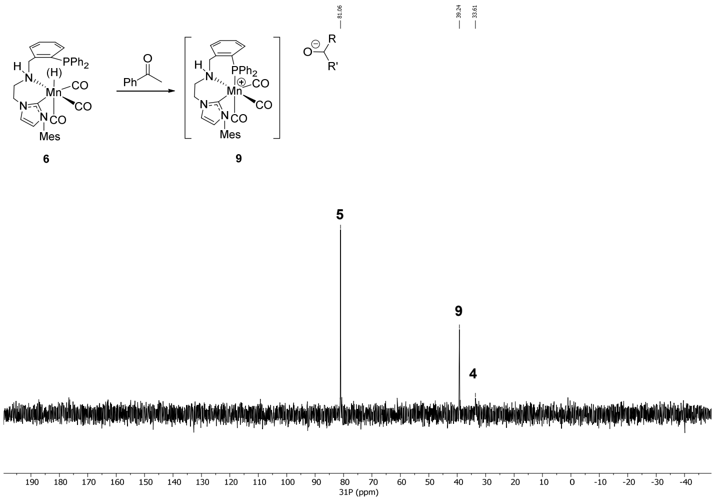

Supplementary Figure 30. ^{31}P {^{1}H}NMR spectrum of reaction mixture of in situ-formed Mn-H **5**, **6** (12 h) with 2 eq. acetophenone in THF-d_8. Structure of complex **9** is putative and suggested by the IR data in Supplementary Figure 31.

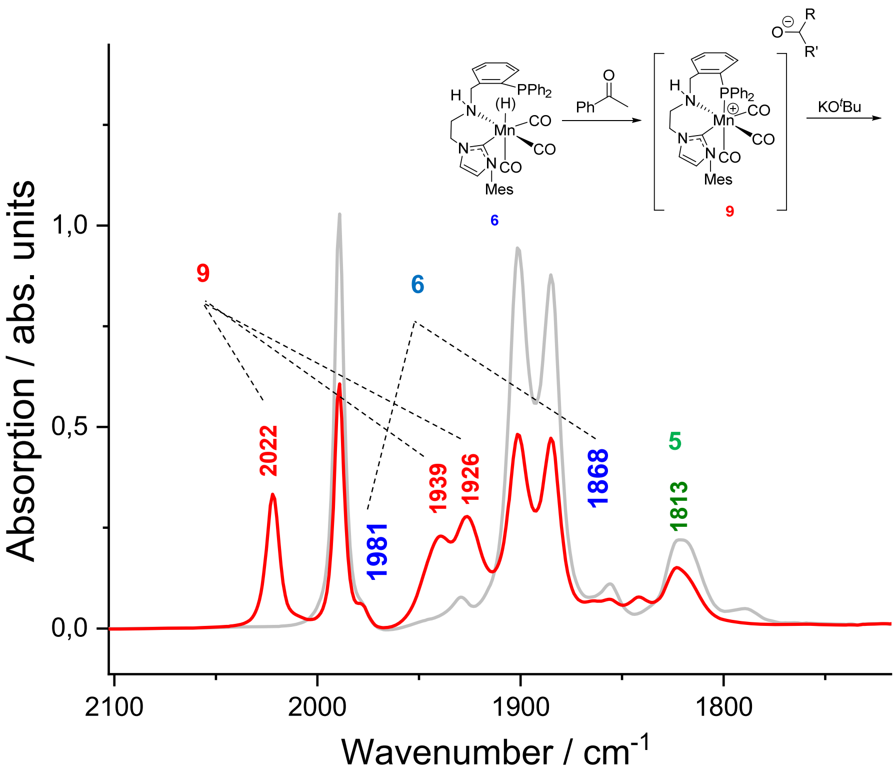

Supplementary Figure 31. Evolution of IR spectrum upon acetophenone treatment of mixture of **5** and **6** (red) and subsequent addition of KO^{t}Bu (grey) in THF. Note the formation of new set of carbonyl resonances (labelled as species **9**) indicative of cationic tricarbonyl complex. Deprotonation and regeneration of amido complex **4** is further noted upon treating the reaction mixture with KO^{t}Bu (grey).

Supplementary Note:

While it is not possible to confirm the identity of proposed complex **9** unambiguously, the IR data and ^{31}P NMR data point to the cationic nature of this tricarbonyl complex. The lack of hydride ligand is indicative of the hydride transfer taking place as well as coordination of P-donor arm that was unbound in the starting material **6**.

NMR tube scale hydrogenation of acetophenone with $KHBEt_3$ activated complex 3:

The hydride region data is depicted in Figure 4a of the manuscript. The same activation sequence as described above for complex **3** (7.9 mg) was used to generate complex **6** (30%) in the NMR tube in the mixture containing complex **4** (67%). The mixture was immediately reacted with acetophenone (2 equiv.), allowed to incubate for 30 minutes and NMR spectrum was recorded (Supplementary Figures 32, 33). Nearly full consumption of the hydride **6** was observed. Immediately afterwards the tube was pressurized with H_2 gas (3 bar) and set aside at room temperature for 1 hour before the NMR spectrum was recorded (Supplementary Figures 34, 35). Acetophenone substrate was completely consumed and a partial recovery of species **6** was detected together with formation of complex **5**.

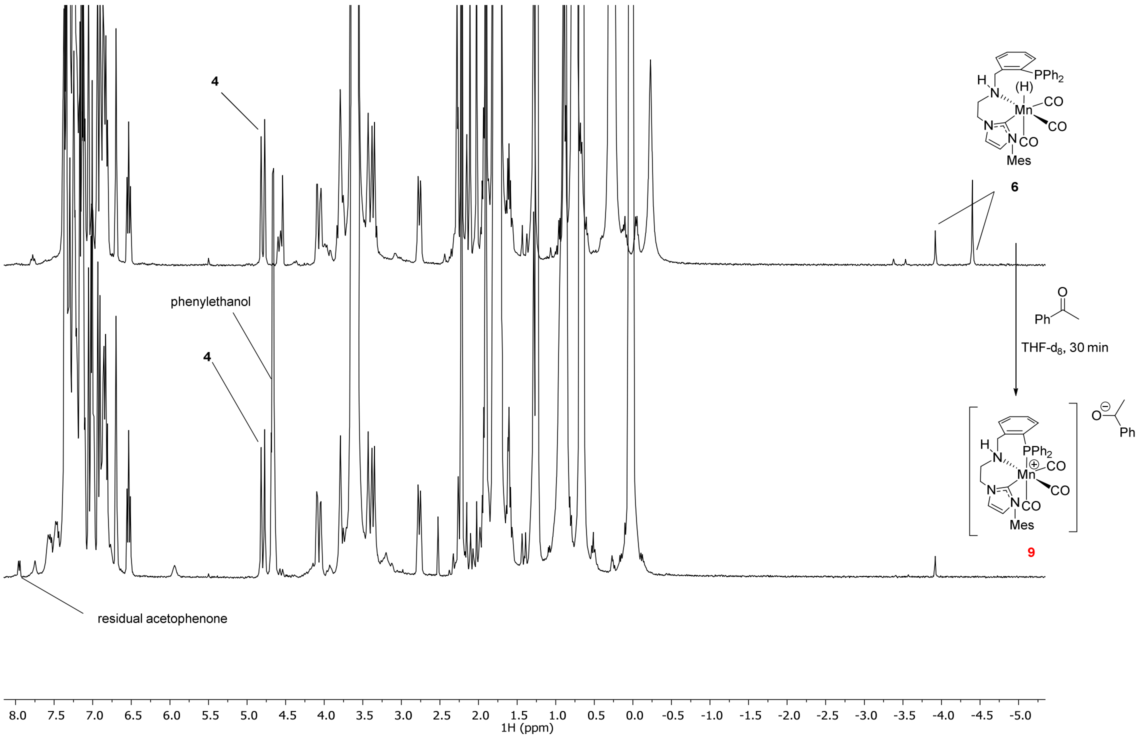

Supplementary Figure 32. ^{1}H-NMR spectrum of reaction mixture of $KBHEt_3$-activated **3** (see Supplementary Figure 22) contacted immediately with 2 eq. acetophenone and incubated for 0.5 h. The main isomer of **6** at -4.4 ppm was fully consumed.

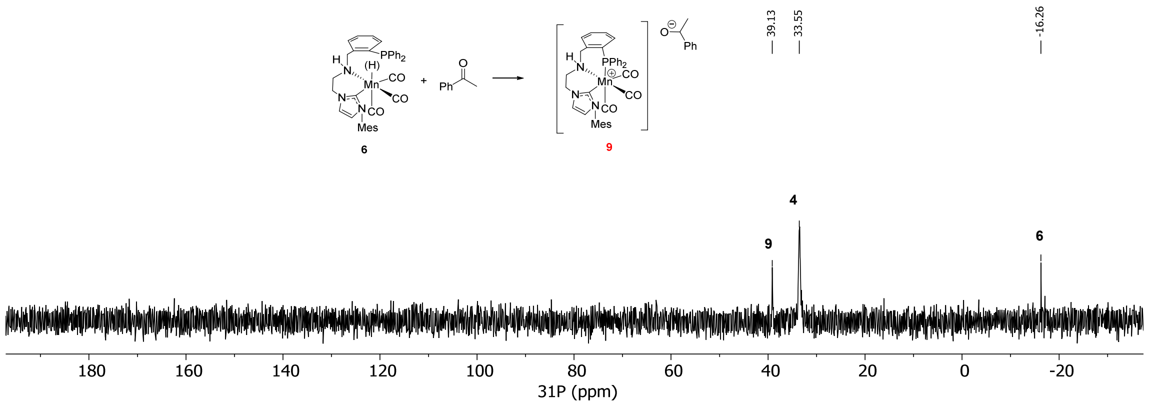

Supplementary Figure 33. $^{31}P\ \{^1H\}$NMR of reaction mixture of $KBHEt_3$-activated **3** contacted immediately with 2 eq. acetophenone and incubated for 0.5 h.

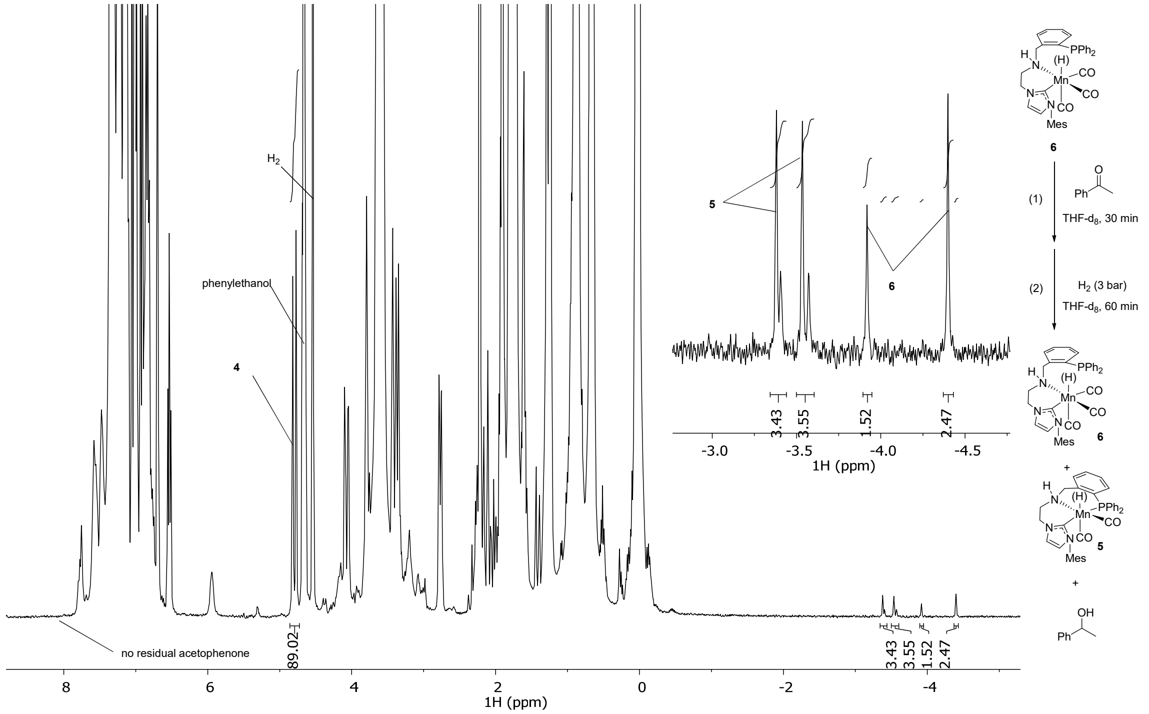

Supplementary Figure 34. ^1H-NMR spectrum of mixture generated as described above contacted with acetophenone and treated with 3 bar H_2 for 1 h. The main isomer of **6** at -4.4 ppm was regenerated and dicarbonyl Mn-H **5** was accumulated through catalytic turnover. Total content of hydride species upon treatment with hydrogen was 10.75% compared to 37% obtained before the reaction with acetophenone (Supplementary Figures 22 and 32)

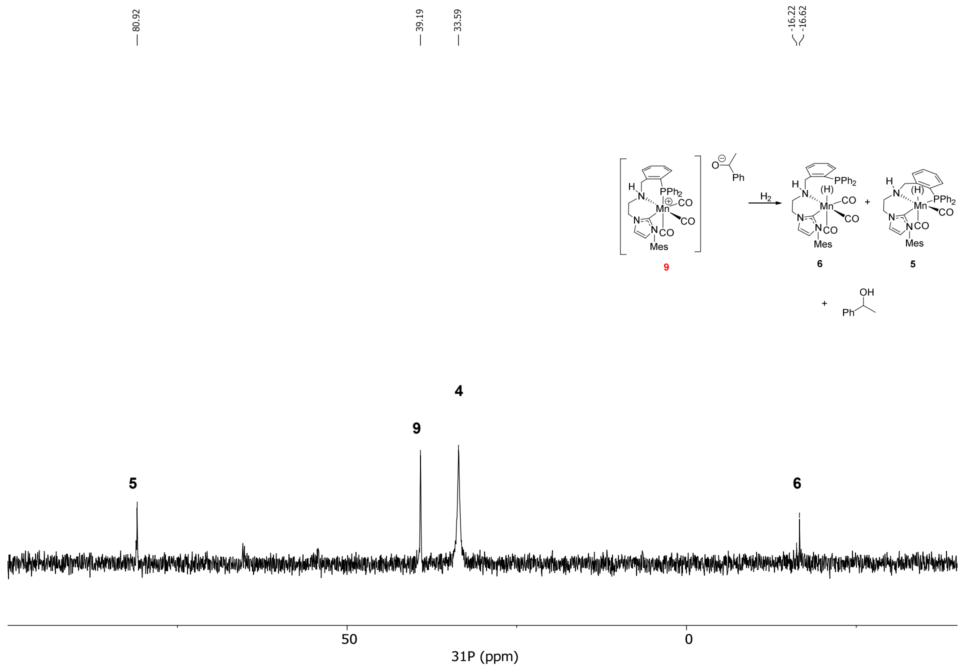

Supplementary Figure 35. ^{31}P {^{1}H}NMR spectrum of mixture described in Supplementary Figure 33 treated with 3 bar H_2 for 1 h.

General procedure of Catalytic Hydrogenation:

Liquid substrates were passed through a plug of neutral alumina, degassed and stored over molecular sieves in the glove box. Mn catalysts, $KBHEt_3$ and KO^tBu, substrates and solvents were handled in the glovebox. Kinetic study were done with a autosampler setup.[3]

For initial experiments and optimisation, products were analyzed on an Agilent 6890 gas chromatograph equipped with an FID detector. Method details: Agilent CP-Chirasil-Dex CB column (25 m, 0.25 mm ID, 0.25 μm film thickness) and temperature profile from 120 °C (hold 1 min) up to 180 °C with ramp of 20 °C/min and hold at 180 °C for 2 minutes. Mass balances were verified to be within 90 % – 110 % for all experiments. Products were identified using retention times and peak areas from analytically pure reference samples.

The real-time hydrogen pressure during hydrogenations was recorded and transformed to corresponding H_2 consumption (mmol) via ideal gas law (PV = nRT).

Catalytic hydrogenation with Mn catalyst 3:

Stock solutions of **3** (0.01 M) were prepared. KO^tBu (5.6 mg, 1 mol%), substrate (5 mmol), solvent (3 mL), dodecane (56.8 μL, 0.25 mmol), complex **3** (250, 500, 1000 μL for S/Mn 20000, 10000, 5000, 4000 respectively) were mixed into a 4 mL glass vials and transferred into a stainless steel autoclave in the glovebox. The system was purged with N_2 (3×8 bar) and H_2 (1×30 bar), pressurized with H_2 to specified pressure, and heated to specified temperature. After the reaction, resulting mixture was quenched with HCl aqueous (50 μL, 1 M) and then GC samples were prepared by dilution of the reaction mixture in THF (20 μL into 1 mL THF).

For the reaction with $KBHEt_3$, stock solution of **3** (0.01 M) was prepared in 0.9 mL dioxane and activated with 0.1 mL $KBHEt_3$ solution (1M in THF) for 0.5 h. Then 25, 37.5, 45, 47.5 μL of $KBHEt_3$ solution and 250, 125, 50, 25 μL of complex **3** were used for catalysis at 50, 25, 10, and 5 ppm catalyst loadings respectively.

Kinetic study of acetophenone hydrogenation with Mn catalyst 3 on a large scale:

Inside glovebox, a stock solution of **3** (0.0125 M) was prepared in 0.875 mL dioxane and activated with 0.125 mL $KBHEt_3$ solution (1M in THF) for 0.5 h. A 1mL syringe was loaded with complex **3** (500, 250, 100, 50 μL) and $KBHEt_3$ solution (62.5, 93.8, 112.5, 118.8 μL) in dioxane (total volume is 0.7 mL), and a 20 mL syringe was loaded with acetophenone (1.460 mL, 12.5 mmol) and dodecane (113.6 μL, 0.625 mmol) in 10 mL dioxane. Under N_2 flow, the substrate syringe was first injected into high pressure stainless steel reactor in which a glass beaker was inserted in advance. In an injection port the dissolved catalyst was then placed. The system was purged with H_2 (3×10 bar). The reaction was carried out at 120 °C, 50 bar H_2 pressure with stirring at 500 rpm. The samples of different time were taken via a tubing reach into the reaction mixture. Data plotted in Figure 4 of the manuscript.

Optimization of Reaction Conditions.

Supplementary Table 1. Screening of solvent and base amount for acetophenone hydrogenation with **3**.[a]

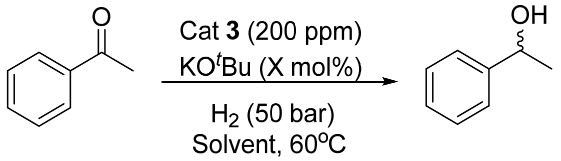

Entry	solvent	X (mol%)	t (h)[b]	Y_{alc} (%)[c]
1	Dioxane	1	8	99
2	Diethyl ether	1	4	99
3	IPA	1	8	99
4	Toluene	1	23	45
5	MeCN	1	20	0
6	MeOH	1	20	13
7	THF	1	23	99
8	MTBE	1	8	99
9	Dioxane	1	6	99
10	Dioxane	0.5	6	99
11	Dioxane	0.1	6	99
12	Dioxane	0.05	6	99
13	Dioxane	0.04	7	5
14	Dioxane	0.03	7	3
15	dioxane	0.02	7	TRACE

[a] Reactions were conducted with acetophenone (5 mmol), Mn catalyst **3** (200 ppm), KOtBu (1 mol%) in solvent (3 mL) at 60°C under 50 bar H_2. [b] Total reaction time and that of GC analysis, for H_2 uptake traces see Supplementary Figure 36. [c] Yield determined by GC with dodecane as internal standard.

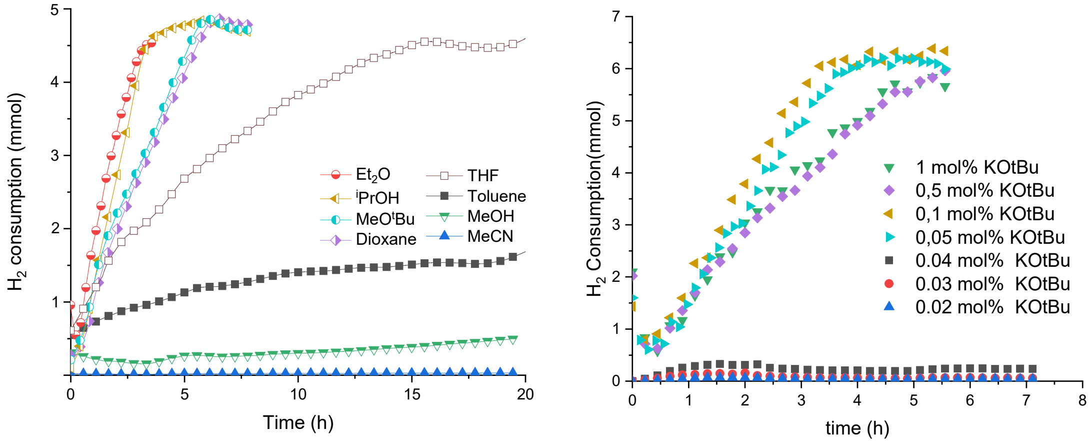

Supplementary Figure 36. H_2 consumption traces for acetophenone hydrogenation with **3** at different solvents (left) and base amounts (right) for the data in Supplementary Table 1.

Supplementary Table 2. Effect of $KBHEt_3$ loading and H_2 pressure on the catalytic activity of **3**.[a]

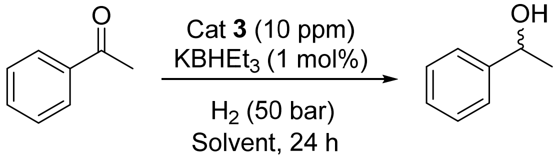

entry	X (mol%)	H_2 pressure (bar)	t (h)[b]	Y_{alc} (%)[c]
1	1	50	10	99
2	0.5	50	10	99
3	0.1	50	17	99
4	0.05	50	17	24
5	1	40	18	99
6	1	30	18	99
7	1	20	18	99
8	1	10	18	61
9[d]	1	50	18	0

[a] Reactions were conducted with acetophenone (5 mmol), Mn catalyst **3** (10 ppm), $KHBEt_3$ (X mol%) in 1,4-dioxane (3 mL) under varied H_2 pressure at 120°C; [b] Total reaction time and that of GC analysis, for H_2 uptake traces see Supplementary Figure 37. [c]Yield determined by GC with dodecane as internal standard. [d]5 bar of CO was added to the system with H_2 at the total pressure of 50 bar.

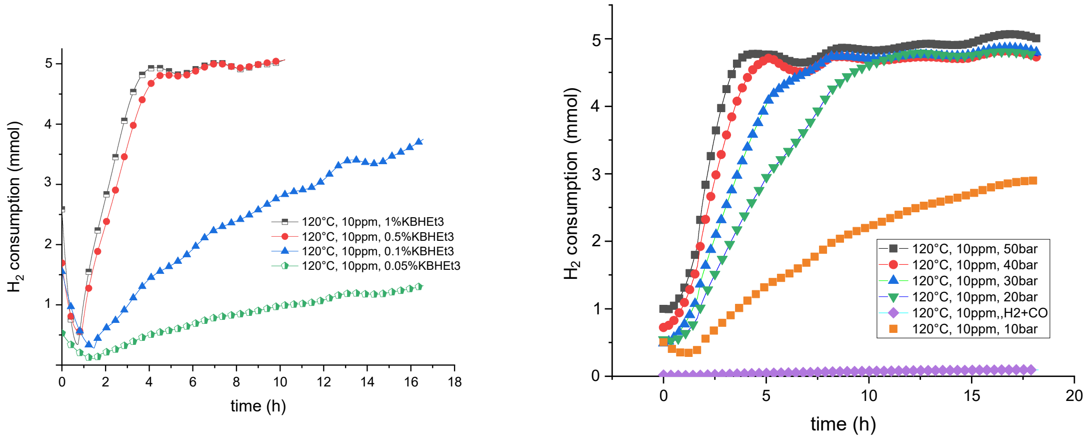

Supplementary Figure 37. H_2 consumption traces for $KBHEt_3$ promoted acetophenone hydrogenation with **3** at different base amounts (left) and pressures (right) used for selection of optimal $KBHEt_3$ amount and H_2 pressure. Data is listed in Supplementary Table 2.

Hydrogen Consumptions traces

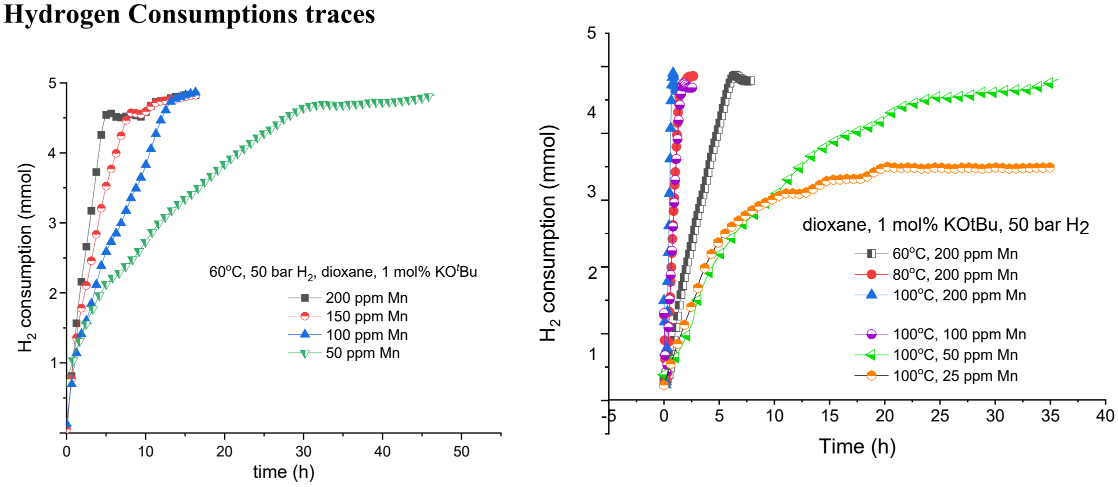

Supplementary Figure 38. H_2 consumption traces of KO^{t}Bu promoted acetophenone hydrogenation with **3** at different catalyst loadings (left) and temperatures (right) for experiments listed in Table 1 of the manuscript.

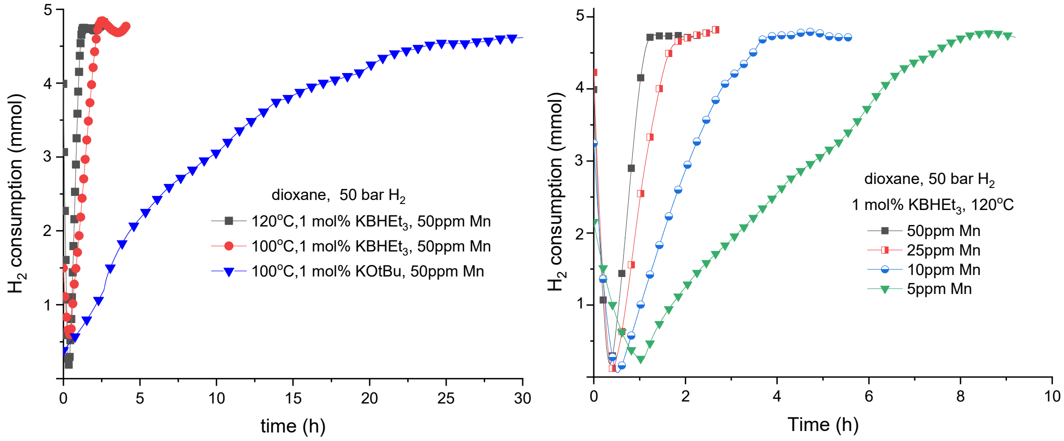

Supplementary Figure 39. H_2 consumption traces of $KBHEt_3$ promoted acetophenone hydrogenation with **3** at different temperatures (left) and catalyst loadings (right) for experiments listed in Table 2 of the manuscript.

Substrate scope

General Procedure: Inside glovebox, stock solution of **3** (0.01 M) was prepared in 0.9 mL dioxane and activated with 0.1 mL $KBHEt_3$ (1M THF solution) for 0.5 h. $KBHEt_3$ solution (25 μL), substrate (5 mmol), solvent (3 mL), dodecane (56.8 μL, 0.25 mmol), complex **3** (250, 500, 1000 μL for 50, 100, 200 ppm catalyst loading, respectively) were mixed into a 4 mL glass vials and transferred into a stainless steel autoclave. The system was purged with N_2 (3×8 bar) and H_2 (1×30 bar), pressurized with H_2 to specified pressure, and heated to specified temperature. After the reaction, resulting mixture was quenched with HCl aqueous (50 μL, 1 M) and then GC samples were prepared by dilution of the reaction mixture in THF (20 μL into 1 mL THF). Unless otherwise stated, products were identified and quantified by GC-MS. Measurements were performed on an Agilent 7890B gas chromatograph equipped with FID and MS detectors. Method details: Agilent HP-5 column (30 m, 0.25 mm ID, 0.25 μm film thickness) and temperature profile from 60 °C (hold 2 min) up to 300 °C with ramp of 20 °C/min and hold at 180 °C for 5 minutes. Mass balances were verified to be within 90 % – 110 % for all experiments. The analysis of formic acid esters hydrogenation (**8y, 8z, 8za**) were carried an Agilent 7890 gas chromatograph equipped with an FID detector. Method details: Restek-Stabilwax column (30 m, 0.32 mm ID, 0.25 μm film thickness) and temperature profile from 50 °C (hold 1.5 min) with ramp of 12 °C/min and hold at 200 °C for 1 minute. Products were identified using retention times and peak areas from analytically pure reference samples.

Supplementary Table 3. Substrate scope for carbonyl derivatives hydrogenations with 3.[a]

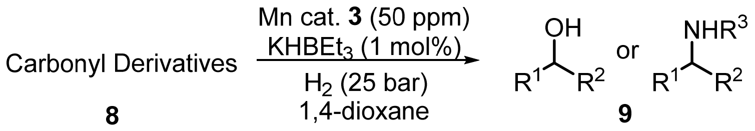

Entry	Substrate	Yield/%	TON	Uncertainty	Mass balance
1	**8a**	98	19600	529.2	0.98
2	**8b**	81	16200	437.4	1.01
3	**8c**	99	19800	534.6	0.97
4	**8d**	>99	20000	540	0.93
5	**8e**	>99	20000	540	0.97
6	**8f**	99	19800	534.6	0.95
7	**8h**	97	19400	523.8	0.98
8	**8i**	>99	20000	540	0.96
9[b]	**8m**	>99	20000	540	0.99
10[b]	**8n**	>99	20000	540	1.00
11[b]	**8o**	>99	20000	540	1.02
12[b]	**8p**	>99	20000	540	0.99
13	**8q**	trace			
14[b]	**8r**	99	19800	534.6	0.93
15[b]	**8s**	>99	20000	540	0.99
16[b]	**8t**	>99	20000	540	0.94
17[b]	**8u**	55	11000	297	0.91
18[c]	**8v**	90	9000	243	0.95
19[c]	**8x**	>99	20000	540	0.99
20[d]	**8y**	>99	20000	540	0.98
21[d]	**8z**	>99	20000	540	0.96
22[d]	**8aa**	99	20000	540	0.98

[a] Typical conditions: 5 mmol substrate, complex **3** (50 ppm), $KBHEt_3$ (1 mol%) in 1,4-dioxane (3 mL), 120 °C, P = 25 bar H_2, 24 h. Yields determined by GC-FID with dodecane internal standard. [b] Reactions carried out in isopropanol (3 mL) at 80 °C instead. [c] 100 ppm of **3** used in isopropanol (3 mL), 120 °C. [d] 200 ppm of **3** used.

Supplementary Table 4. Transfer hydrogenation activities for representative carbonyl compounds with Mn catalyst 3.

Without H_2 atmosphere, the reduction yields of substrates **8k**, **8o** and **8s** are low (9-32%), which indicates that hydrogenation pathway is dominant during the reaction in iPrOH.

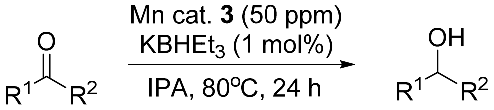

Entry	Substrate	H_2 (bar)	Yield (%)
1	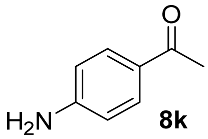 **8k**	25	99
		0	14
2	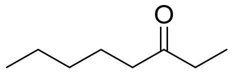 **8o**	25	99
		0	32
3	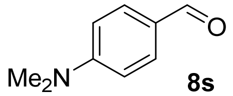 **8s**	25	99
		0	9

Hydrogenation of compounds 8g, 8j, 8k, 8l, 8w.

For compounds **9g**, **9j**, **9k, 9l**, **9w**, which could not be identified by MS, reaction mixture were quenched, evaporated, and purified by column chromatography using short silica plug. The products were identified by NMR.

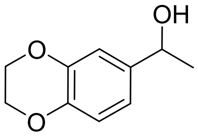

9g

According to the general procedure with 1-(2,3-dihydrobenzo[b][1,4]dioxin-6-yl)ethan-1-one (**8g**) (890.9 mg, 5 mmol). The crude product was purified by column chromatography to obtain **9g** as yellowish solid (938.1 mg, 99% yield). ^{1}H NMR (400 MHz, $CDCl_3$) δ 6.85 (s, 1H), 6.83 – 6.73 (m, 2H), 4.79 – 4.67 (m, 1H), 4.21 – 4.20 (m, 4H), 3.65 – 3.64 (m, 1H), 1.43 – 1.40 (m, 3H). ^{13}C {^{1}H}NMR (101 MHz, $CDCl_3$) δ 143.3, 142.7, 139.4, 118.5, 117.1, 114.4, 69.7, 67.0, 64.3, 25.0. The spectroscopic data correspond to those reported in literature.[4]

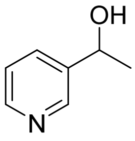

9j

According to the general procedure with 1-(pyridin-3-yl)ethan-1-one (**8i**) (122.2 mg, 1.25 mmol). The crude product was purified by column chromatography to obtain **9i** as yellowish liquid (124.2 mg, 99% yield). ^{1}H NMR (400 MHz, $CDCl_3$) δ 8.26 (s, 1H), 8.15 (d, J = 4.9 Hz, 1H), 7.58 (d, J = 7.9 Hz, 1H), 7.09-7.06 (s, 1H), 5.10 (s, 1H), 4.73

(q, J = 6.5 Hz, 1H), 1.32 (d, J = 6.6 Hz, 1H). ^{13}C {^{1}H}NMR (101 MHz, $CDCl_3$) δ 147.6, 146.8, 141.9, 133.6, 123.5, 67.1, 67.1, 25.1, 25.1. The spectroscopic data correspond to those reported in literature.[5]

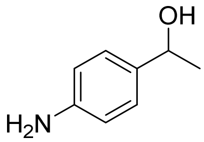

9k

According to the general procedure with 1-(4-aminophenyl)ethan-1-one (**8k**) (337.5 mg, 2.5 mmol). The crude product was purified by column chromatography to obtain **9k** as yellowish liquid (340.8 mg, 99% yield). ^{1}H NMR (400 MHz, $CDCl_3$) δ 7.13 (d, *J* = 8.4 Hz, 2H), 6.62 (d, *J* = 8.4 Hz, 2H), 4.75 (qd, *J* = 6.5, 3.4 Hz, 1H), 3.64 (s, 2H), 2.23 (d, *J* = 3.4 Hz, 1H), 1.43 (d, *J* = 6.5 Hz, 3H). ^{13}C {^{1}H}NMR (101 MHz, $CDCl_3$) δ 145.7, 136.0, 126.6, 115.1, 70.0, 24.8. The spectroscopic data correspond to those reported in literature.[6]

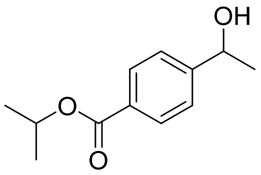

9l

According to the general procedure with methyl 4-acetylbenzoate (**8l**) (222.7 mg, 1.25 mmol). The crude product was purified by column chromatography to obtain **9i** as yellowish liquid (295.1 mg, 99% yield). ^{1}H NMR (400 MHz, $CDCl_3$) δ 7.85 (d, J = 7.9 Hz, 2H), 7.30 (d, J = 8.0 Hz, 2H), 5.10 (sep, J = 6.0 Hz, 1H), 4.79 (q, J = 6.4 Hz, 1H), 3.78 (s, 1H), 1.36 (d, J = 6.4 Hz, 3H), 1.26 (d, J = 6.5 Hz, 6H). ^{13}C {^{1}H}NMR (101 MHz, $CDCl_3$) δ 166.1, 151.3, 129.5, 125.2, 69.5, 68.3, 25.2, 21.8. The spectroscopic data correspond to those reported in literature.[7]

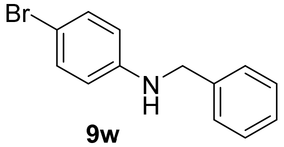

9w

According to the general procedure with methyl (E)-N-(4-bromophenyl)-1-phenylmethanimine (**8w**) (647.5 mg, 2.5 mmol). The crude product was purified by column chromatography to obtain **9i** as yellowish liquid (671.8 mg, 99% yield). ^{1}H NMR (400 MHz, $CDCl_3$) δ 7.43 – 7.29 (m, 5H), 7.28 (d, *J* = 8.8 Hz, 2H),6.55 (d, J = 8.8 Hz, 2H), 4.32 (d, *J* = 5.7 Hz, 2H), 4.10 (d, *J* = 5.7 Hz, 1H). ^{13}C {^{1}H}NMR (101 MHz, $CDCl_3$) δ 147.2, 139.0, 132.0, 128.8, 127.5, 127.5, 114.6, 109.2, 48.3.[8]

NMR Spectra of Products 9g, 9j, 9k, 9l, 9w

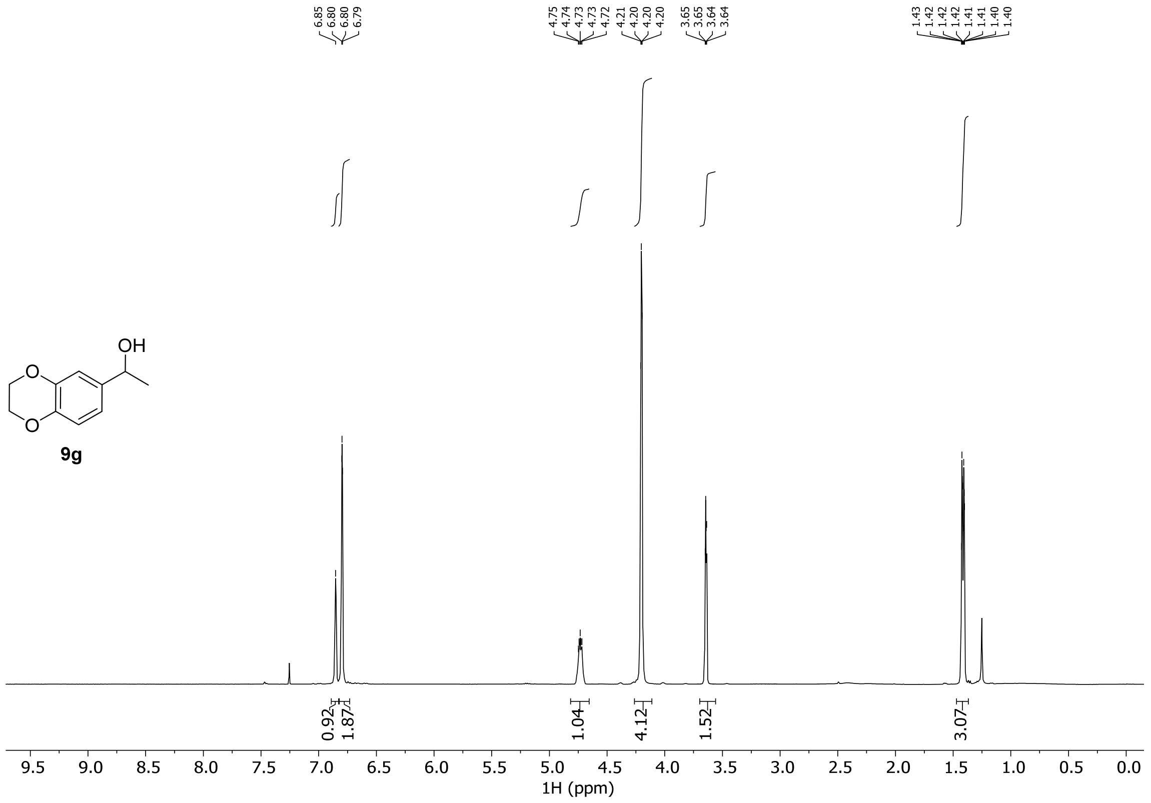

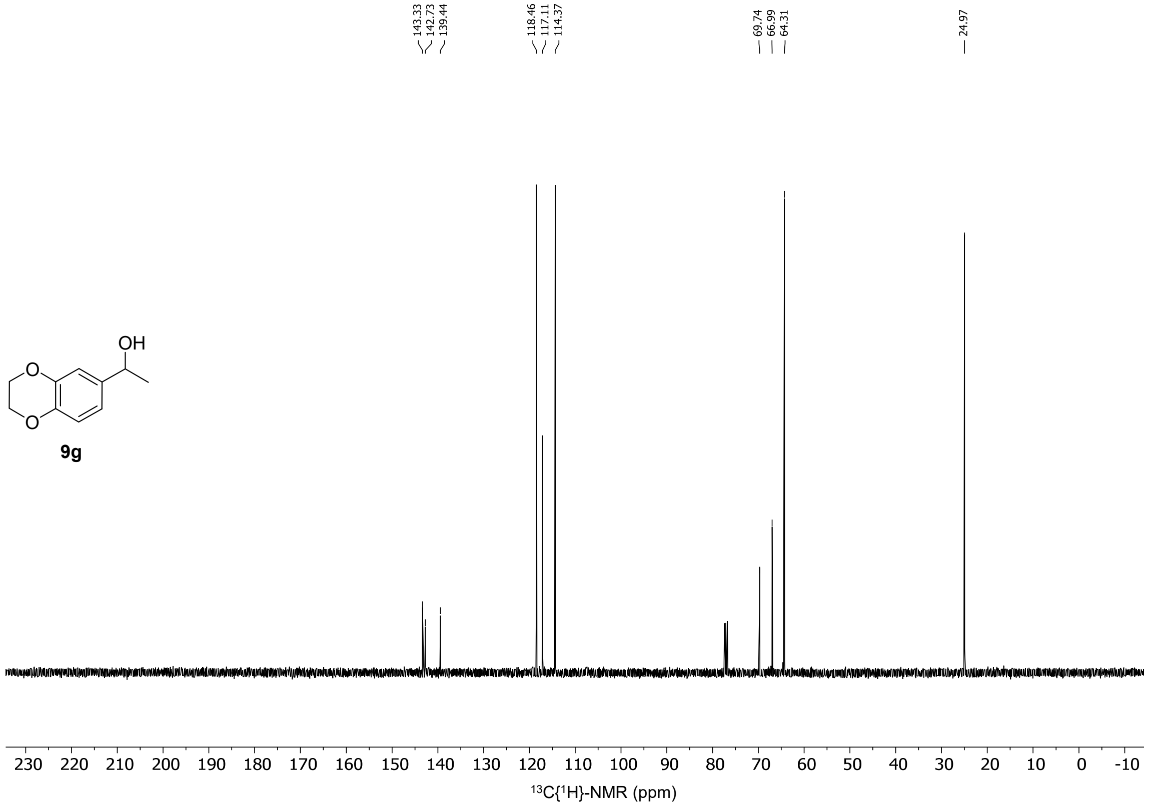

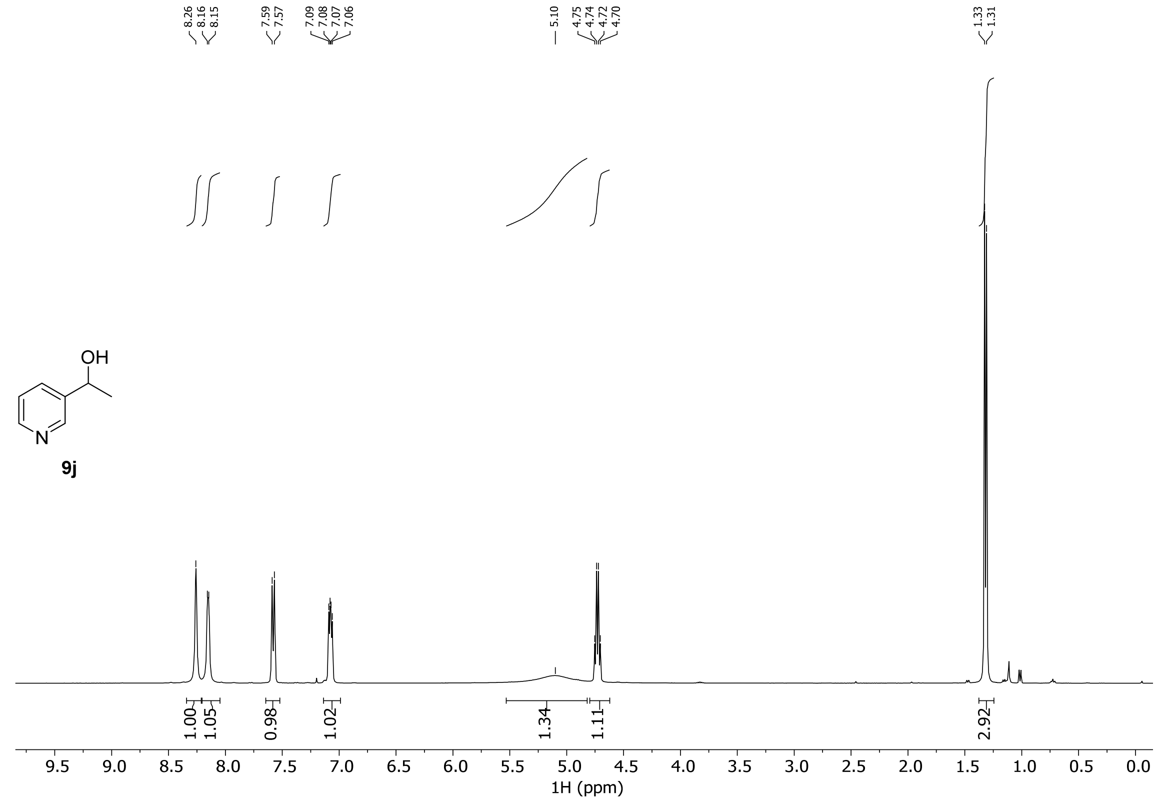

8.26
8.16
8.15
7.59
7.57
7.09
7.08
7.07
7.06
5.10
4.75
4.74
4.72
4.70
1.33
1.31
OH
N
9j
1.00
1.05
0.98
1.02
1.34
1.11
2.92
9.5 9.0 8.5 8.0 7.5 7.0 6.5 6.0 5.5 5.0 4.5 4.0 3.5 3.0 2.5 2.0 1.5 1.0 0.5 0.0
1H (ppm)

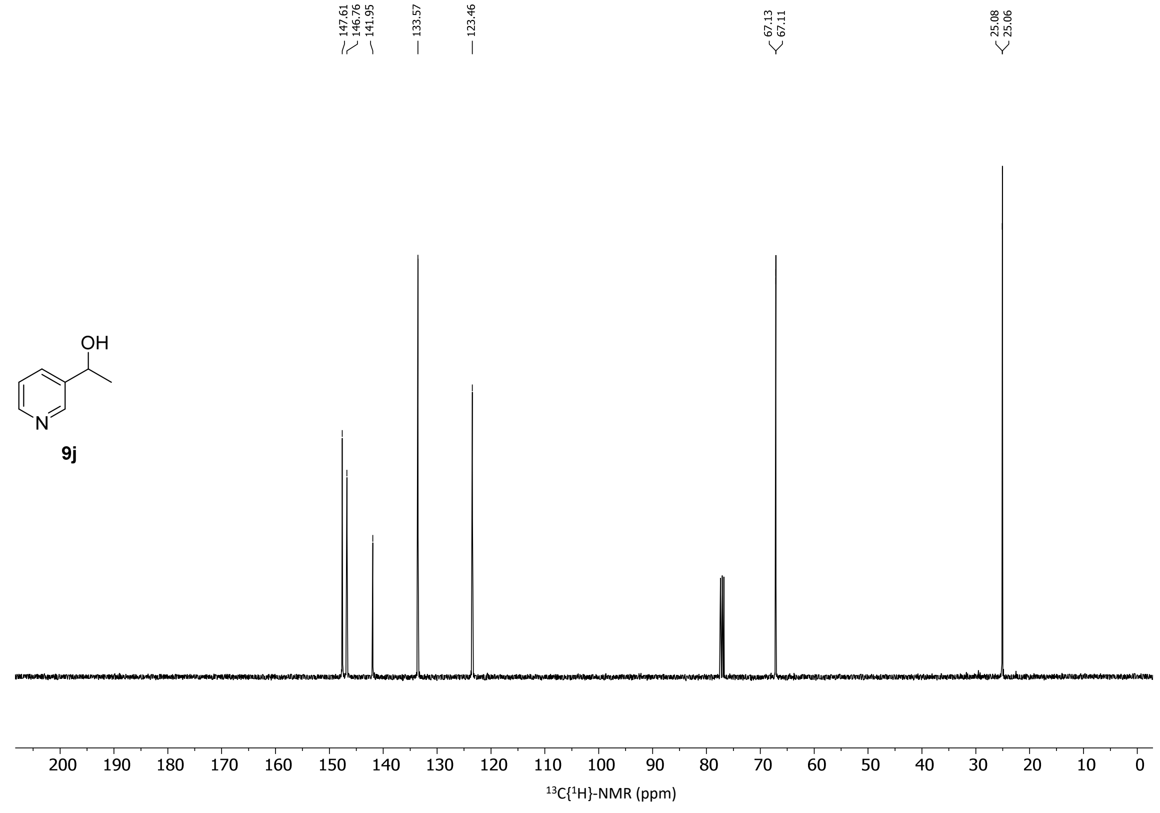

147.61
146.76
141.95
133.57
123.46
67.13
67.11
25.08
25.06
OH
N
9j
200 190 180 170 160 150 140 130 120 110 100 90 80 70 60 50 40 30 20 10 0
13C{1H}-NMR (ppm)

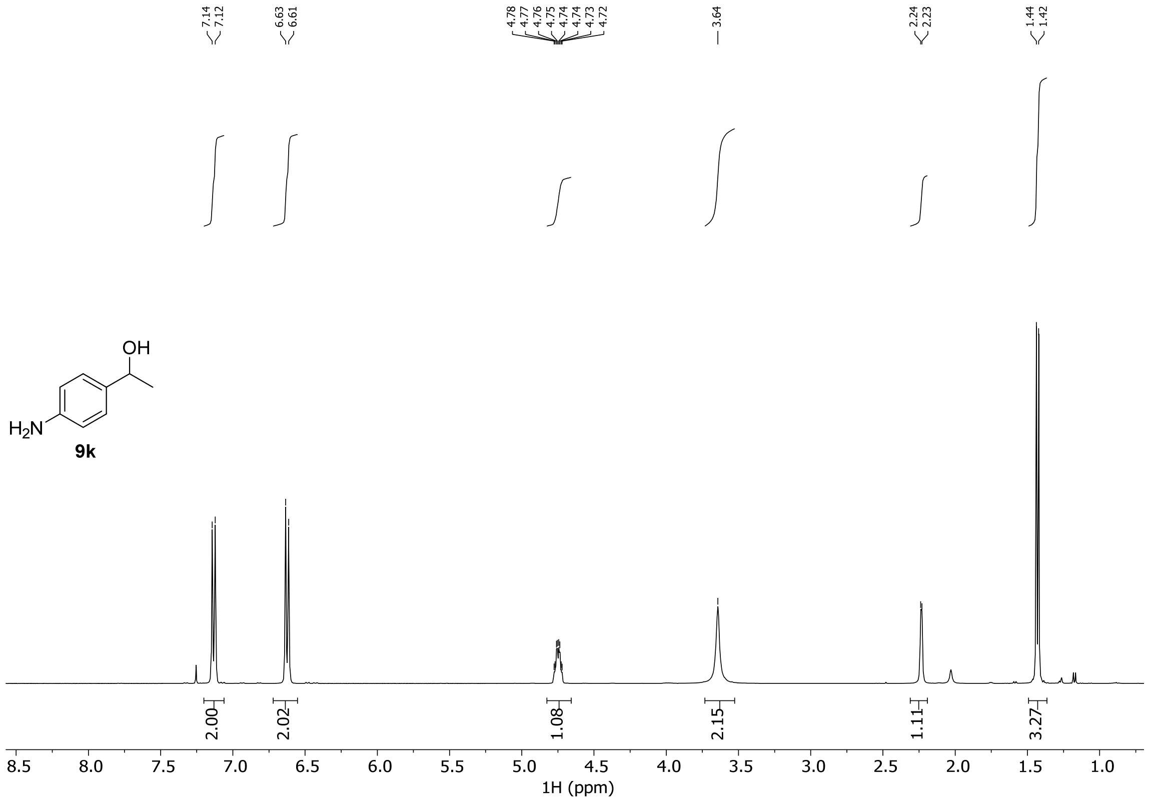
7.14
7.12
6.63
6.61
4.78
4.77
4.76
4.75
4.74
4.74
4.73
4.72
3.64
2.24
2.23
1.44
1.42
OH
H2N
9k
2.00
2.02
1.08
2.15
1.11
3.27
8.5
8.0
7.5
7.0
6.5
6.0
5.5
5.0
4.5
4.0
3.5
3.0
2.5
2.0
1.5
1.0
1H (ppm)

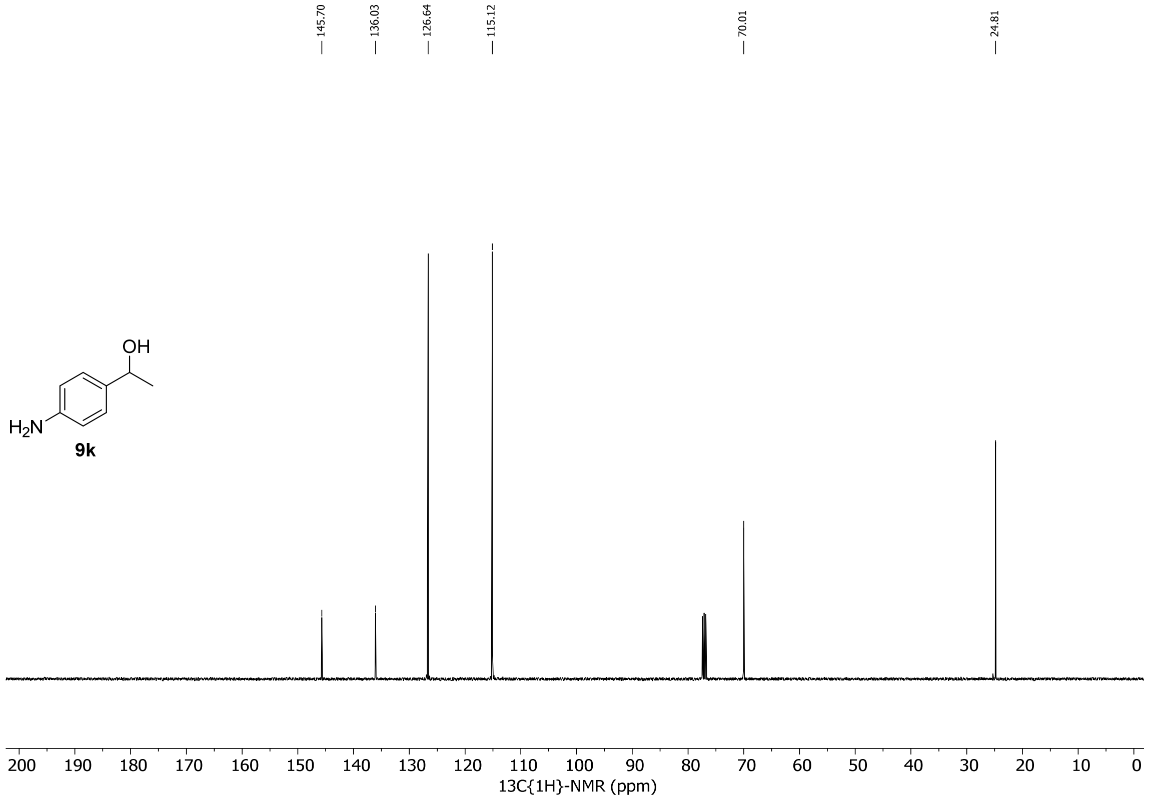
145.70
136.03
126.64
115.12
70.01
24.81
OH
H2N
9k
200
190
180
170
160
150
140
130
120
110
100
90
80
70
60
50
40
30
20
10
0
13C{1H}-NMR (ppm)

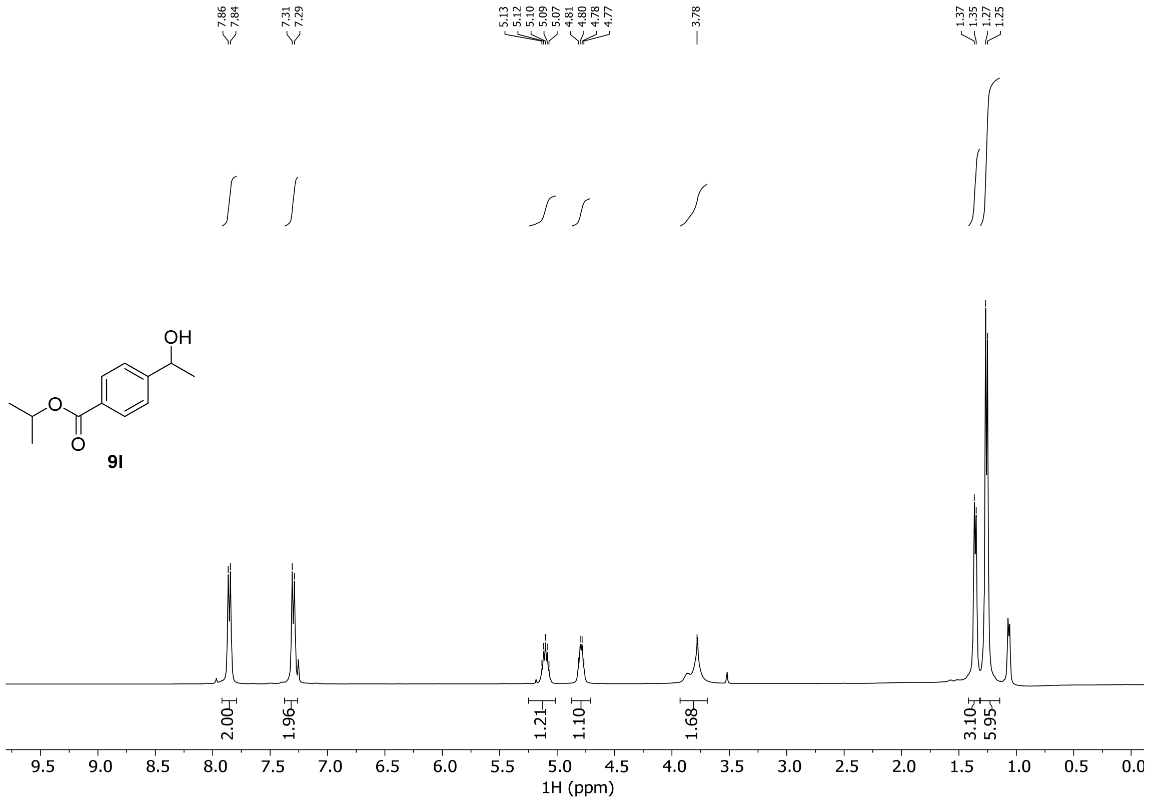
7.86
7.84
7.31
7.29
5.13
5.12
5.10
5.09
5.07
4.81
4.80
4.78
4.77
3.78
1.37
1.35
1.27
1.25
OH
O
O
9l
2.00
1.96
1.21
1.10
1.68
3.10
5.95
1H (ppm)

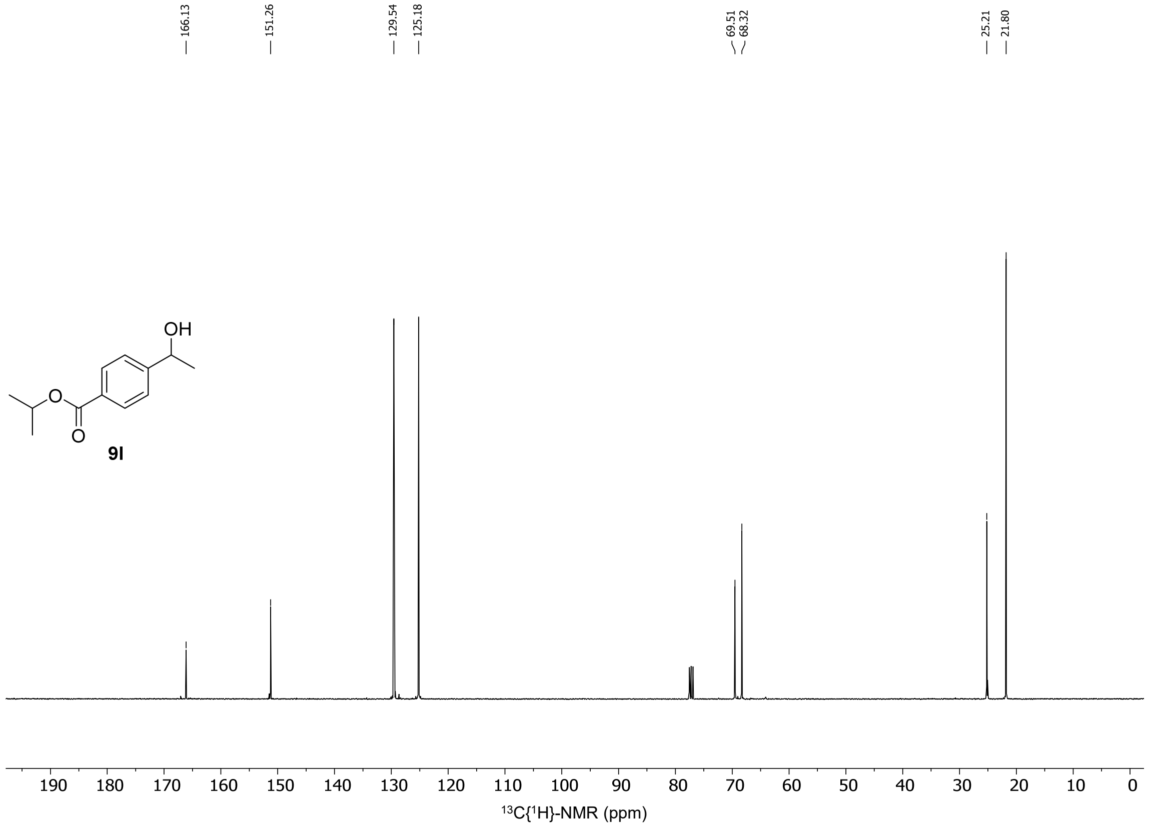
166.13
151.26
129.54
125.18
69.51
68.32
25.21
21.80
OH
O
O
9l
13C{1H}-NMR (ppm)

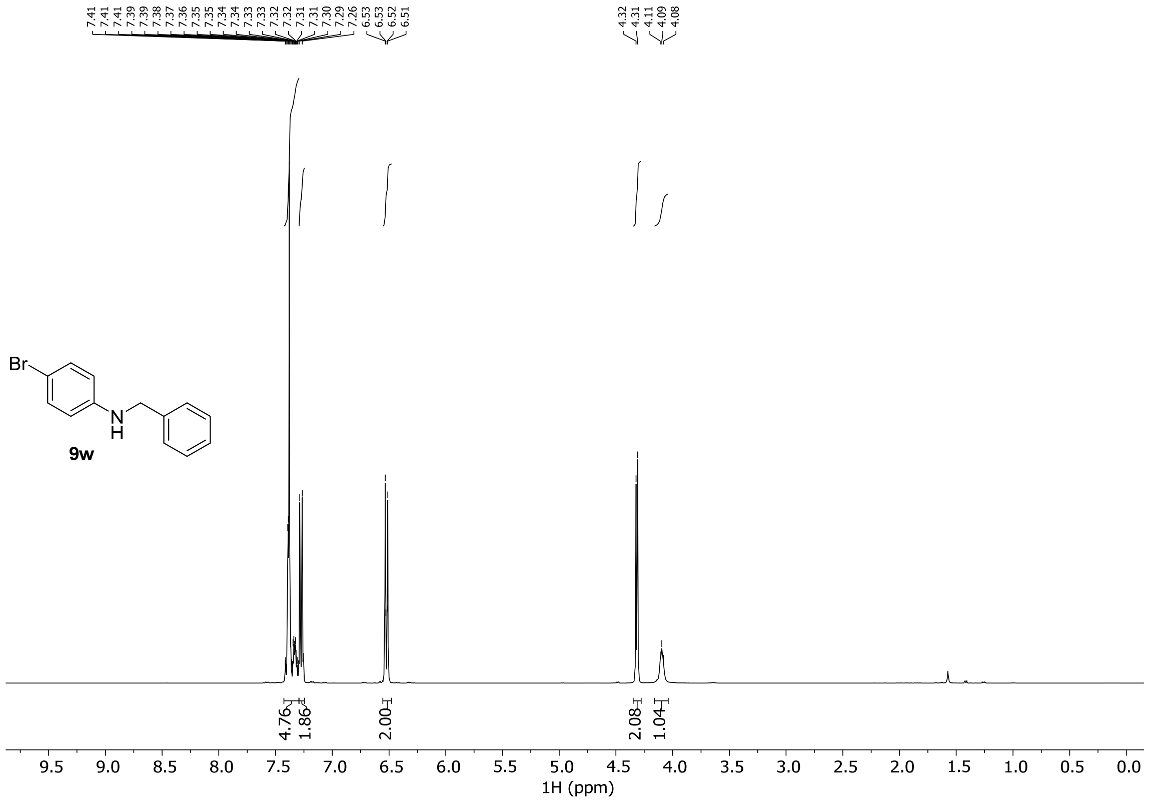
7.41
7.41
7.41
7.39
7.39
7.38
7.37
7.36
7.35
7.35
7.34
7.34
7.33
7.33
7.32
7.32
7.31
7.31
7.30
7.29
7.26
6.53
6.53
6.52
6.51
4.32
4.31
4.11
4.09
4.08
Br
N
H
9w
4.76
1.86
2.00
2.08
1.04
9.5 9.0 8.5 8.0 7.5 7.0 6.5 6.0 5.5 5.0 4.5 4.0 3.5 3.0 2.5 2.0 1.5 1.0 0.5 0.0
1H (ppm)

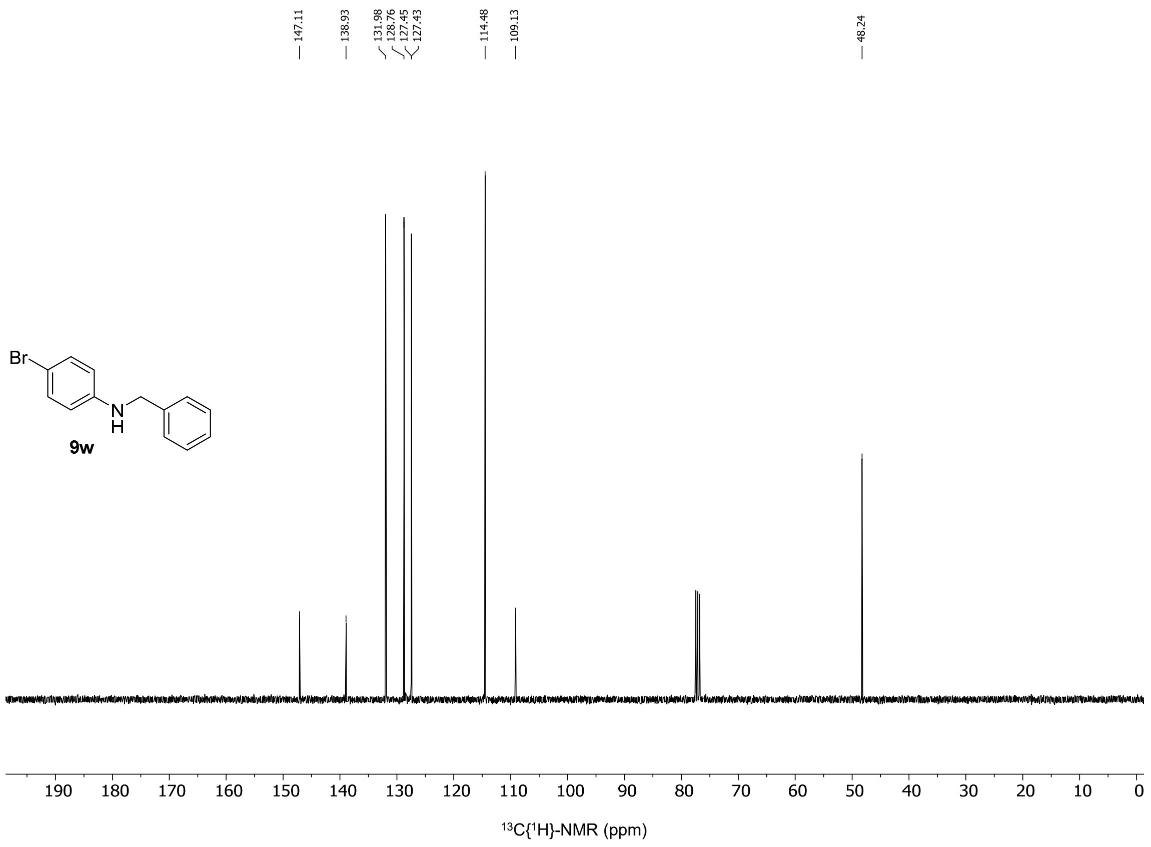
147.11
138.93
131.98
128.76
127.45
127.43
114.48
109.13
48.24
Br
N
H
9w
190 180 170 160 150 140 130 120 110 100 90 80 70 60 50 40 30 20 10 0
13C{1H}-NMR (ppm)

Computational Studies.

All quantum chemical calculations were performed using Gaussian16 software.[9] Geometries and IR spectra were calculated at PBE0-D3/6-311+G(d,p) level of theory with a continuous description of the THF solvent using the SMD model. ^{1}H NMR shielding was computed for the optimized geometries at PBE0/6-311++G(2d,2p) level of theory, which shows good performance for the calculation of shielding constants;[10,11] the solvent (d_8-THF) was taken into account with the PCM model, which works well for modeling NMR spectra in solutions. [12,13]

The calculated IR frequencies were scaled with the factor f = 0.9576, which was chosen for best agreement of experimental and calculated data. It is applied to minimize the error of the harmonic approach for the vibration frequency calculation[14]. The Half width of the plotted calculated spectra is 5 cm^{-1}.

While definitive assignment of specific solution structures to isomers of 5 and 6 is challenging we could rule out the formation of *fac* isomers and narrow the scope of potential isomers of **5**/**6** to positional isomers with inverted axial arrangement of CO and hydride ligands.

Analysis of *fac* isomers.

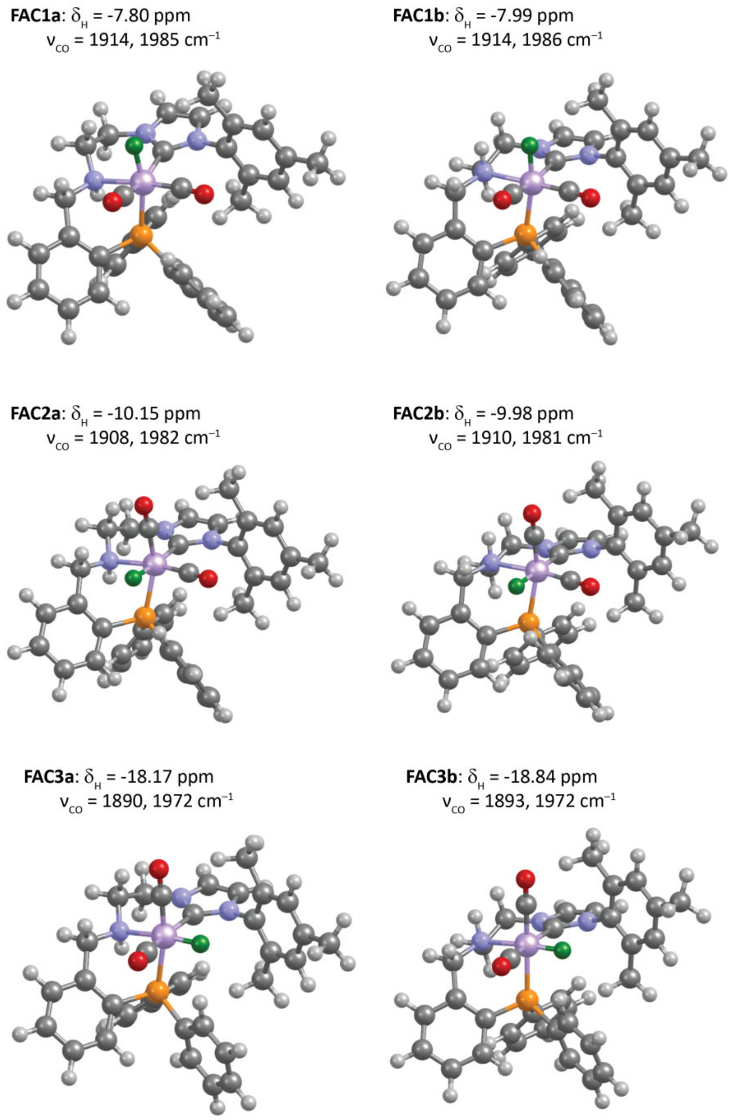

Supplementary Figure 40. DFT-calculated structures, CO ligand vibration frequencies and hydride resonances for *fac* isomers of **5**. Hydride ligand shown in green.

For all calculated *fac* isomers of **5** we observed significantly high field shifted hydride resonance values varying from -7.8 to -18.8 ppm that allowed us to rule out the formation of hydride species with *fac*-bound CNP ligand.

Another characteristic feature of *fac* isomers is the placement of the hydride ligand *trans* to donor groups of the CNP ligand that results in significant high field shifts that were not experimentally observed.

Analysis of *mer* isomers.

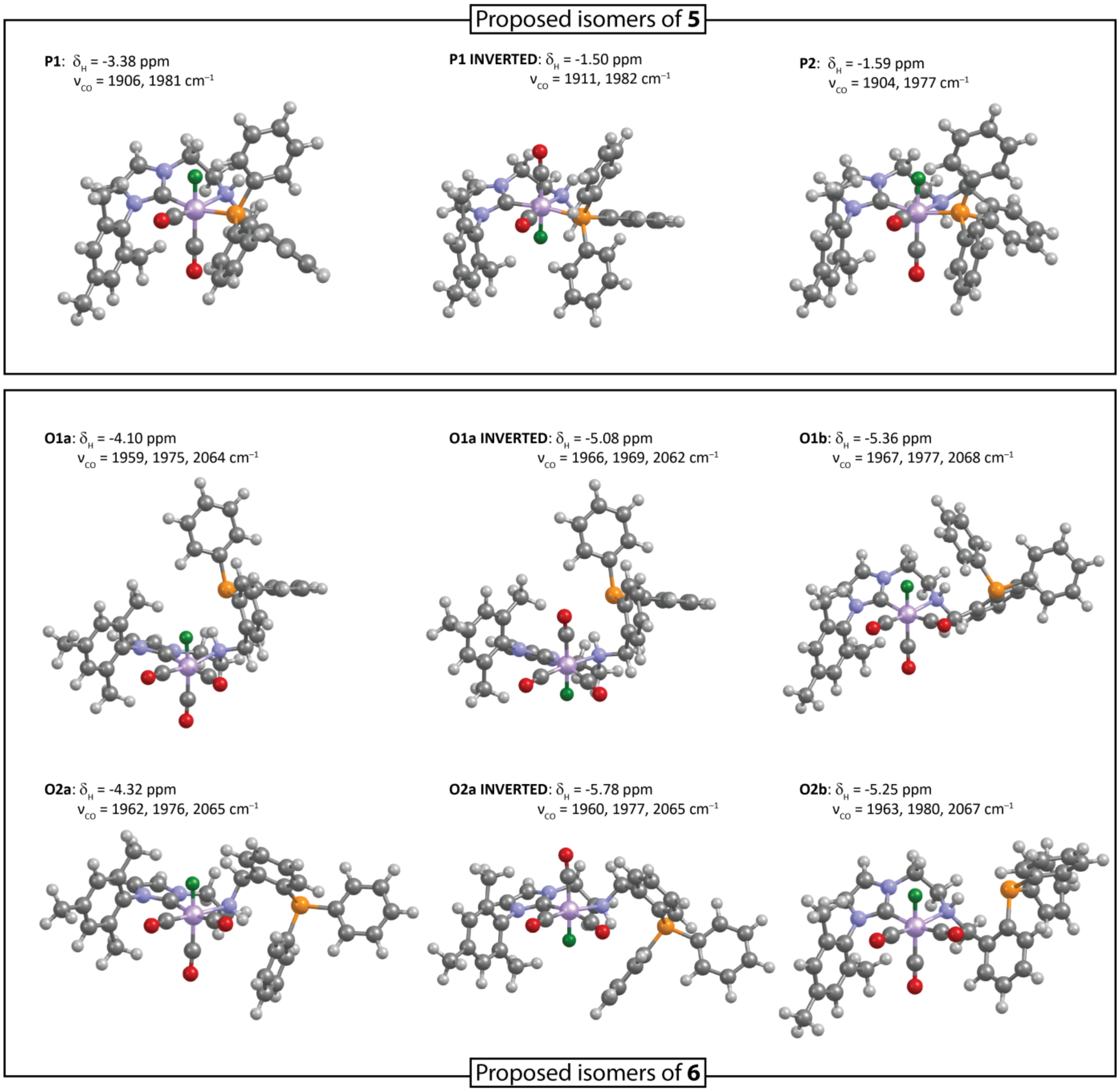

Supplementary Figure 41. DFT-calculated structures, CO ligand vibration frequencies and hydride resonances for *mer* isomers of **5** and **6**. Hydride ligand shown in green. "O" stands for open, "P" for pincer.

All analyzed isomers feature similar CO ligand vibration frequencies and differ marginally in calculated hydride donor resonance values within ca 1 ppm.

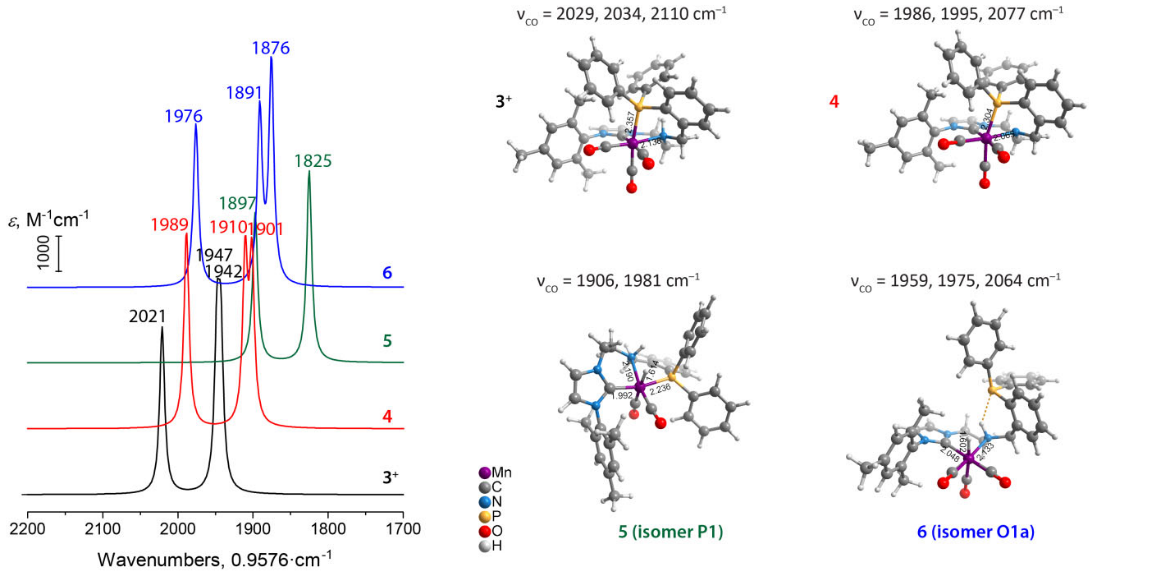

Supplementary Figure 42. DFT-computed scaled IR spectra of complex **3**, **4**, and selected isomers of **5** and **6**. See Supplementary Figure 41 for alternative structures.

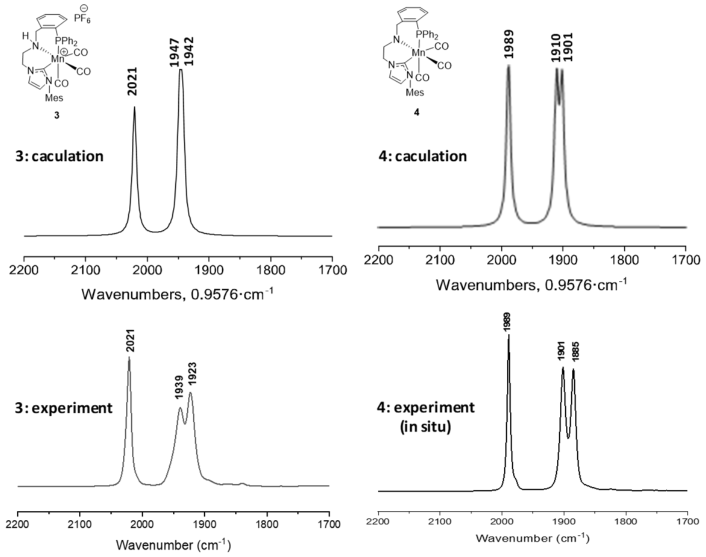

Supplementary Figure 43. Experimental IR spectra of **3** (bottom left), **4** (bottom right) and DFT-calculated IR spectra of **3** (top left) and **4** (top right) in THF. The results matched well, providing a reference for the set of scaling factor.

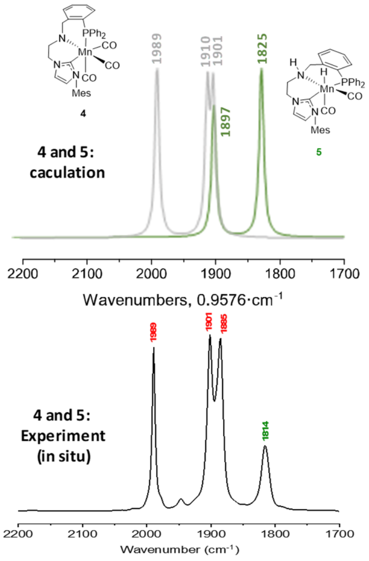

Supplementary Figure 44. Experimental IR spectra (bottom) of in situ mixture of **4** and **5**, DFT-calculated IR spectra (top) of the mixture of **4** (grey) and **5** (green) in THF. The experimental band of 1814 cm^{-1} should be Mn-H complex **5**, while the other band overlapped with complex **4**.

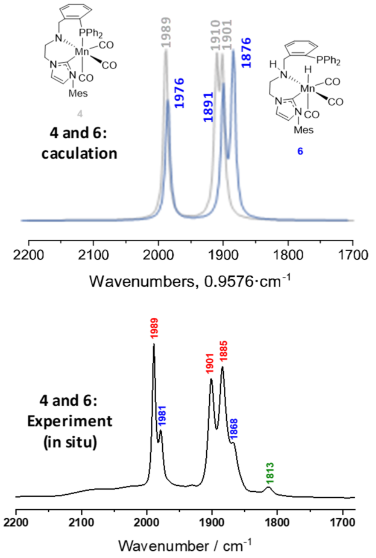

Supplementary Figure 45. Experimental IR spectra (bottom) of in situ mixture of **4** and **6**, DFT-calculated IR spectra (top) of the mixture of **4** (grey) and **6** (blue) in THF. The experimental band of 1981, 1814 cm^{-1} should be Mn-H complex **6**, while the last band overlapped with complex **4**.

Crystal Structure Analysis Details.

X-ray crystal structure determination of Mn complex 4: Crystals suitable for X-ray diffraction were obtained by slow pentane vapour diffusion into THF solution of **4**. Crystallographic data: $C_{36}H_{33}MnN_3O_3P$, *Fw* =641.56 g mol^{-1}, yellow block, 0.28x0.21x0.11 mm^3 (crystal size), triclinic, *P-1*, a = 9.2569(2) Å, b = 11.6213(2) Å, c = 16.3438(2) Å, α = 92.1528(7) °, β = 93.1681(7) °, γ = 93.8695(7) °, V = 1750.02(6) Å^3, Z = 2, Dx = 1.217 g cm^{-3}, μ = 0.460 mm^{-1}. 24537 reflections were measured by a Bruker D8-Venture Photon area detector (MoKα radiation, λ = 0.71073 Å^{-1}) up to a resolution of (sin(Θ)/λ)max = 0.63 Å^{-1} at a temperature of 100 K. Reflections were corrected for adsorption and scaled on the basis of multiple measured reflections using the SADABS program (0.879 – 0.928 correction range).[15] 7180 Reflections were unique (Rint = 0.0333). Using ShelXle[16], the structures were solved with SHELXS-14[16] by using direct methods and refined with SHELXL-2018[17] on F2 for all reflections. Non-hydrogen atoms were refined by using anisotropic displacement parameters. Positions of hydrogen atoms were calculated for idealized positions. 0 Parameters were refined without restraints. R1 = 0.0314 for 6167 reflections with $I > 2\sigma(I)$ and wR2 = 0.0795 for 7180 reflections. S = 1.038. Residual electron density was between 0.36 and -0.36 eÅ-3. Geometry calculations and checks for higher symmetry were performed with the PLATON program.[18] The PLATON/SQUEEZE[19] was used as a tool to calculate the disordered solvent contribution to the calculated structural factors because the solvent could not be described correctly.

CCDC-1994375 contains the supplementary crystallographic data for this paper. These data can be obtained free of charge from The Cambridge Crystallographic Data Centre *via* www.ccdc.cam.ac.uk/data_request/cif.

Supplementary References.

1. Occhipinti, G.; Bjørsvik, H.-R.; Törnroos, K. W.; Fürstner, A.; Jensen, V. R., The first imidazolium-substituted metal alkylidene. *Organometallics* **2007,** *26*, 4383-4385.
2. Jarvis, A. G.; Sehnal, P. E.; Bajwa, S. E.; Whitwood, A. C.; Zhang, X.; Cheung, M. S.; Lin, Z.; Fairlamb, I. J., A Remarkable cis-and trans-Spanning Dibenzylidene Acetone Diphosphine Chelating Ligand (dbaphos). *Chem. –Eur. J.* **2013,** *19*, 6034-6043.
3. Technische Universiteit Delft, Sampling arrangement, Patent 2024908, February 14, 2020.
4. Boit, T. B.; Mehta, M. M.; Garg, N. K., Base-Mediated Meerwein–Ponndorf–Verley Reduction of Aromatic and Heterocyclic Ketones. *Org. Lett.* **2019,** *21*, 6447-6451.
5. Yu, J.; Long, J.; Yang, Y.; Wu, W.; Xue, P.; Chung, L. W.; Dong, X.-Q.; Zhang, X., Iridium-catalyzed asymmetric hydrogenation of ketones with accessible and modular ferrocene-based amino-phosphine acid (f-ampha) ligands. *Org. Lett.* **2017,** *19*, 690-693.
6. Motoyama, Y.; Kamo, K.; Nagashima, H., Catalysis in polysiloxane gels: platinum-catalyzed hydrosilylation of polymethylhydrosiloxane leading to reusable catalysts for reduction of nitroarenes. *Org. Lett.* **2009,** *11*, 1345-1348.
7. Hsu, S. F.; Plietker, B., Selective Transfer Hydrogenation and Hydrogenation of Ketones Using a Defined Monofunctional (P^ N (Bn)^ N (Bn)^ P)–RuII Complex. *Chem. –Eur. J.* **2014,** *20*, 4242-4245.
8. Freitag, F.; Irrgang, T.; Kempe, R., Mechanistic Studies of Hydride Transfer to Imines from a Highly Active and Chemoselective Manganate Catalyst. *J. Am. Chem. Soc.* **2019,** *141*, 11677-11685.
9. Gaussian 16, Revision B.01, M. J. Frisch, G. W. Trucks, H. B. Schlegel, G. E. Scuseria, M. A. Robb, J. R. Cheeseman, G. Scalmani, V. Barone, G. A. Petersson, H. Nakatsuji, X. Li, M. Caricato, A. V. Marenich, J. Bloino, B. G. Janesko, R. Gomperts, B. Mennucci, H. P. Hratchian, J. V. Ortiz, A. F. Izmaylov, J. L. Sonnenberg, D. Williams-Young, F. Ding, F. Lipparini, F. Egidi, J. Goings, B. Peng, A. Petrone, T. Henderson, D. Ranasinghe, V. G. Zakrzewski, J. Gao, N. Rega, G. Zheng, W. Liang, M. Hada, M. Ehara, K. Toyota, R. Fukuda, J. Hasegawa, M. Ishida, T. Nakajima, Y. Honda, O. Kitao, H. Nakai, T. Vreven, K. Throssell, J. A. Montgomery, Jr., J. E. Peralta, F. Ogliaro, M. J. Bearpark, J. J. Heyd, E. N. Brothers, K. N. Kudin, V. N. Staroverov, T. A. Keith, R. Kobayashi, J. Normand, K. Raghavachari, A. P. Rendell, J. C. Burant, S. S. Iyengar, J. Tomasi, M. Cossi, J. M. Millam, M. Klene, C. Adamo, R. Cammi, J. W. Ochterski, R. L. Martin, K. Morokuma, O. Farkas, J. B. Foresman, and D. J. Fox, Gaussian, Inc., Wallingford CT, 2016.
10. I. Yu. Chernyshov, M. V. Vener, I. G. Shenderovich, Local-structure effects on ^{31}P NMR chemical shift tensors in solid state. *J. Chem. Phys.* **2019**, *150*, 144706.
11. B. Maryasin, H. Zipse, Theoretical studies of ^{31}P NMR spectral properties of phosphanes and related compounds in solution. *Phys. Chem. Chem. Phys.* **2011**, *13*, 5150–5158.
12. M. Dračínský, P. Bouř, Computational Analysis of Solvent Effects in NMR Spectroscopy, *J. Chem. Theory Comput.* **2010**, *6*, 288–299.
13. V. A. Semenov, D. O. Samultsev, L. B. Krivdin, Solvent effects in the GIAO-DFT calculations of the ^{15}N NMR chemical shifts of azoles and azines. *Magn. Reson. Chem.* **2014**, *52*, 686–693.
14. A. L. Spek, Structure validation in chemical crystallography. *Acta Cryst.* **2009**, *D65*, 148–155.
15. Bruker **(2013)**. *APEX2*, *SAINT, XPREP* and *SADABS*. Bruker AXS Inc., Madison, Wisconsin USA.
16. B. Hübschle, G. M. Sheldrick, D Dittrich, *J. Appl. Cryst.* **2011**, *44*, 1281 – 1284.
17. G. M. Sheldrick, *Acta Cryst.* **2015**, *C71*, 3-8.
18. *PLATON*. A. L. Spek, *Acta Cryst.* **2009**, *D65*, 148–155.
19. *PLATON/SQUEEZE*. Spek, A. L., *Acta Cryst.* **2015**, *C71*, 9-18.
