# Supplementary material for: Robust and efficient hydrogenation of carbonyl compounds catalysed by mixed donor Mn(I) pincer complexes
Source: Nat Commun. 2021 Jan 4;12:12. doi: 10.1038/s41467-020-20168-2 (PMC7782525; doi:10.1038/s41467-020-20168-2)
Supplement: Supplementary file 3 — Source Data [file 41467_2020_20168_MOESM3_ESM.zip › CNP dataset/GC traces.pdf]

MS-Table 1, entry 1

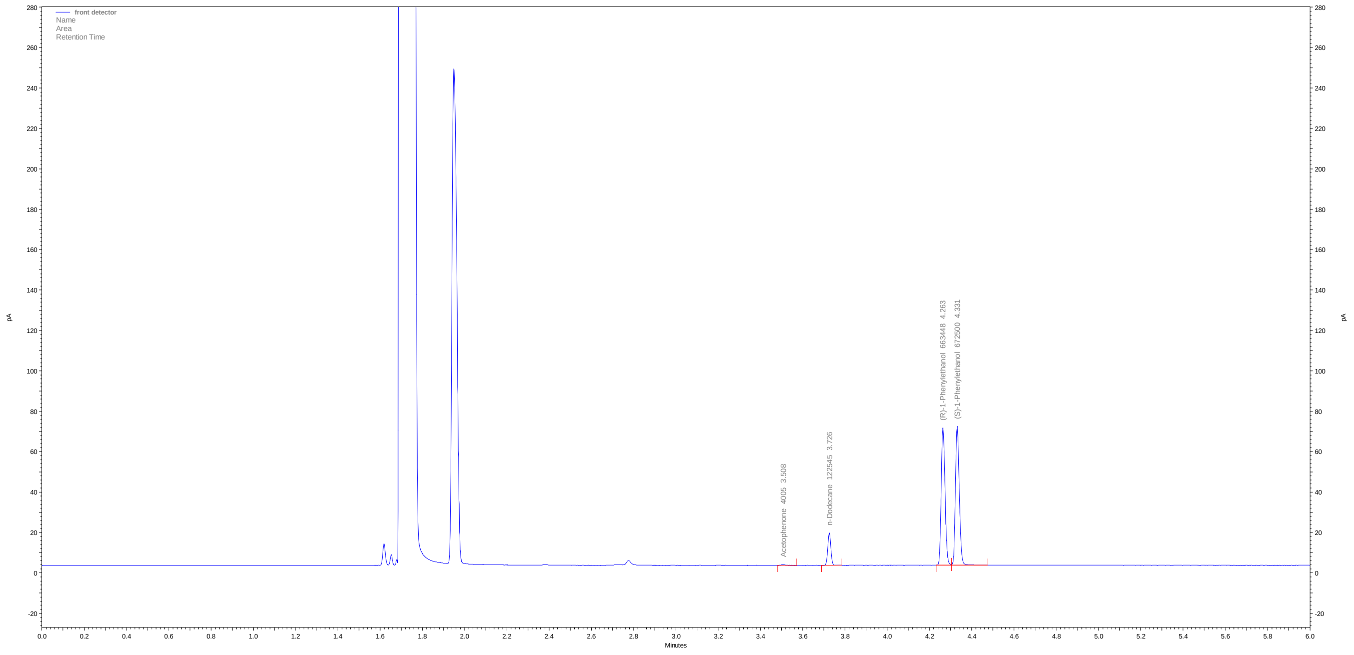

D:\TUD204708\6890 GC Data\Wenjun\WJ181-1.rslt\WJ181-1.dat, front detector

MS-Table 1, entry 2

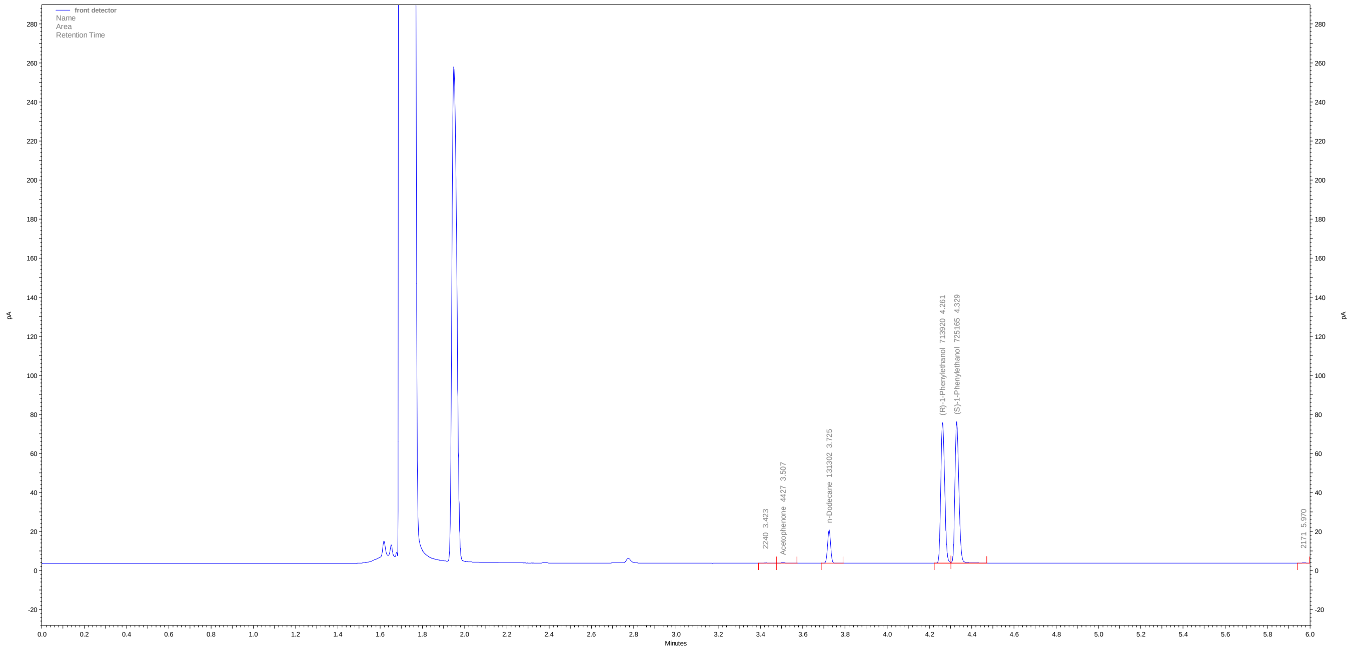

D:\TUD204708\6890 GC Data\Wenjun\WJ181-3.rslt\WJ181-3.dat, front detector

MS-Table 1, entry 3

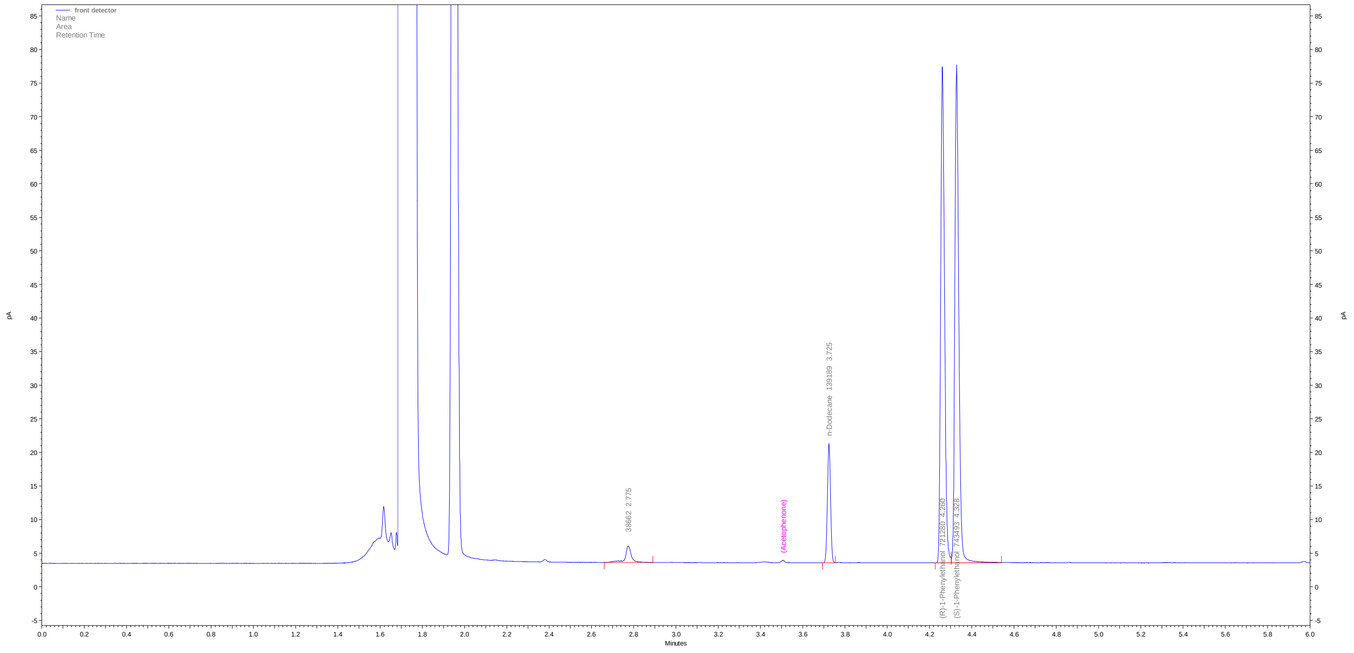

D:\TUD204708\6890 GC Data\Wenjun\WJ181-4.rslt\WJ181-4 - Copy.dat, front detector

MS-Table 1, entry 4

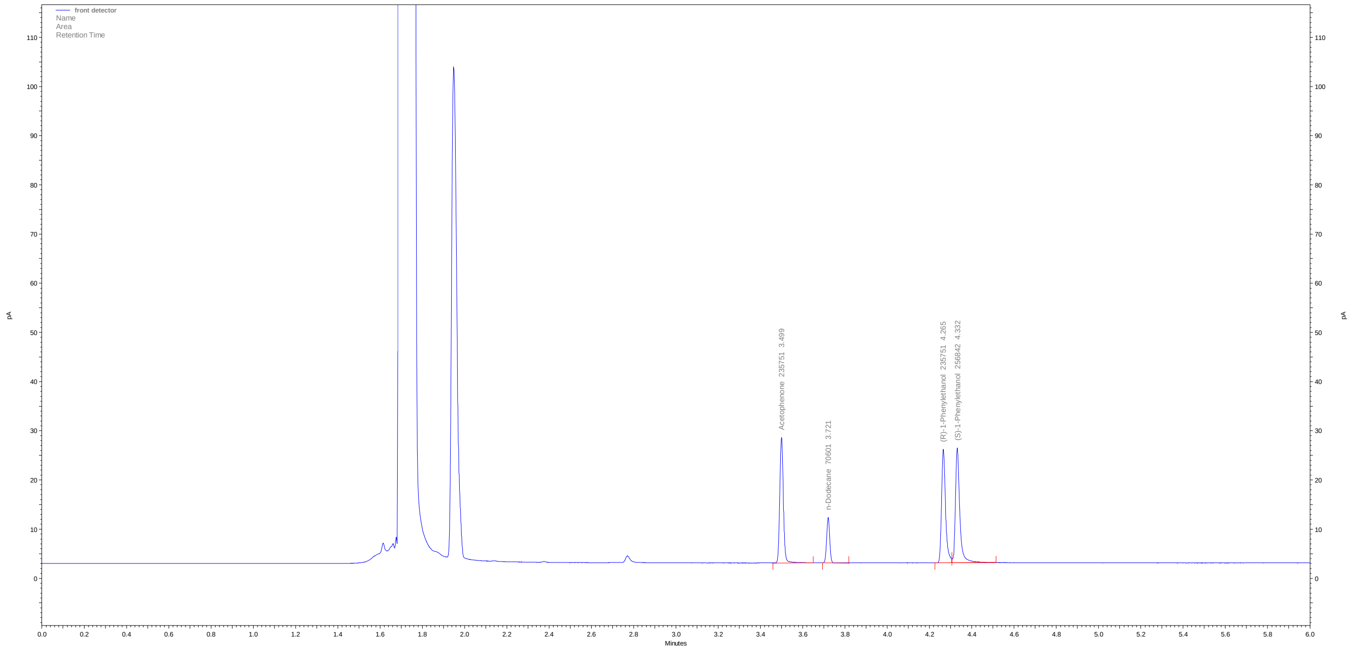

D:\TUD204708\6890 GC Data\Wenjun\WJ-206-51-PNP.rslt\WJ-206-51-PNP.dat, front detector

MS-Table 1, entry 5

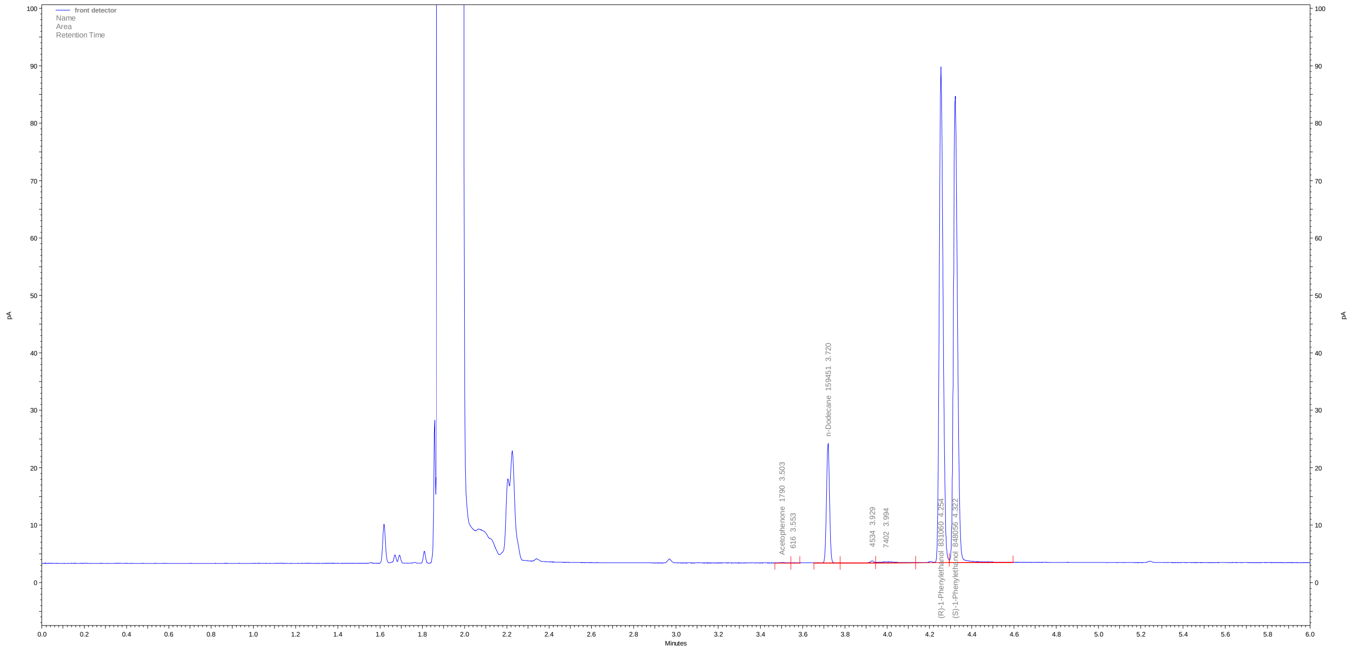

D:\TUD204708\6890 GC Data\Wenjun\WJ193-1-6-191-7-12.rslt\WJ191-7, front detector

MS-Table 1, entry 6

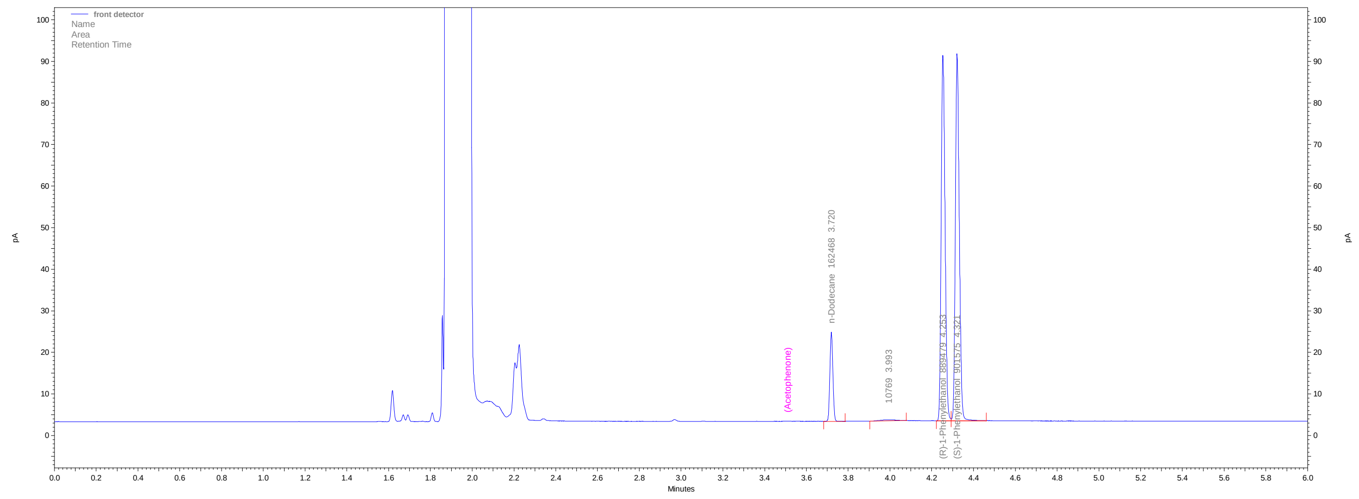

D:\TUD204708\6890 GC Data\Wenjun\WJ-193-10.rslt\WJ-193-10.dat, front detector

MS-Table 1, entry 7

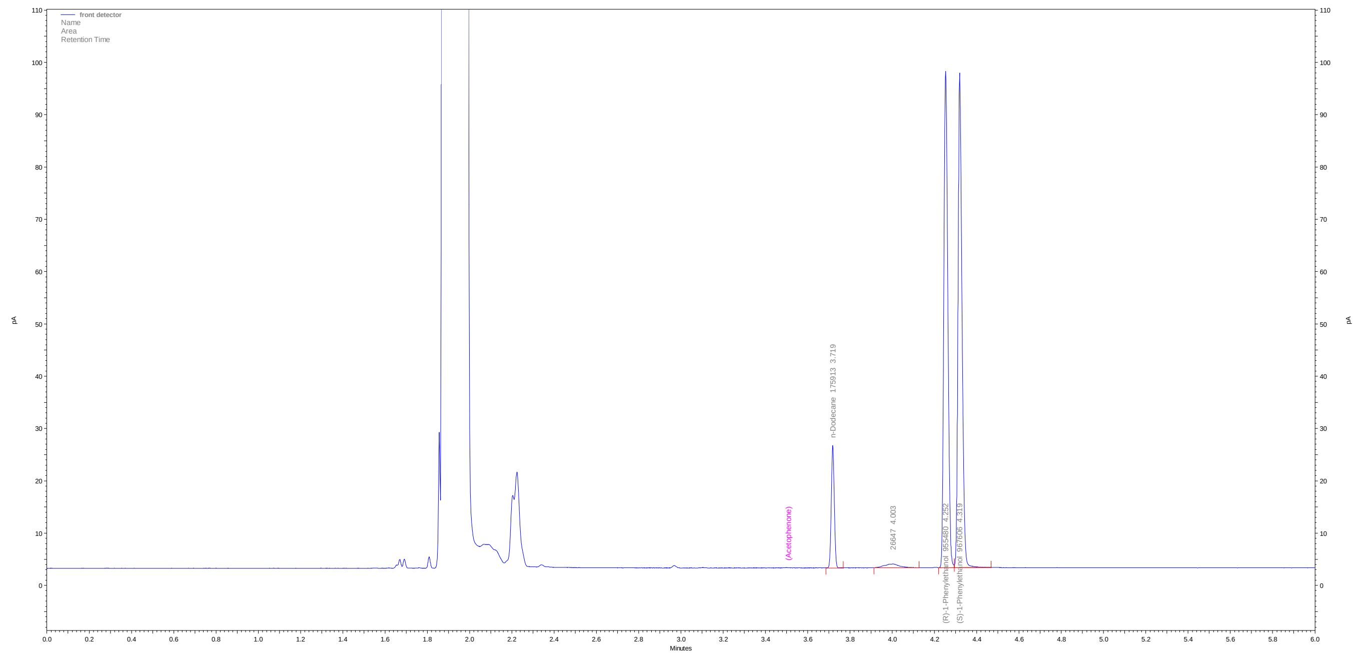

D:\TUD204708\6890 GC Data\Wenjun\WJ197-40.rslt\WJ197-40.dat, front detector

MS-Table 1, entry 8

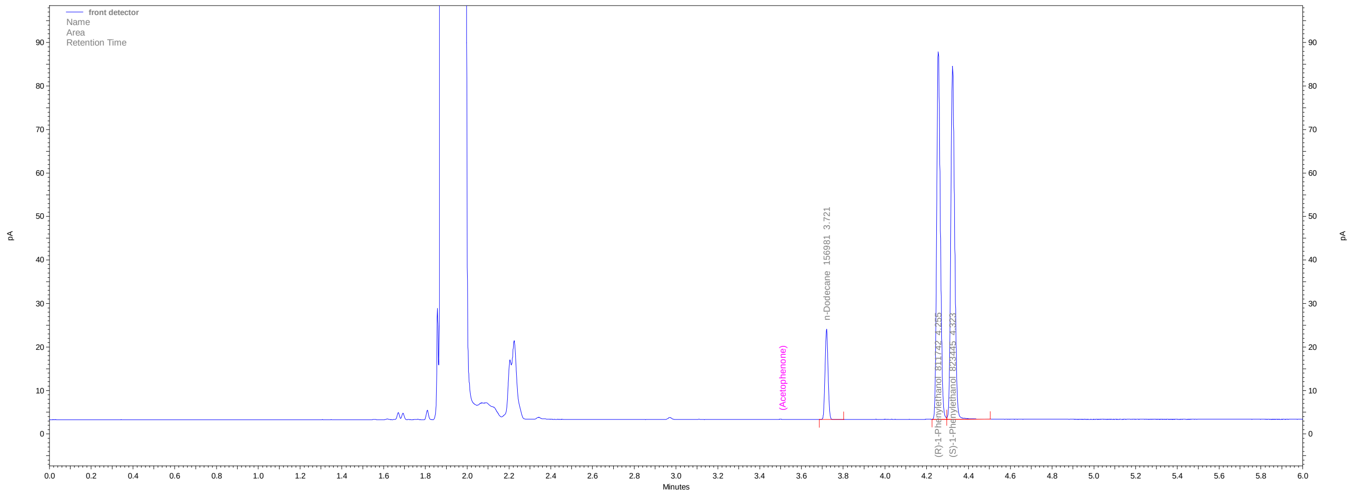

D:\TUD204708\6890 GC Data\Wenjun\WJ197-41-heatfirst.rslt\WJ197-41-heatfirst.dat, front detector

MS-Table 1, entry 9

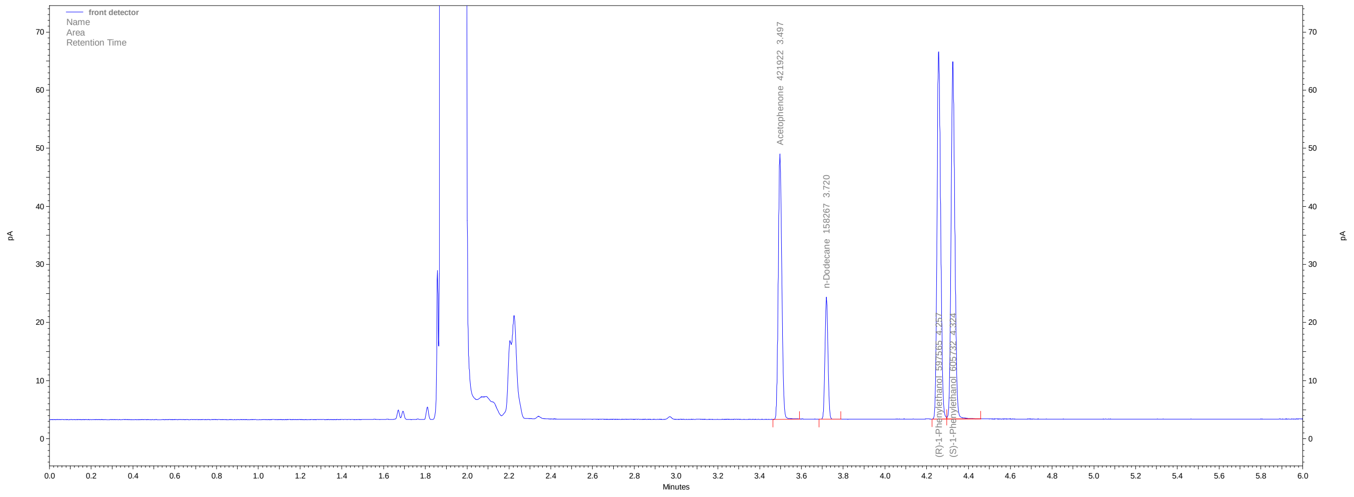

D:\TUD204708\6890 GC Data\Wenjun\WJ197-42-heatfirst.rslt\WJ197-42-heatfirst.dat, front detector

MS-Table 2, entry 1

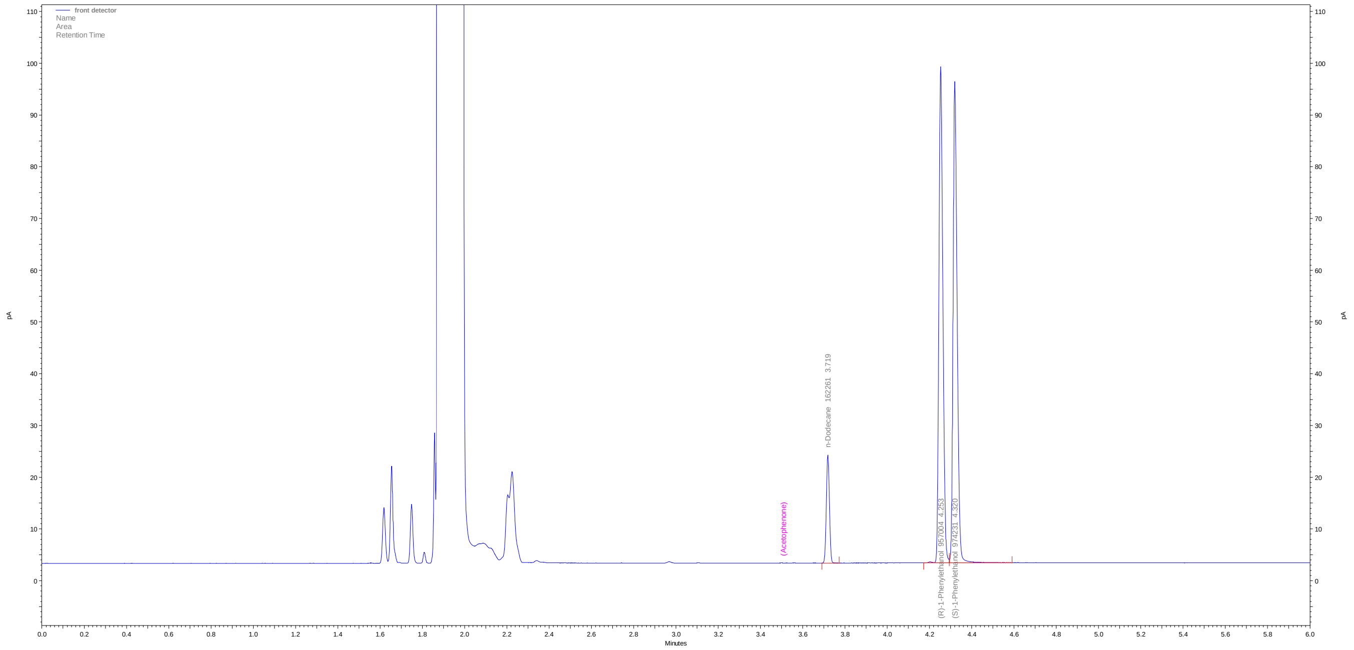

D:\TUD204708\6890 GC Data\Wenjun\WJ197-58-63.rslt\WJ197-59, front detector

MS-Table 2, entry 2

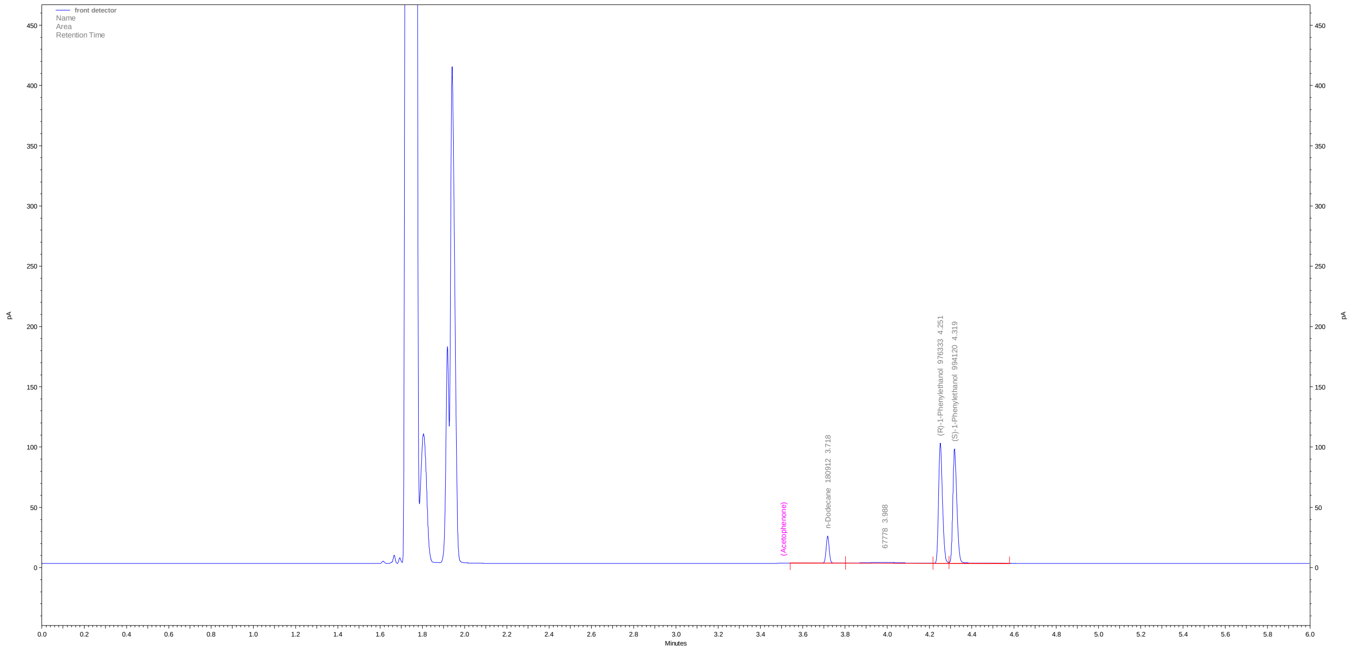

D:\TUD204708\6890 GC Data\Wenjun\WJ196-1-6.rslt\WJ196-5, front detector

MS-Table 2, entry 3

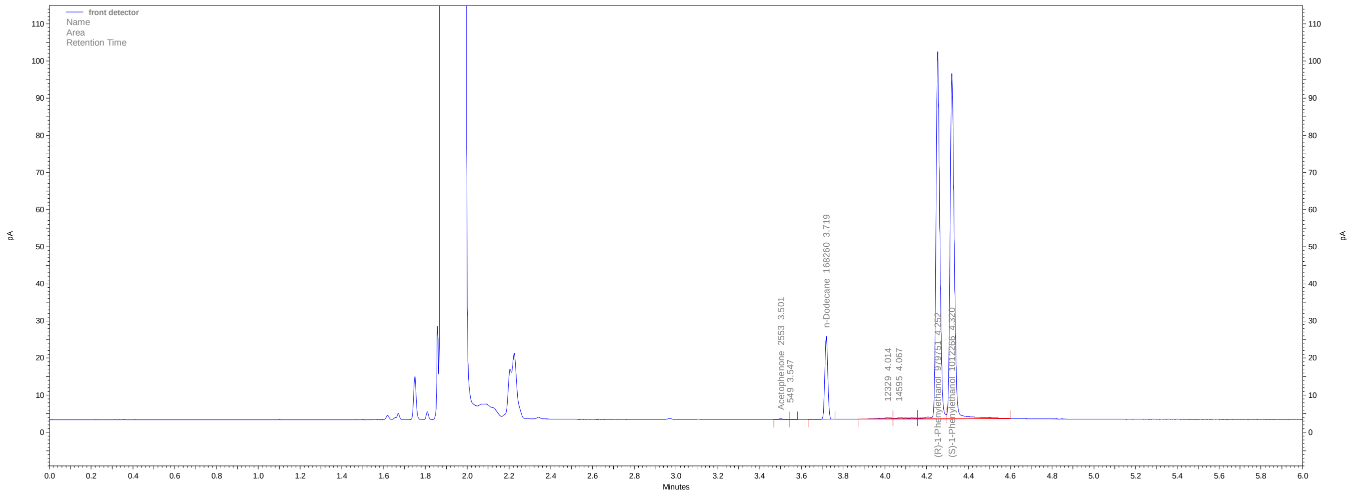

D:\TUD204708\6890 GC Data\Wenjun\WJ196-13-18.rslt\WJ196-16, front detector

MS-Table 2, entry 4

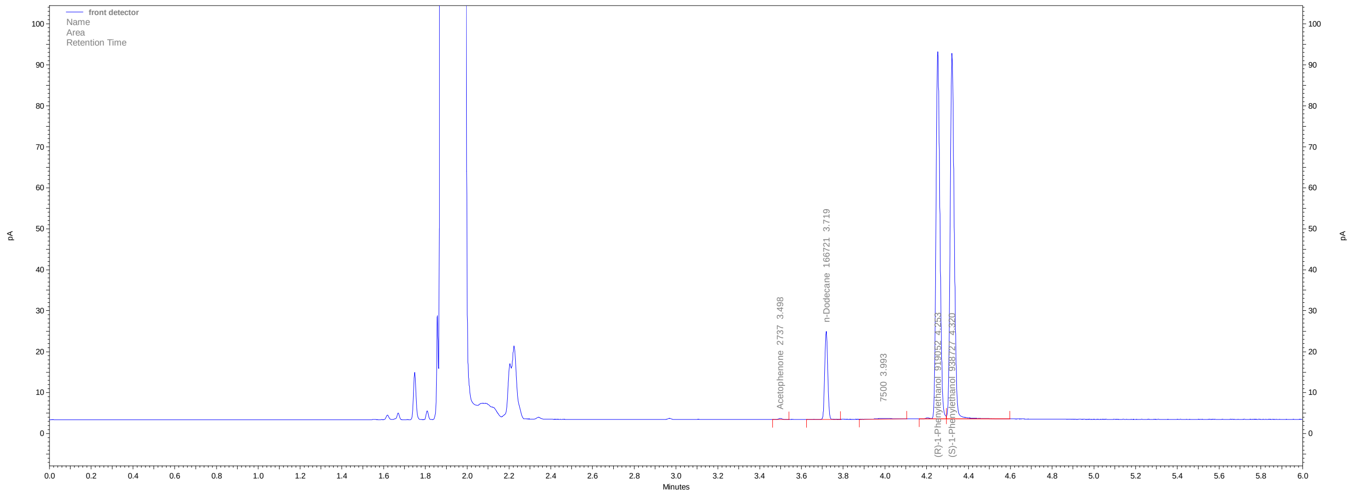

D:\TUD204708\6890 GC Data\Wenjun\WJ196-13-18.rslt\WJ196-17, front detector

MS-Table 2, entry 5

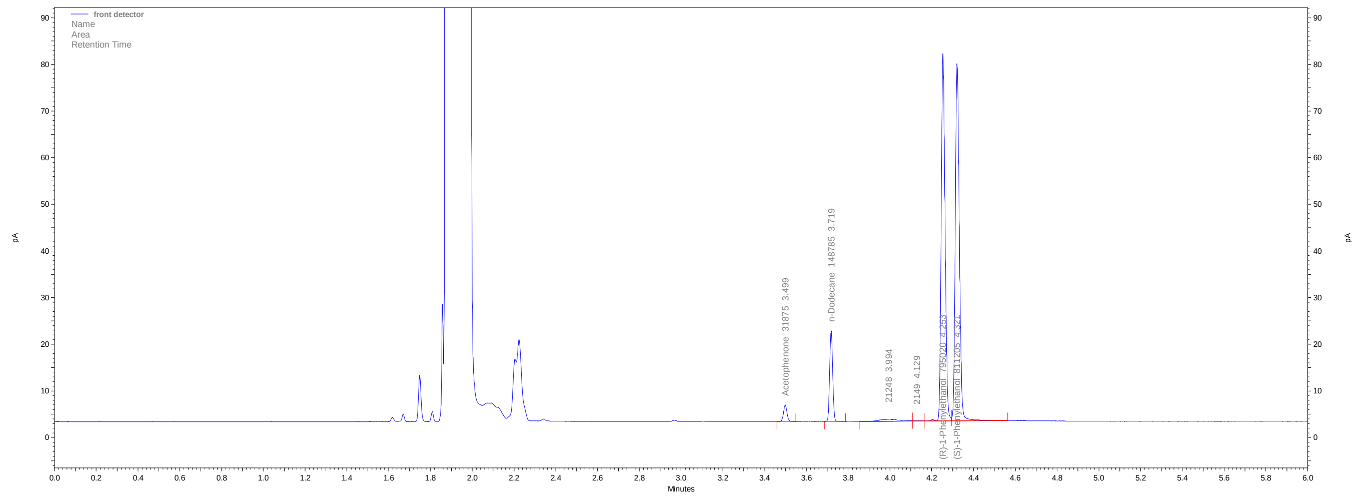

D:\TUD204708\6890 GC Data\Wenjun\WJ196-13-18.rslt\WJ196-18, front detector

MS-Table 2, entry 6

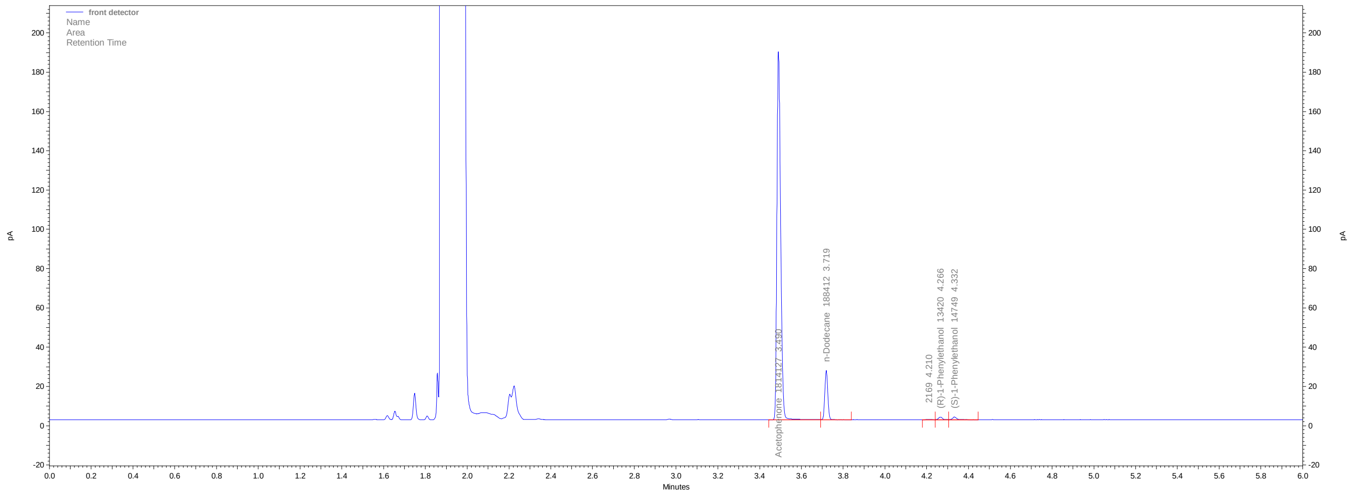

D:\TUD204708\6890 GC Data\Wenjun\wj197-90-95.rslt\WJ197-93.dat, front detector

MS-Table 2, entry 7

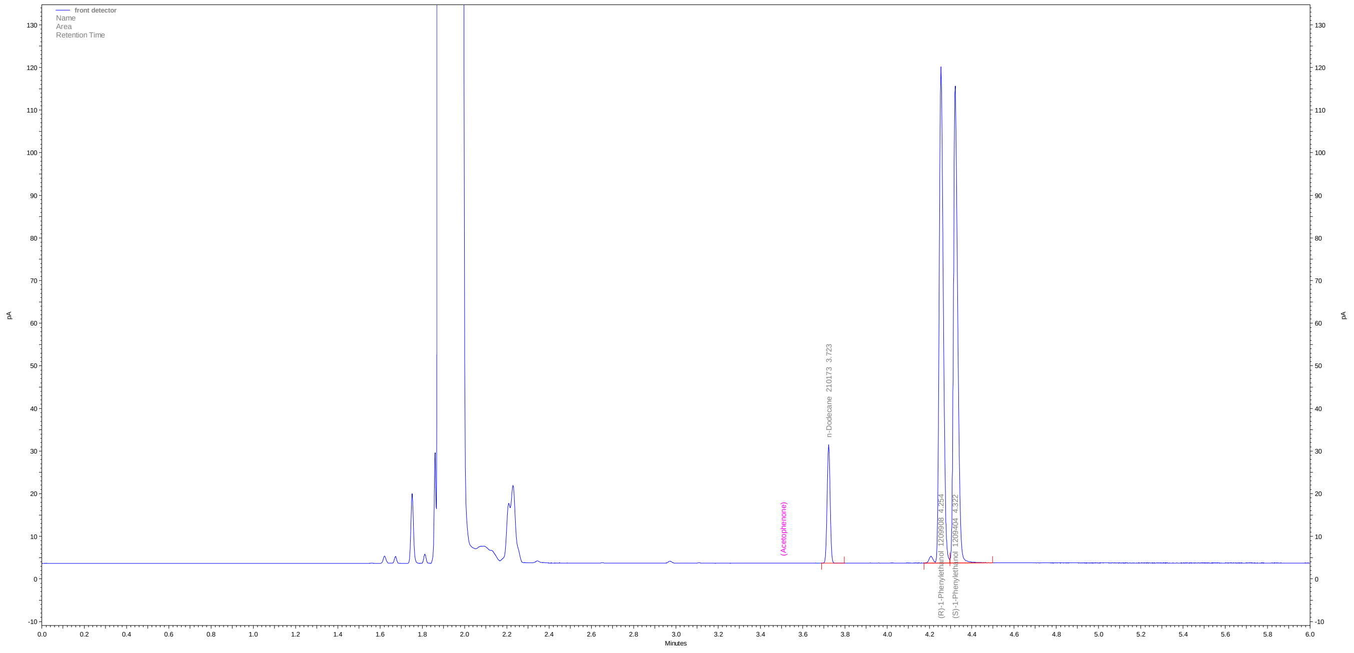

D:\TUD204708\6890 GC Data\Wenjun\WJC7-2.rslt\WJC7-2.dat, front detector

Supplementary-Table 1, entry 1

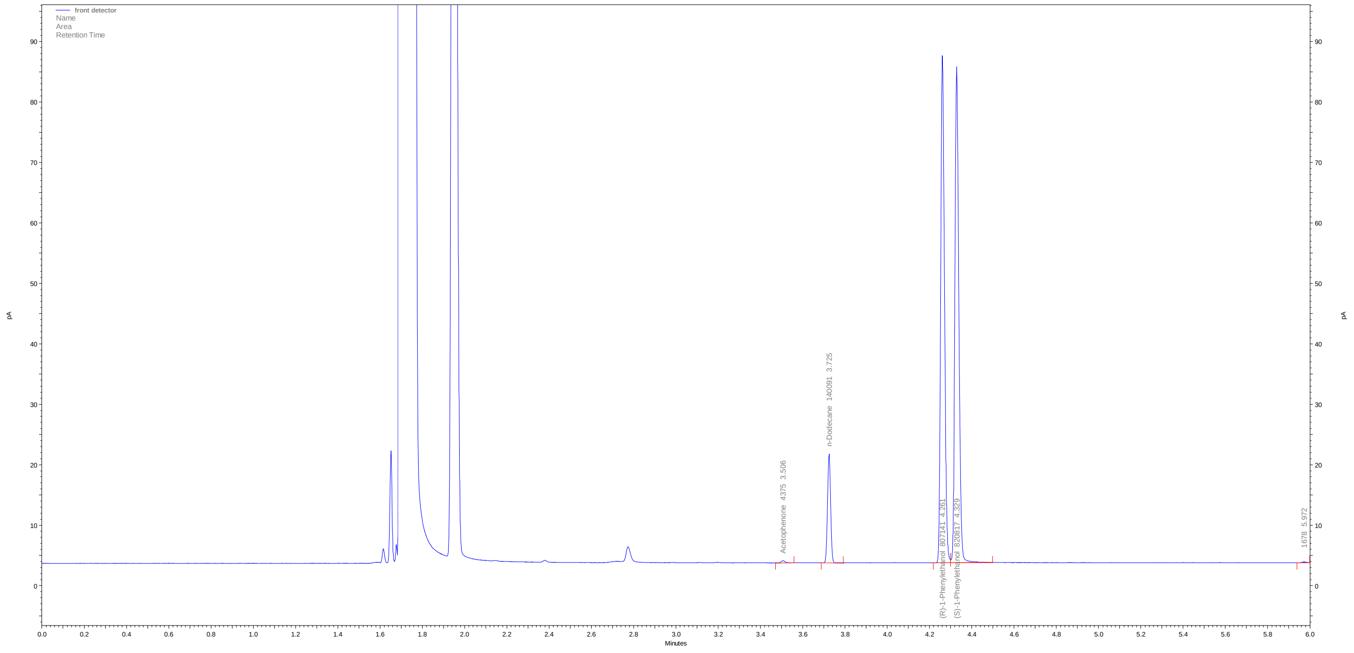

D:\TUD204708\6890 GC Data\ROBIN\RvS200ppmdioxane-17-07-19.rslt\RvS200ppmdioxane-17-07-19.dat, front detector

Supplementary-Table 1, entry 2

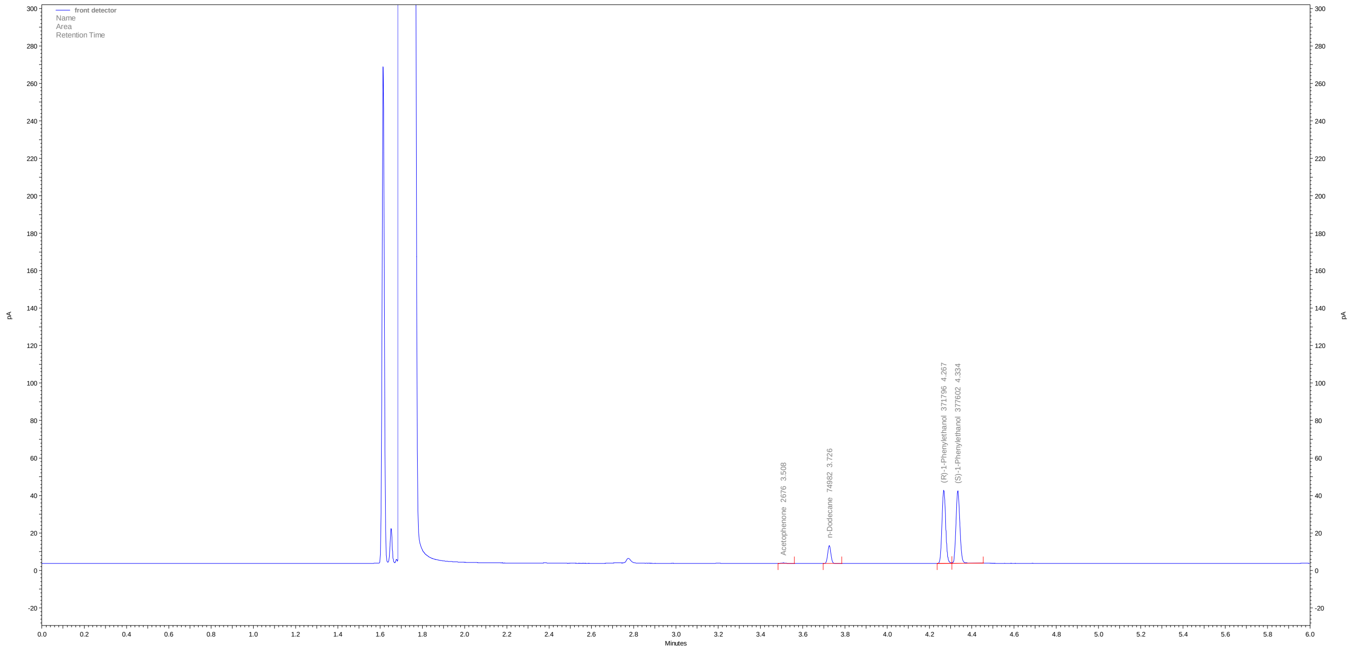

D:\TUD204708\6890 GC Data\ROBIN\RvS200ppmEt2O-19-07-19.rslt\RvS200ppmEt2O-19-07-19.dat, front detector

Supplementary-Table 1, entry 3

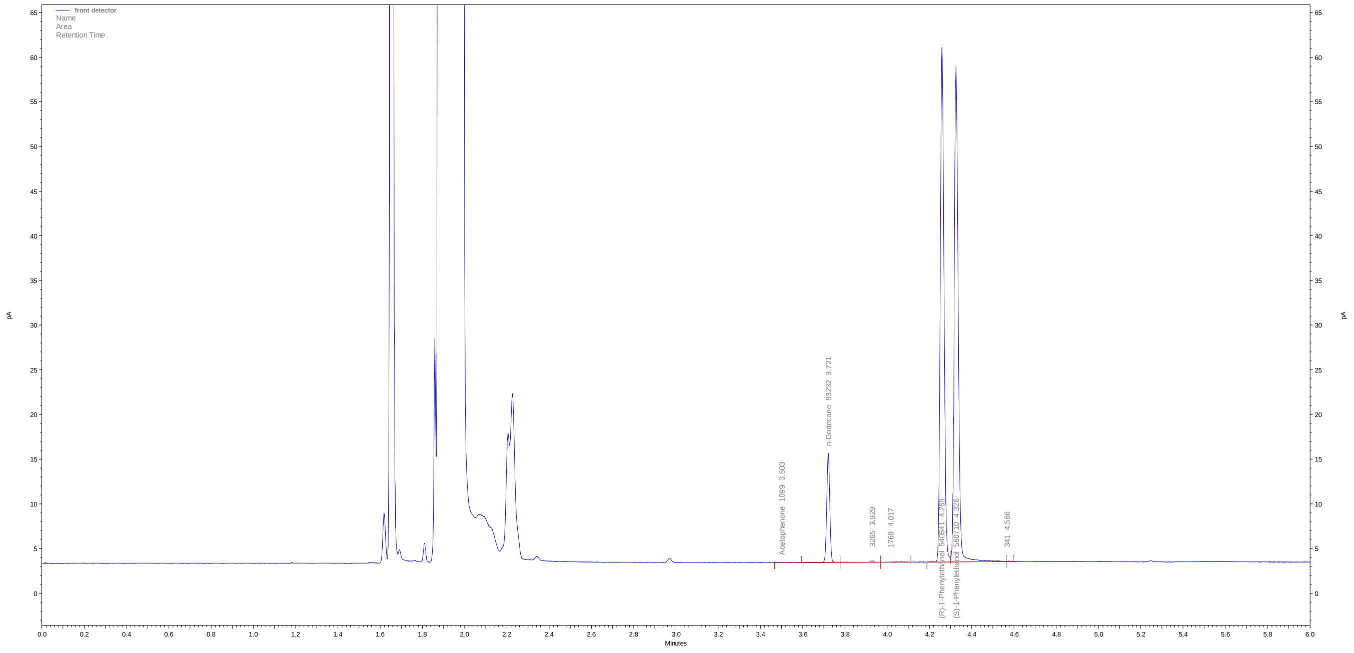

D:\TUD204708\6890 GC Data\Wenjun\WJ193-1-6-191-7-12.rslt\WJ193-3, front detector

Supplementary-Table 1, entry 4

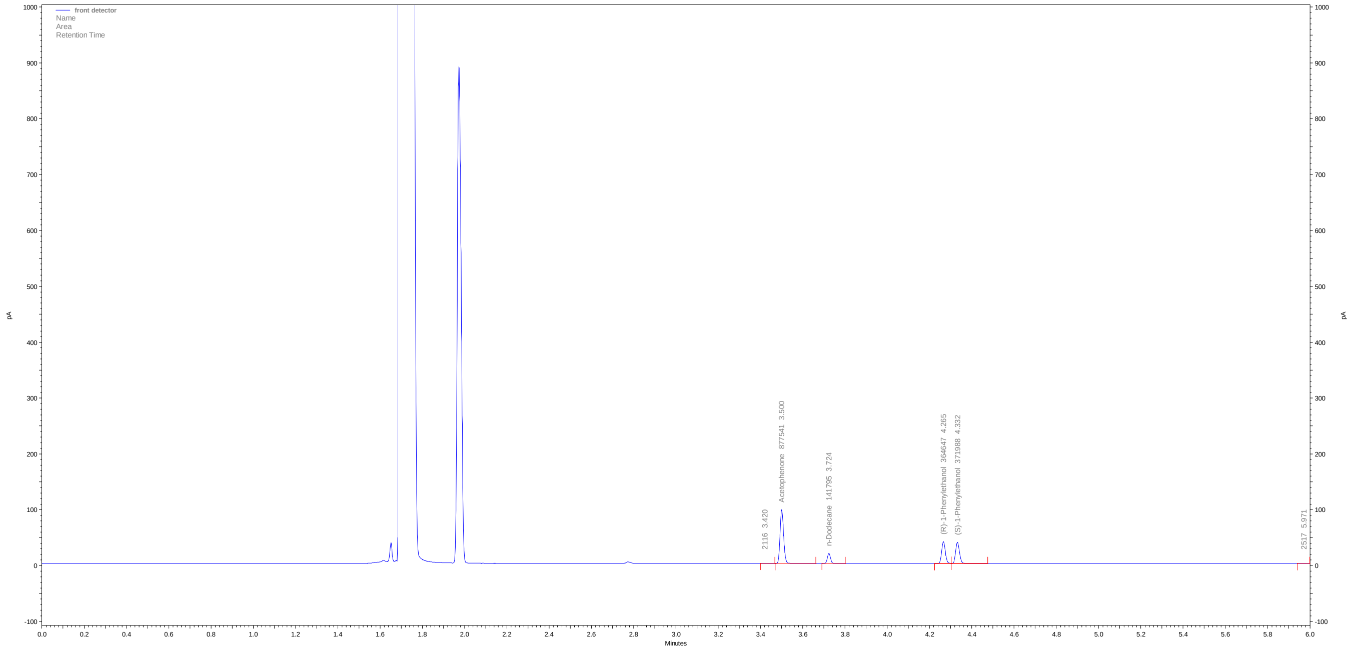

D:\TUD204708\6890 GC Data\ROBIN\RvS200ppmtoluene2-18-07-19.rslt\RvS200ppmtoluene2-18-07-19.dat, front detector

Supplementary-Table 1, entry 5

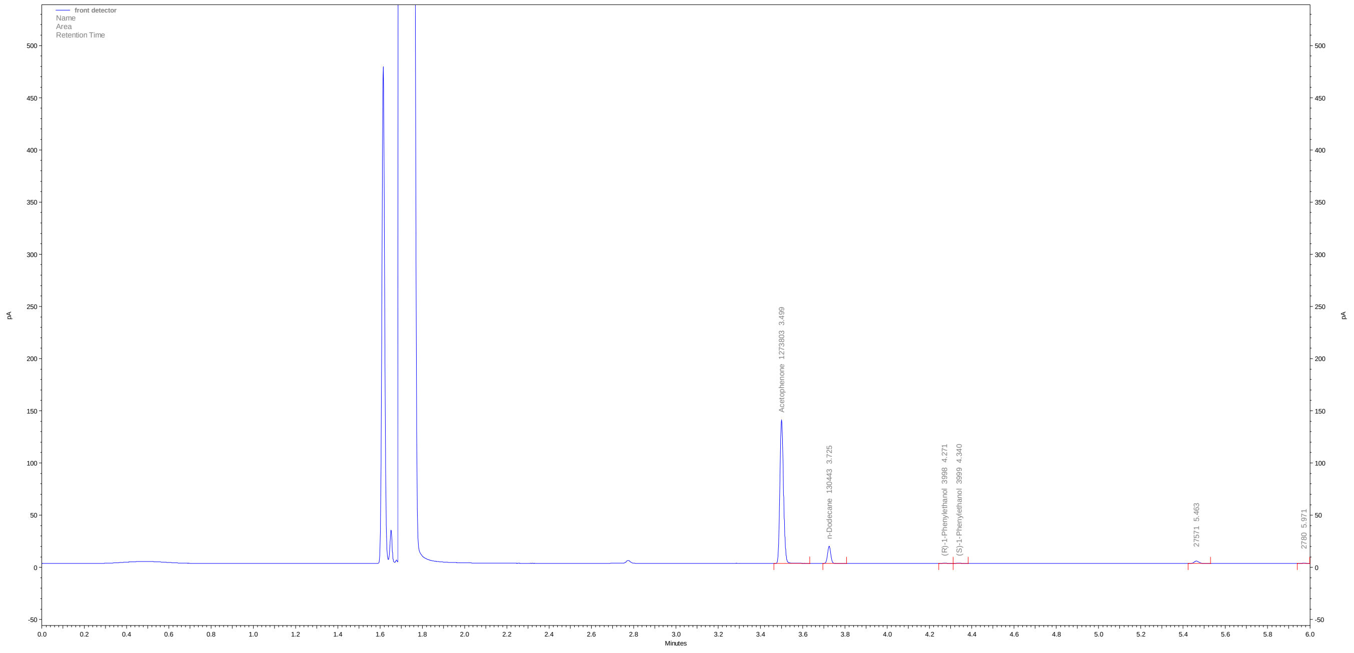

D:\TUD204708\6890 GC Data\ROBIN\RvS200ppmMeCN-20-07-19.rslt\RvS200ppmMeCN-20-07-19.dat, front detector

Supplementary-Table 1, entry 6

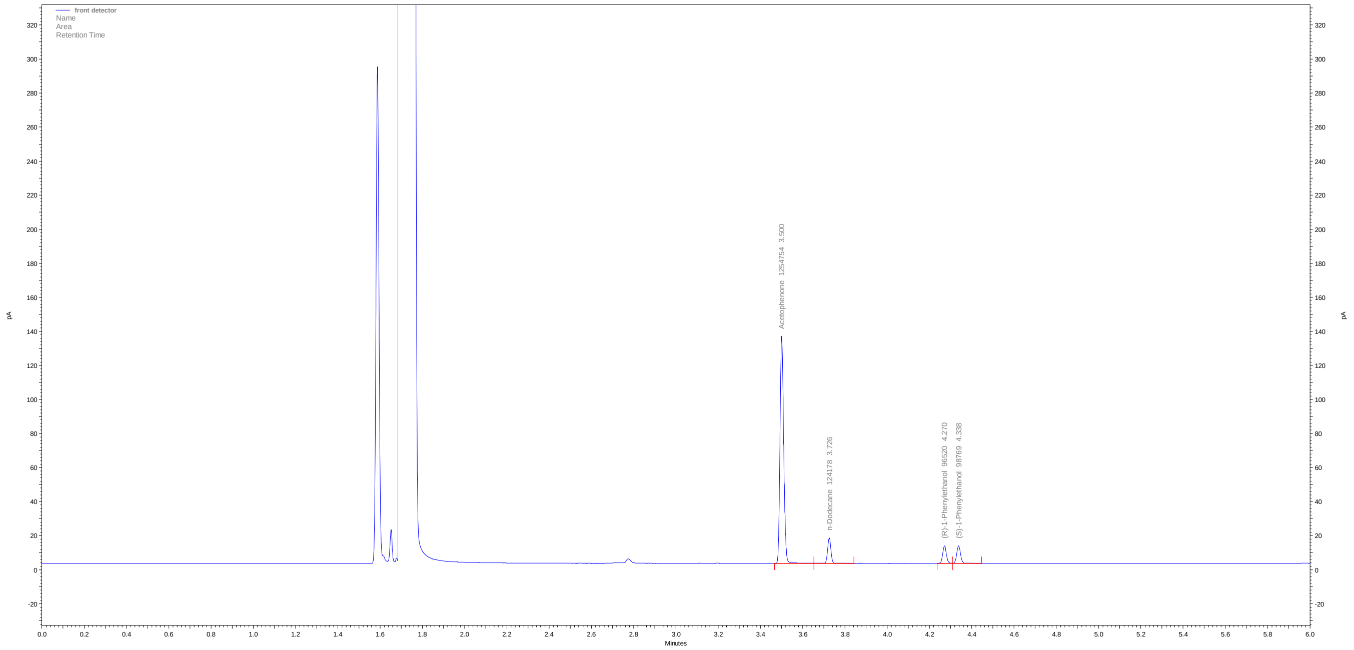

D:\TUD204708\6890 GC Data\ROBIN\RvS200ppmMeOH-20-07-19.rslt\RvS200ppmMeOH-20-07-19.dat, front detector

Supplementary-Table 1, entry 7

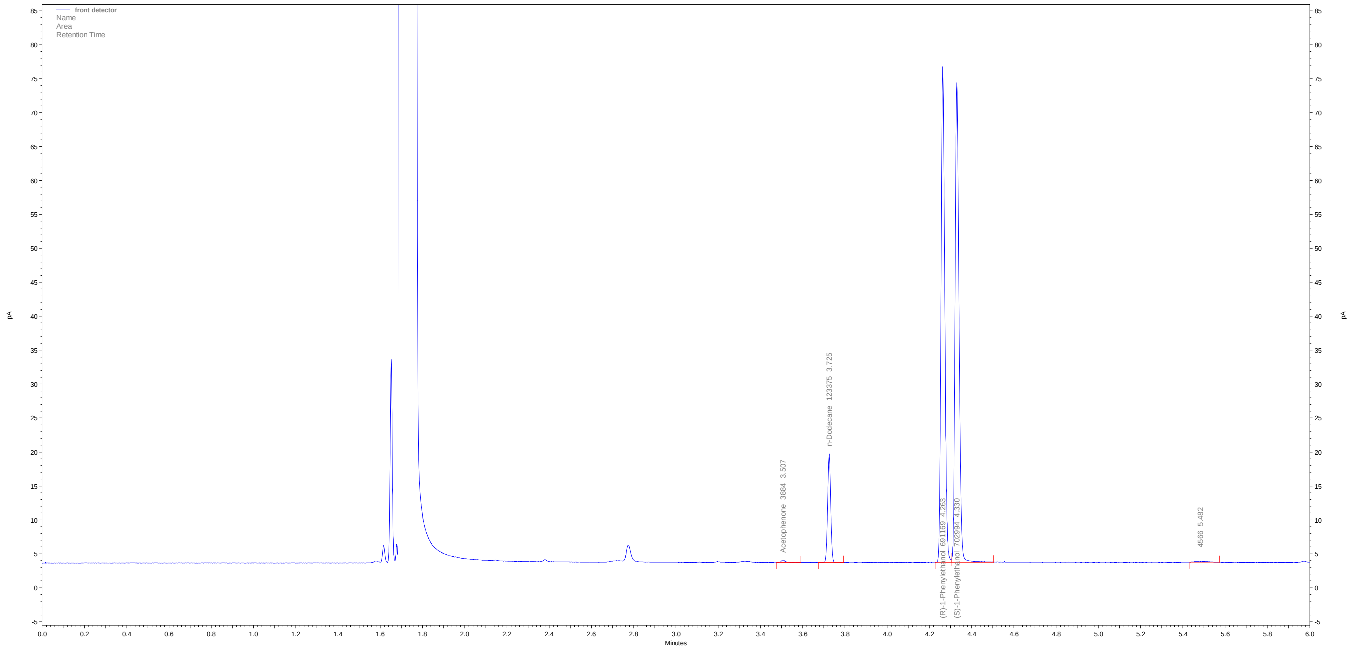

D:\TUD204708\6890 GC Data\ROBIN\RvS200ppmTHF2-18-07-19.rslt\RvS200ppmTHF2-18-07-19.dat, front detector

Supplementary-Table 1, entry 8

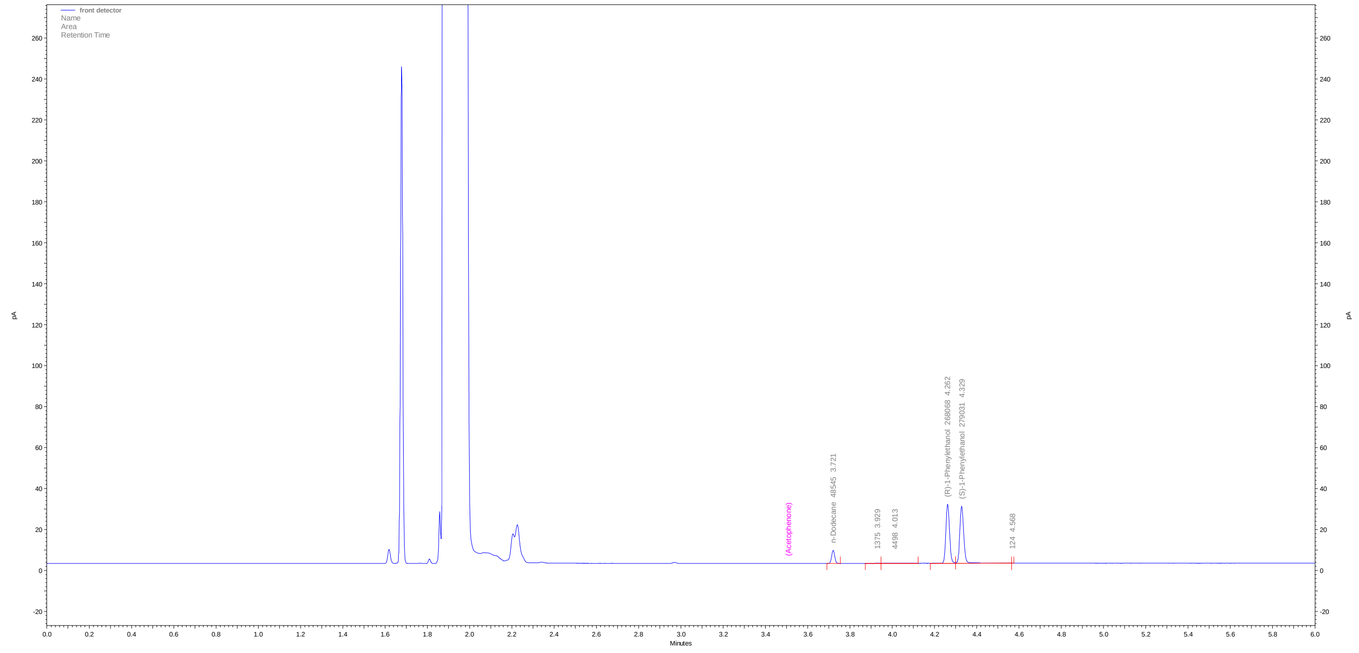

D:\TUD204708\6890 GC Data\Wenjun\WJ193-1-6-191-7-12.rslt\WJ193-4, front detector

Supplementary-Table 1, entry 9

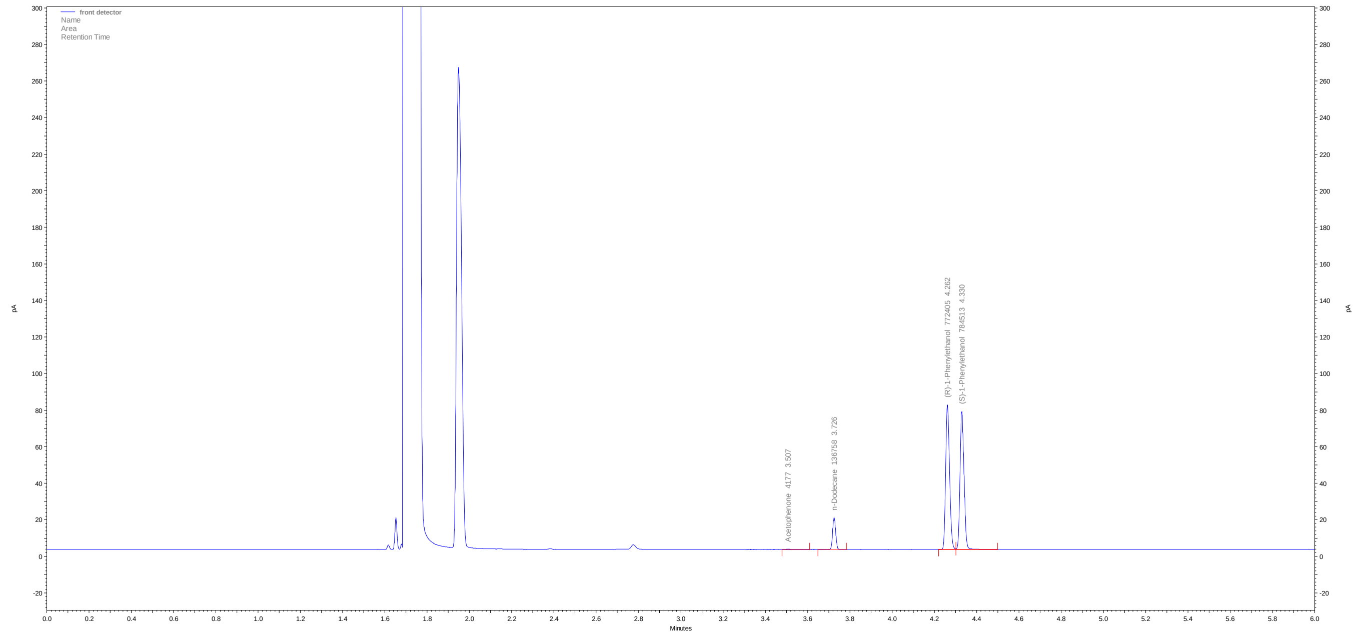

D:\TUD204708\6890 GC Data\Wenjun\WJ155-1.rslt\WJ155-1.dat, front detector

Supplementary-Table 1, entry 10

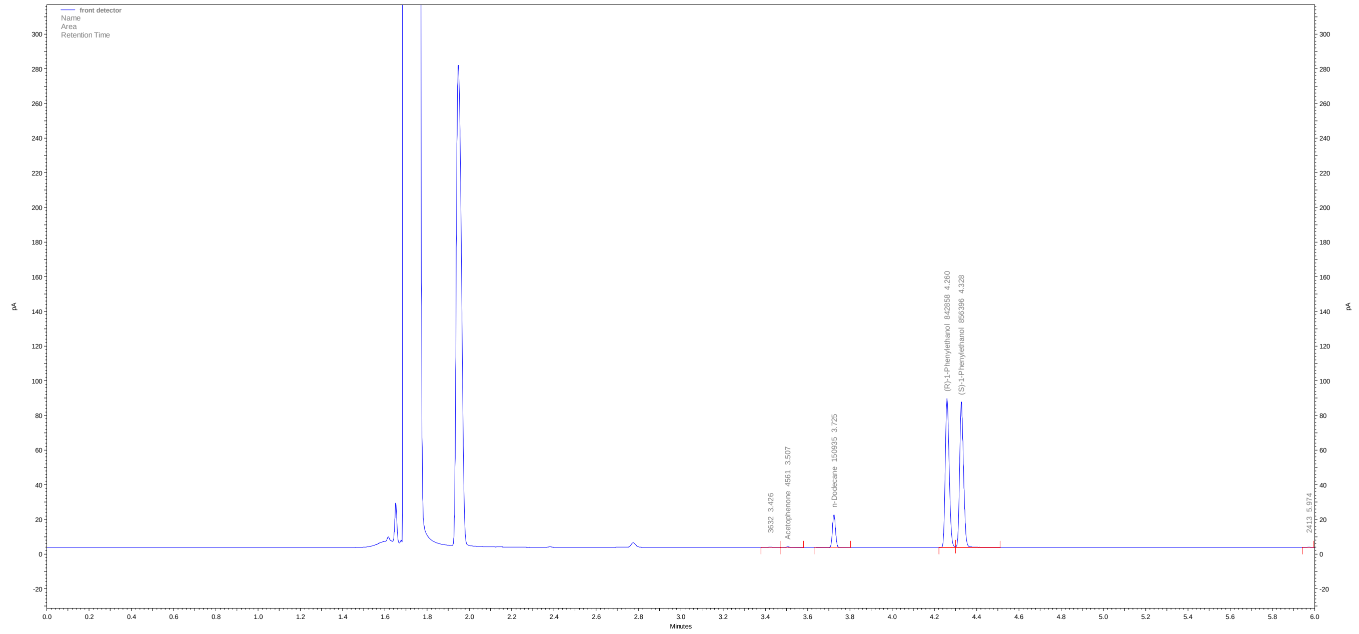

D:\TUD204708\6890 GC Data\Wenjun\WJ155-2.rslt\WJ155-2.dat, front detector

Supplementary-Table 1, entry 11

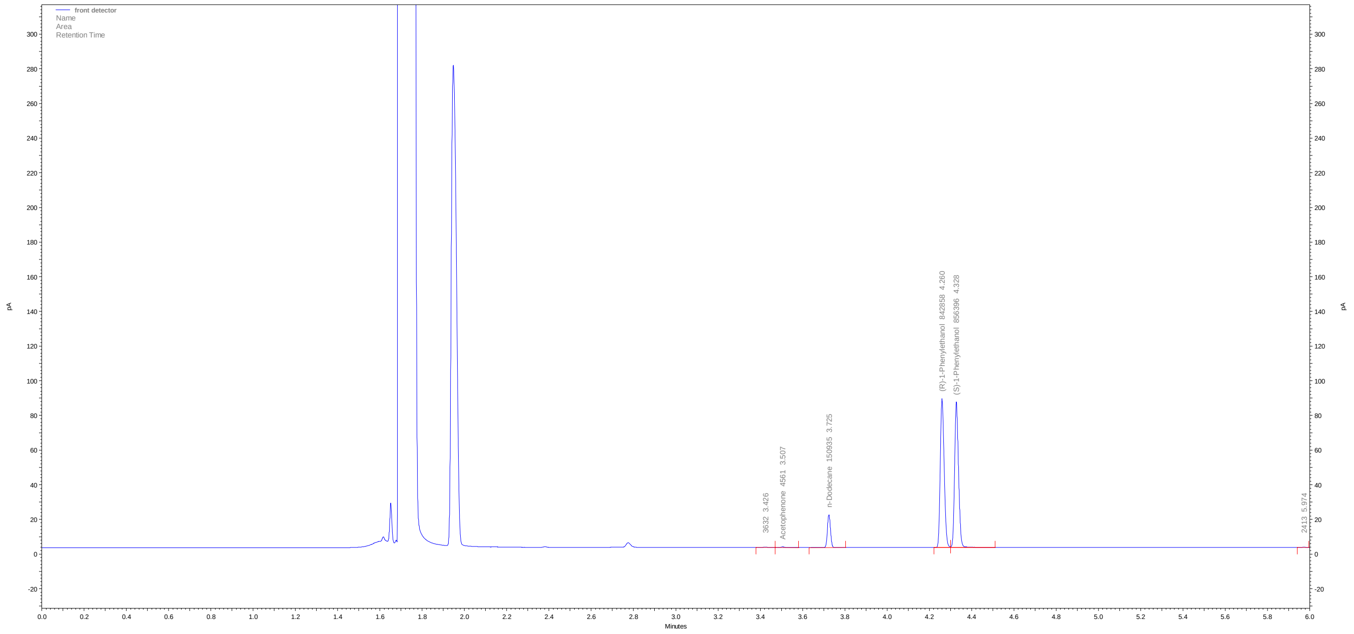

D:\TUD204708\6890 GC Data\Wenjun\WJ155-2.rslt\WJ155-2.dat, front detector

Supplementary-Table 1, entry 12

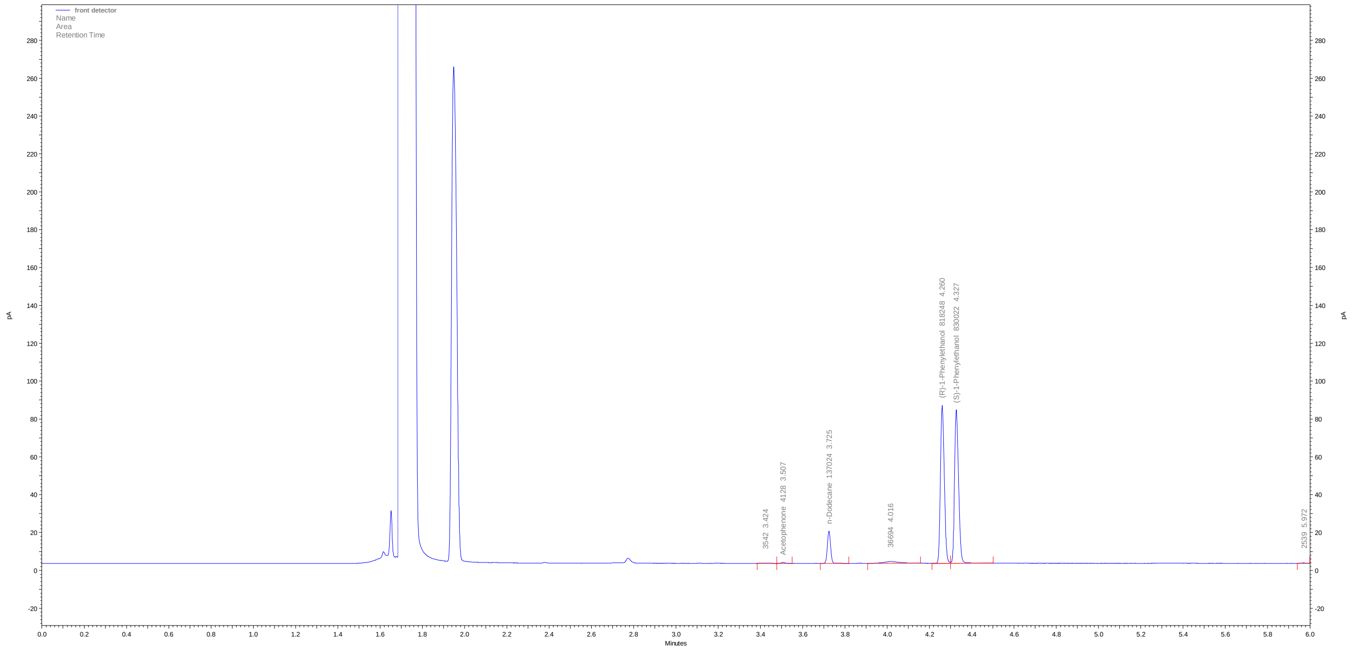

D:\TUD204708\6890 GC Data\Wenjun\WJ155-4.rslt\WJ155-4.dat, front detector

Supplementary-Table 1, entry 13

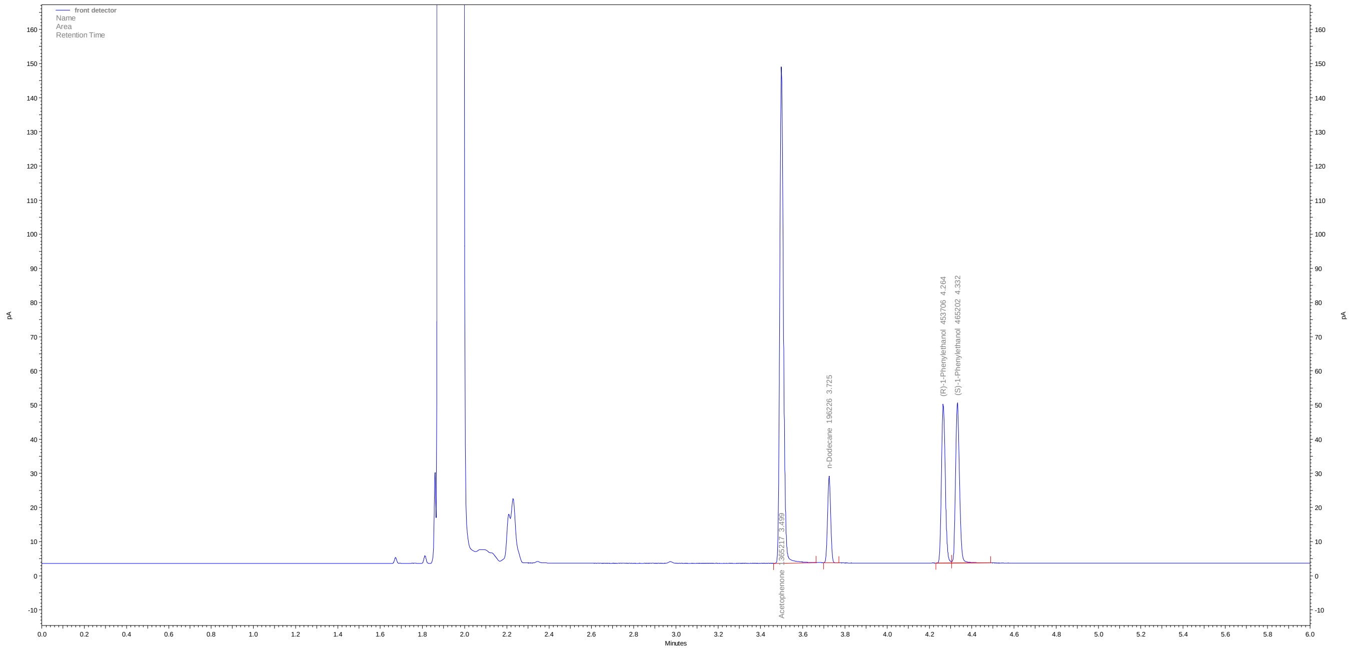

D:\TUD204708\6890 GC Data\Wenjun\wj-175-3.rslt\wj-175-3.dat, front detector

Supplementary-Table 1, entry 14

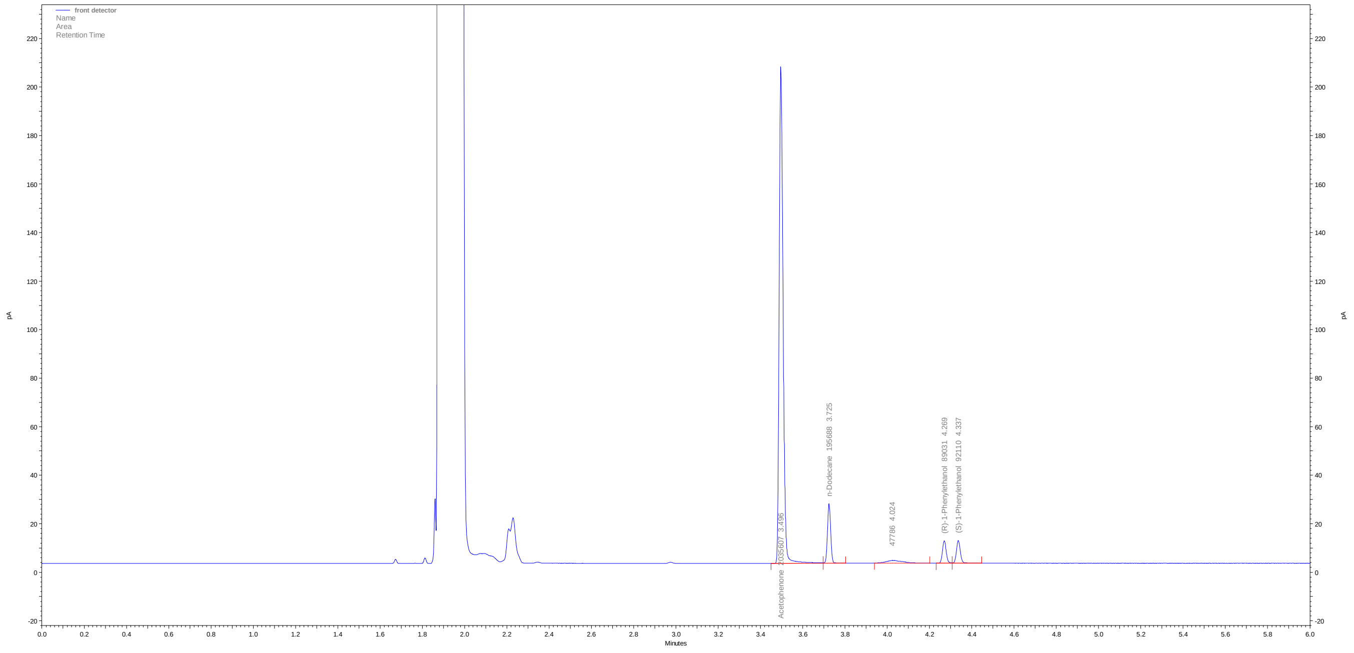

D:\TUD204708\6890 GC Data\Wenjun\wj-175-4.rslt\wj-175-4.dat, front detector

Supplementary-Table 2, entry 1

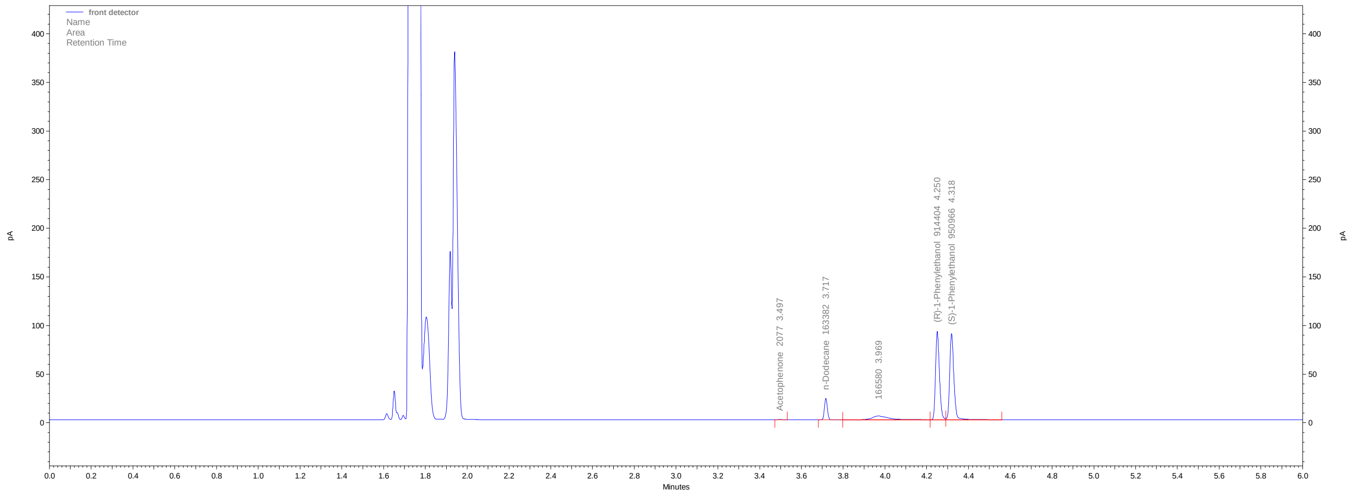

D:\TUD204708\6890 GC Data\Wenjun\WJ197-120.rslt\WJ197-120.dat, front detector

Supplementary-Table 2, entry 2

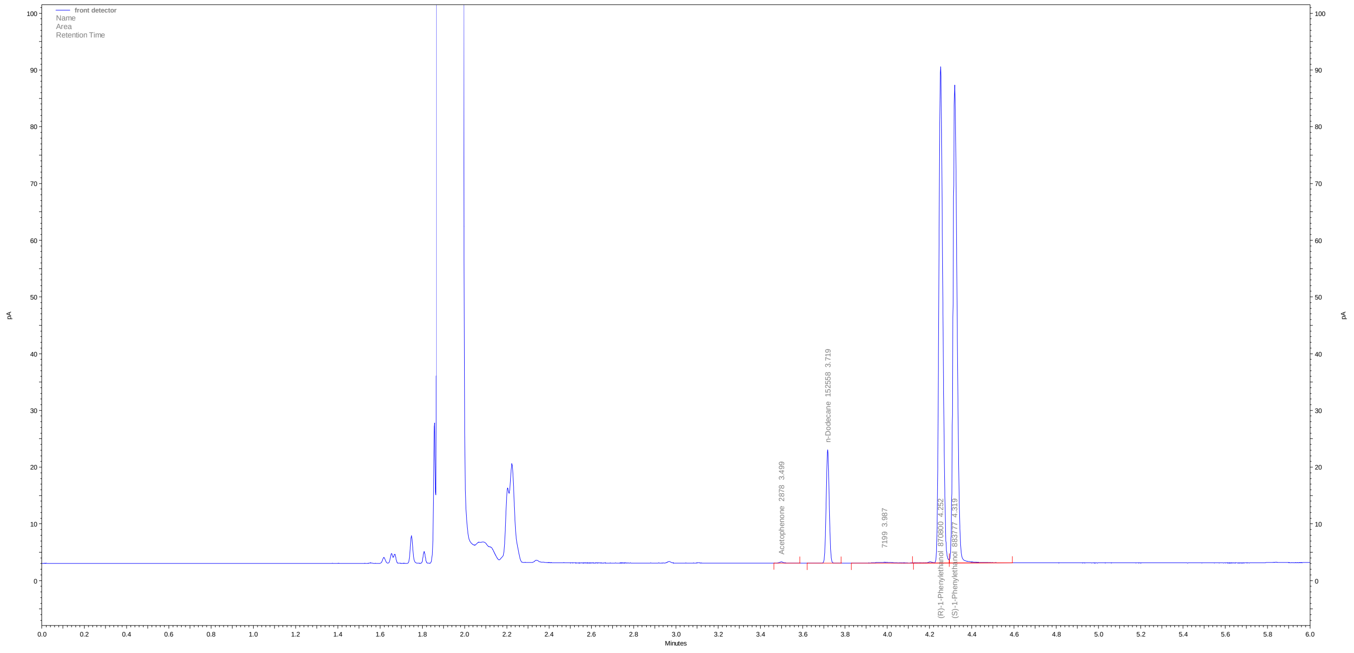

D:\TUD204708\6890 GC Data\Wenjun\wj197-90-95.rslt\WJ197-92, front detector

Supplementary-Table 2, entry 3

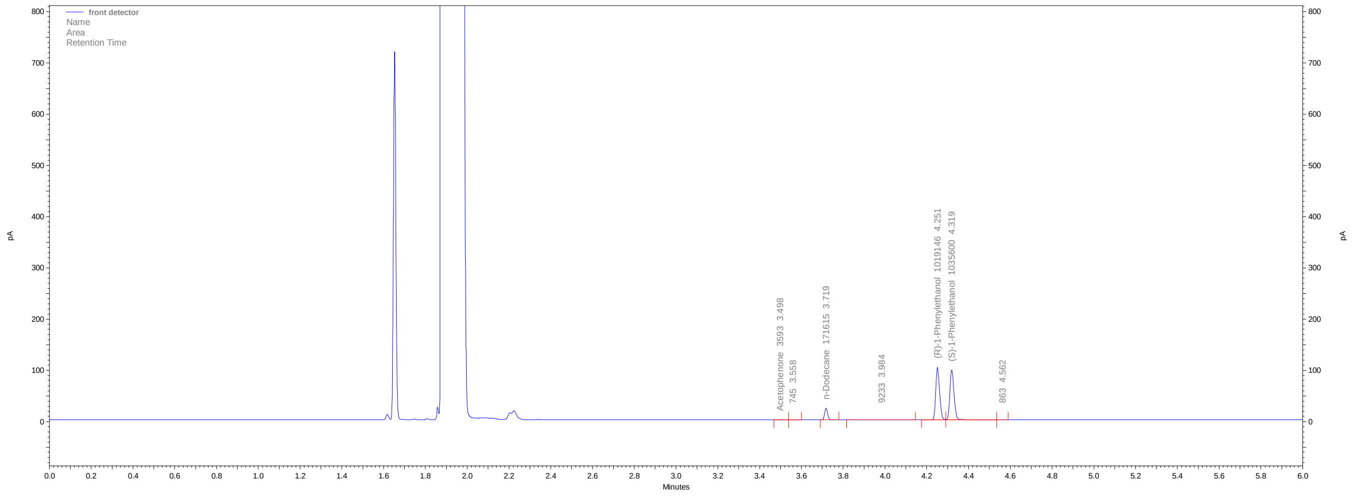

D:\TUD204708\6890 GC Data\Wenjun\WJ197-76-81.rslt\WJ197-79, front detector

Supplementary-Table 2, entry 4

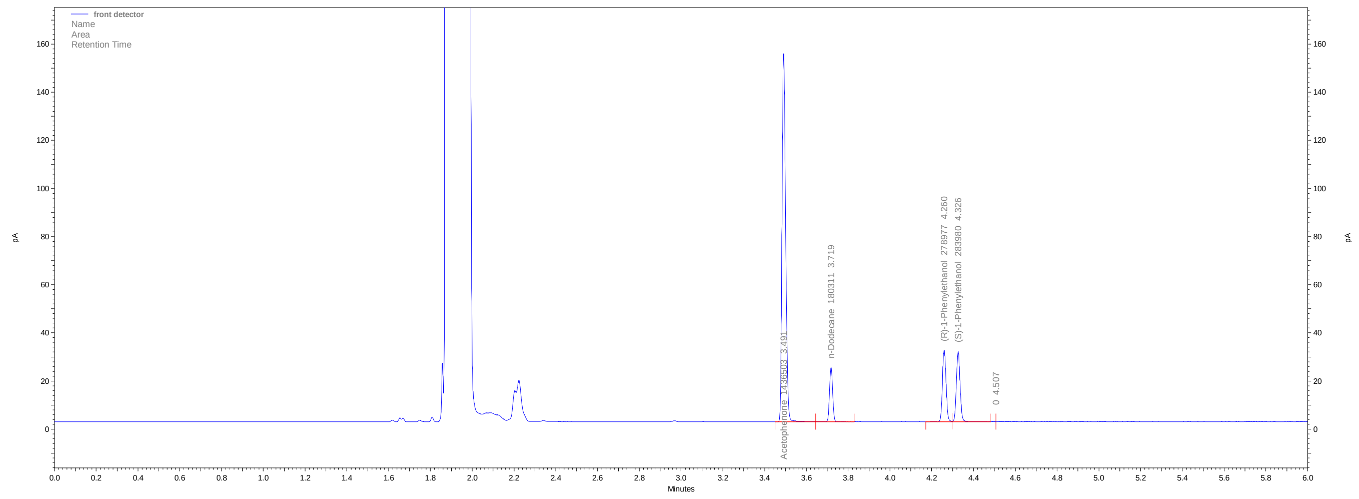

D:\TUD204708\6890 GC Data\Wenjun\wj197-90-95.rslt\WJ197-95, front detector

Supplementary-Table 2, entry 5

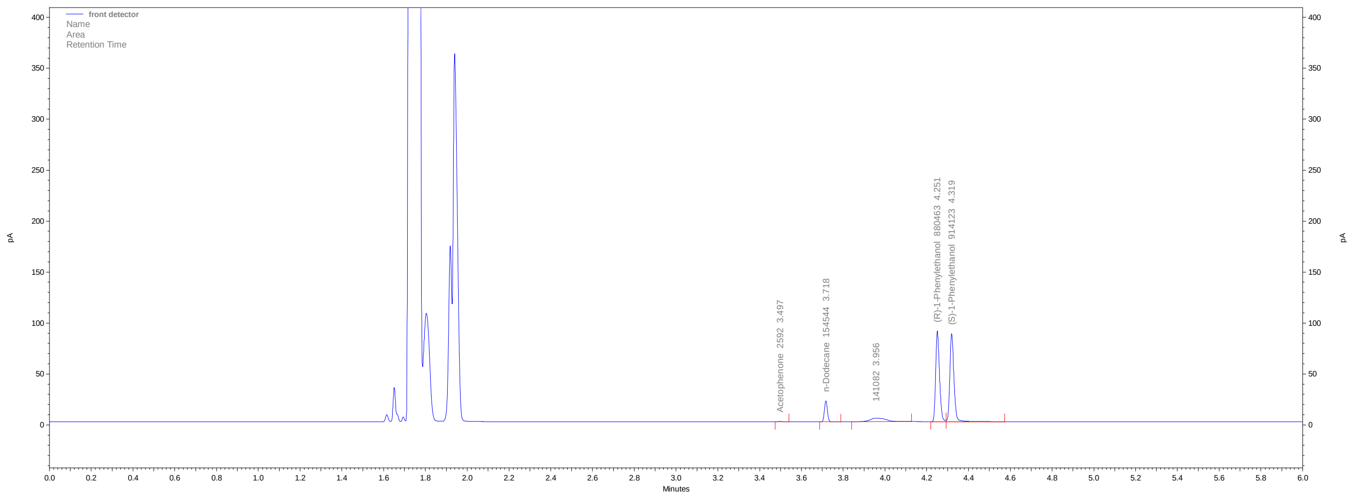

D:\TUD204708\6890 GC Data\Wenjun\WJ197-121.rslt\WJ197-121.dat, front detector

Supplementary-Table 2, entry 6

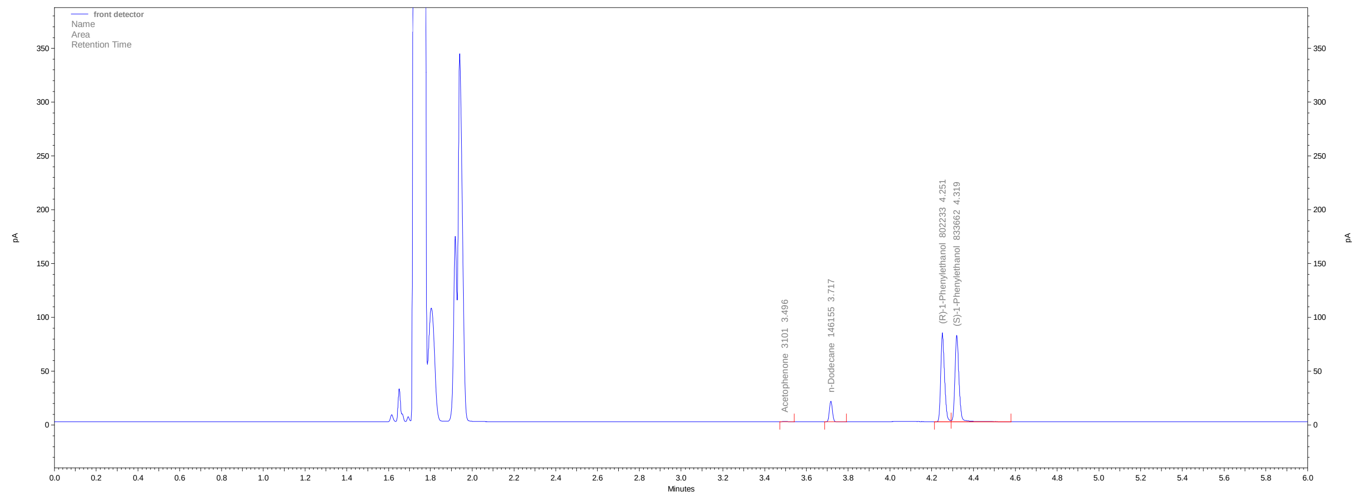

D:\TUD204708\6890 GC Data\Wenjun\WJ197-122.rslt\WJ197-122.dat, front detector

Supplementary-Table 2, entry 7

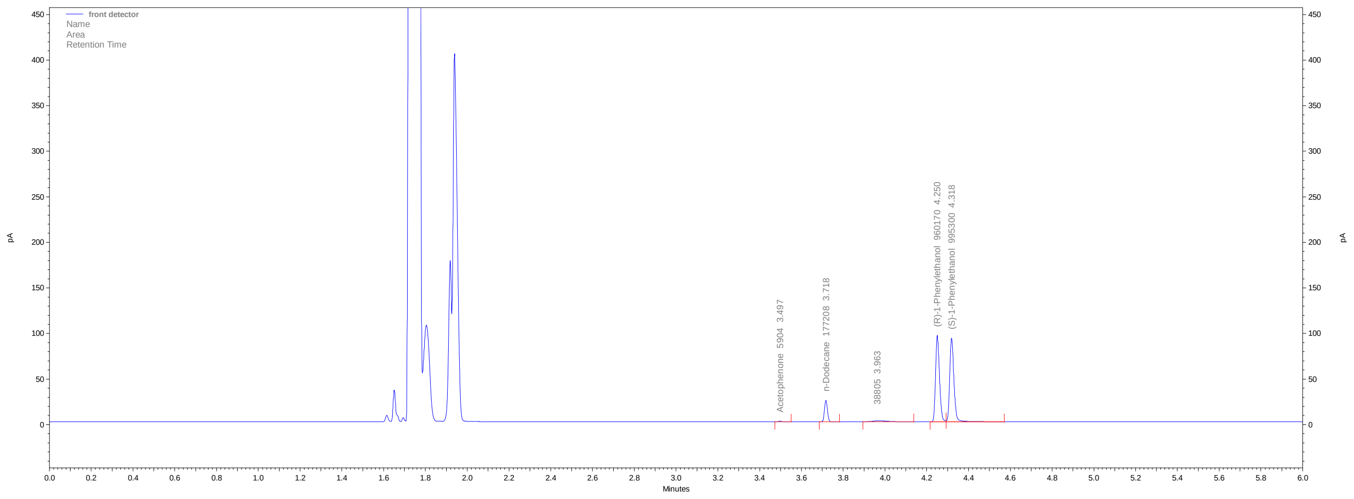

D:\TUD204708\6890 GC Data\Wenjun\WJ197-124.rslt\WJ197-124.dat, front detector

Supplementary-Table 2, entry 8

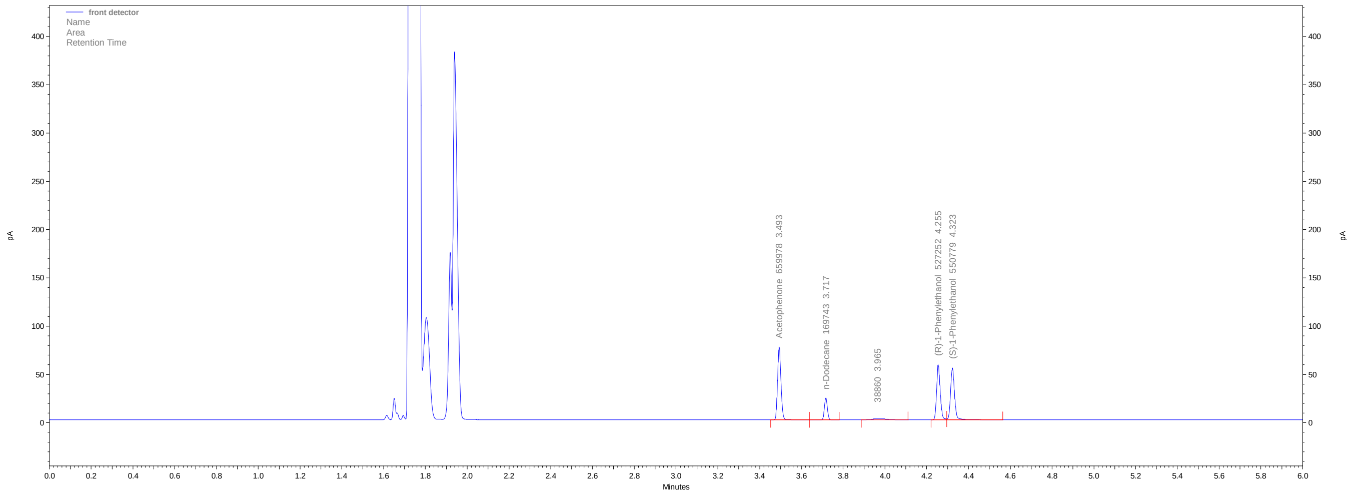

D:\TUD204708\6890 GC Data\Wenjun\WJ197-125.rslt\WJ197-125.dat, front detector

Supplementary-Table 2, entry 9

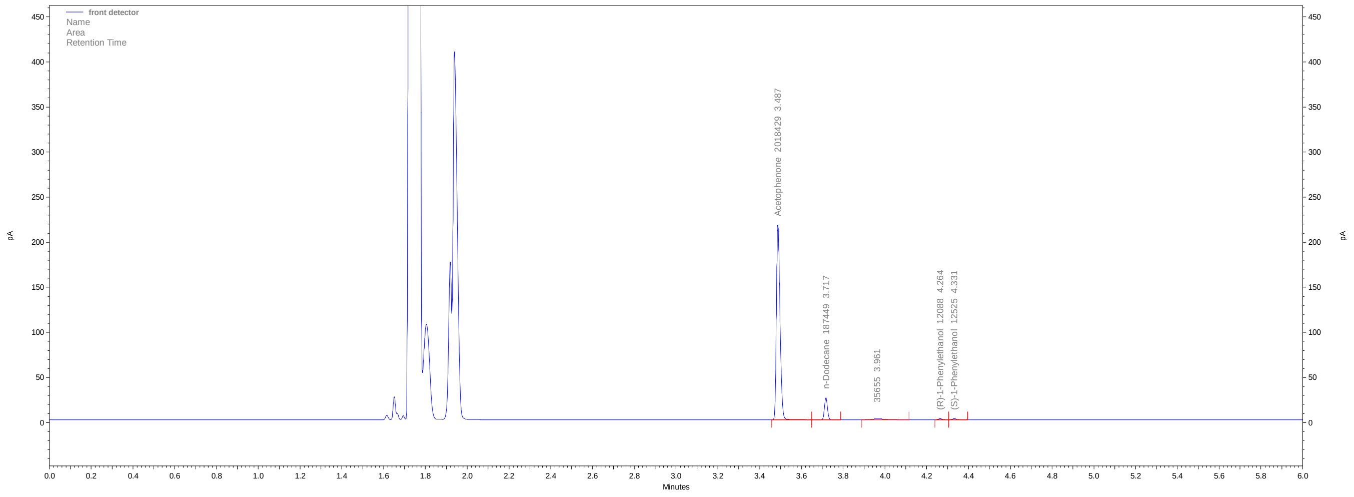

D:\TUD204708\6890 GC Data\Wenjun\WJ197-123.rslt\WJ197-123.dat, front detector
