# Supplementary material for: Robust and efficient hydrogenation of carbonyl compounds catalysed by mixed donor Mn(I) pincer complexes
Source: Nat Commun. 2021 Jan 4;12:12. doi: 10.1038/s41467-020-20168-2 (PMC7782525; doi:10.1038/s41467-020-20168-2)
Supplement: Supplementary file 3 — Source Data [file 41467_2020_20168_MOESM3_ESM.zip › CNP dataset/GC-MS of substrate scope.pdf]

# Single Injection Report

Agilent Technologies

**Data file:** D:\CDSProjects\GCMS Liliana\Results\Wenjun\WJ206-50ppm-25bar-IPA-3.sirslt\WJ206-50ppm-25bar-IPA-3.dx

**Sample name:** WJ206-50ppm-25bar-IPA-3

**Description:**

**Sample amount:** 0.000

**Instrument:** GCMS

**Injection date:** 2020-01-11 16:20:15+01:00

**Acq. method:** DB5MS 60-300_WY.amx

**Analysis method:** Bob_FID+MS.pmx

**Last changed:** 2019-08-30 14:05:20+02:00

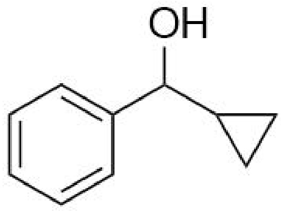


**9a**, 98%

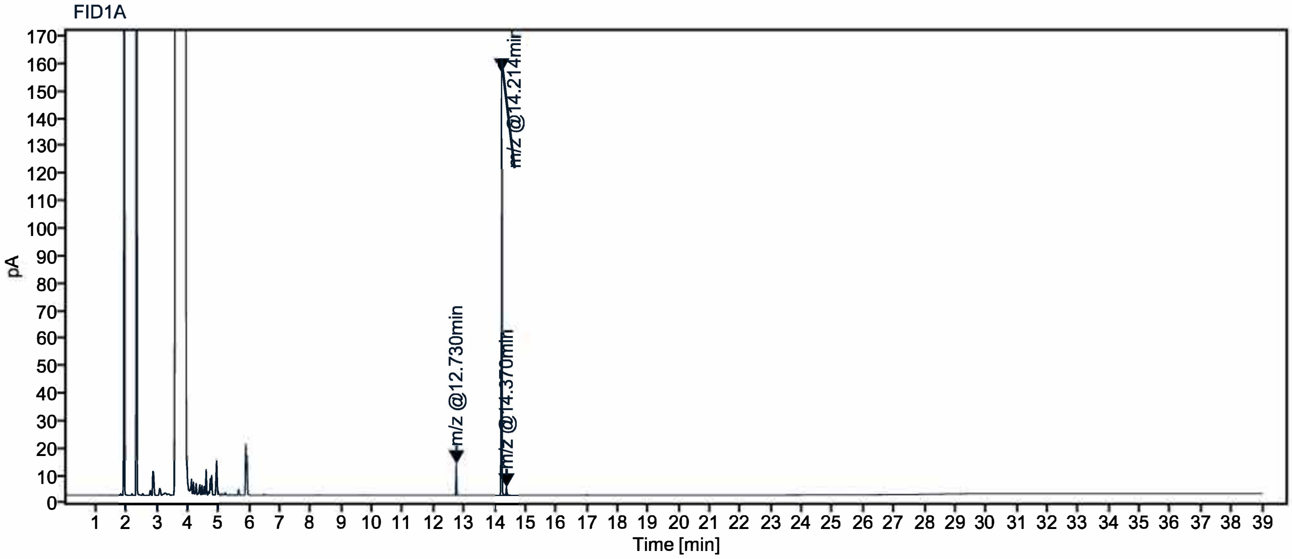


**Signal:** FID1A

| RT [min] | Area | Height |
|---|---|---|
| 12.730 | 15.6765 | 11.5164 |
| 14.214 | 240.1210 | 154.1037 |
| 14.370 | 5.0100 | 3.1329 |
| Sum | 260.8075 | |

**Signal:** MS1Front TIC SCAN EI

| RT [min] | Area | Height |
|---|---|---|
| 12.752 | 480644.5763 | 364800.7300 |
| 14.236 | 7862476.5096 | 5517057.8007 |
| 14.393 | 124863.6816 | 86508.2805 |
| Sum | 8467984.7675 | |

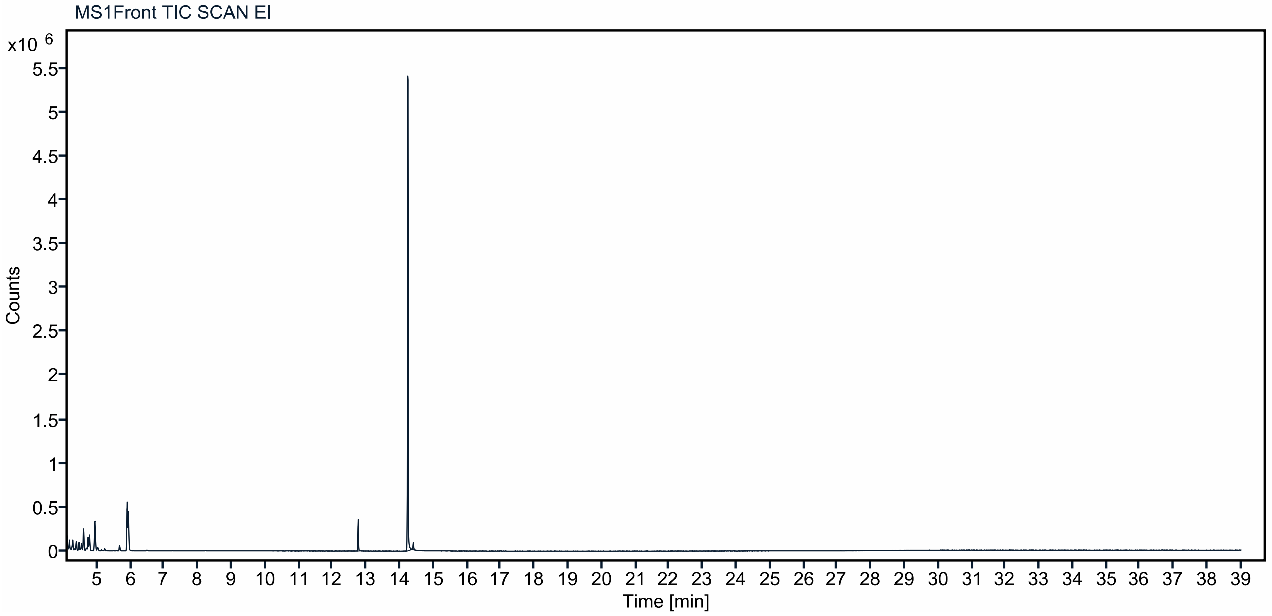


| RT | Compound Name | Score | Rev. Score | Prob. % | CAS # | Library Id |
|---|---|---|---|---|---|---|
| 12.752 | Dodecane | 925 | 930 | 20.34 | 112-40-3 | 26119 |

| RT | Compound Name | Score | Rev. Score | Prob. % | CAS # | Library Id |
|---|---|---|---|---|---|---|
| 14.236 | Cyclopropyl phenylmethanol | 909 | 910 | 85.52 | 1007-03-0 | 110604 |

| RT | Compound Name | Score | Rev. Score | Prob. % | CAS # | Library Id |
|---|---|---|---|---|---|---|
| 14.393 | Cyclopropyl phenyl ketone | 883 | 883 | 65.76 | 3481-02-5 | 86870 |

# Single Injection Report

Agilent Technologies

| | |
|---|---|
| **Data file:** | D:\CDSProjects\GCMS Liliana\Results\Wenjun\WJ206-50ppm-25bar-IPA-3-INITIAL.sirslt\WJ206-50ppm-25bar-IPA-3-INITIAL.dx |
| **Sample name:** | WJ206-50ppm-25bar-IPA-3-INITIAL |
| **Description:** | |
| **Sample amount:** | 0.000 |
| **Instrument:** | GCMS |
| **Injection date:** | 2020-01-11 17:04:01+01:00 |
| **Acq. method:** | DB5MS 60-300_WY.amx |
| **Analysis method:** | Bob_FID+MS.pmx |
| **Last changed:** | 2019-08-30 14:05:20+02:00 |

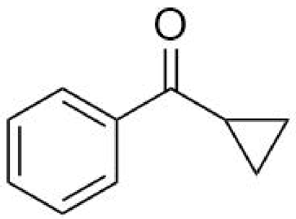

**8a**

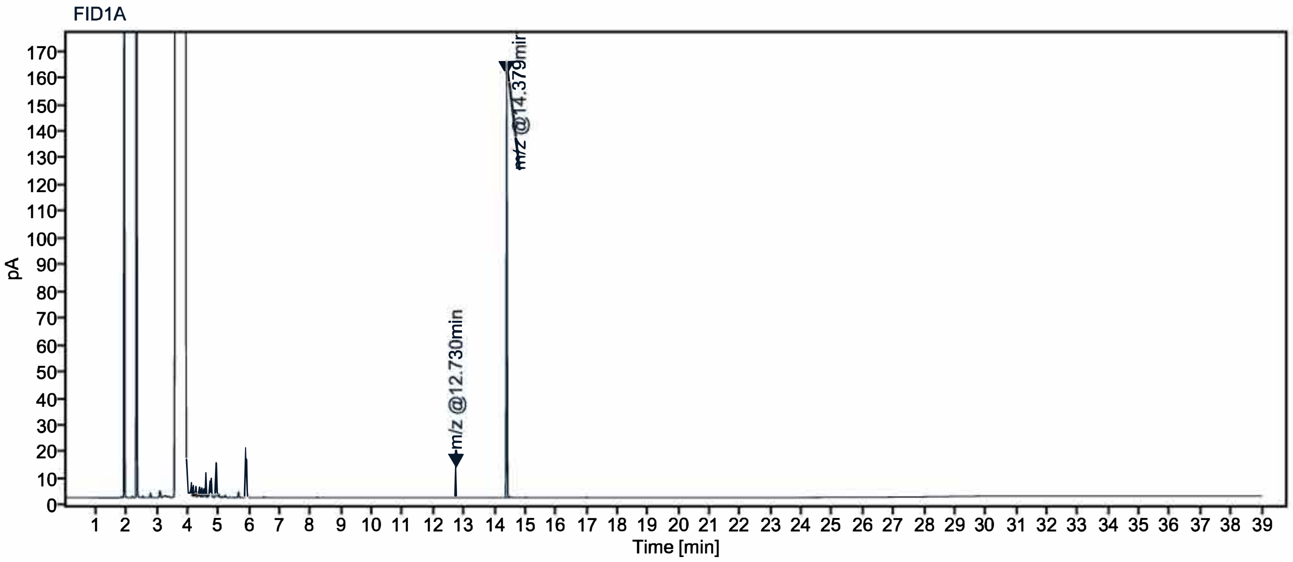


**Signal:** FID1A

| RT [min] | Area | Height |
|---|---|---|
| 12.730 | 15.5620 | 11.3704 |
| 14.379 | 242.8386 | 158.3949 |
| Sum | 258.4006 | |

**Signal:** MS1Front TIC SCAN EI

| RT [min] | Area | Height |
|---|---|---|
| 12.752 | 474439.3194 | 357905.2958 |
| 14.401 | 8998228.0413 | 6175450.7033 |
| Sum | 9472667.3607 | |

# Single Injection Report

Agilent Technologies

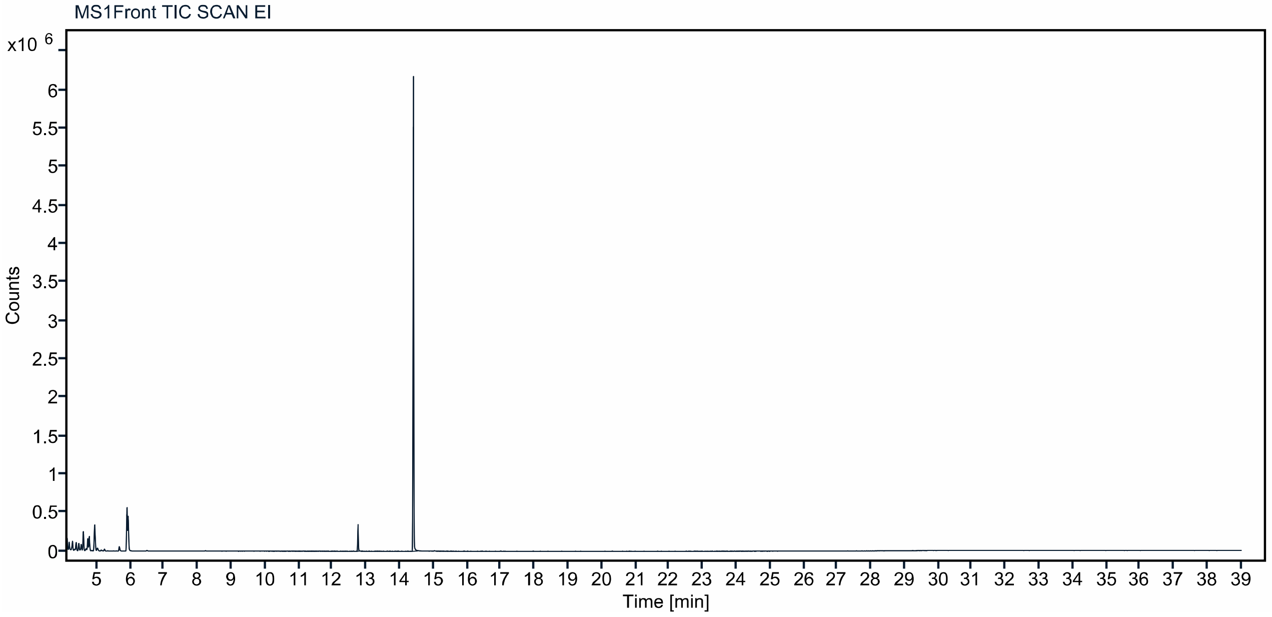


| RT | Compound Name | Score | Rev. Score | Prob. % | CAS # | Library Id |
|---|---|---|---|---|---|---|
| 12.752 | Dodecane | 913 | 920 | 20.38 | 112-40-3 | 26119 |

| RT | Compound Name | Score | Rev. Score | Prob. % | CAS # | Library Id |
|---|---|---|---|---|---|---|
| 14.401 | Cyclopropyl phenyl ketone | 947 | 947 | 88.32 | 3481-02-5 | 86870 |

# Single Injection Report

Agilent Technologies

**Data file:** D:\CDSProjects\GCMS Liliana\Results\Wenjun\WJ206-17-IPA-120-50PPM.sirslt\WJ206-17-IPA-120-50PPM.dx

**Sample name:** WJ206-17-IPA-120-50PPM

**Description:**

**Sample amount:** 0.000

**Instrument:** GCMS

**Injection date:** 2020-02-14 11:51:15+01:00

**Acq. method:** DB5MS 60-300_WY.amx

**Analysis method:** Bob_FID+MS.pmx

**Last changed:** 2019-08-30 14:05:20+02:00

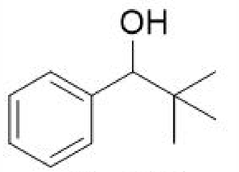


**9b**, 81%

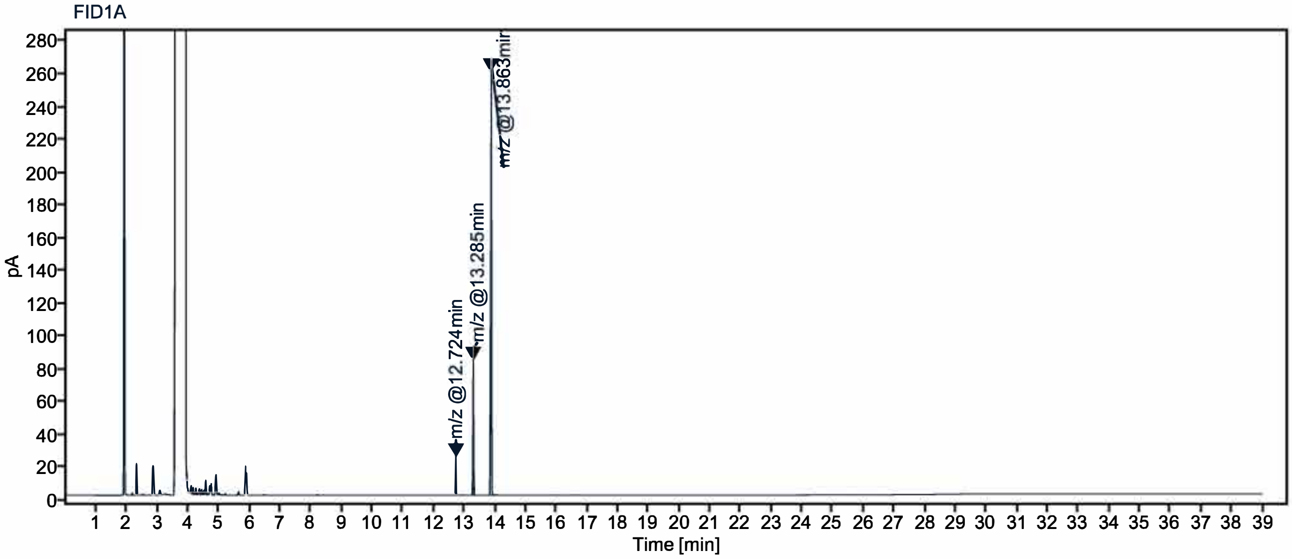


**Signal:** FID1A

| RT [min] | Area | Height |
|---|---|---|
| 12.724 | 31.8647 | 23.4921 |
| 13.285 | 116.3424 | 82.5125 |
| 13.863 | 415.1819 | 258.2947 |
| Sum | 563.3890 | |

**Signal:** MS1Front TIC SCAN EI

| RT [min] | Area | Height |
|---|---|---|
| 12.746 | 863983.3389 | 680734.4558 |
| 13.307 | 3695112.2620 | 2746469.7002 |
| 13.885 | 12981905.1278 | 8559718.9922 |
| Sum | 17541000.7286 | |

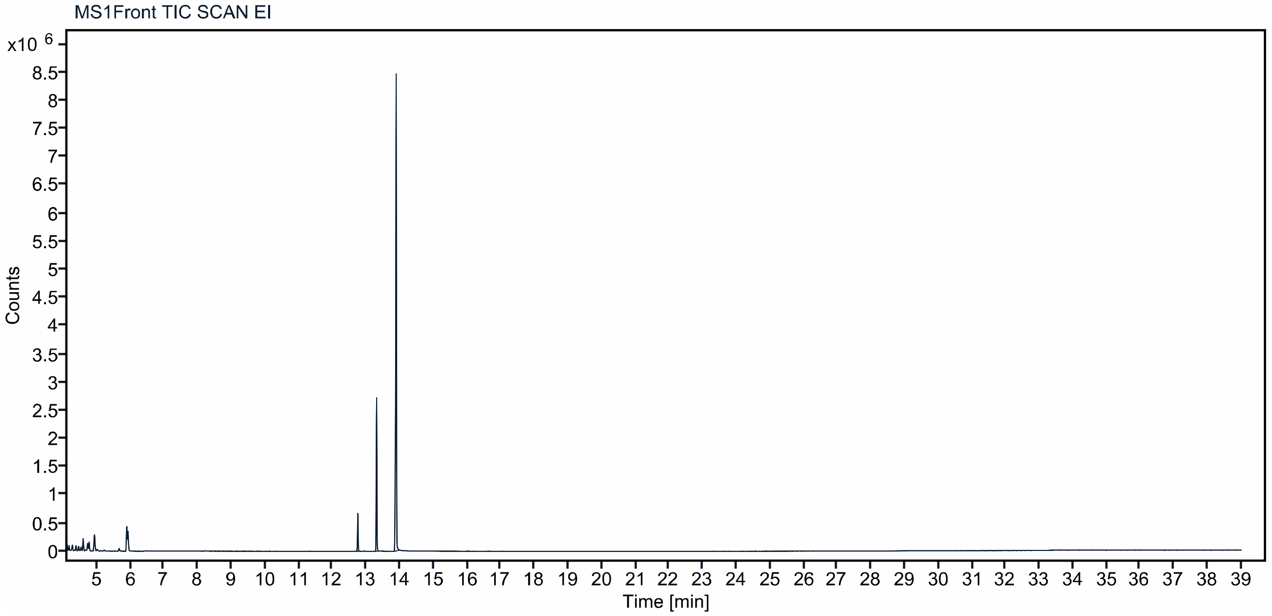


| RT | Compound Name | Score | Rev. Score | Prob. % | CAS # | Library Id |
|---|---|---|---|---|---|---|
| 12.746 | Dodecane | 929 | 931 | 22.45 | 112-40-3 | 26119 |

| RT | Compound Name | Score | Rev. Score | Prob. % | CAS # | Library Id |
|---|---|---|---|---|---|---|
| 13.307 | Phenyl tert-butyl ketone | 886 | 889 | 33.34 | 938-16-9 | 86963 |

| RT | Compound Name | Score | Rev. Score | Prob. % | CAS # | Library Id |
|---|---|---|---|---|---|---|
| 13.885 | 2,2-Dimethyl-1-phenyl-1-propanol | 900 | 913 | 46.19 | 3835-64-1 | 91414 |

# Single Injection Report

Agilent Technologies

**Data file:** D:\CDSProjects\GCMS Liliana\Results\Wenjun\WJ206-17-IPA-120-50PPM-initial.sirslt\WJ206-17-IPA-120-50PPM-initial.dx
**Sample name:** WJ206-17-IPA-120-50PPM-initial
**Description:**
**Sample amount:** 0.000

**Instrument:** GCMS
**Injection date:** 2020-02-14 12:35:02+01:00
**Acq. method:** DB5MS 60-300_WY.amx
**Analysis method:** Bob_FID+MS.pmx
**Last changed:** 2019-08-30 14:05:20+02:00

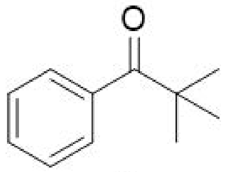

8b

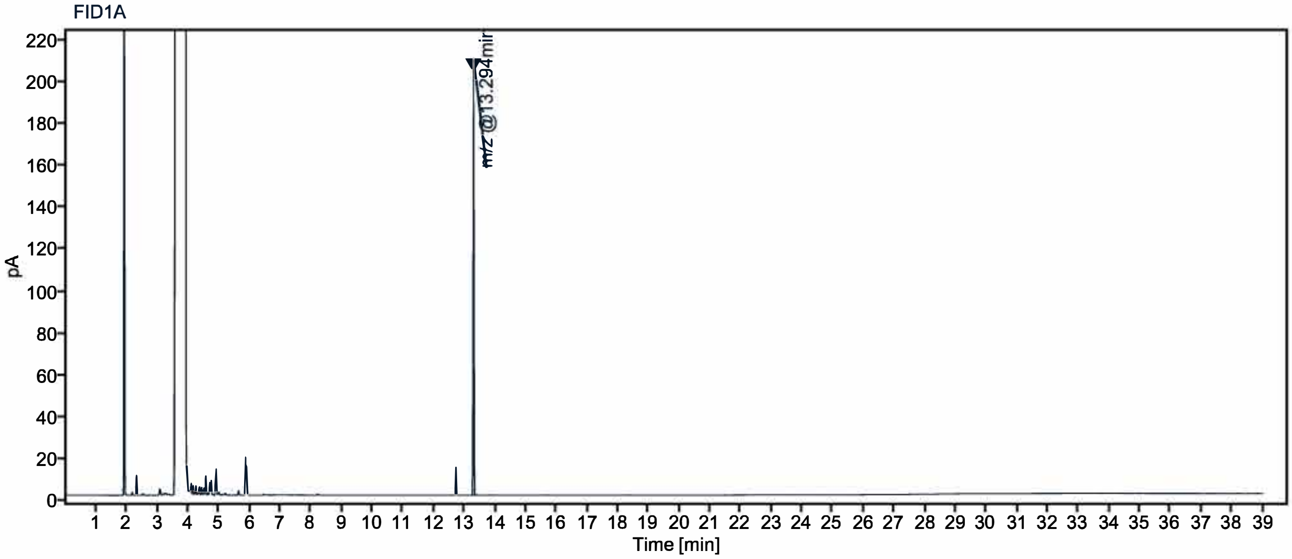


**Signal:** FID1A

| RT [min] | Area | Height |
|---|---|---|
| 13.294 | 297.8659 | 201.4497 |
| Sum | 297.8659 | |

**Signal:** MS1Front TIC SCAN EI

| RT [min] | Area | Height |
|---|---|---|
| 12.746 | 448868.6990 | 345291.9477 |
| 13.315 | 9972528.5901 | 6937548.5804 |
| Sum | 10421397.2891 | |

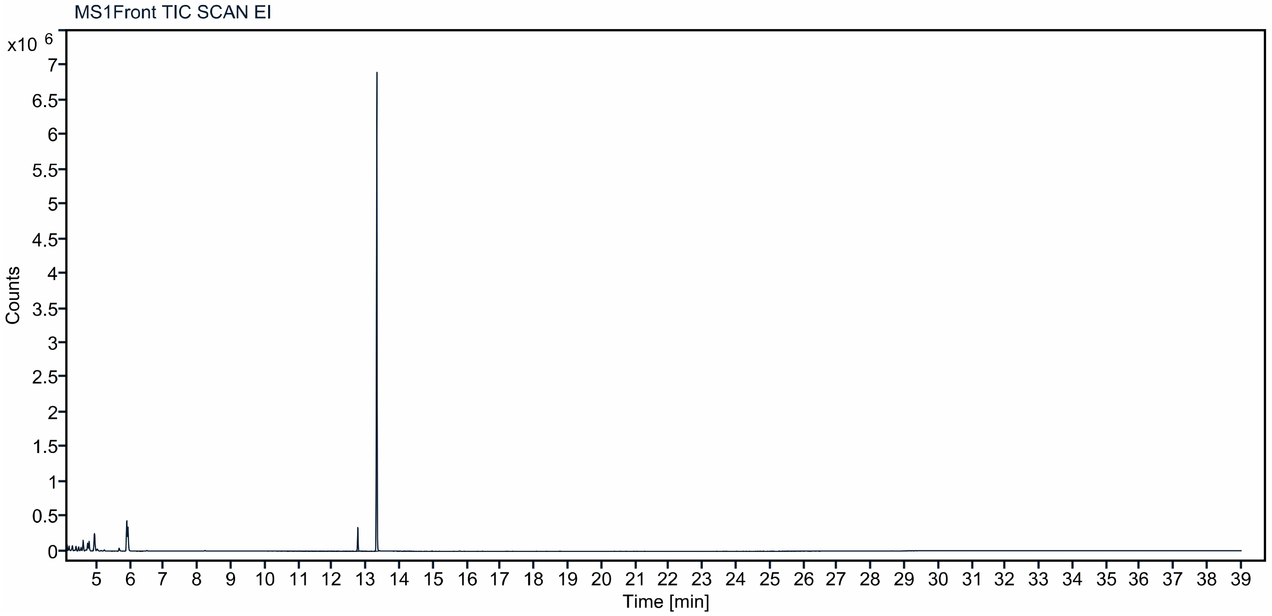


| RT | Compound Name | Score | Rev. Score | Prob. % | CAS # | Library Id |
|---|---|---|---|---|---|---|
| 12.746 | Dodecane | 908 | 915 | 18.11 | 112-40-3 | 26119 |

| RT | Compound Name | Score | Rev. Score | Prob. % | CAS # | Library Id |
|---|---|---|---|---|---|---|
| 13.315 | Phenyl tert-butyl ketone | 893 | 896 | 39.16 | 938-16-9 | 86963 |

# Single Injection Report

Agilent Technologies

**Data file:** D:\CDSProjects\GCMS Liliana\Results\Wenjun\WJ206-50ppm-25bar-diox-1.sirslt\WJ206-50ppm-25bar-diox-1.dx

**Sample name:** WJ206-50ppm-25bar-diox-1

**Description:**

**Sample amount:** 0.000

**Instrument:** GCMS

**Injection date:** 2020-01-11 13:25:09+01:00

**Acq. method:** DB5MS 60-300_WY.amx

**Analysis method:** Bob_FID+MS.pmx

**Last changed:** 2019-08-30 14:05:20+02:00

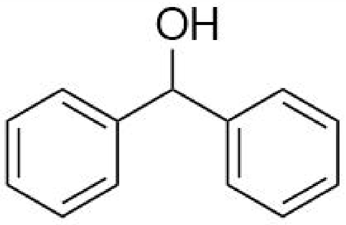

**9c**, 99%

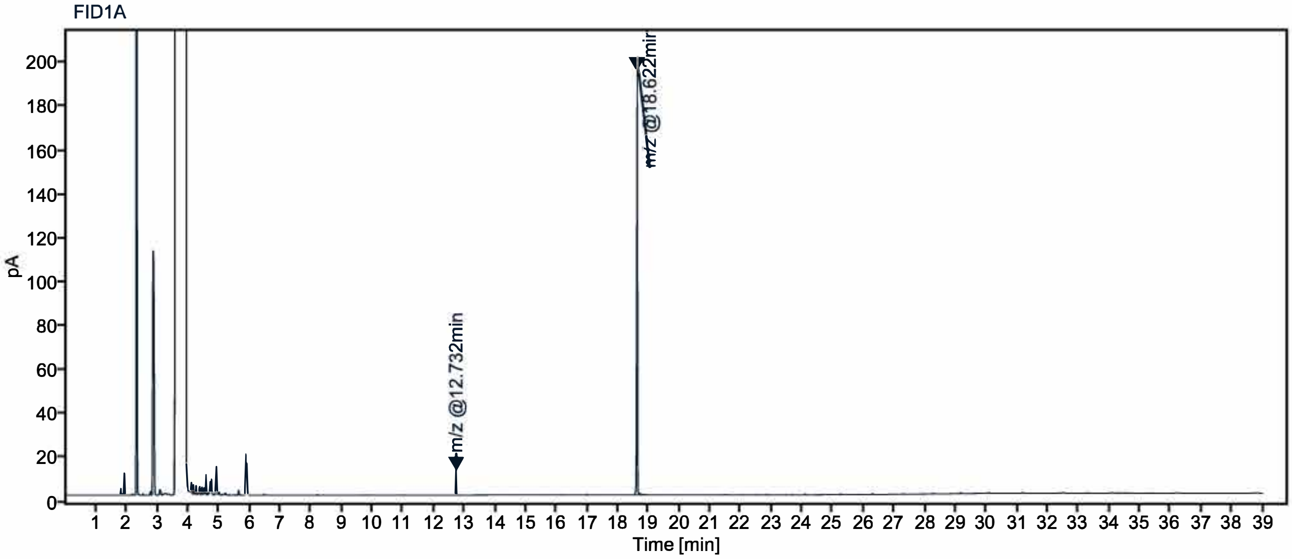


**Signal:** FID1A

| RT [min] | Area | Height |
|---|---|---|
| 12.732 | 15.4991 | 11.2409 |
| 18.622 | 322.4307 | 193.3878 |
| Sum | 337.9298 | |

**Signal:** MS1Front TIC SCAN EI

| RT [min] | Area | Height |
|---|---|---|
| 12.754 | 494448.6266 | 372051.1096 |
| 18.646 | 11234512.3003 | 7814415.2494 |
| Sum | 11728960.9270 | |

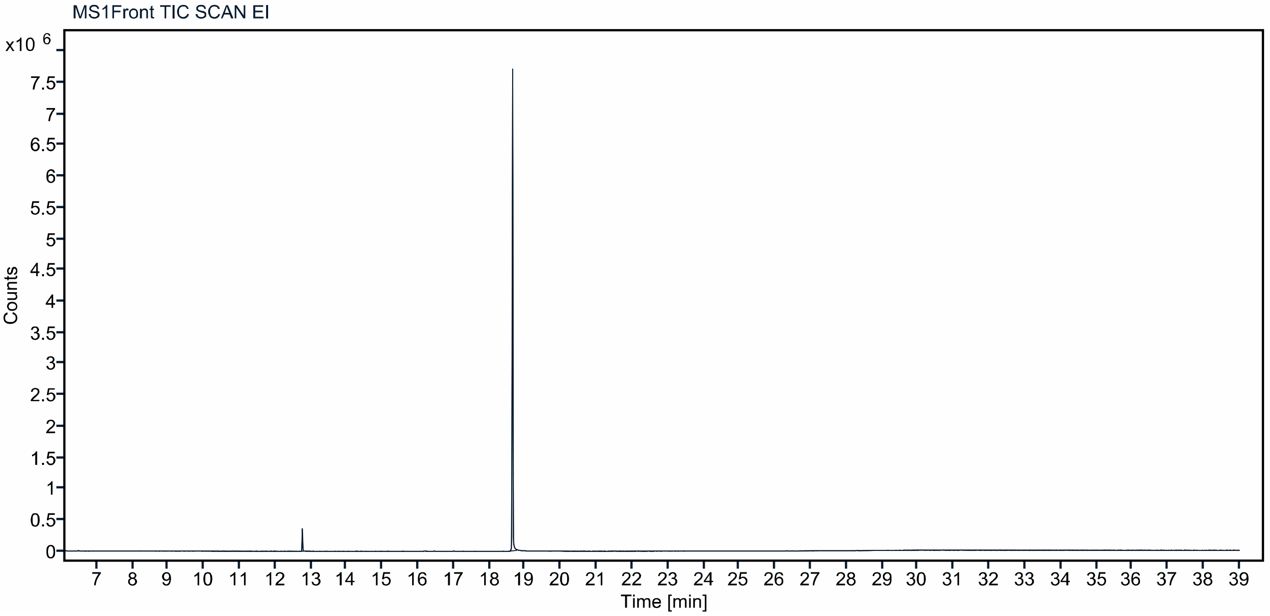


| RT | Compound Name | Score | Rev. Score | Prob. % | CAS # | Library Id |
|---|---|---|---|---|---|---|
| 12.754 | Dodecane | 930 | 937 | 25.32 | 112-40-3 | 26119 |

| RT | Compound Name | Score | Rev. Score | Prob. % | CAS # | Library Id |
|---|---|---|---|---|---|---|
| 18.646 | Benzenemethanol, α-phenyl- | 964 | 967 | 93.6 | 91-01-0 | 86338 |

# Single Injection Report

**Data file:** D:\CDSProjects\GCMS Liliana\Results\Wenjun\WJ206-50ppm-25bar-diox-1-initial.sirslt\WJ206-50ppm-25bar-diox-1-initial.dx

**Sample name:** WJ206-50ppm-25bar-diox-1-initial

**Description:**

**Sample amount:** 0.000

**Instrument:** GCMS

**Injection date:** 2020-01-11 14:08:52+01:00

**Acq. method:** DB5MS 60-300_WY.amx

**Analysis method:** Bob_FID+MS.pmx

**Last changed:** 2019-08-30 14:05:20+02:00

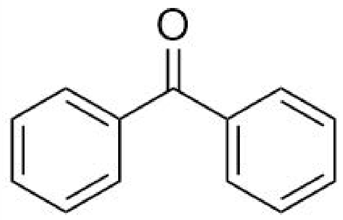


**8c**

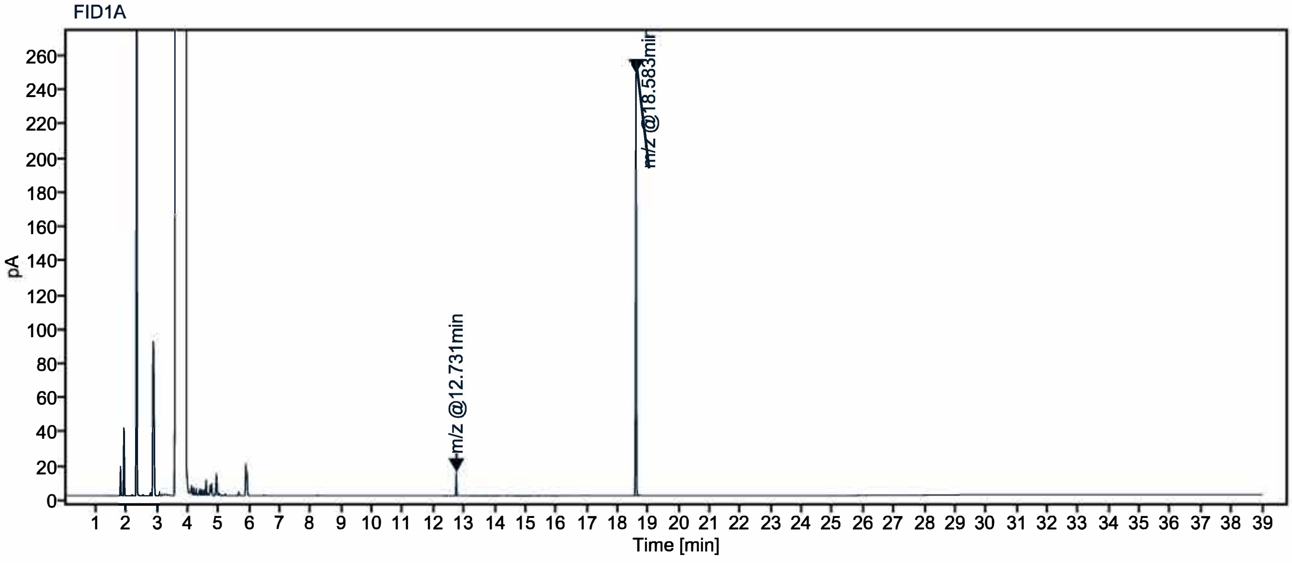


**Signal:** FID1A

| RT [min] | Area | Height |
|---|---|---|
| 12.731 | 19.4139 | 14.1466 |
| 18.583 | 400.5525 | 247.1659 |
| Sum | 419.9664 | |

**Signal:** MS1Front TIC SCAN EI

| RT [min] | Area | Height |
|---|---|---|
| 12.753 | 624137.7940 | 480038.7108 |
| 18.606 | 17196546.2100 | 11397919.7447 |
| Sum | 17820684.0039 | |

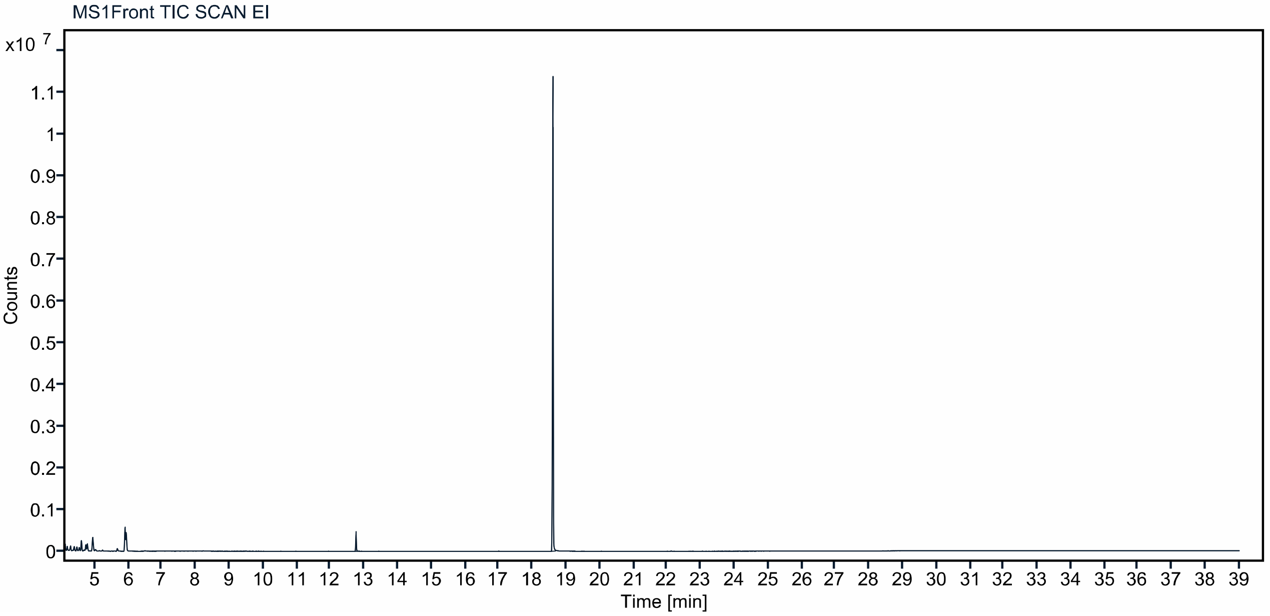


| RT | Compound Name | Score | Rev. Score | Prob. % | CAS # | Library Id |
|---|---|---|---|---|---|---|
| 12.753 | Dodecane | 929 | 933 | 21.93 | 112-40-3 | 26119 |

| RT | Compound Name | Score | Rev. Score | Prob. % | CAS # | Library Id |
|---|---|---|---|---|---|---|
| 18.606 | Benzophenone | 946 | 949 | 86.74 | 119-61-9 | 87049 |

# Single Injection Report

Agilent Technologies

| | |
|---|---|
| **Data file:** | D:\CDSProjects\GCMS Liliana\Results\Wenjun\WJ206-18-DIOX-50ppm-120oC.sirslt\WJ206-18-DIOX-50ppm-120oC.dx |
| **Sample name:** | WJ206-18-DIOX-50ppm-120oC |
| **Description:** | |
| **Sample amount:** | 0.000 |
| **Instrument:** | GCMS |
| **Injection date:** | 2020-02-12 00:09:16+01:00 |
| **Acq. method:** | DB5MS 60-300_WY.amx |
| **Analysis method:** | Bob_FID+MS.pmx |
| **Last changed:** | 2019-08-30 14:05:20+02:00 |

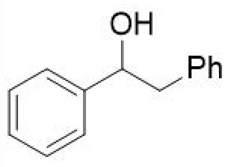


**9d**, >99%

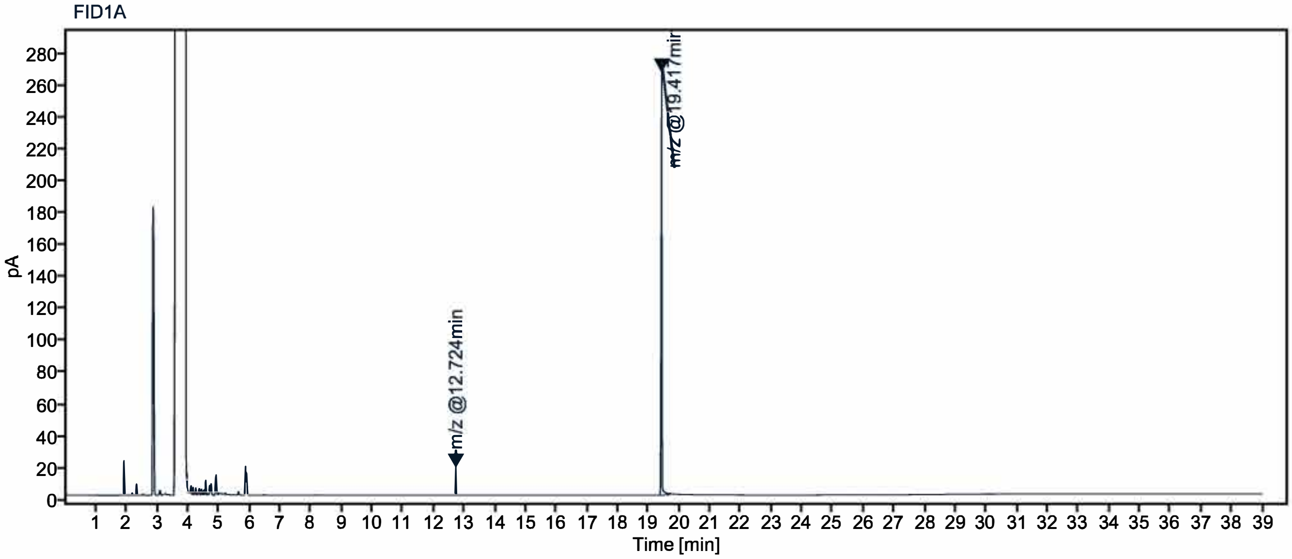


**Signal:** FID1A

| RT [min] | Area | Height |
|---|---|---|
| 12.724 | 24.1528 | 17.7297 |
| 19.417 | 497.7882 | 265.9659 |
| Sum | 521.9409 | |

**Signal:** MS1Front TIC SCAN EI

| RT [min] | Area | Height |
|---|---|---|
| 12.746 | 596028.1699 | 469456.3604 |
| 19.441 | 12304795.7512 | 7944709.4741 |
| Sum | 12900823.9212 | |

MS1Front TIC SCAN EI

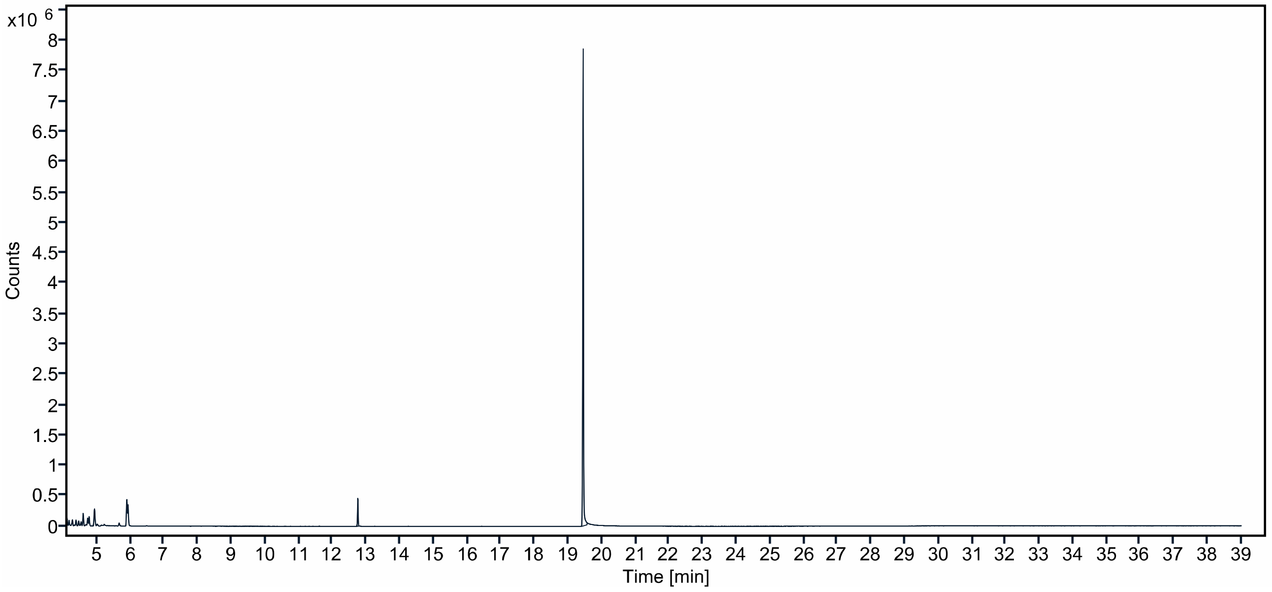


| RT | Compound Name | Score | Rev. Score | Prob. % | CAS # | Library Id |
|---|---|---|---|---|---|---|
| 12.746 | Dodecane | 925 | 929 | 21.79 | 112-40-3 | 26119 |

| RT | Compound Name | Score | Rev. Score | Prob. % | CAS # | Library Id |
|---|---|---|---|---|---|---|
| 19.441 | Benzeneethanol, α-phenyl- | 931 | 932 | 93.73 | 614-29-9 | 71932 |

# Single Injection Report

Agilent Technologies

**Data file:** D:\CDSProjects\GCMS Liliana\Results\Wenjun\WJ206-18-diox-50ppm-120oC-initial.sirslt\WJ206-18-diox-50ppm-120oC-initial.dx

**Sample name:** WJ206-18-diox-50ppm-120oC-initial

**Description:**

**Sample amount:** 0.000

**Instrument:** GCMS

**Injection date:** 2020-02-12 04:32:12+01:00

**Acq. method:** DB5MS 60-300_WY.amx

**Analysis method:** Bob_FID+MS.pmx

**Last changed:** 2019-08-30 14:05:20+02:00

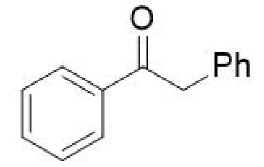


**8d**

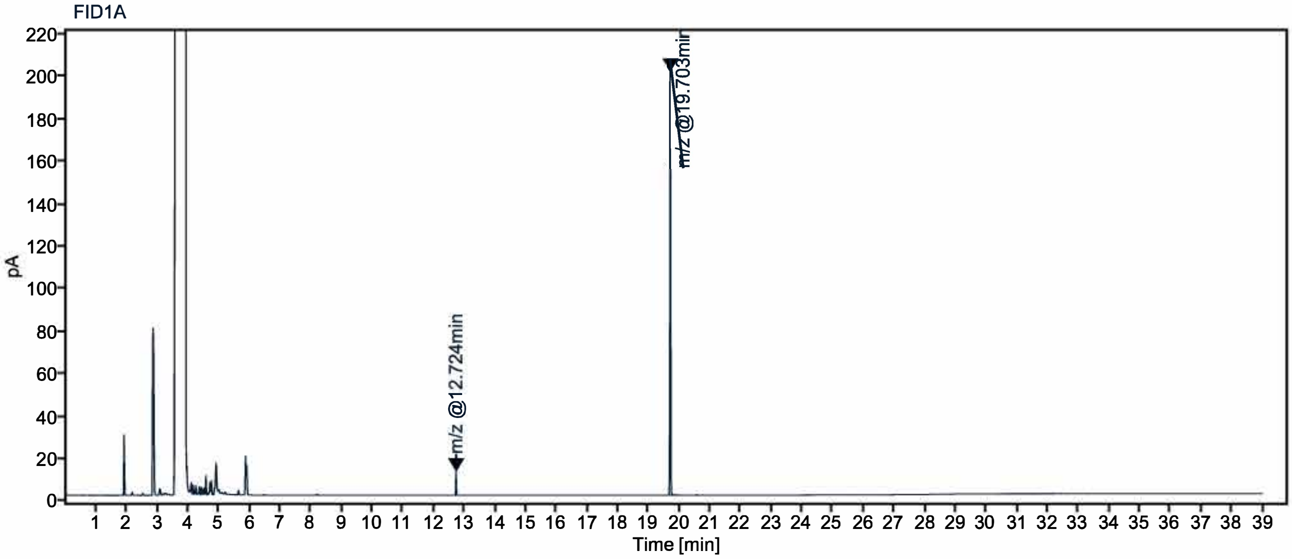


**Signal:** FID1A

| RT [min] | Area | Height |
|---|---|---|
| 12.724 | 15.2124 | 11.1817 |
| 19.703 | 336.1287 | 199.4435 |
| Sum | 351.3411 | |

**Signal:** MS1Front TIC SCAN EI

| RT [min] | Area | Height |
|---|---|---|
| 12.747 | 347611.0771 | 264562.8767 |
| 19.728 | 9317827.7797 | 6255534.2110 |
| Sum | 9665438.8568 | |

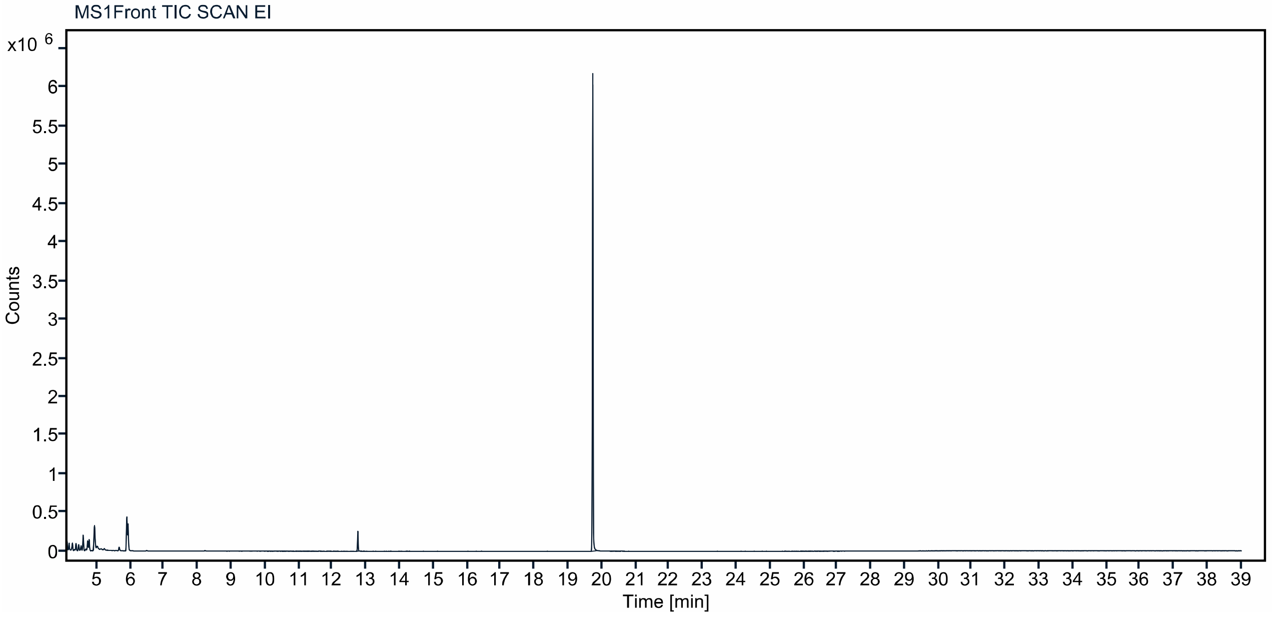


| RT | Compound Name | Score | Rev. Score | Prob. % | CAS # | Library Id |
|---|---|---|---|---|---|---|
| 12.747 | Dodecane | 892 | 898 | 16.92 | 112-40-3 | 26119 |

| RT | Compound Name | Score | Rev. Score | Prob. % | CAS # | Library Id |
|---|---|---|---|---|---|---|
| 19.728 | Ethanone, 1,2-diphenyl- | 938 | 942 | 44.75 | 451-40-1 | 86099 |

# Single Injection Report

Agilent Technologies

**Data file:** D:\CDSProjects\GCMS Liliana\Results\Wenjun\WJ206-27-diox-50PPM.sirslt\WJ206-27-diox-50PPM.dx

**Sample name:** WJ206-27-diox-50PPM

**Description:**

**Sample amount:** 0.000

**Instrument:** GCMS

**Injection date:** 2020-01-14 15:42:05+01:00

**Acq. method:** DB5MS 60-300_WY.amx

**Analysis method:** Bob_FID+MS.pmx

**Last changed:** 2019-08-30 14:05:20+02:00

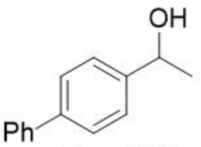


**9e**, >99%

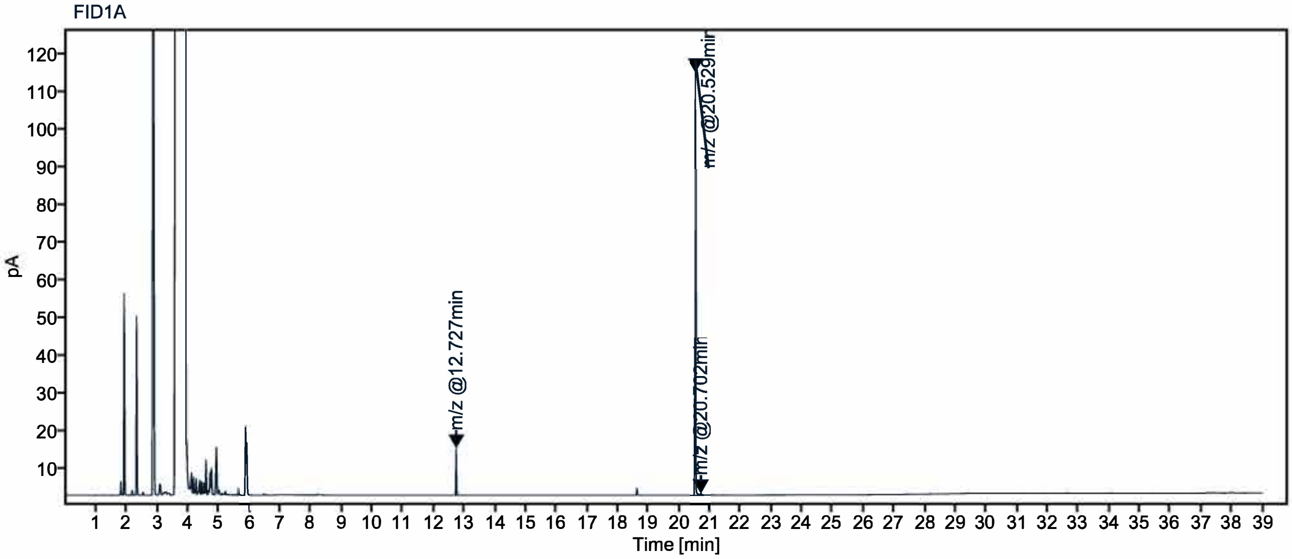


**Signal:** FID1A

| RT [min] | Area | Height |
|---|---|---|
| 12.727 | 17.2096 | 12.5482 |
| 20.529 | 176.3435 | 112.3824 |
| 20.702 | 1.6690 | 0.5811 |
| Sum | 195.2222 | |

**Signal:** MS1Front TIC SCAN EI

| RT [min] | Area | Height |
|---|---|---|
| 12.751 | 501292.2207 | 381692.1691 |
| 20.559 | 2983377.3992 | 2119390.6512 |
| Sum | 3484669.6199 | |

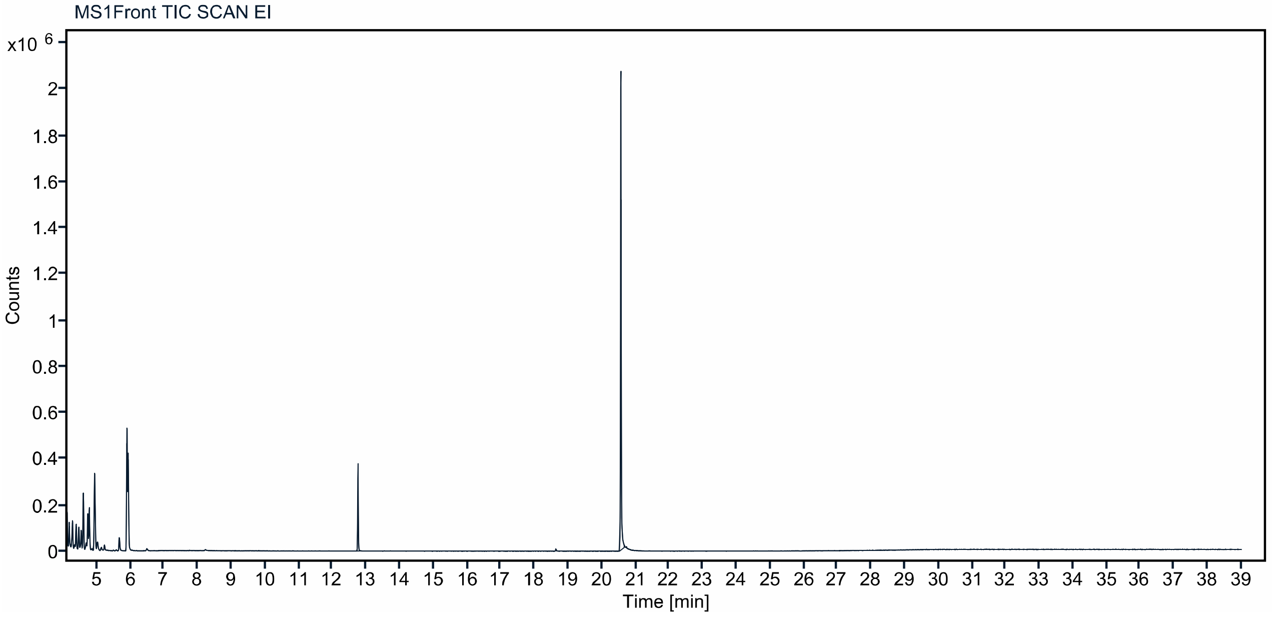


| RT | Compound Name | Score | Rev. Score | Prob. % | CAS # | Library Id |
|---|---|---|---|---|---|---|
| 12.751 | Dodecane | 938 | 940 | 25.82 | 112-40-3 | 26119 |

| RT | Compound Name | Score | Rev. Score | Prob. % | CAS # | Library Id |
|---|---|---|---|---|---|---|
| 20.559 | 4-Biphenyl methyl carbinol | 820 | 823 | 74.47 | 3562-73-0 | 159524 |

# Single Injection Report

Agilent Technologies

| | |
|---|---|
| **Data file:** | D:\CDSProjects\GCMS Liliana\Results\Wenjun\WJ206-27-diox-50PPM-initial.sirslt\WJ206-27-diox-50PPM-initial.dx |
| **Sample name:** | WJ206-27-diox-50PPM-initial |
| **Description:** | |
| **Sample amount:** | 0.000 |
| **Instrument:** | GCMS |
| **Injection date:** | 2020-01-14 20:05:10+01:00 |
| **Acq. method:** | DB5MS 60-300_WY.amx |
| **Analysis method:** | Bob_FID+MS.pmx |
| **Last changed:** | 2019-08-30 14:05:20+02:00 |

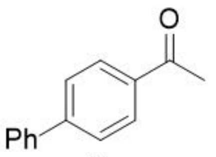


8e

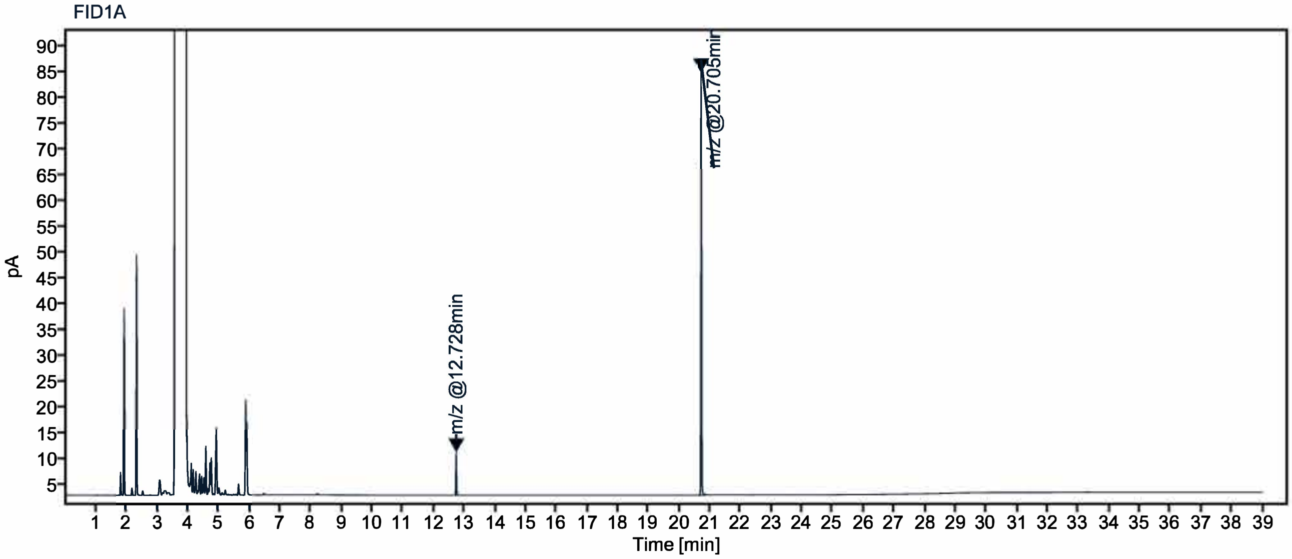


**Signal:** FID1A

| RT [min] | Area | Height |
|---|---|---|
| 12.728 | 11.8033 | 8.4473 |
| 20.705 | 126.6609 | 82.0413 |
| Sum | 138.4642 | |

**Signal:** MS1Front TIC SCAN EI

| RT [min] | Area | Height |
|---|---|---|
| 12.751 | 294355.1232 | 218914.2099 |
| 20.734 | 2698622.60 89 | 1925370.89 23 |
| Sum | 2992977.73 21 | |

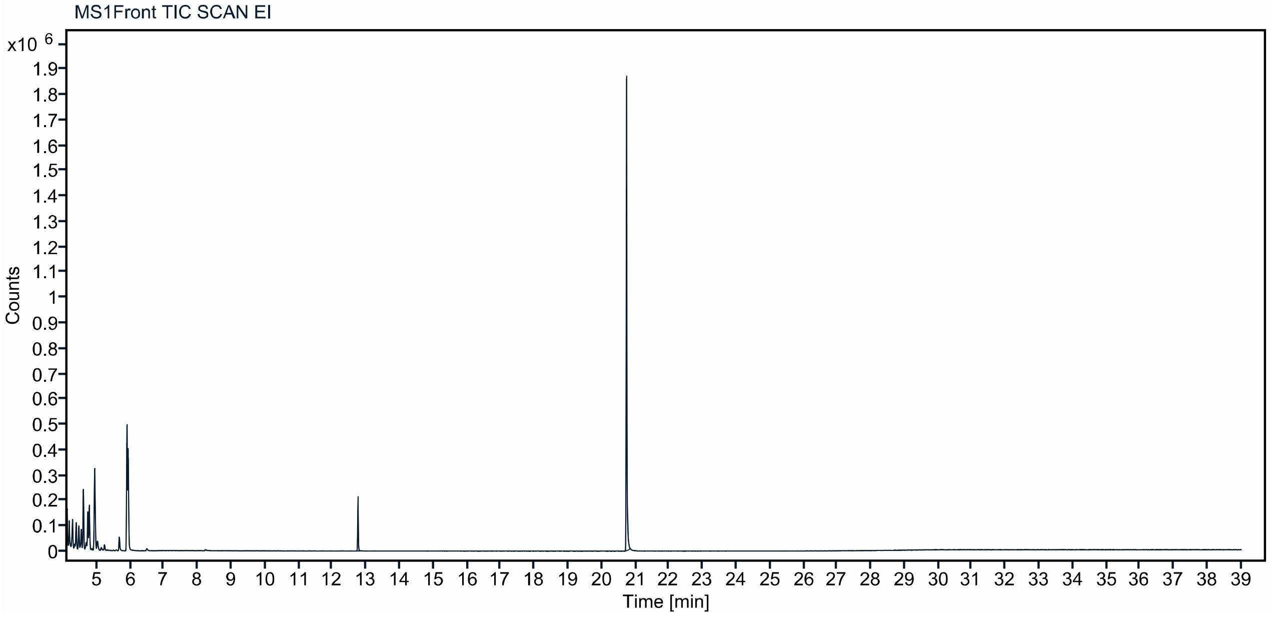


| RT | Compound Name | Score | Rev. Score | Prob. % | CAS # | Library Id |
|---|---|---|---|---|---|---|
| 12.751 | Dodecane | 892 | 898 | 14.46 | 112-40-3 | 26119 |

| RT | Compound Name | Score | Rev. Score | Prob. % | CAS # | Library Id |
|---|---|---|---|---|---|---|
| 20.734 | Ethanone, 1-[1,1'-biphenyl]-4-yl- | 945 | 948 | 70.51 | 92-91-1 | 184955 |

# Single Injection Report

Agilent Technologies

**Data file:** D:\CDSProjects\GCMS Liliana\Results\Wenjun\WJ206-20-diox-50PPM.sirslt\WJ206-20-diox-50PPM.dx

**Sample name:** WJ206-20-diox-50PPM

**Description:**

**Sample amount:** 0.000

**Instrument:** GCMS

**Injection date:** 2020-01-14 13:30:41+01:00

**Acq. method:** DB5MS 60-300_WY.amx

**Analysis method:** Bob_FID+MS.pmx

**Last changed:** 2019-08-30 14:05:20+02:00

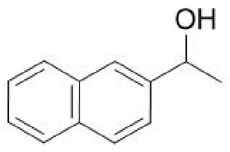


**9f**, 99%

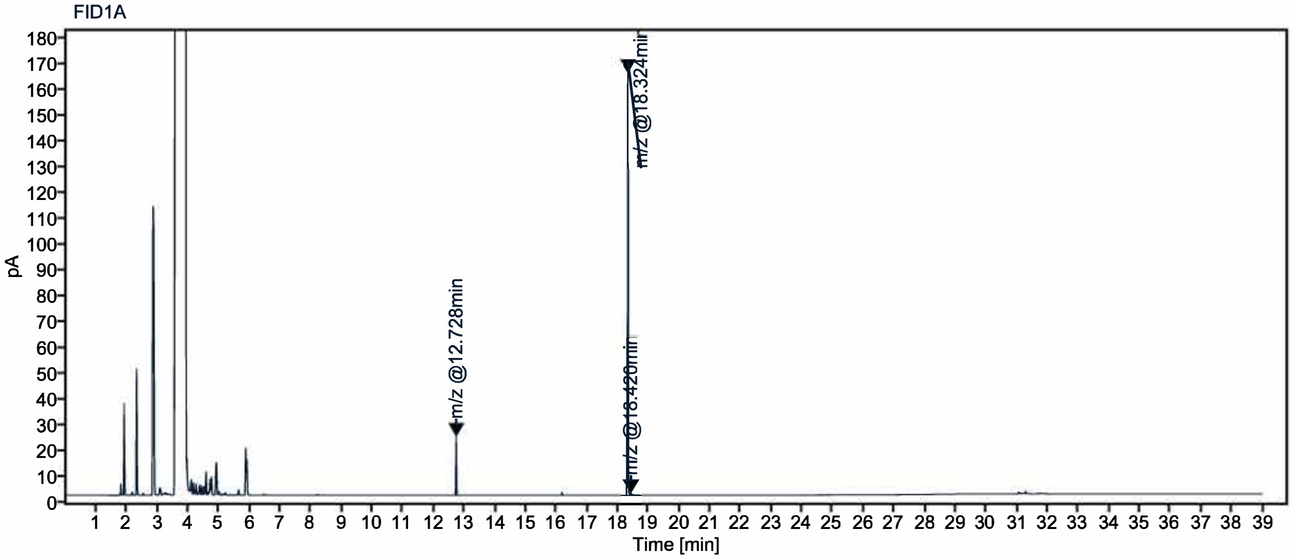


**Signal:** FID1A

| RT [min] | Area | Height |
|---|---|---|
| 12.728 | 31.0473 | 22.9242 |
| 18.324 | 270.4531 | 164.0689 |
| 18.420 | 3.5192 | 0.9038 |
| Sum | 305.0196 | |

**Signal:** MS1Front TIC SCAN EI

| RT [min] | Area | Height |
|---|---|---|
| 12.751 | 1010529.9640 | 790286.5735 |
| 18.350 | 8029519.6661 | 5684421.5044 |
| 18.443 | 197335.5722 | 52686.4090 |
| Sum | 9237385.2023 | |

# Single Injection Report

Agilent Technologies

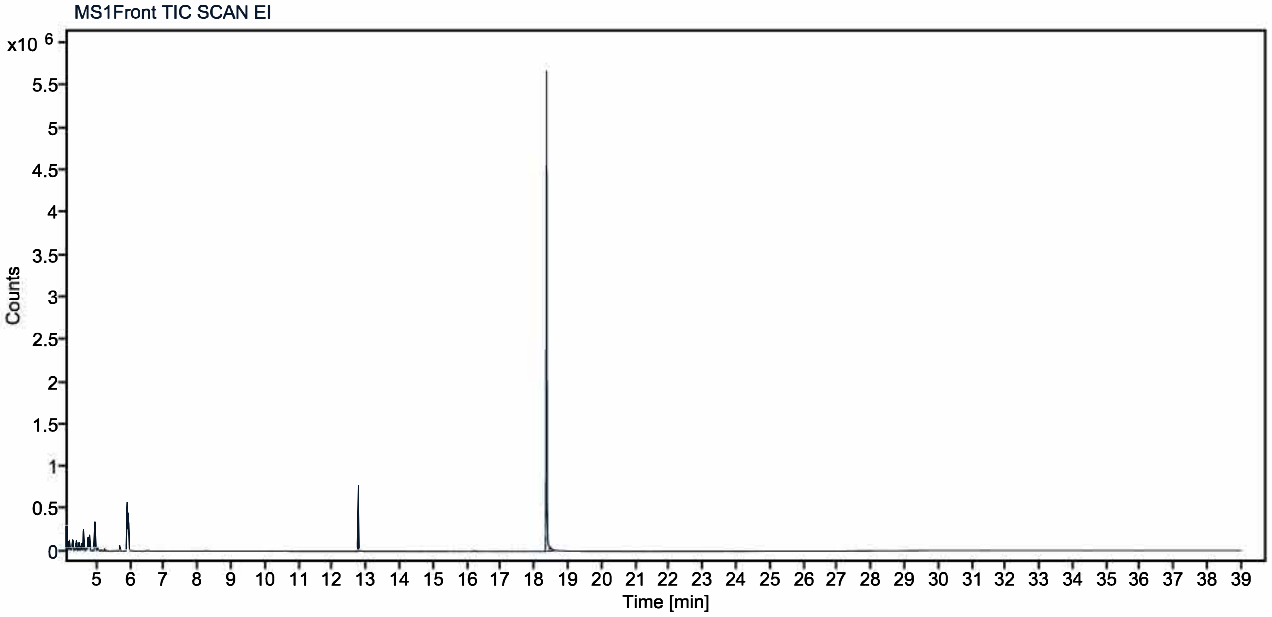


| RT | Compound Name | Score | Rev. Score | Prob. % | CAS # | Library Id |
|---|---|---|---|---|---|---|
| 12.751 | Dodecane | 947 | 949 | 25.74 | 112-40-3 | 26119 |

| RT | Compound Name | Score | Rev. Score | Prob. % | CAS # | Library Id |
|---|---|---|---|---|---|---|
| 18.35 | α-Methyl-2-naphthalenemethanol | 907 | 920 | 88.46 | 7228-47-9 | 124280 |

# Single Injection Report

Agilent Technologies

**Data file:** D:\CDSProjects\GCMS Liliana\Results\Wenjun\WJ206-20-diox-50PPM-initial-2.sirslt\WJ206-20-diox-50PPM-initial-2.dx

**Sample name:** WJ206-20-diox-50PPM-initial-2

**Description:**

**Sample amount:** 0.000

**Instrument:** GCMS

**Injection date:** 2020-01-14 17:53:37+01:00

**Acq. method:** DB5MS 60-300_WY.amx

**Analysis method:** Bob_FID+MS.pmx

**Last changed:** 2019-08-30 14:05:20+02:00

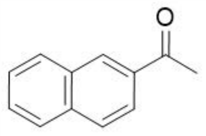

8f

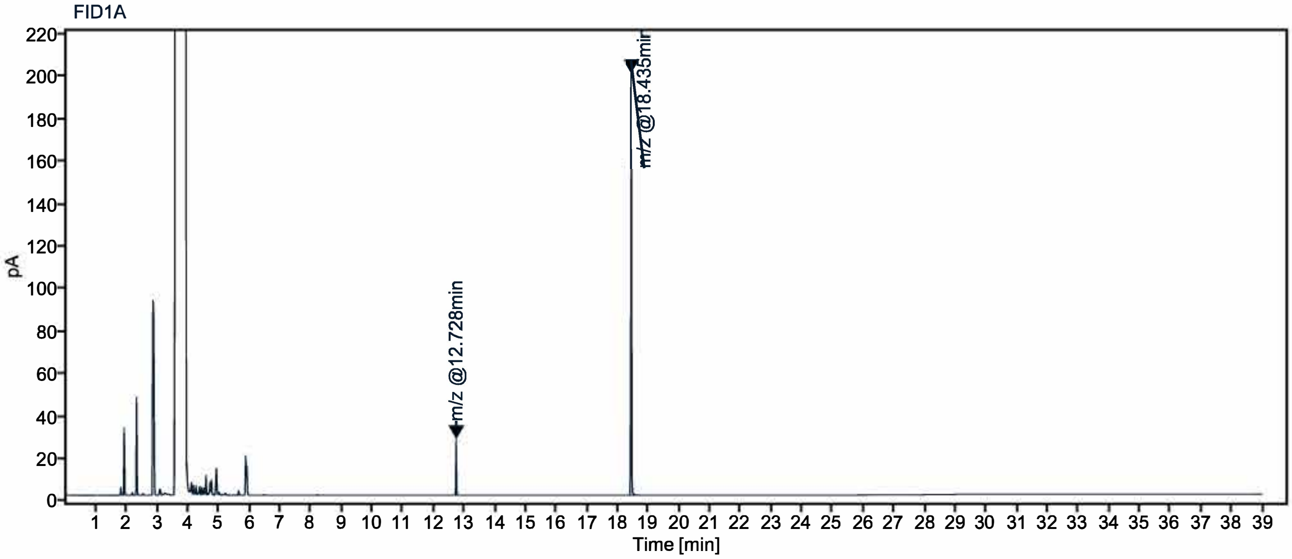


**Signal:** FID1A

| RT [min] | Area | Height |
|---|---|---|
| 12.728 | 36.6827 | 26.7361 |
| 18.435 | 340.4053 | 199.0208 |
| Sum | 377.0880 | |

**Signal:** MS1Front TIC SCAN EI

| RT [min] | Area | Height |
|---|---|---|
| 12.751 | 1139456.8510 | 885481.6753 |
| 18.460 | 11683854.8044 | 7768979.2723 |
| Sum | 12823311.6554 | |

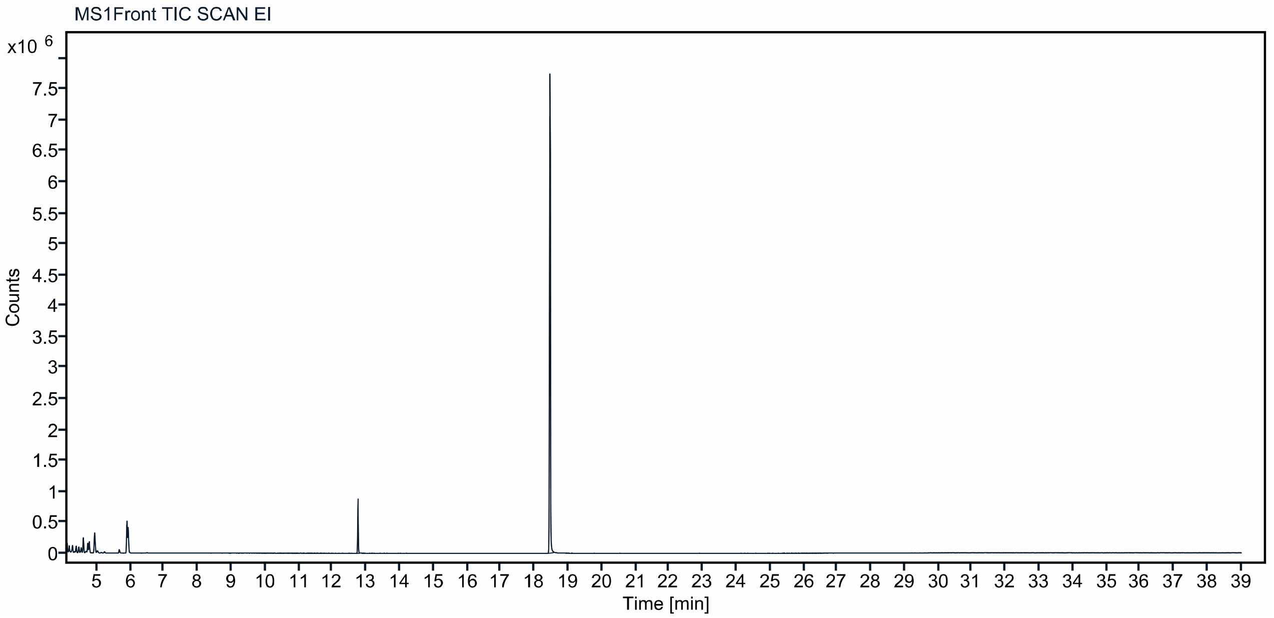


| RT | Compound Name | Score | Rev. Score | Prob. % | CAS # | Library Id |
|---|---|---|---|---|---|---|
| 12.751 | Dodecane | 933 | 935 | 22.54 | 112-40-3 | 26119 |

| RT | Compound Name | Score | Rev. Score | Prob. % | CAS # | Library Id |
|---|---|---|---|---|---|---|
| 18.46 | 2-Naphthyl methyl ketone | 953 | 959 | 76.67 | 93-08-3 | 122152 |

# Single Injection Report

Agilent Technologies

**Data file:** D:\CDSProjects\GCMS Liliana\Results\Wenjun\WJ206-26-ipa-50ppm-120oC.sirslt\WJ206-26-ipa-50ppm-120oC.dx
**Sample name:** WJ206-26-ipa-50ppm-120oC
**Description:**
**Sample amount:** 0.000

**Instrument:** GCMS
**Injection date:** 2020-02-12 02:20:39+01:00
**Acq. method:** DB5MS 60-300_WY.amx
**Analysis method:** Bob_FID+MS.pmx
**Last changed:** 2019-08-30 14:05:20+02:00

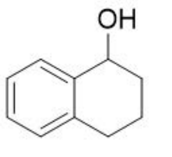


**9h**, 97%

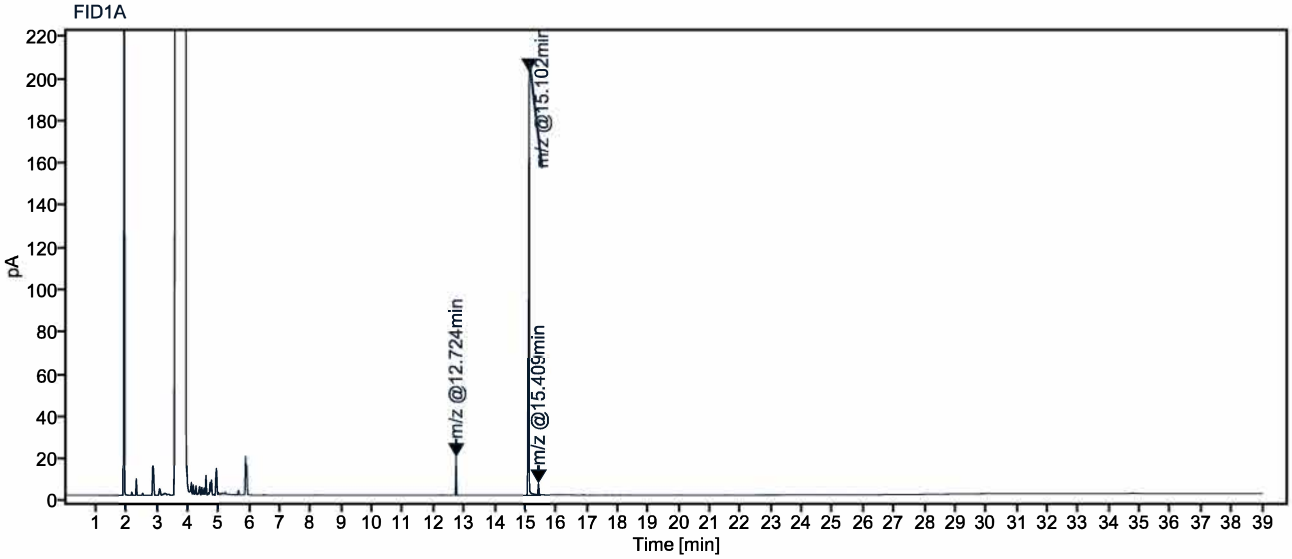


**Signal:** FID1A

| RT [min] | Area | Height |
|---|---|---|
| 12.724 | 24.9248 | 18.3547 |
| 15.102 | 361.4992 | 200.4867 |
| 15.409 | 9.1680 | 5.7752 |
| Sum | 395.5920 | |

**Signal:** MS1Front TIC SCAN EI

| RT [min] | Area | Height |
|---|---|---|
| 12.747 | 634969.5012 | 494130.3232 |
| 15.125 | 10989744.2760 | 6646741.8747 |
| 15.435 | 301356.5777 | 121565.7836 |
| Sum | 11926070.3550 | |

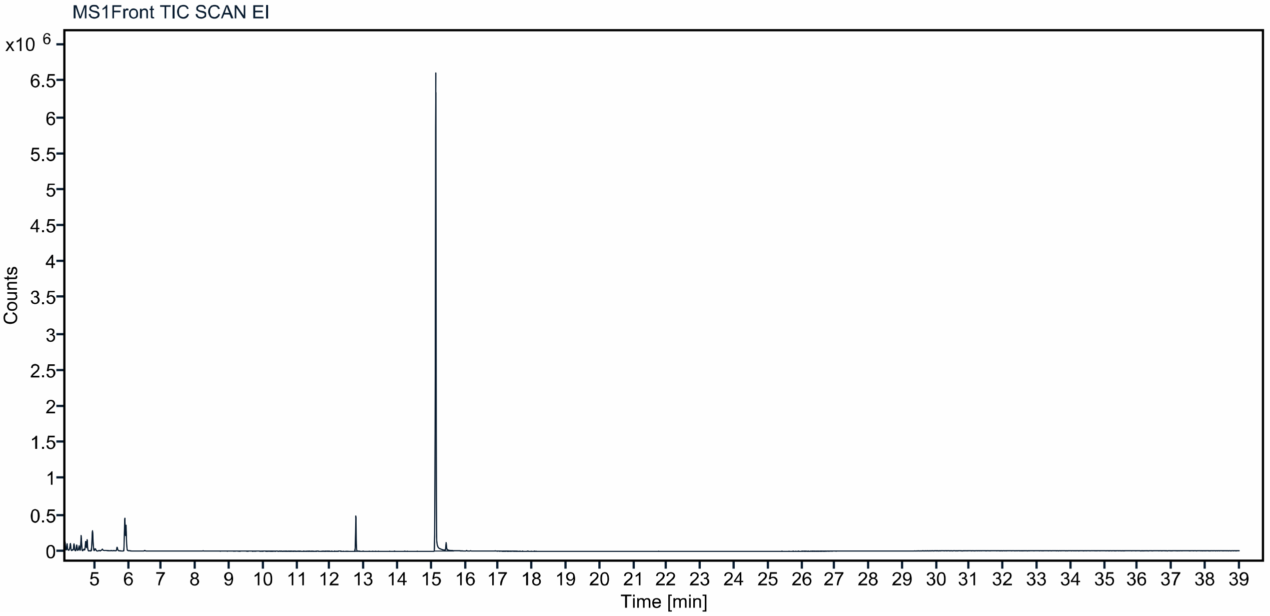


| RT | Compound Name | Score | Rev. Score | Prob. % | CAS # | Library Id |
|---|---|---|---|---|---|---|
| 12.747 | Dodecane | 918 | 924 | 20.88 | 112-40-3 | 26119 |

| RT | Compound Name | Score | Rev. Score | Prob. % | CAS # | Library Id |
|---|---|---|---|---|---|---|
| 15.125 | 1-Naphthalenol, 1,2,3,4-tetrahydro- | 930 | 932 | 93.36 | 529-33-9 | 125678 |

| RT | Compound Name | Score | Rev. Score | Prob. % | CAS # | Library Id |
|---|---|---|---|---|---|---|
| 15.435 | 1(2H)-Naphthalenone, 3,4-dihydro- | 898 | 901 | 79.46 | 529-34-0 | 106769 |

# Single Injection Report

**Data file:** D:\CDSProjects\GCMS Liliana\Results\Wenjun\WJ206-26-ipa-50ppm-120oC-initial.sirslt\WJ206-26-ipa-50ppm-120oC-initial.dx
**Sample name:** WJ206-26-ipa-50ppm-120oC-initial
**Description:**
**Sample amount:** 0.000

**Instrument:** GCMS
**Injection date:** 2020-02-12 06:43:45+01:00
**Acq. method:** DB5MS 60-300_WY.amx
**Analysis method:** Bob_FID+MS.pmx
**Last changed:** 2019-08-30 14:05:20+02:00

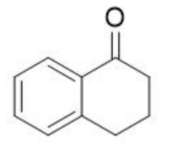

8h

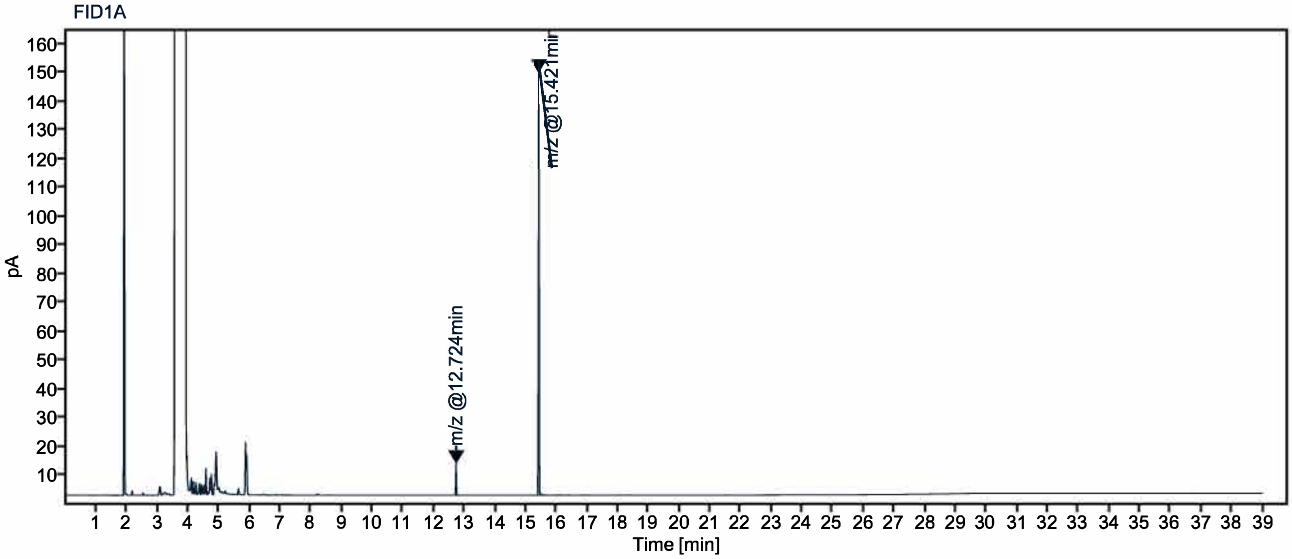


**Signal:** FID1A

| RT [min] | Area | Height |
|---|---|---|
| 12.724 | 14.9957 | 11.0536 |
| 15.421 | 228.7642 | 146.9339 |
| Sum | 243.7600 | |

**Signal:** MS1Front TIC SCAN EI

| RT [min] | Area | Height |
|---|---|---|
| 12.747 | 336658.9791 | 255266.8644 |
| 15.444 | 6416468.7113 | 4519816.2301 |
| Sum | 6753127.6904 | |

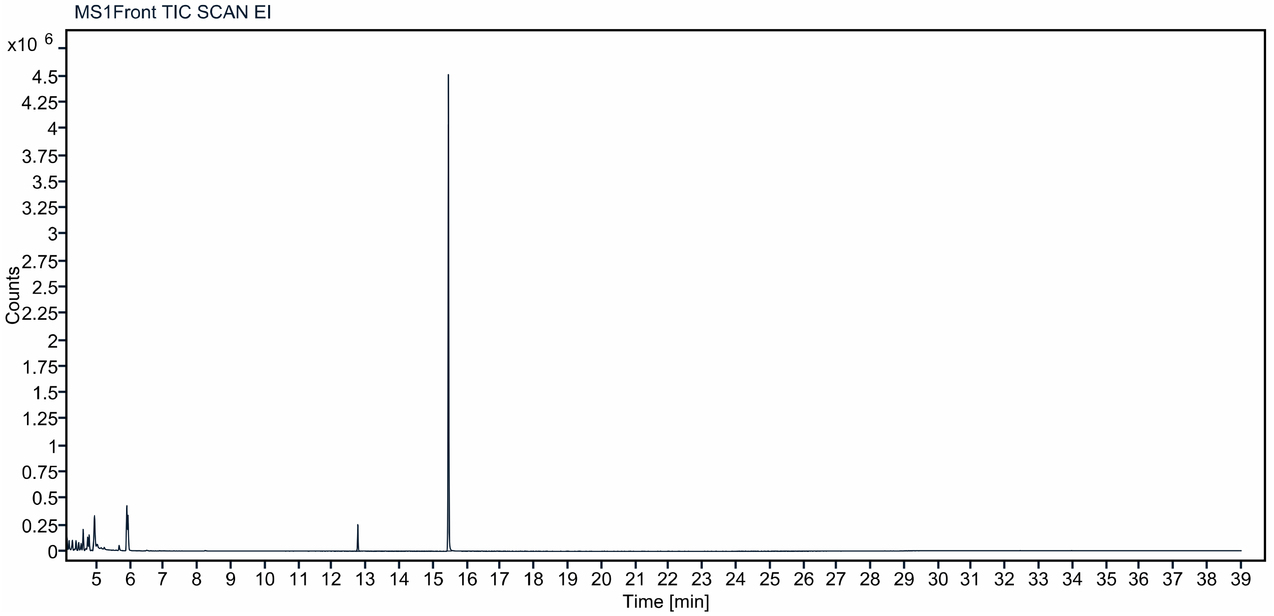


| RT | Compound Name | Score | Rev. Score | Prob. % | CAS # | Library Id |
|---|---|---|---|---|---|---|
| 12.747 | Dodecane | 893 | 898 | 16.95 | 112-40-3 | 26119 |

| RT | Compound Name | Score | Rev. Score | Prob. % | CAS # | Library Id |
|---|---|---|---|---|---|---|
| 15.444 | 1(2H)-Naphthalenone, 3,4-dihydro- | 945 | 946 | 91.01 | 529-34-0 | 106769 |

# Single Injection Report

Agilent Technologies

| | |
|---|---|
| **Data file:** | D:\CDSProjects\GCMS Liliana\Results\Wenjun\WJ206-23-DIOX-50ppm-120oC.sirslt\WJ206-23-DIOX-50ppm-120oC.dx |
| **Sample name:** | WJ206-23-DIOX-50ppm-120oC |
| **Description:** | |
| **Sample amount:** | 0.000 |

| | |
|---|---|
| **Instrument:** | GCMS |
| **Injection date:** | 2020-02-12 01:36:52+01:00 |
| **Acq. method:** | DB5MS 60-300_WY.amx |
| **Analysis method:** | Bob_FID+MS.pmx |
| **Last changed:** | 2019-08-30 14:05:20+02:00 |

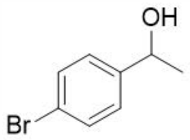


**9j**, >99%

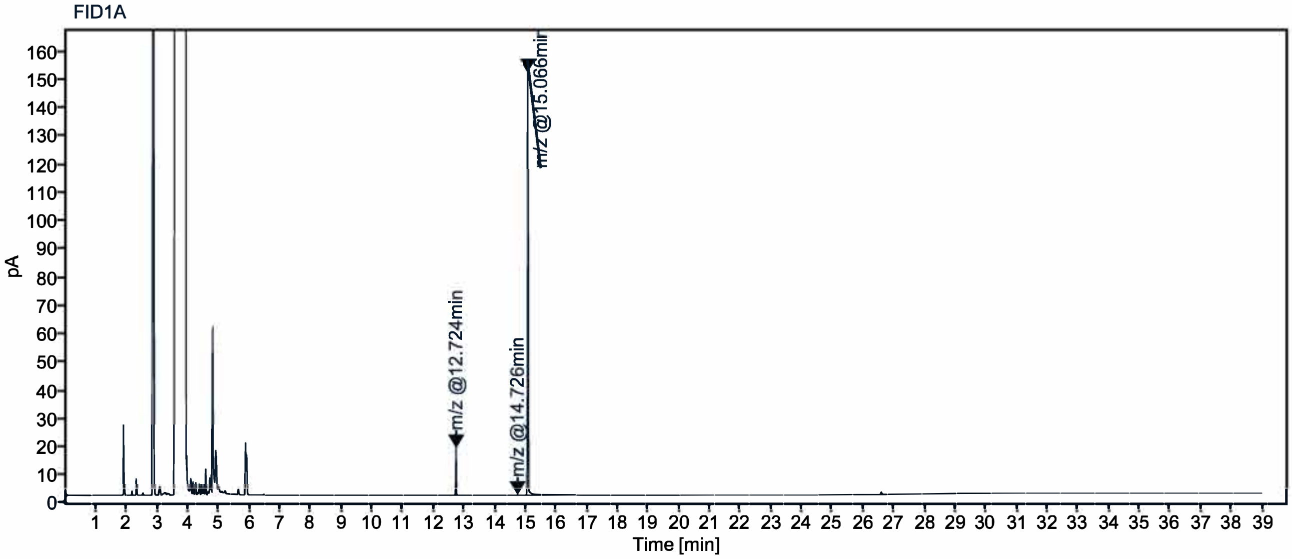


**Signal:** FID1A

| RT [min] | Area | Height |
|---|---|---|
| 12.724 | 22.9501 | 16.7714 |
| 14.726 | 0.3330 | 0.1656 |
| 15.066 | 262.8731 | 149.6982 |
| Sum | 286.1563 | |

**Signal:** MS1Front TIC SCAN EI

| RT [min] | Area | Height |
|---|---|---|
| 12.747 | 578838.9240 | 447948.8022 |
| 15.090 | 8439071.3779 | 5663906.2153 |
| Sum | 9017910.3019 | |

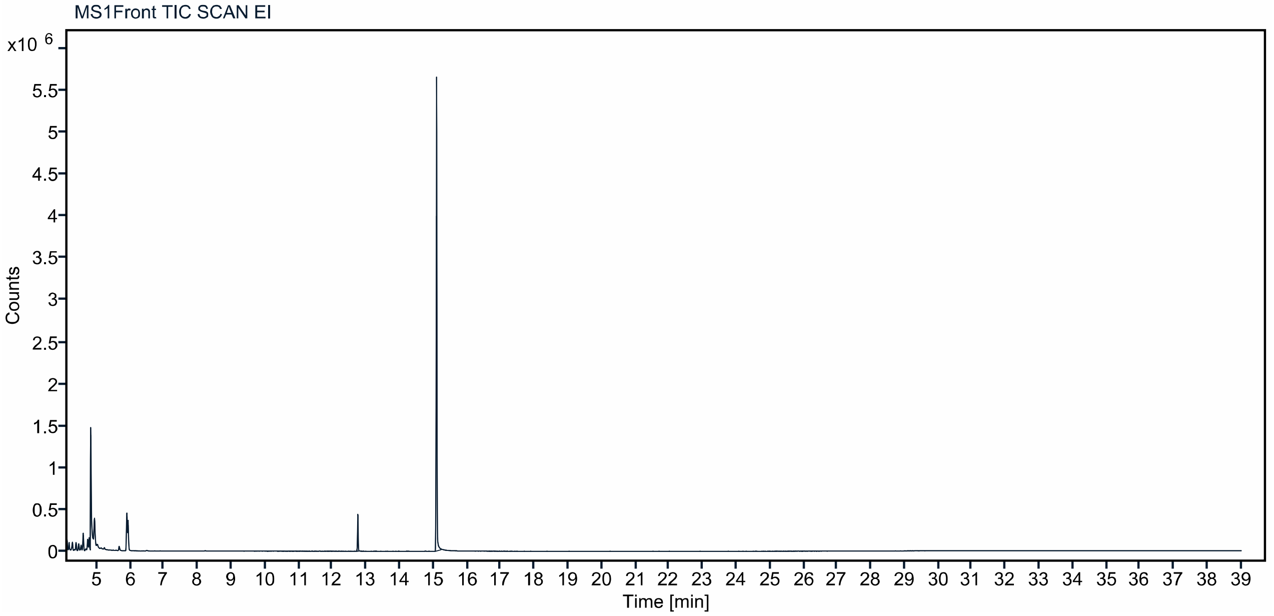


| RT | Compound Name | Score | Rev. Score | Prob. % | CAS # | Library Id |
|---|---|---|---|---|---|---|
| 12.747 | Dodecane | 923 | 927 | 20.08 | 112-40-3 | 26119 |

| RT | Compound Name | Score | Rev. Score | Prob. % | CAS # | Library Id |
|---|---|---|---|---|---|---|
| 15.09 | Benzenemethanol, 4-bromo-α-methyl- | 934 | 940 | 61.26 | 5391-88-8 | 51931 |

# Single Injection Report

Agilent Technologies

| | |
|---|---|
| **Data file:** | D:\CDSProjects\GCMS Liliana\Results\Wenjun\WJ206-23-diox-50ppm-120oC-initial.sirslt\WJ206-23-diox-50ppm-120oC-initial.dx |
| **Sample name:** | WJ206-23-diox-50ppm-120oC-initial |
| **Description:** | |
| **Sample amount:** | 0.000 |
| **Instrument:** | GCMS |
| **Injection date:** | 2020-02-12 05:59:56+01:00 |
| **Acq. method:** | DB5MS 60-300_WY.amx |
| **Analysis method:** | Bob_FID+MS.pmx |
| **Last changed:** | 2019-08-30 14:05:20+02:00 |

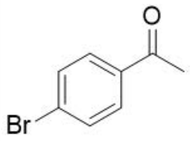


**8j**

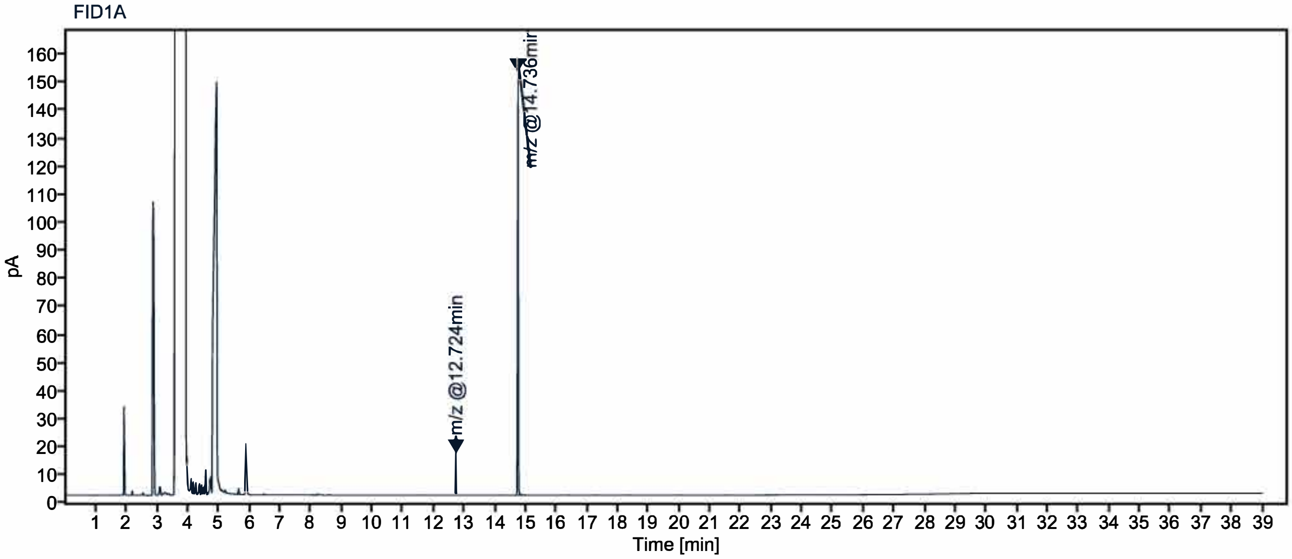


**Signal:** FID1A

| RT [min] | Area | Height |
|---|---|---|
| 12.724 | 20.7375 | 15.0200 |
| 14.736 | 248.8597 | 150.8484 |
| Sum | 269.5972 | |

**Signal:** MS1Front TIC SCAN EI

| RT [min] | Area | Height |
|---|---|---|
| 12.747 | 495990.9249 | 378426.1644 |
| 14.760 | 9473482.3788 | 6335763.9219 |
| Sum | 9969473.3037 | |

MS1Front TIC SCAN EI

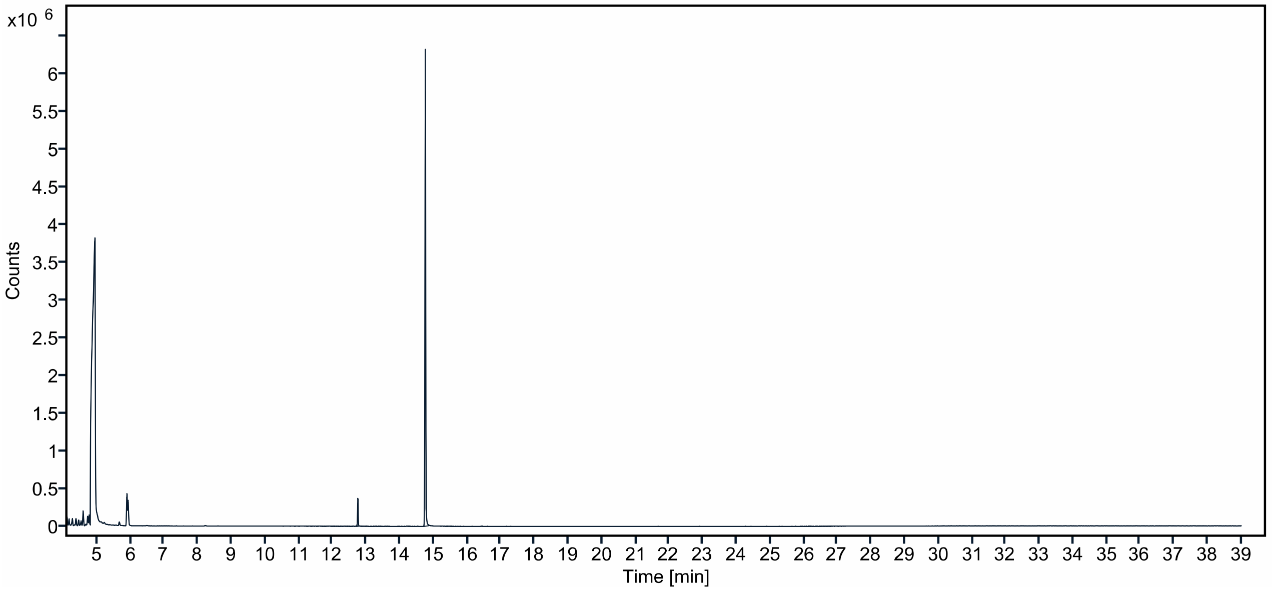


| RT | Compound Name | Score | Rev. Score | Prob. % | CAS # | Library Id |
|---|---|---|---|---|---|---|
| 12.747 | Dodecane | 909 | 914 | 17.99 | 112-40-3 | 26119 |

| RT | Compound Name | Score | Rev. Score | Prob. % | CAS # | Library Id |
|---|---|---|---|---|---|---|
| 14.76 | Ethanone, 1-(3-bromophenyl)- | 937 | 938 | 41.8 | 2142-63-4 | 187407 |

**Data file:** D:\CDSProjects\GCMS Liliana\Results\Wenjun\WJ206-9-IPA-70oC-50ppm.sirslt\WJ206-9-IPA-70oC-50ppm.dx

**Sample name:** WJ206-9-IPA-70oC-50ppm

**Description:**

**Sample amount:** 0.000

**Instrument:** GCMS

**Injection date:** 2020-02-10 23:42:41+01:00

**Acq. method:** DB5MS 60-300_WY.amx

**Analysis method:** Bob_FID+MS.pmx

**Last changed:** 2019-08-30 14:05:20+02:00

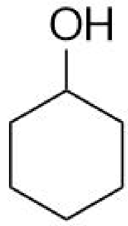


**9m**, >99%

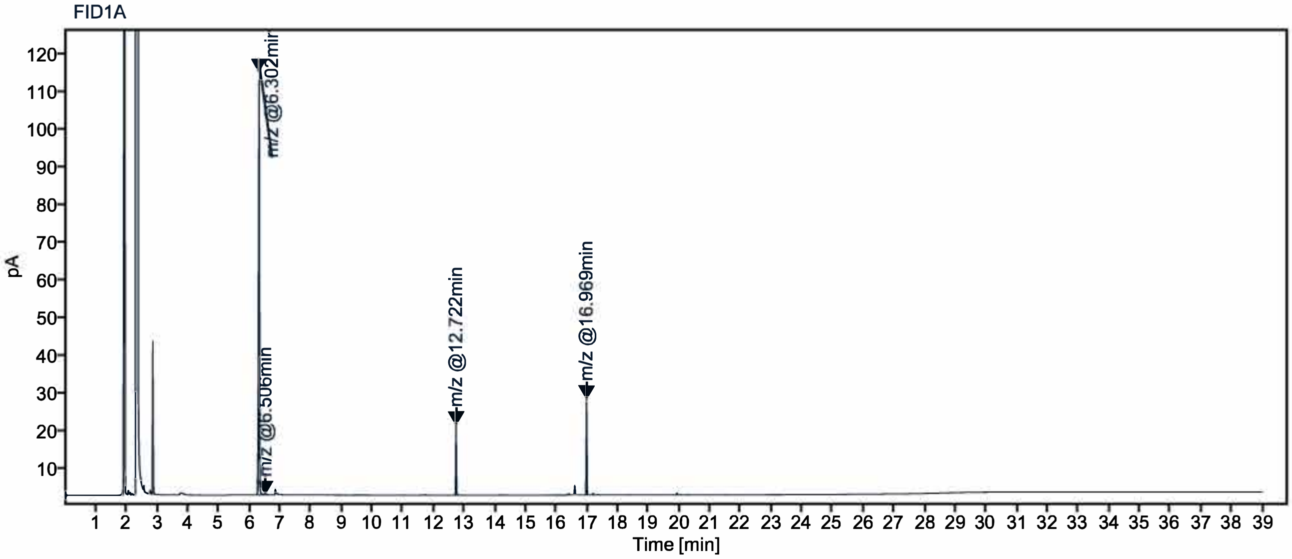


**Signal:** FID1A

| RT [min] | Area | Height |
|---|---|---|
| 6.302 | 228.0554 | 111.9545 |
| 6.506 | 0.8202 | 0.1328 |
| 12.722 | 25.2149 | 18.6525 |
| 16.969 | 35.5588 | 25.4922 |
| Sum | 289.6494 | |

**Signal:** MS1Front TIC SCAN EI

| RT [min] | Area | Height |
|---|---|---|
| 6.324 | 5400443.1213 | 2698985.9582 |
| 12.746 | 720484.1915 | 559735.0003 |
| 16.995 | 844044.0596 | 680075.7330 |
| Sum | 6964971.3723 | |

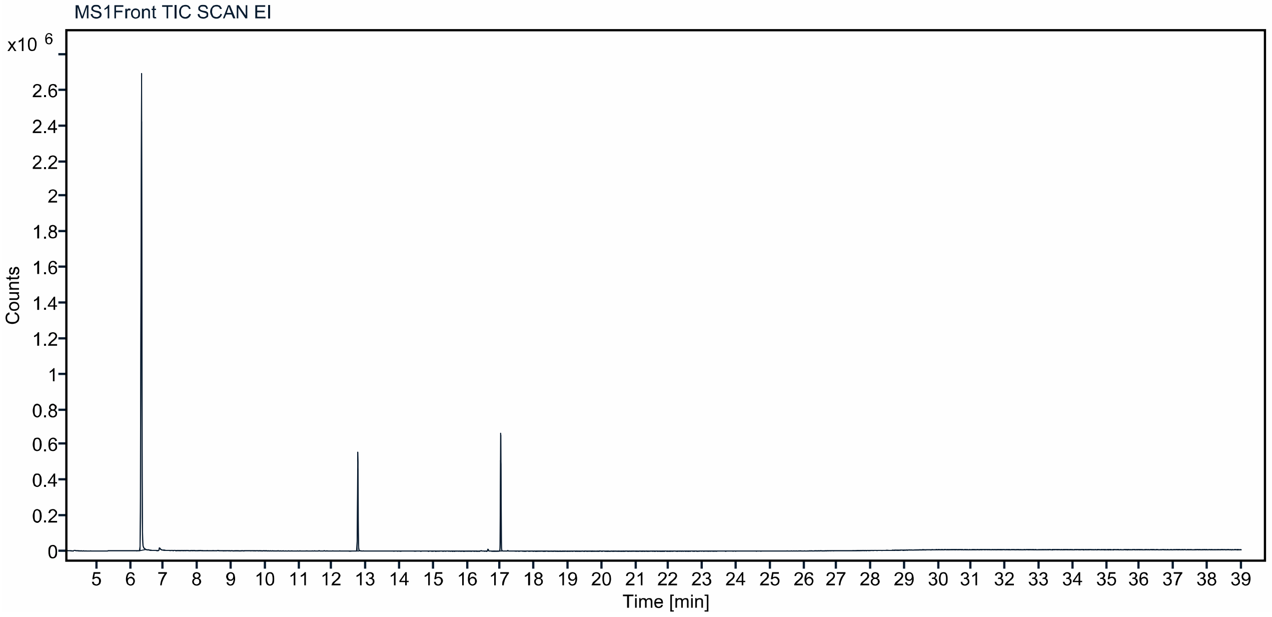


| RT | Compound Name | Score | Rev. Score | Prob. % | CAS # | Library Id |
|---|---|---|---|---|---|---|
| 6.324 | Cyclohexanol | 951 | 951 | 59.28 | 108-93-0 | 27688 |

| RT | Compound Name | Score | Rev. Score | Prob. % | CAS # | Library Id |
|---|---|---|---|---|---|---|
| 12.746 | Dodecane | 933 | 937 | 22.48 | 112-40-3 | 26119 |

| RT | Compound Name | Score | Rev. Score | Prob. % | CAS # | Library Id |
|---|---|---|---|---|---|---|
| 16.995 | Butylated Hydroxytoluene | 949 | 952 | 79.78 | 128-37-0 | 206178 |

| | |
|---|---|
| **Data file:** | D:\CDSProjects\GCMS Liliana\Results\Wenjun\WJ206-9-IPA-70oC-50ppm-initial-2.sirslt\WJ206-9-IPA-70oC-50ppm-initial-2.dx |
| **Sample name:** | WJ206-9-IPA-70oC-50ppm-initial-2 |
| **Description:** | |
| **Sample amount:** | 0.000 |
| **Instrument:** | GCMS |
| **Injection date:** | 2020-02-11 02:38:06+01:00 |
| **Acq. method:** | DB5MS 60-300_WY.amx |
| **Analysis method:** | Bob_FID+MS.pmx |
| **Last changed:** | 2019-08-30 14:05:20+02:00 |

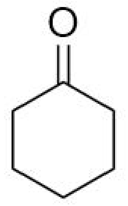

**8m**

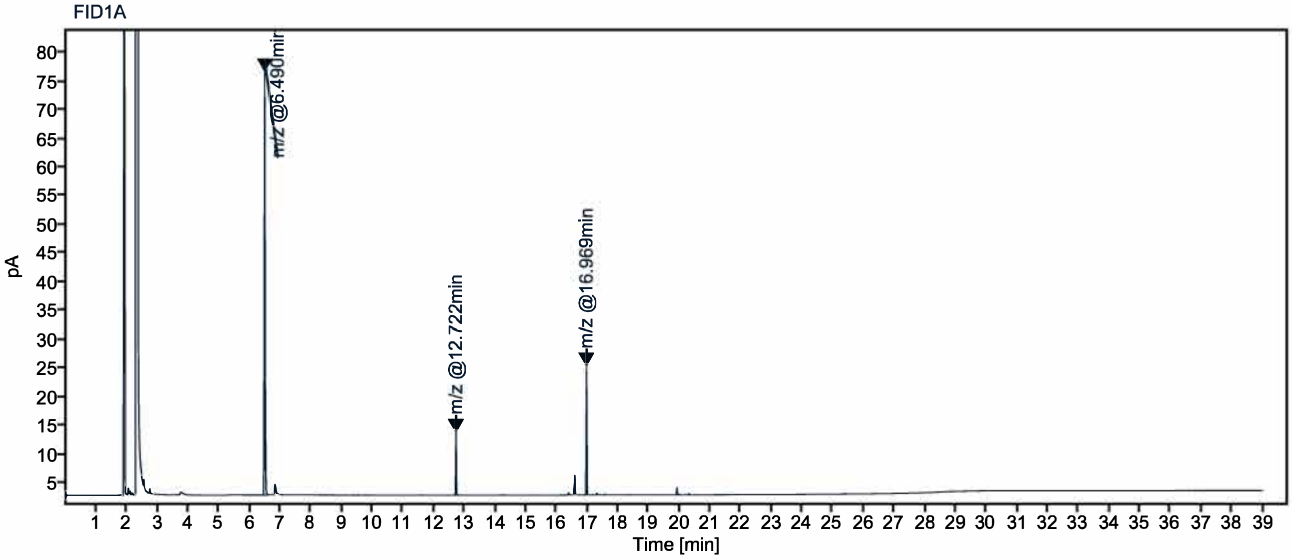


**Signal:** FID1A

| RT [min] | Area | Height |
|---|---|---|
| 6.490 | 134.0543 | 73.6597 |
| 12.722 | 15.2024 | 10.9860 |
| 16.969 | 31.8215 | 22.4821 |
| Sum | 181.0782 | |

**Signal:** MS1Front TIC SCAN EI

| RT [min] | Area | Height |
|---|---|---|
| 6.512 | 2974682.5138 | 1663673.0276 |
| 12.746 | 374151.7481 | 285629.3670 |
| 16.995 | 656168.0188 | 517523.1766 |
| Sum | 4005002.2807 | |

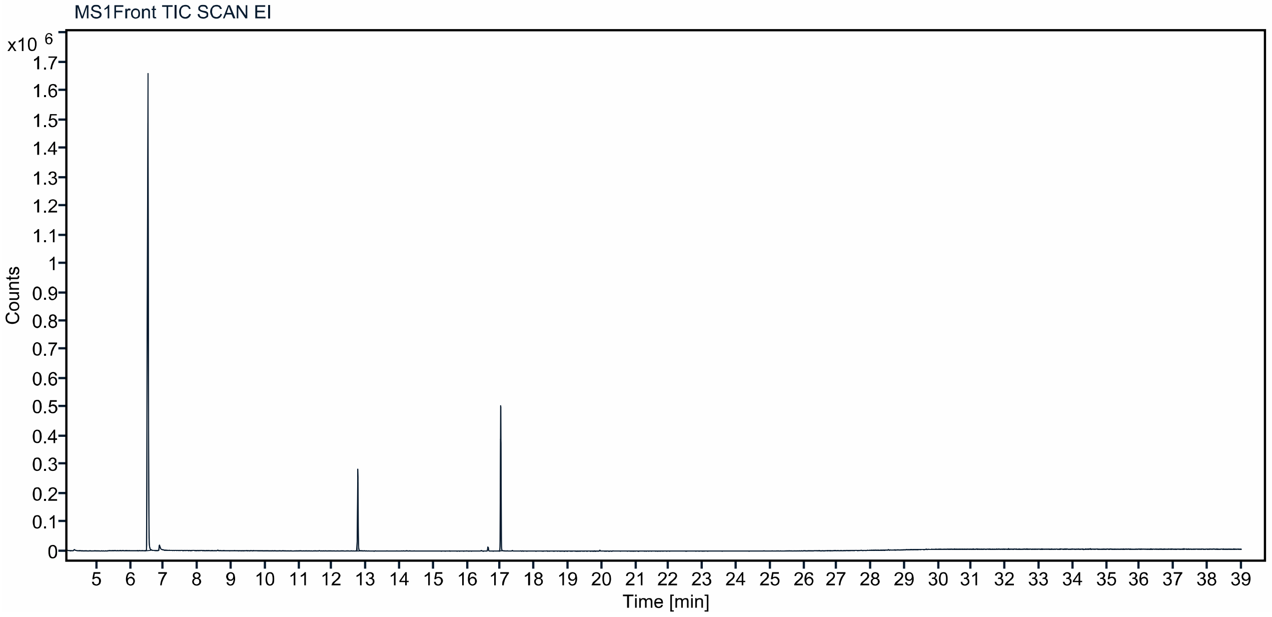


| RT | Compound Name | Score | Rev. Score | Prob. % | CAS # | Library Id |
|---|---|---|---|---|---|---|
| 6.512 | Cyclopentanone, 2-methyl- | 927 | 945 | 50.03 | 1120-72-5 | 5100 |

| RT | Compound Name | Score | Rev. Score | Prob. % | CAS # | Library Id |
|---|---|---|---|---|---|---|
| 12.746 | Dodecane | 911 | 916 | 21.75 | 112-40-3 | 26119 |

| RT | Compound Name | Score | Rev. Score | Prob. % | CAS # | Library Id |
|---|---|---|---|---|---|---|
| 16.995 | Butylated Hydroxytoluene | 946 | 949 | 81.47 | 128-37-0 | 206178 |

Agilent Technologies

**Data file:** D:\CDSProjects\GCMS Liliana\Results\Wenjun\WJ206-32-IPA-70oC-50ppm.sirslt\WJ206-32-IPA-70oC-50ppm.dx
**Sample name:** WJ206-32-IPA-70oC-50ppm
**Description:**
**Sample amount:** 0.000

**Instrument:** GCMS
**Injection date:** 2020-02-11 01:10:25+01:00
**Acq. method:** DB5MS 60-300_WY.amx
**Analysis method:** Bob_FID+MS.pmx
**Last changed:** 2019-08-30 14:05:20+02:00

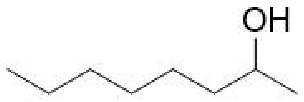

**9n**, >99%

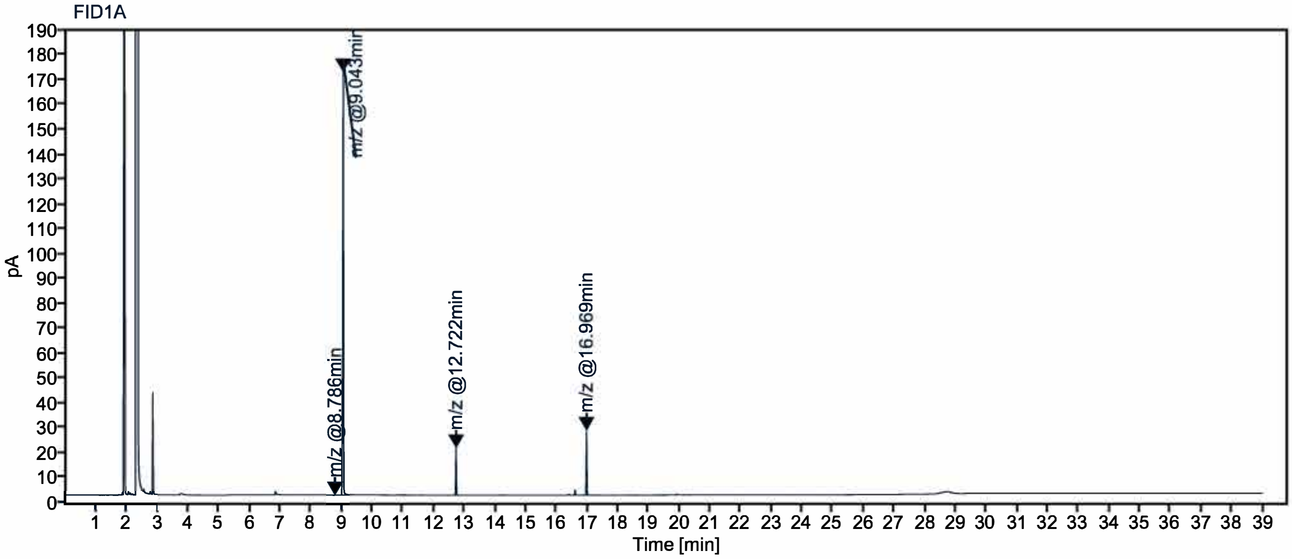


**Signal:** FID1A

| RT [min] | Area | Height |
|---|---|---|
| 8.786 | 0.0501 | 0.0347 |
| 9.043 | 314.0849 | 170.2418 |
| 12.722 | 26.2094 | 19.0489 |
| 16.969 | 35.9780 | 26.0262 |
| Sum | 376.3224 | |

**Signal:** MS1Front TIC SCAN EI

| RT [min] | Area | Height |
|---|---|---|
| 9.065 | 8263961.6601 | 4702874.7812 |
| 12.746 | 733296.2342 | 560777.9173 |
| 16.995 | 832776.2779 | 669581.0892 |
| Sum | 9830034.1722 | |

Agilent Technologies

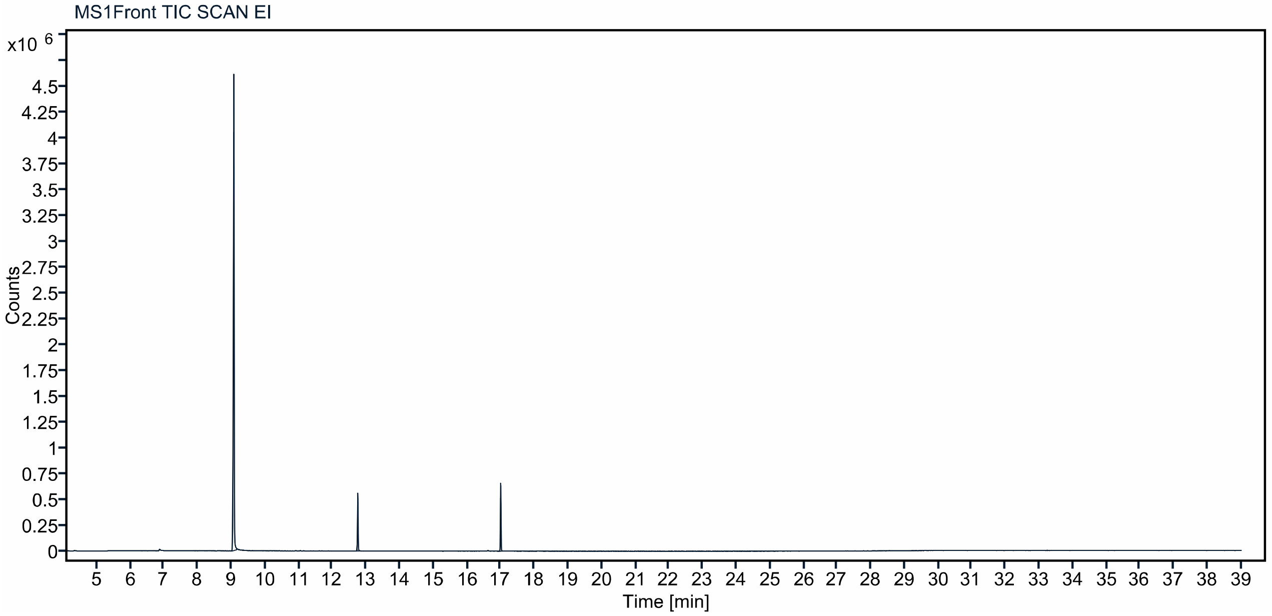


| RT | Compound Name | Score | Rev. Score | Prob. % | CAS # | Library Id |
|---|---|---|---|---|---|---|
| 9.065 | 2-Octanol, (R)- | 948 | 950 | 33.78 | 5978-70-1 | 18457 |

| RT | Compound Name | Score | Rev. Score | Prob. % | CAS # | Library Id |
|---|---|---|---|---|---|---|
| 12.746 | Dodecane | 938 | 942 | 23.03 | 112-40-3 | 26119 |

| RT | Compound Name | Score | Rev. Score | Prob. % | CAS # | Library Id |
|---|---|---|---|---|---|---|
| 16.995 | Butylated Hydroxytoluene | 948 | 951 | 81.55 | 128-37-0 | 206178 |

# Single Injection Report

Agilent Technologies

**Data file:** D:\CDSProjects\GCMS Liliana\Results\Wenjun\WJ206-32-IPA-70oC-50ppm-initial.sirslt\WJ206-32-IPA-70oC-50ppm-initial.dx
**Sample name:** WJ206-32-IPA-70oC-50ppm-initial
**Description:**
**Sample amount:** 0.000

**Instrument:** GCMS
**Injection date:** 2020-02-11 03:21:56+01:00
**Acq. method:** DB5MS 60-300_WY.amx
**Analysis method:** Bob_FID+MS.pmx
**Last changed:** 2019-08-30 14:05:20+02:00

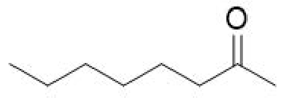

8n

FID1A

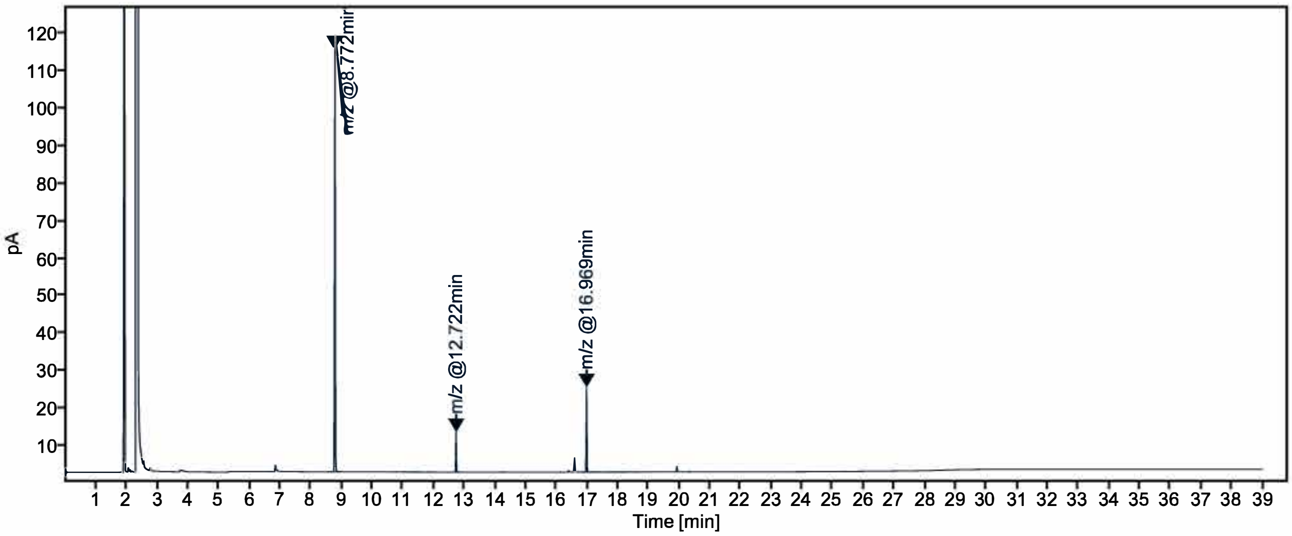


**Signal:** FID1A

| RT [min] | Area | Height |
|---|---|---|
| 8.772 | 176.6635 | 112.9304 |
| 12.722 | 14.8873 | 10.7572 |
| 16.969 | 31.0794 | 22.7359 |
| Sum | 222.6302 | |

**Signal:** MS1Front TIC SCAN EI

| RT [min] | Area | Height |
|---|---|---|
| 8.793 | 4771235.8098 | 3101960.8969 |
| 12.746 | 365825.8813 | 272229.9668 |
| 16.995 | 626029.5827 | 504917.4131 |
| Sum | 5763091.2739 | |

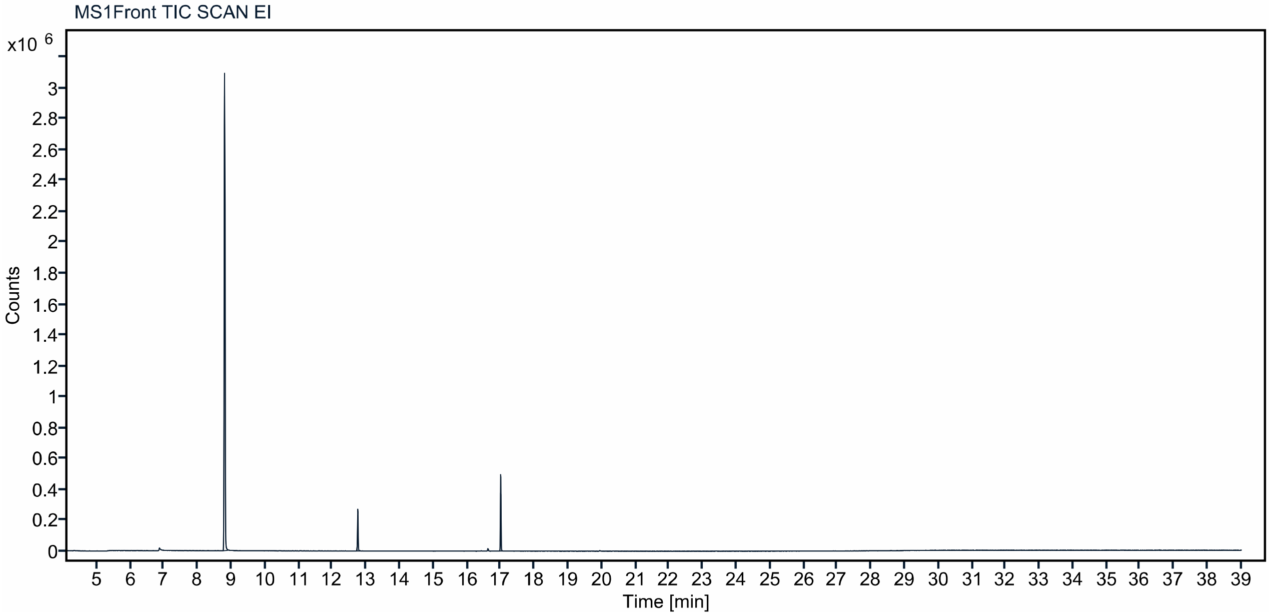


| RT | Compound Name | Score | Rev. Score | Prob. % | CAS # | Library Id |
|---|---|---|---|---|---|---|
| 8.793 | 2-Octanone | 937 | 938 | 81.08 | 111-13-7 | 8621 |

| RT | Compound Name | Score | Rev. Score | Prob. % | CAS # | Library Id |
|---|---|---|---|---|---|---|
| 12.746 | Dodecane | 903 | 912 | 20.18 | 112-40-3 | 26119 |

| RT | Compound Name | Score | Rev. Score | Prob. % | CAS # | Library Id |
|---|---|---|---|---|---|---|
| 16.995 | Butylated Hydroxytoluene | 938 | 941 | 79.3 | 128-37-0 | 206178 |

# Single Injection Report

Agilent Technologies

**Data file:** D:\CDSProjects\GCMS Liliana\Results\Wenjun\WJ206-6-ipa-50PPM-2.sirslt\WJ206-6-ipa-50PPM-2.dx
**Sample name:** WJ206-6-ipa-50PPM-2
**Description:**
**Sample amount:** 0.000

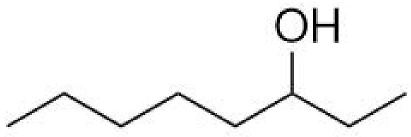


**9o**, >99%

**Instrument:** GCMS
**Injection date:** 2020-01-14 12:46:50+01:00
**Acq. method:** DB5MS 60-300_WY.amx
**Analysis method:** Bob_FID+MS.pmx
**Last changed:** 2019-08-30 14:05:20+02:00

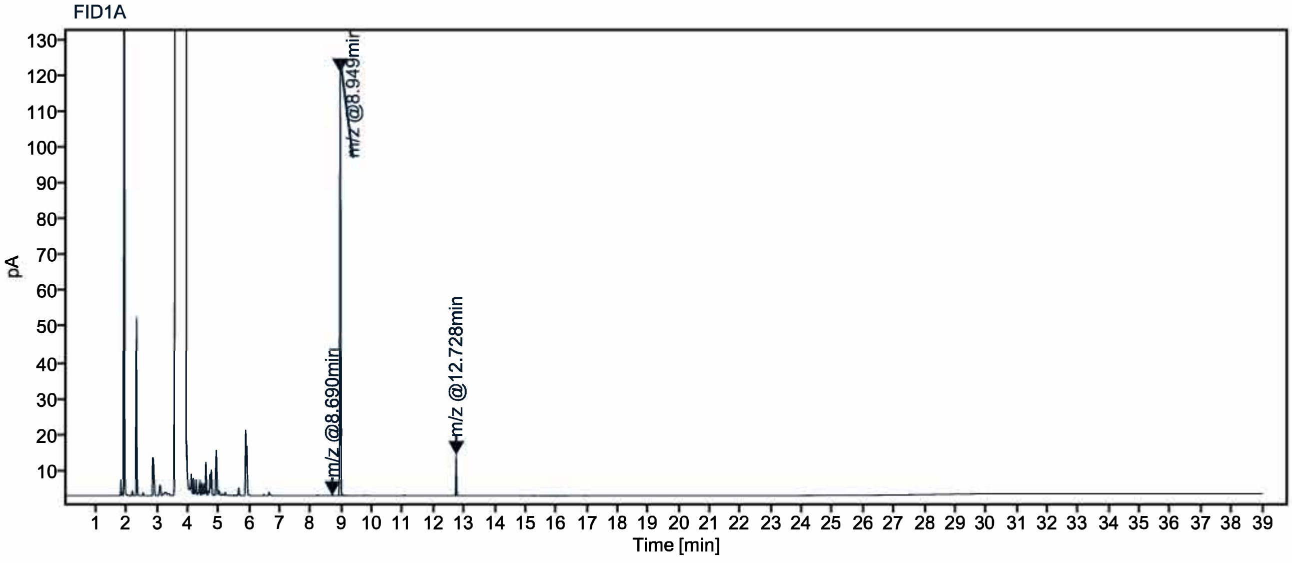


**Signal:** FID1A

| RT [min] | Area | Height |
|---|---|---|
| 8.690 | 0.4261 | 0.1905 |
| 8.949 | 191.8232 | 118.3783 |
| 12.728 | 15.8025 | 11.5735 |
| Sum | 208.0517 | |

**Signal:** MS1Front TIC SCAN EI

| RT [min] | Area | Height |
|---|---|---|
| 8.720 | 4912.4561 | 3347.4956 |
| 8.971 | 6076599.6229 | 3913409.7763 |
| 12.751 | 505106.2061 | 383999.0856 |
| Sum | 6586618.2851 | |

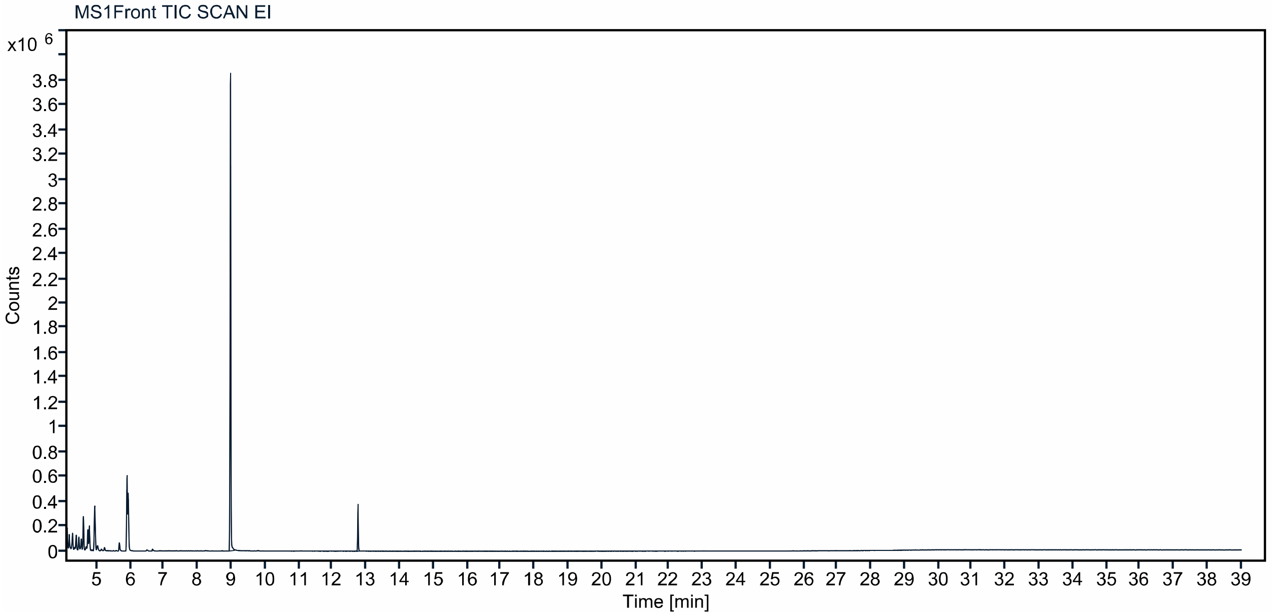


| RT | Compound Name | Score | Rev. Score | Prob. % | CAS # | Library Id |
|---|---|---|---|---|---|---|
| 8.971 | 3-Octanol | 940 | 959 | 72.51 | 589-98-0 | 32467 |

| RT | Compound Name | Score | Rev. Score | Prob. % | CAS # | Library Id |
|---|---|---|---|---|---|---|
| 12.751 | Dodecane | 927 | 932 | 22.39 | 112-40-3 | 26119 |

# Single Injection Report

**Data file:** D:\CDSProjects\GCMS Liliana\Results\Wenjun\WJ206-6-ipa-50PPM-initial-2.sirslt\WJ206-6-ipa-50PPM-initial-2.dx

**Sample name:** WJ206-6-ipa-50PPM-initial-2

**Description:**

**Sample amount:** 0.000

**Instrument:** GCMS

**Injection date:** 2020-01-14 17:09:43+01:00

**Acq. method:** DB5MS 60-300_WY.amx

**Analysis method:** Bob_FID+MS.pmx

**Last changed:** 2019-08-30 14:05:20+02:00

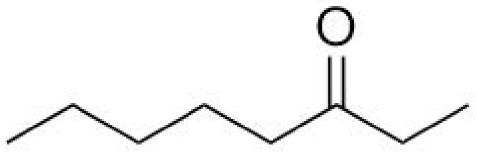

**8o**

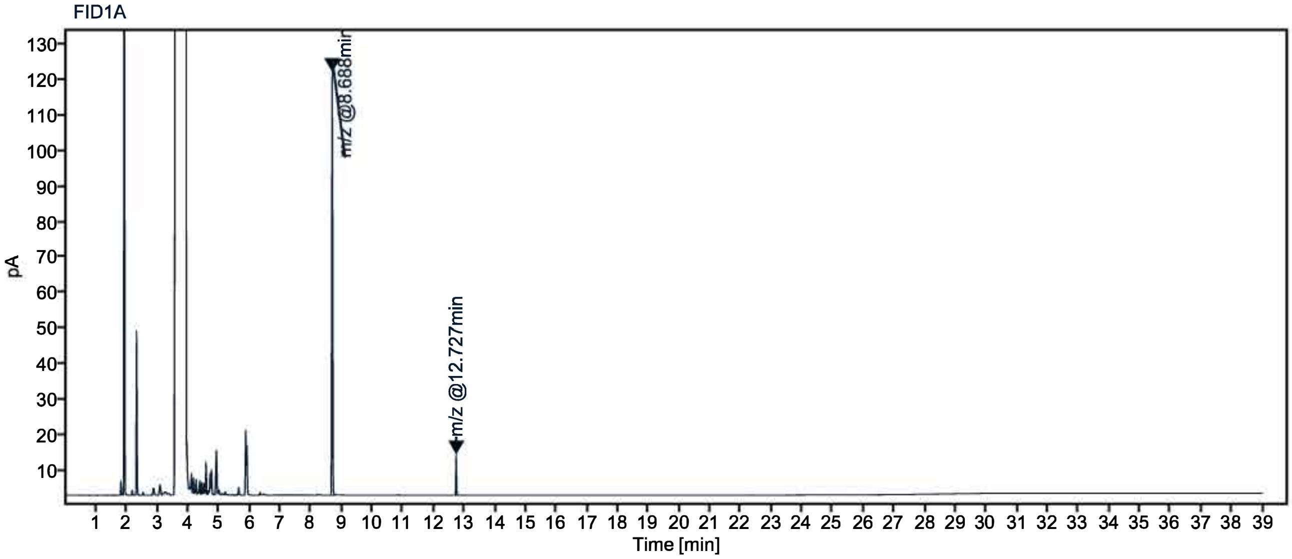


**Signal:** FID1A

| RT [min] | Area | Height |
|---|---|---|
| 8.688 | 186.3100 | 119.5323 |
| 12.727 | 15.8357 | 11.6210 |
| Sum | 202.1456 | |

**Signal:** MS1Front TIC SCAN EI

| RT [min] | Area | Height |
|---|---|---|
| 8.710 | 5061623.2418 | 3339678.2639 |
| 12.751 | 453452.9693 | 347321.6038 |
| Sum | 5515076.2111 | |

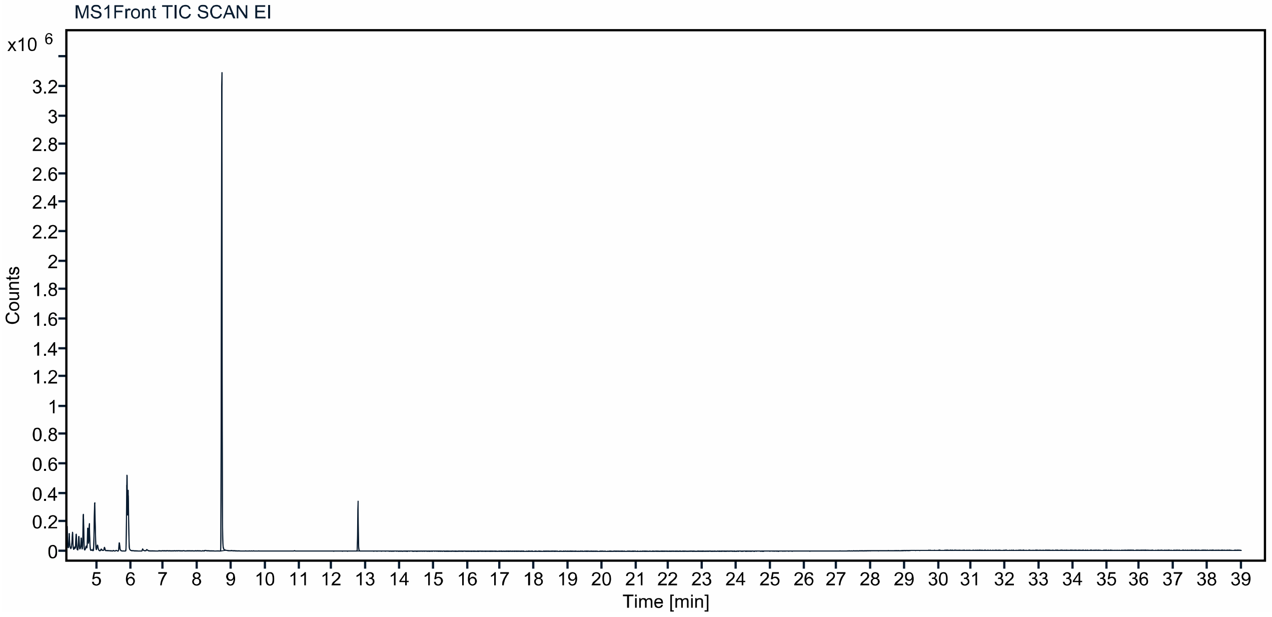


| RT | Compound Name | Score | Rev. Score | Prob. % | CAS # | Library Id |
|---|---|---|---|---|---|---|
| 8.71 | 3-Octanone | 940 | 942 | 72.91 | 106-68-3 | 8527 |

| RT | Compound Name | Score | Rev. Score | Prob. % | CAS # | Library Id |
|---|---|---|---|---|---|---|
| 12.751 | Dodecane | 921 | 924 | 20.25 | 112-40-3 | 26119 |

# Single Injection Report

Agilent Technologies

| | |
|---|---|
| **Data file:** | D:\CDSProjects\GCMS Liliana\Results\Wenjun\WJ206-4-IPA-50PPM.sirslt\WJ206-4-IPA-50PPM.dx |
| **Sample name:** | WJ206-4-IPA-50PPM |
| **Description:** | |
| **Sample amount:** | 0.000 |
| **Instrument:** | GCMS |
| **Injection date:** | 2020-01-14 12:03:07+01:00 |
| **Acq. method:** | DB5MS 60-300_WY.amx |
| **Analysis method:** | Bob_FID+MS.pmx |
| **Last changed:** | 2019-08-30 14:05:20+02:00 |

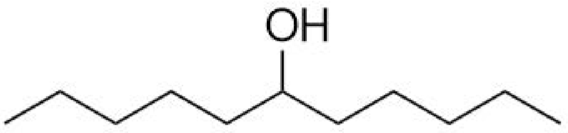


**9p**, >99%

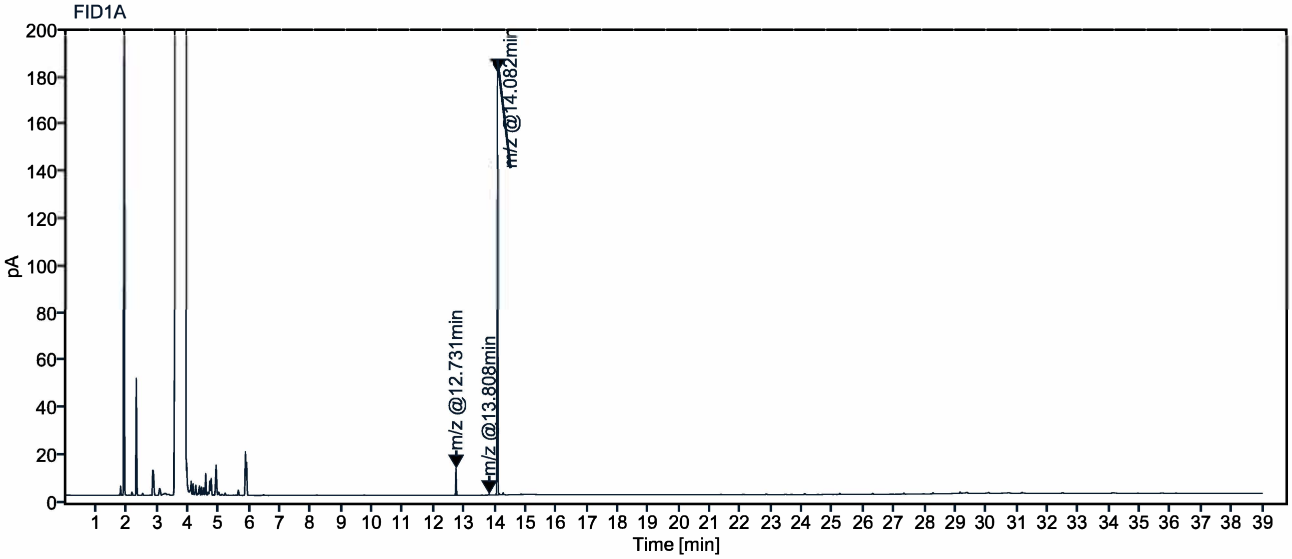


**Signal:** FID1A

| RT [min] | Area | Height |
|---|---|---|
| 12.731 | 15.7747 | 11.4620 |
| 13.808 | 0.8046 | 0.5766 |
| 14.082 | 263.5956 | 179.2062 |
| Sum | 280.1750 | |

**Signal:** MS1Front TIC SCAN EI

| RT [min] | Area | Height |
|---|---|---|
| 12.754 | 514923.9560 | 394516.4844 |
| 13.830 | 12049.0411 | 8745.0783 |
| 14.105 | 10312501.2672 | 7558452.6701 |
| Sum | 10839474.2643 | |

MS1Front TIC SCAN EI

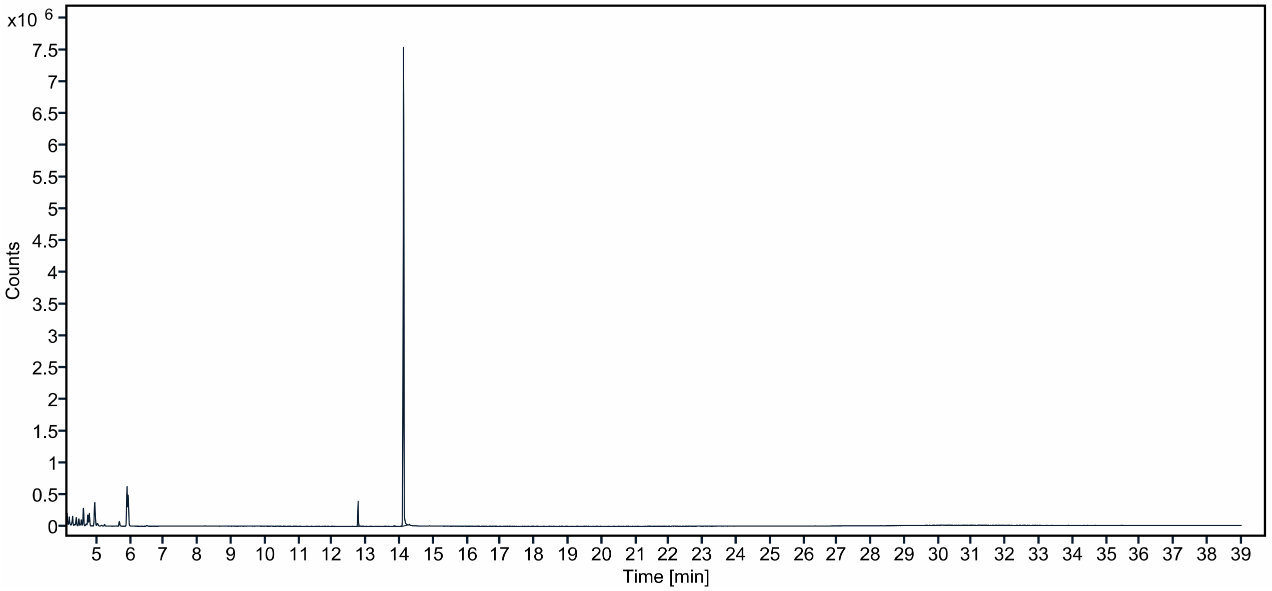


| RT | Compound Name | Score | Rev. Score | Prob. % | CAS # | Library Id |
|---|---|---|---|---|---|---|
| 12.754 | Dodecane | 939 | 943 | 26.05 | 112-40-3 | 26119 |

| RT | Compound Name | Score | Rev. Score | Prob. % | CAS # | Library Id |
|---|---|---|---|---|---|---|
| 13.83 | 6-Undecanone | 825 | 825 | 65.67 | 927-49-1 | 11428 |

| RT | Compound Name | Score | Rev. Score | Prob. % | CAS # | Library Id |
|---|---|---|---|---|---|---|
| 14.105 | 6-Undecanol | 916 | 923 | 63.54 | 23708-56-7 | 57013 |

# Single Injection Report

Agilent Technologies

| | |
|---|---|
| **Data file:** | D:\CDSProjects\GCMS Liliana\Results\Wenjun\WJ206-4-ipa-50PPM-initial.sirslt\WJ206-4-ipa-50PPM-initial.dx |
| **Sample name:** | WJ206-4-ipa-50PPM-initial |
| **Description:** | |
| **Sample amount:** | 0.000 |
| **Instrument:** | GCMS |
| **Injection date:** | 2020-01-14 16:25:57+01:00 |
| **Acq. method:** | DB5MS 60-300_WY.amx |
| **Analysis method:** | Bob_FID+MS.pmx |
| **Last changed:** | 2019-08-30 14:05:20+02:00 |

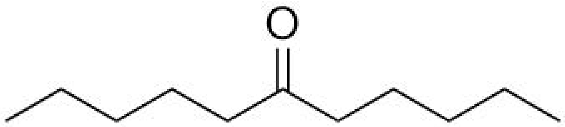

**8p**

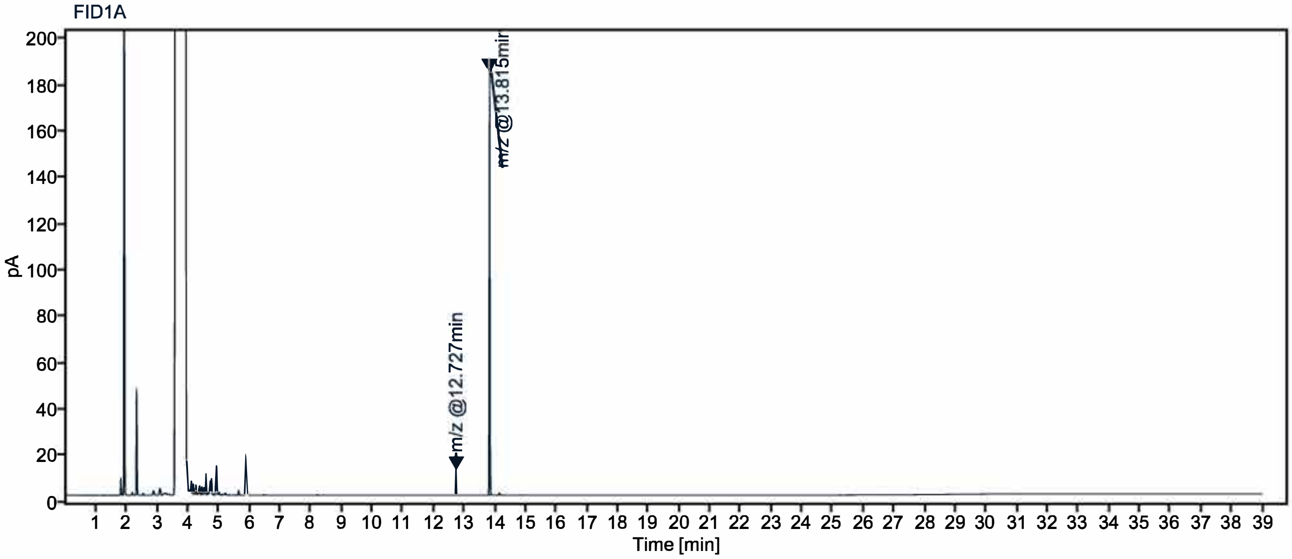


**Signal:** FID1A

| RT [min] | Area | Height |
|---|---|---|
| 12.727 | 14.9898 | 10.8843 |
| 13.815 | 255.9865 | 182.7660 |
| Sum | 270.9762 | |

**Signal:** MS1Front TIC SCAN EI

| RT [min] | Area | Height |
|---|---|---|
| 12.751 | 428763.2052 | 323159.4695 |
| 13.838 | 9383325.71 24 | 6891505.77 59 |
| Sum | 9812088.91 75 | |

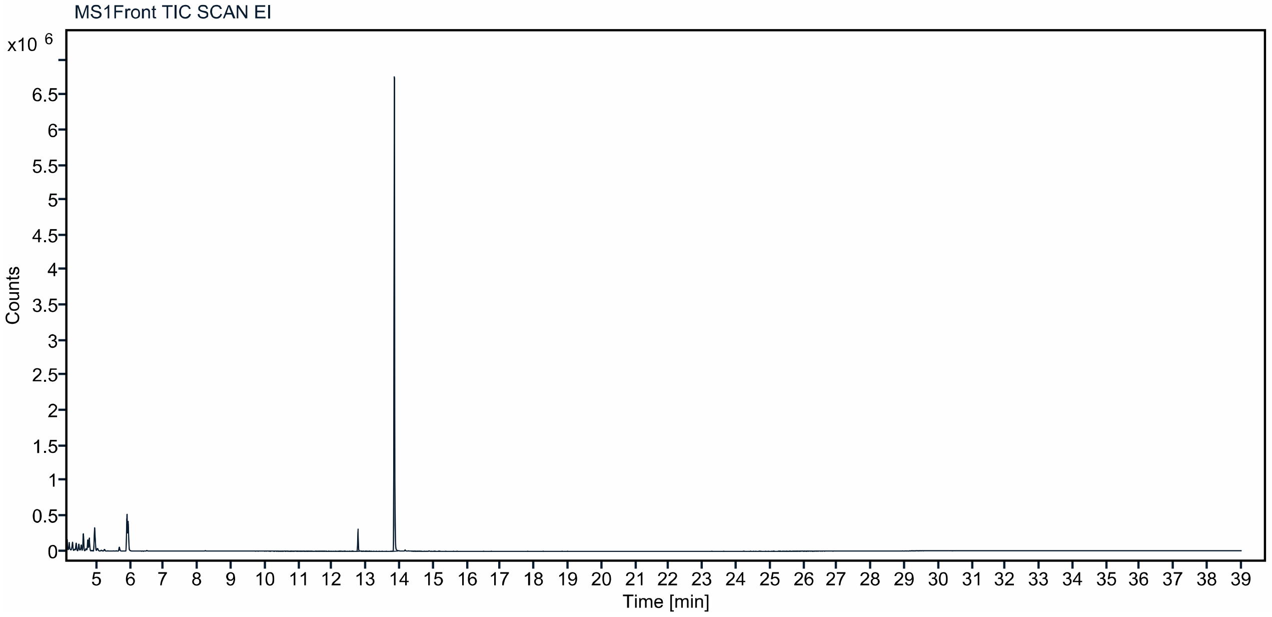


| RT | Compound Name | Score | Rev. Score | Prob. % | CAS # | Library Id |
|---|---|---|---|---|---|---|
| 12.751 | Dodecane | 922 | 927 | 20.53 | 112-40-3 | 26119 |

| RT | Compound Name | Score | Rev. Score | Prob. % | CAS # | Library Id |
|---|---|---|---|---|---|---|
| 13.838 | 6-Undecanone | 931 | 931 | 86.23 | 927-49-1 | 11428 |

# Single Injection Report

Agilent Technologies

**Data file:** D:\CDSProjects\GCMS Liliana\Results\Wenjun\WJ206-35-IPA-80-50PPM-5mmol-50bar.sirslt\WJ206-35-IPA-80-50PPM-5mmol-50bar.dx

**Sample name:** WJ206-35-IPA-80-50PPM-5mmol-50bar

**Description:**

**Sample amount:** 0.000

**Instrument:** GCMS

**Injection date:** 2020-02-15 15:15:48+01:00

**Acq. method:** DB5MS 60-300_WY.amx

**Analysis method:** Bob_FID+MS.pmx

**Last changed:** 2019-08-30 14:05:20+02:00

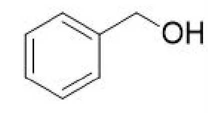


**9r**, 99%

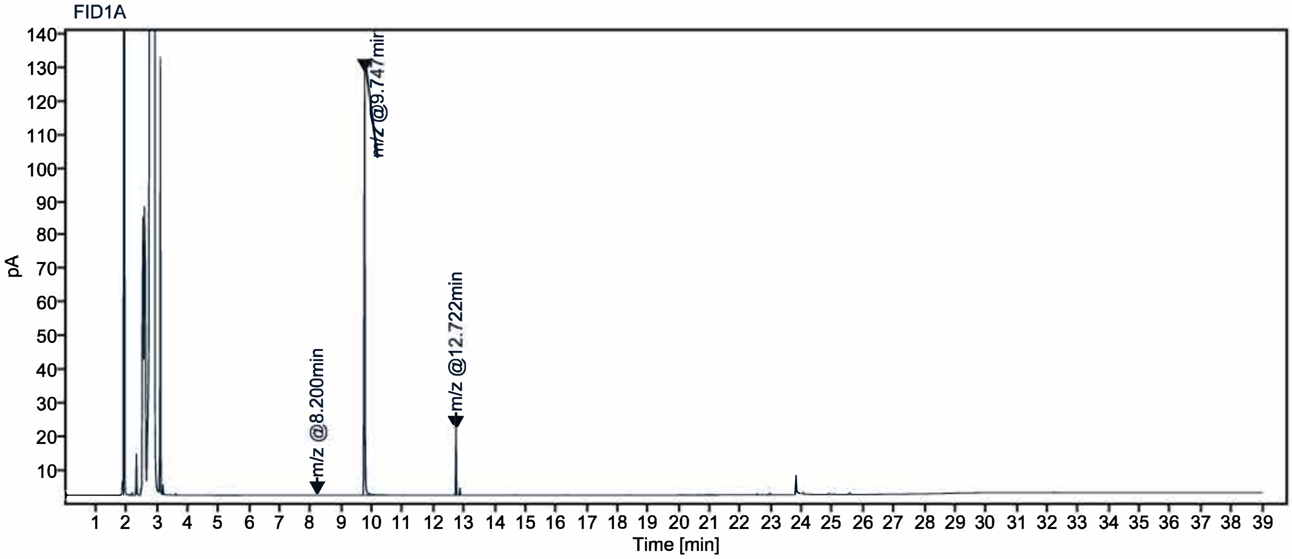


**Signal:** FID1A

| RT [min] | Area | Height |
|---|---|---|
| 8.200 | 0.1667 | 0.0375 |
| 9.747 | 251.1583 | 125.5393 |
| 12.722 | 26.7581 | 19.5907 |
| Sum | 278.0830 | |

**Signal:** MS1Front TIC SCAN EI

| RT [min] | Area | Height |
|---|---|---|
| 9.768 | 5799118.1920 | 3112859.5081 |
| 12.745 | 731518.8642 | 562001.2296 |
| Sum | 6530637.0562 | |

# Single Injection Report

Agilent Technologies

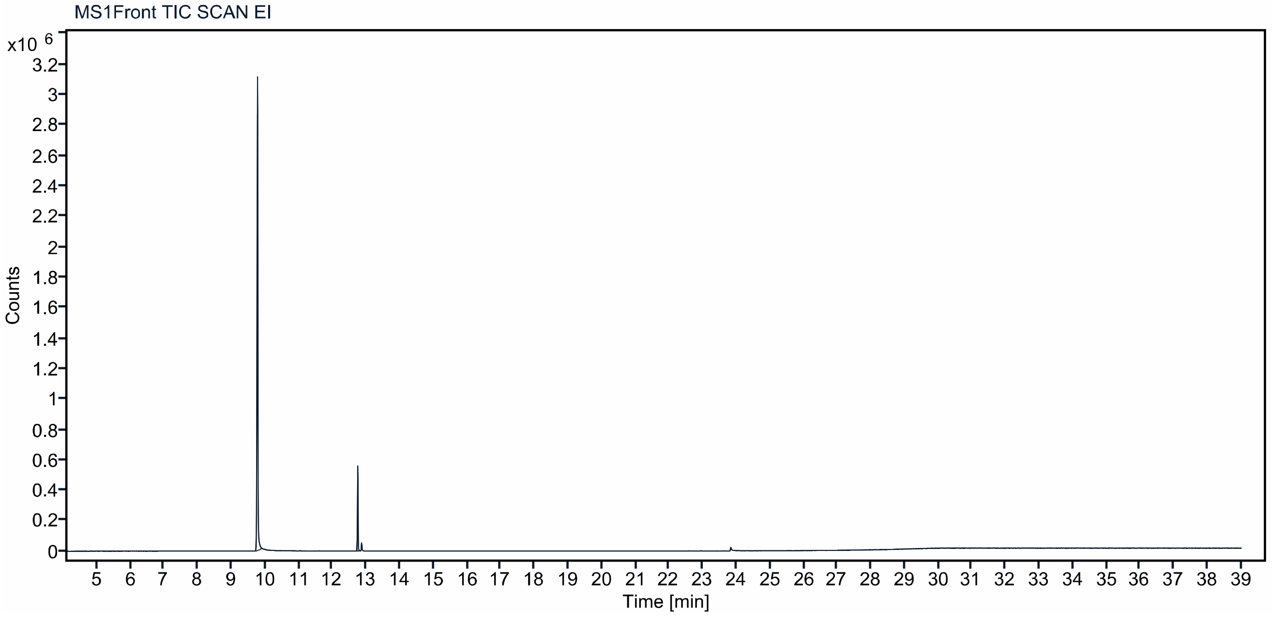


| RT | Compound Name | Score | Rev. Score | Prob. % | CAS # | Library Id |
|---|---|---|---|---|---|---|
| 9.768 | Benzyl alcohol | 949 | 950 | 65.26 | 100-51-6 | 53588 |

| RT | Compound Name | Score | Rev. Score | Prob. % | CAS # | Library Id |
|---|---|---|---|---|---|---|
| 12.745 | Dodecane | 931 | 935 | 21.71 | 112-40-3 | 26119 |

# Single Injection Report

Agilent Technologies

**Data file:** D:\CDSProjects\GCMS Liliana\Results\Wenjun\WJ206-35-IPA-80-50PPM-5mmol-50bar-initial.sirslt\WJ206-35-IPA-80-50PPM-5mmol-50bar-initial.dx

**Sample name:** WJ206-35-IPA-80-50PPM-5mmol-50bar-initial

**Description:**

**Sample amount:** 0.000

**Instrument:** GCMS

**Injection date:** 2020-02-15 15:59:37+01:00

**Acq. method:** DB5MS 60-300_WY.amx

**Analysis method:** Bob_FID+MS.pmx

**Last changed:** 2019-08-30 14:05:20+02:00

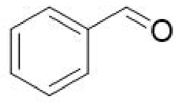

**8r**

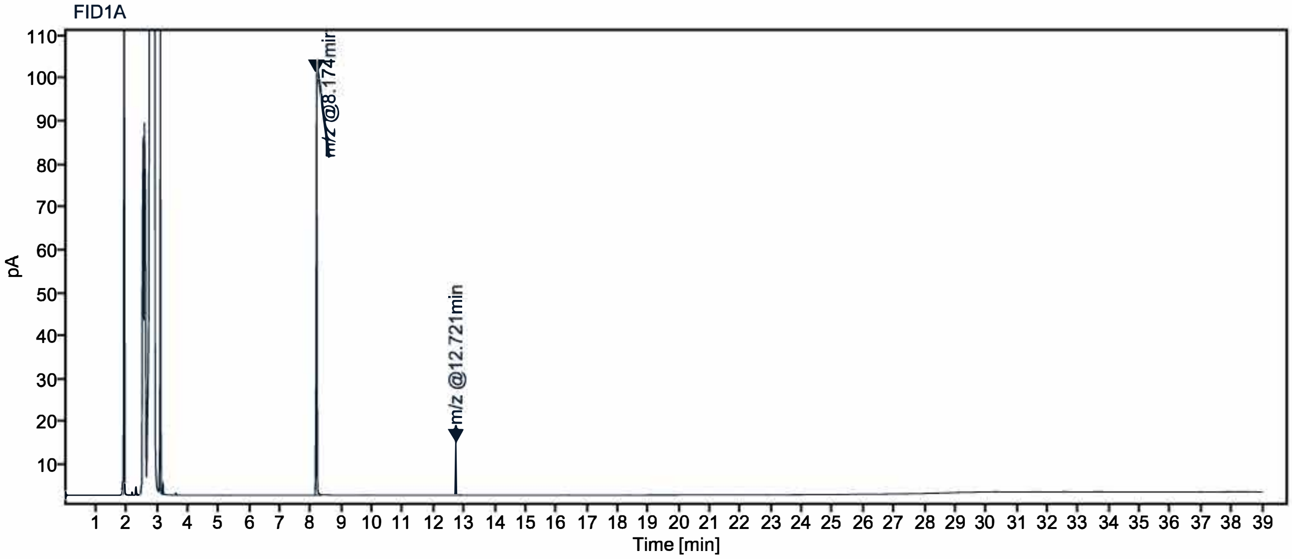


**Signal:** FID1A

| RT [min] | Area | Height |
|---|---|---|
| 8.174 | 168.4509 | 98.4905 |
| 12.721 | 16.8661 | 12.3071 |
| Sum | 185.3170 | |

**Signal:** MS1Front TIC SCAN EI

| RT [min] | Area | Height |
|---|---|---|
| 8.196 | 4399759.24 79 | 2627529.51 30 |
| 12.744 | 421370.4537 | 319272.3221 |
| Sum | 4821129.70 16 | |

# Single Injection Report

Agilent Technologies

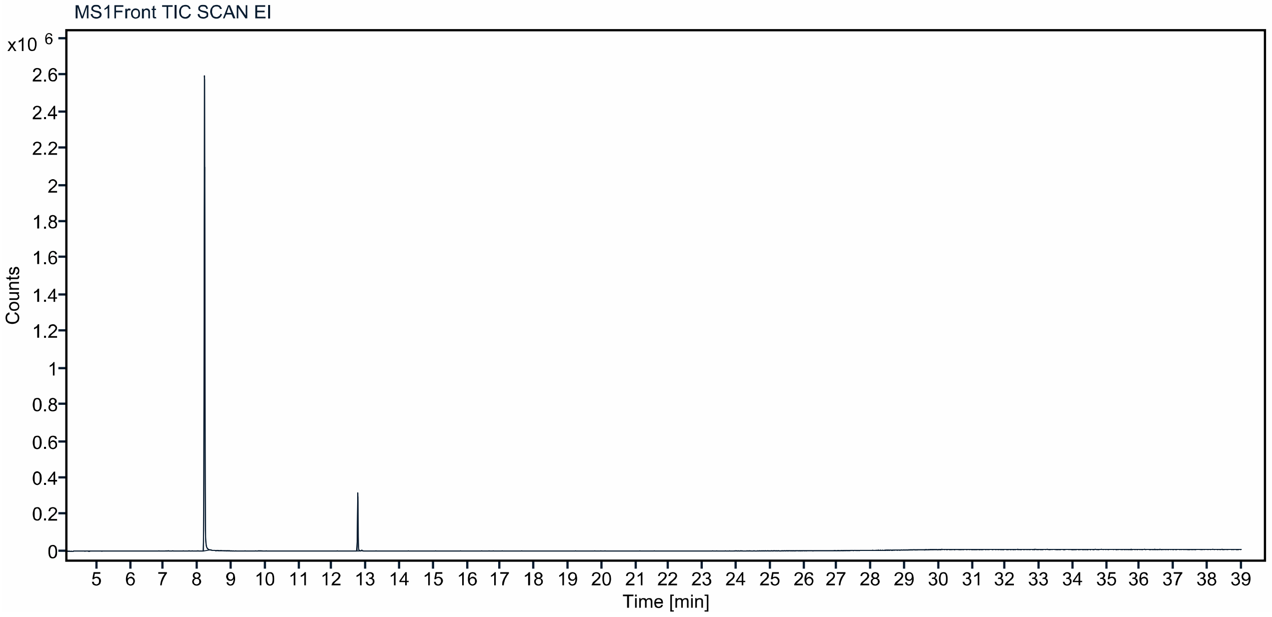


| RT | Compound Name | Score | Rev. Score | Prob. % | CAS # | Library Id |
|---|---|---|---|---|---|---|
| 8.196 | Benzaldehyde | 929 | 932 | 74 | 100-52-7 | 51446 |

| RT | Compound Name | Score | Rev. Score | Prob. % | CAS # | Library Id |
|---|---|---|---|---|---|---|
| 12.744 | Dodecane | 919 | 925 | 19.28 | 112-40-3 | 26119 |

# Single Injection Report

Agilent Technologies

| | |
|---|---|
| **Data file:** | D:\CDSProjects\GCMS Liliana\Results\Wenjun\WJ206-45-80-IPA-50PPM-quench.sirslt\WJ206-45-80-IPA-50PPM-quench.dx |
| **Sample name:** | WJ206-45-80-IPA-50PPM-quench |
| **Description:** | |
| **Sample amount:** | 0.000 |
| **Instrument:** | GCMS |
| **Injection date:** | 2020-03-07 22:11:43+01:00 |
| **Acq. method:** | DB5MS 60-300_WY.amx |
| **Analysis method:** | Bob_FID+MS.pmx |
| **Last changed:** | 2019-08-30 14:05:20+02:00 |

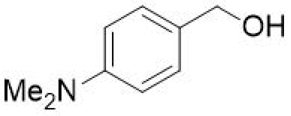


**9s**, >99%

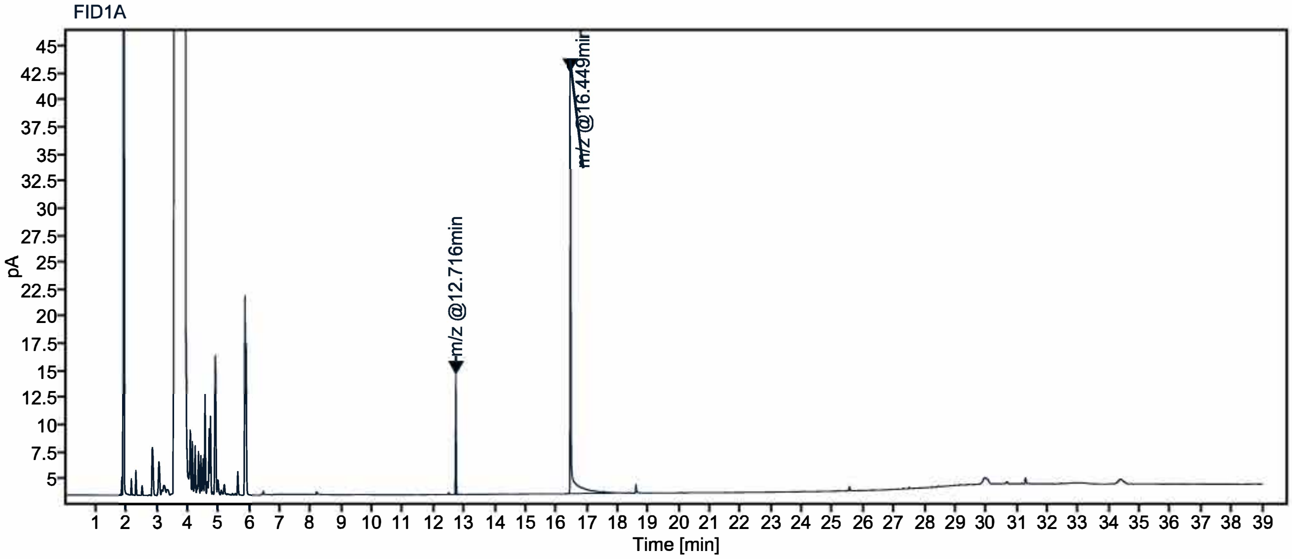


**Signal:** FID1A

| RT [min] | Area | Height |
|---|---|---|
| 12.716 | 15.0980 | 11.1263 |
| 16.449 | 87.4223 | 38.9619 |
| Sum | 102.5203 | |

**Signal:** MS1Front TIC SCAN EI

| RT [min] | Area | Height |
|---|---|---|
| 12.738 | 464054.6985 | 348572.4852 |
| 16.475 | 1069806.92 51 | 671879.8464 |
| Sum | 1533861.62 37 | |

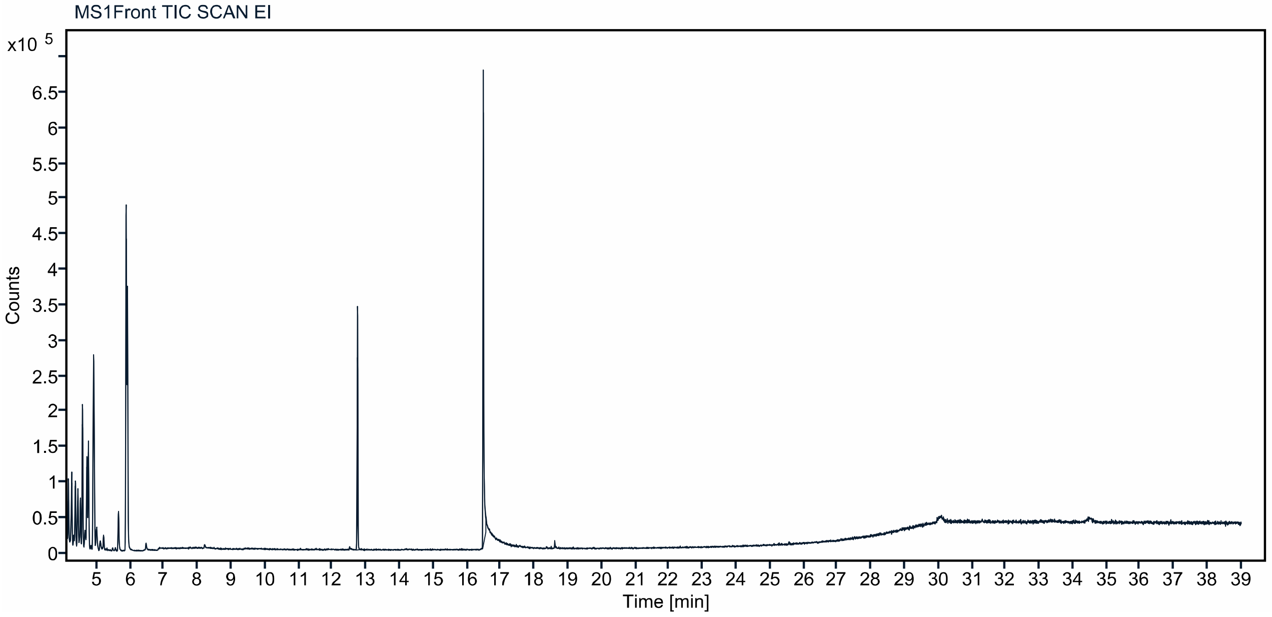


| RT | Compound Name | Score | Rev. Score | Prob. % | CAS # | Library Id |
|---|---|---|---|---|---|---|
| 12.738 | Dodecane | 922 | 930 | 24.19 | 112-40-3 | 26119 |

| RT | Compound Name | Score | Rev. Score | Prob. % | CAS # | Library Id |
|---|---|---|---|---|---|---|
| 16.475 | Benzenemethanol, 4-(dimethylamino)- | 808 | 941 | 61.9 | 1703-46-4 | 130010 |

# Single Injection Report

Agilent Technologies

**Data file:** D:\CDSProjects\GCMS Liliana\Results\Wenjun\WJ206-45-80-IPA-50PPM-initial.sirslt\WJ206-45-80-IPA-50PPM-initial.dx

**Sample name:** WJ206-45-80-IPA-50PPM-initial

**Description:**

**Sample amount:** 0.000

**Instrument:** GCMS

**Injection date:** 2020-03-07 22:55:35+01:00

**Acq. method:** DB5MS 60-300_WY.amx

**Analysis method:** Bob_FID+MS.pmx

**Last changed:** 2019-08-30 14:05:20+02:00

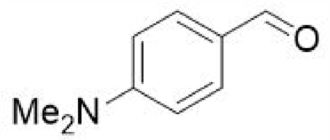


**8s**

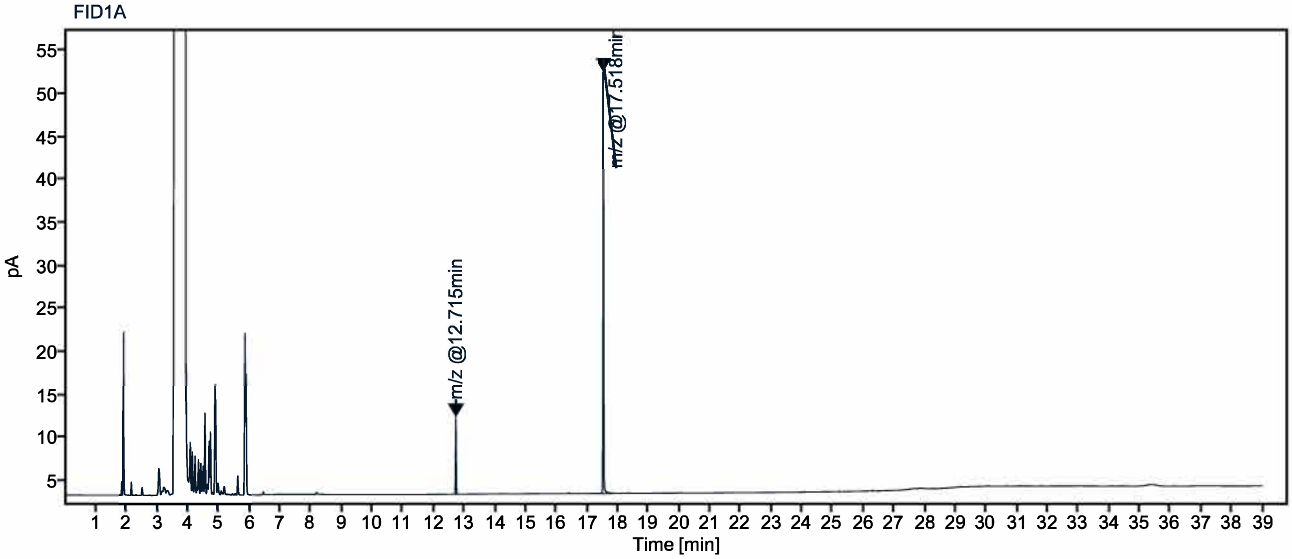


**Signal:** FID1A

| RT [min] | Area | Height |
|---|---|---|
| 12.715 | 12.3857 | 9.0162 |
| 17.518 | 77.1756 | 49.0882 |
| Sum | 89.5613 | |

**Signal:** MS1Front TIC SCAN EI

| RT [min] | Area | Height |
|---|---|---|
| 12.737 | 351252.7579 | 263083.8263 |
| 17.544 | 2718894.7076 | 1909392.5749 |
| 17.617 | 41776.4708 | 25926.4879 |
| Sum | 3111923.9364 | |

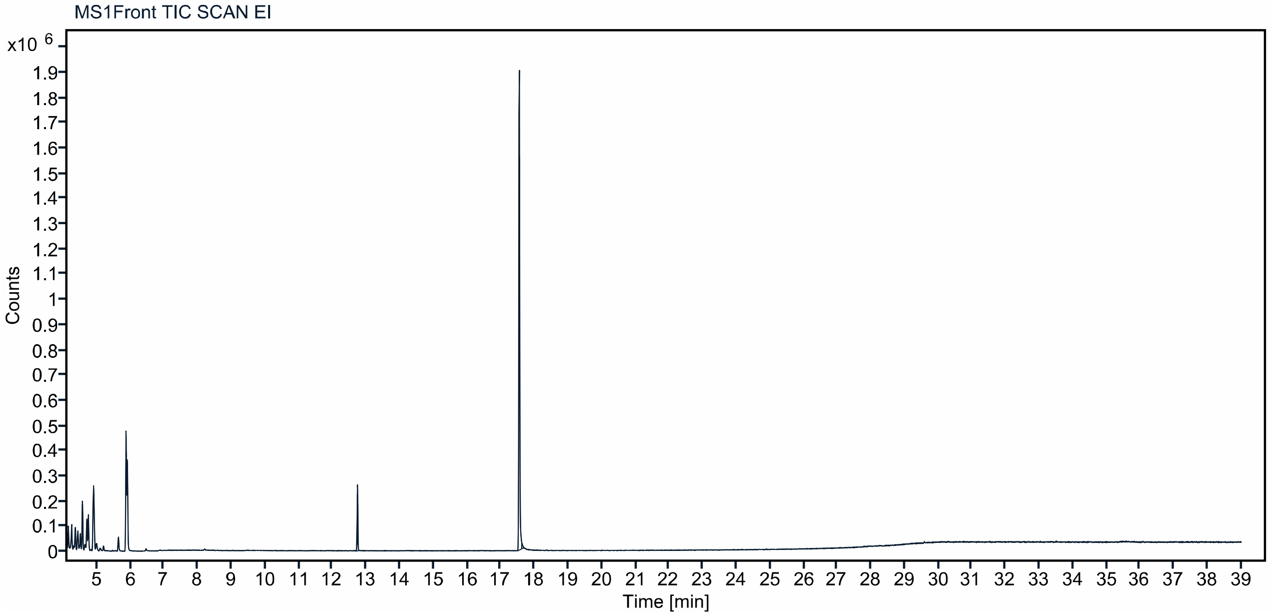


| RT | Compound Name | Score | Rev. Score | Prob. % | CAS # | Library Id |
|---|---|---|---|---|---|---|
| 12.737 | Dodecane | 905 | 922 | 24.15 | 112-40-3 | 26119 |

| RT | Compound Name | Score | Rev. Score | Prob. % | CAS # | Library Id |
|---|---|---|---|---|---|---|
| 17.544 | Benzaldehyde, 4-(dimethylamino)- | 932 | 935 | 87.01 | 100-10-7 | 149616 |

| RT | Compound Name | Score | Rev. Score | Prob. % | CAS # | Library Id |
|---|---|---|---|---|---|---|
| 17.617 | Benzaldehyde, 4-(dimethylamino)- | 764 | 801 | 63.93 | 100-10-7 | 149616 |

# Single Injection Report

Agilent Technologies

| | |
|---|---|
| **Data file:** | D:\CDSProjects\GCMS Liliana\Results\Wenjun\WJ206-49-50ppm-80-IPA-new.sirslt\WJ206-49-50ppm-80-IPA-new.dx |
| **Sample name:** | WJ206-49-50ppm-80-IPA-new |
| **Description:** | |
| **Sample amount:** | 1.000 |

| | |
|---|---|
| **Instrument:** | GCMS |
| **Injection date:** | 2020-03-15 12:22:30+01:00 |
| **Acq. method:** | DB5MS 50-180-300_WY.amx |
| **Analysis method:** | Bob_FID+MS.pmx |
| **Last changed:** | 2019-08-30 14:05:20+02:00 |

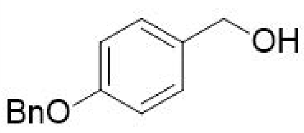


**9t**, 99%

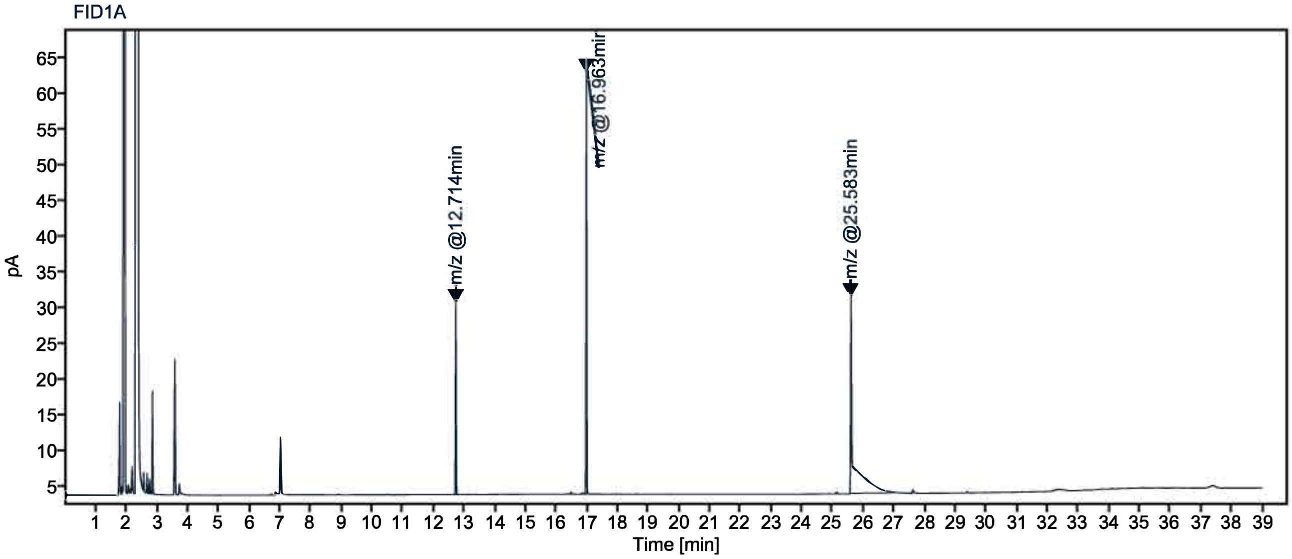


**Signal:** FID1A

| RT [min] | Area | Height |
|---|---|---|
| 12.714 | 36.6342 | 26.8250 |
| 16.963 | 80.6820 | 59.0339 |
| 25.583 | 176.8570 | 27.5325 |
| Sum | 294.1731 | |

**Signal:** MS1Front TIC SCAN EI

| RT [min] | Area | Height |
|---|---|---|
| 12.737 | 1018267.5458 | 775410.7938 |
| 16.988 | 3585273.1354 | 2829016.0232 |
| 25.612 | 357061.4463 | 161529.5577 |
| Sum | 4960602.1275 | |

# Single Injection Report

Agilent Technologies

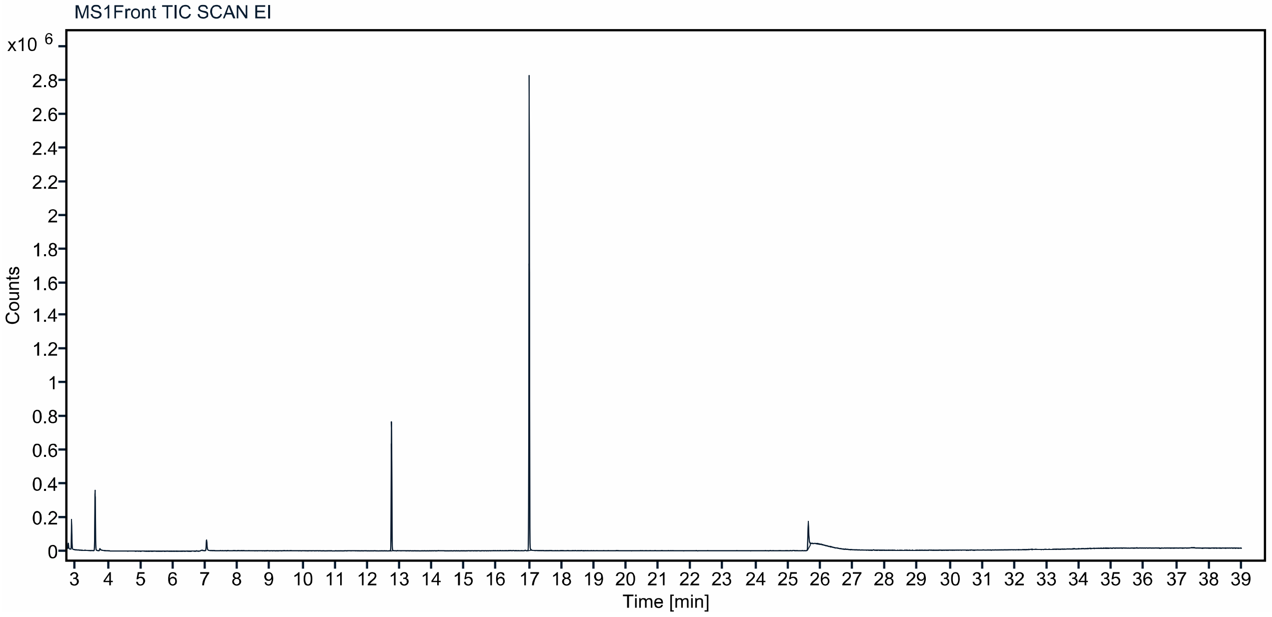


| RT | Compound Name | Score | Rev. Score | Prob. % | CAS # | Library Id |
|---|---|---|---|---|---|---|
| 12.737 | Dodecane | 948 | 949 | 32.06 | 112-40-3 | 26119 |

| RT | Compound Name | Score | Rev. Score | Prob. % | CAS # | Library Id |
|---|---|---|---|---|---|---|
| 16.988 | Butylated Hydroxytoluene | 968 | 970 | 86.35 | 128-37-0 | 206178 |

| RT | Compound Name | Score | Rev. Score | Prob. % | CAS # | Library Id |
|---|---|---|---|---|---|---|
| 25.612 | p-Benzyloxybenzyl alcohol | 878 | 901 | 49.48 | 836-43-1 | 65214 |

# Single Injection Report

Agilent Technologies

**Data file:** D:\CDSProjects\GCMS Liliana\Results\Wenjun\WJ206-49-initial.sirslt\WJ206-49-initial.dx
**Sample name:** WJ206-49-initial
**Description:**
**Sample amount:** 1.000

**Instrument:** GCMS
**Injection date:** 2020-03-15 18:15:07+01:00
**Acq. method:** DB5MS 50-180-300_WY.amx
**Analysis method:** Bob_FID+MS.pmx
**Last changed:** 2019-08-30 14:05:20+02:00

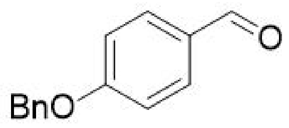


**8t**

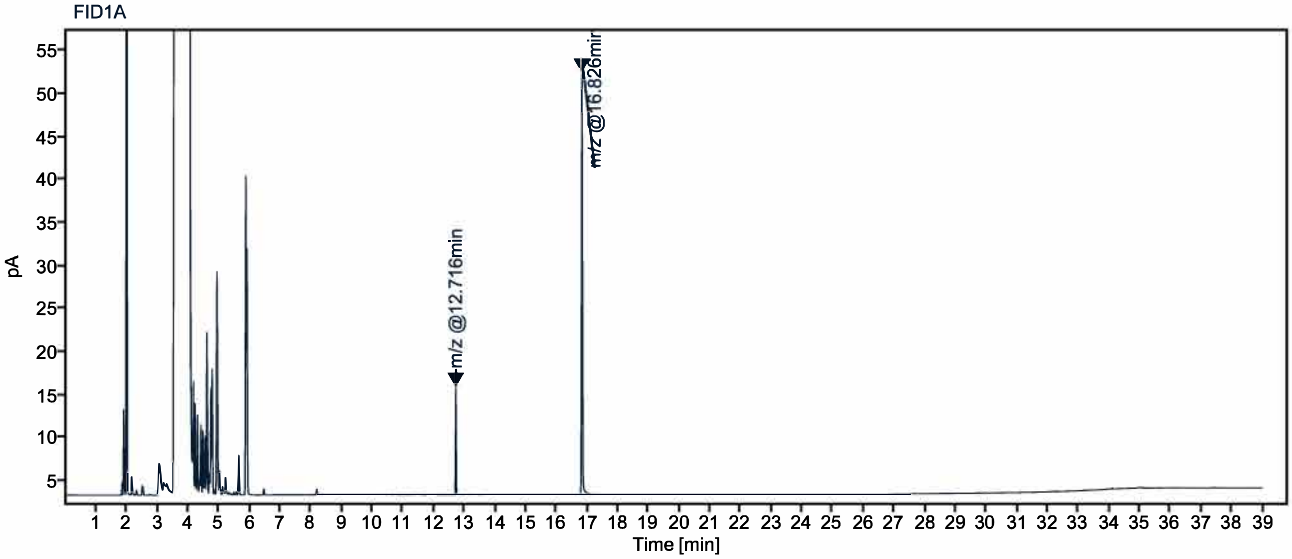


**Signal:** FID1A

| RT [min] | Area | Height |
|---|---|---|
| 12.716 | 17.2442 | 12.6592 |
| 16.826 | 84.3246 | 49.1752 |
| Sum | 101.5688 | |

**Signal:** MS1Front TIC SCAN EI

| RT [min] | Area | Height |
|---|---|---|
| 12.739 | 424267.3726 | 321137.1283 |
| 16.851 | 3114783.6872 | 1972419.9952 |
| Sum | 3539051.0598 | |

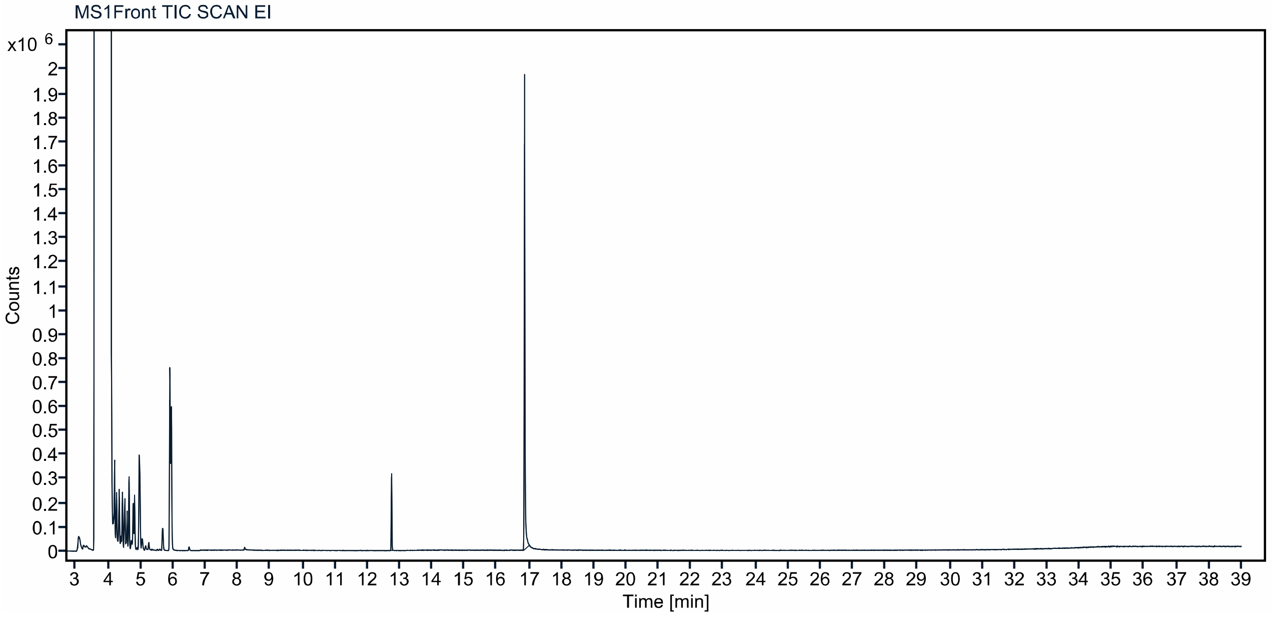


| RT | Compound Name | Score | Rev. Score | Prob. % | CAS # | Library Id |
|---|---|---|---|---|---|---|
| 12.739 | Dodecane | 937 | 948 | 33.73 | 112-40-3 | 26119 |

| RT | Compound Name | Score | Rev. Score | Prob. % | CAS # | Library Id |
|---|---|---|---|---|---|---|
| 16.851 | Acetophenone, 4'-amino- | 959 | 959 | 64.81 | 99-92-3 | 110804 |

# Single Injection Report

Agilent Technologies

**Data file:** D:\CDSProjects\GCMS Liliana\Results\Wenjun\WJ206-47-100ppm-80-IPA-new-initial.sirslt\WJ206-47-100ppm-80-IPA-new-initial.dx

**Sample name:** WJ206-47-100ppm-80-IPA-new-initial

**Description:**

**Sample amount:** 1.000

**Instrument:** GCMS

**Injection date:** 2020-03-15 14:33:53+01:00

**Acq. method:** DB5MS 50-180-300_WY.amx

**Analysis method:** Bob_FID+MS.pmx

**Last changed:** 2019-08-30 14:05:20+02:00

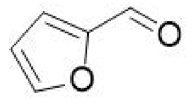

**8u**

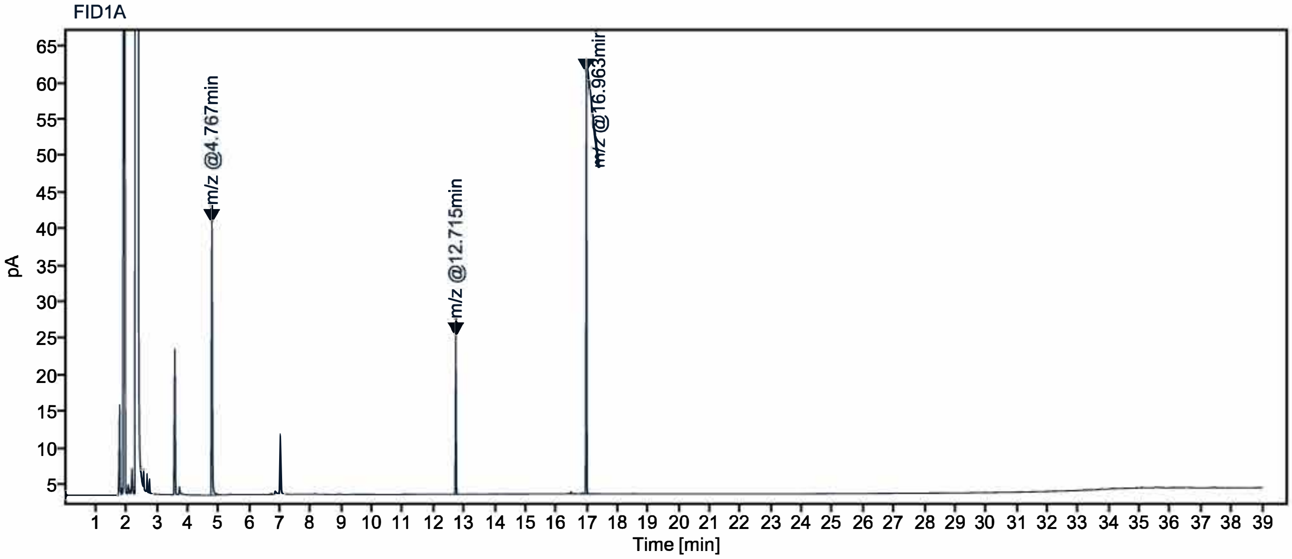


**Signal:** FID1A

| RT [min] | Area | Height |
|---|---|---|
| 4.767 | 72.2713 | 37.2921 |
| 12.715 | 29.8239 | 21.6362 |
| 16.963 | 79.4932 | 57.7487 |
| Sum | 181.5883 | |

**Signal:** MS1Front TIC SCAN EI

| RT [min] | Area | Height |
|---|---|---|
| 4.788 | 1423266.4831 | 771687.2726 |
| 12.737 | 1061026.3677 | 810227.3366 |
| 16.988 | 3180164.8825 | 2500923.4460 |
| Sum | 5664457.7333 | |

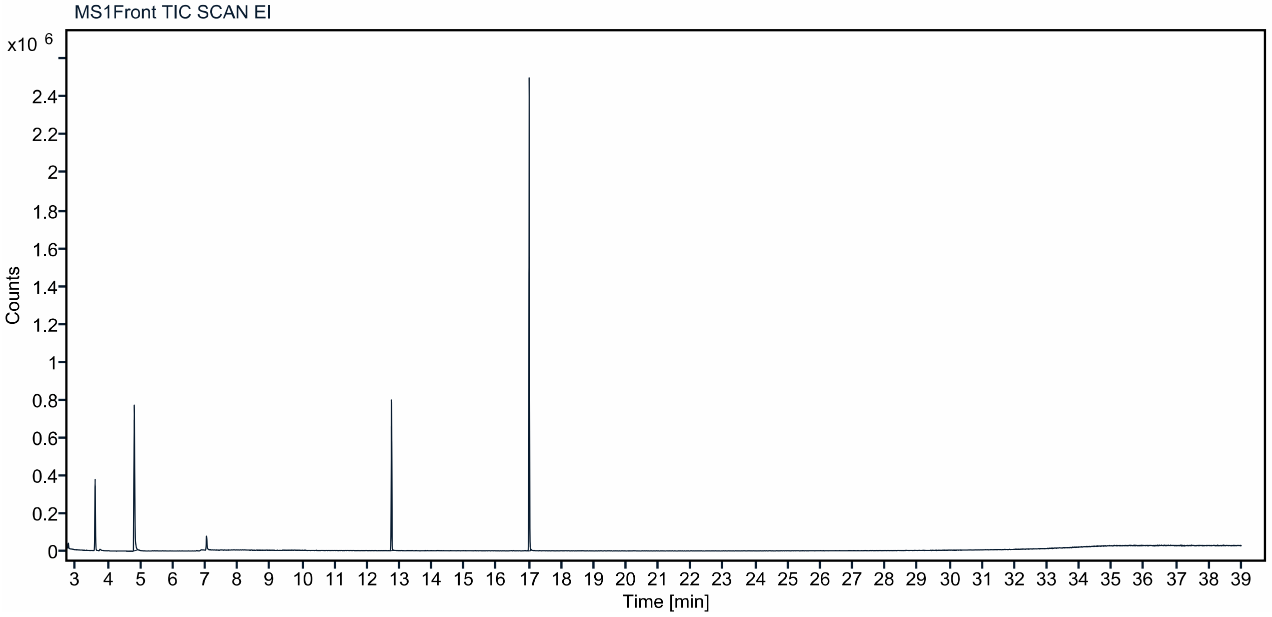


| RT | Compound Name | Score | Rev. Score | Prob. % | CAS # | Library Id |
|---|---|---|---|---|---|---|
| 4.788 | Furfural | 939 | 940 | 64.74 | 98-01-1 | 76294 |

| RT | Compound Name | Score | Rev. Score | Prob. % | CAS # | Library Id |
|---|---|---|---|---|---|---|
| 12.737 | Dodecane | 935 | 938 | 24.89 | 112-40-3 | 26119 |

| RT | Compound Name | Score | Rev. Score | Prob. % | CAS # | Library Id |
|---|---|---|---|---|---|---|
| 16.988 | Butylated Hydroxytoluene | 960 | 961 | 84.51 | 128-37-0 | 206178 |

# Single Injection Report

Agilent Technologies

**Data file:** D:\CDSProjects\GCMS Liliana\Results\Wenjun\WJ206-47-100ppm-80-IPA-new.sirslt\WJ206-47-100ppm-80-IPA-new.dx

**Sample name:** WJ206-47-100ppm-80-IPA-new

**Description:**

**Sample amount:** 1.000

**Instrument:** GCMS

**Injection date:** 2020-03-15 11:38:39+01:00

**Acq. method:** DB5MS 50-180-300_WY.amx

**Analysis method:** Bob_FID+MS.pmx

**Last changed:** 2019-08-30 14:05:20+02:00

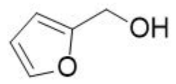


**9u**, 54%

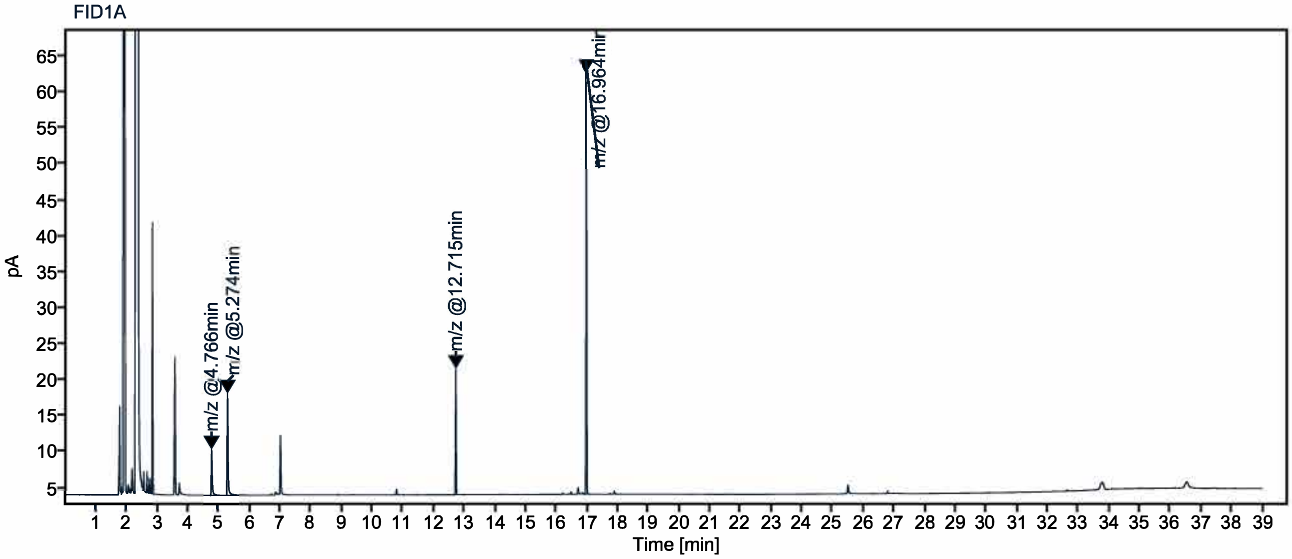


**Signal:** FID1A

| RT [min] | Area | Height |
|---|---|---|
| 4.766 | 14.0647 | 6.3986 |
| 5.274 | 30.7003 | 14.1735 |
| 12.715 | 23.9251 | 17.5301 |
| 16.964 | 79.7770 | 58.5011 |
| Sum | 148.4671 | |

**Signal:** MS1Front TIC SCAN EI

| RT [min] | Area | Height |
|---|---|---|
| 4.789 | 236219.1624 | 111419.9072 |
| 5.297 | 542408.8364 | 306942.4366 |
| 12.737 | 900483.2494 | 689059.7892 |
| 16.988 | 3422167.3282 | 2666796.6937 |
| Sum | 5101278.5764 | |

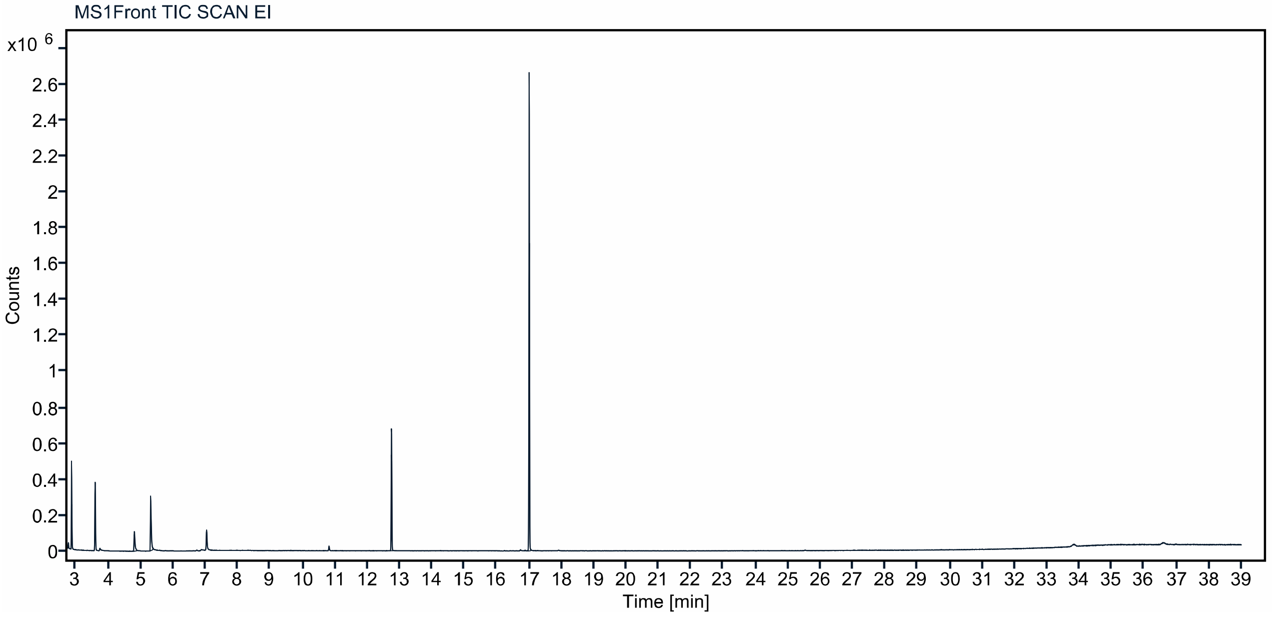


| RT | Compound Name | Score | Rev. Score | Prob. % | CAS # | Library Id |
|---|---|---|---|---|---|---|
| 4.789 | Furfural | 930 | 937 | 55.85 | 98-01-1 | 76294 |

| RT | Compound Name | Score | Rev. Score | Prob. % | CAS # | Library Id |
|---|---|---|---|---|---|---|
| 5.297 | 2-Furanmethanol | 925 | 926 | 73.91 | 98-00-0 | 77694 |

| RT | Compound Name | Score | Rev. Score | Prob. % | CAS # | Library Id |
|---|---|---|---|---|---|---|
| 12.737 | Dodecane | 929 | 932 | 25.68 | 112-40-3 | 26119 |

| RT | Compound Name | Score | Rev. Score | Prob. % | CAS # | Library Id |
|---|---|---|---|---|---|---|
| 16.988 | Butylated Hydroxytoluene | 956 | 957 | 82.43 | 128-37-0 | 206178 |

# Single Injection Report

Agilent Technologies

**Data file:** D:\CDSProjects\GCMS Liliana\Results\Wenjun\WJ206-29-100PPM-ipa-120oC.sirslt\WJ206-29-100PPM-ipa-120oC.dx
**Sample name:** WJ206-29-100PPM-ipa-120oC
**Description:**
**Sample amount:** 0.000

**Instrument:** GCMS
**Injection date:** 2020-01-16 23:56:03+01:00
**Acq. method:** DB5MS 60-300_WY.amx
**Analysis method:** Bob_FID+MS.pmx
**Last changed:** 2019-08-30 14:05:20+02:00

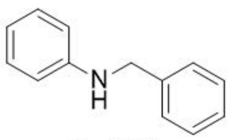

**9v**, 90%

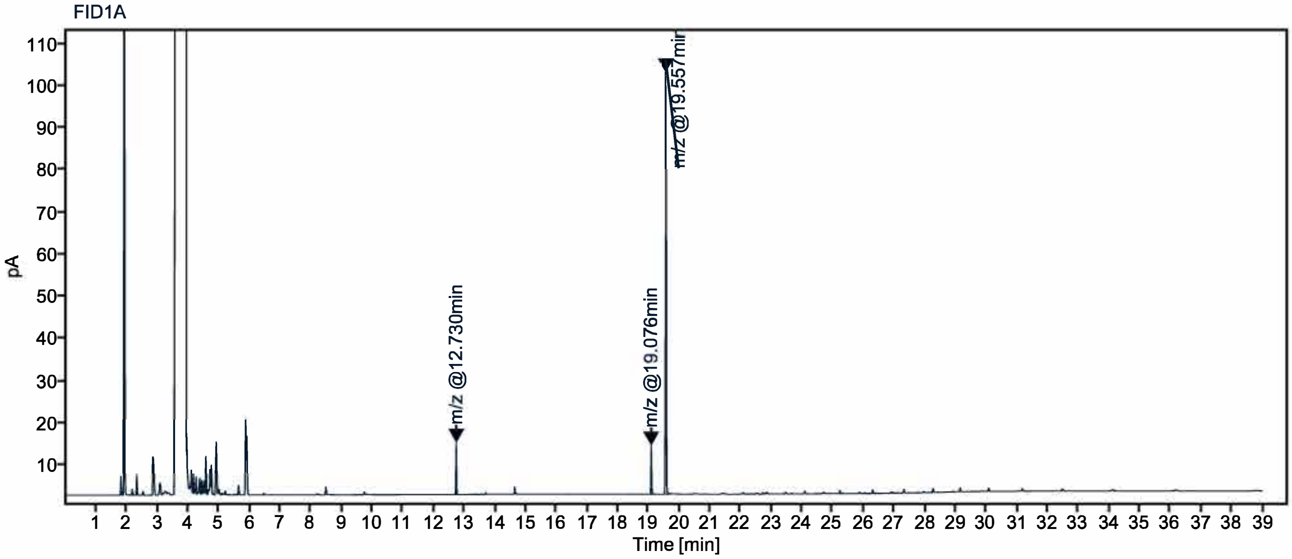


**Signal:** FID1A

| RT [min] | Area | Height |
|---|---|---|
| 12.730 | 16.9134 | 12.3849 |
| 19.076 | 16.9499 | 11.6190 |
| 19.557 | 151.5941 | 100.1635 |
| Sum | 185.4574 | |

**Signal:** MS1Front TIC SCAN EI

| RT [min] | Area | Height |
|---|---|---|
| 12.753 | 454083.4447 | 350489.9936 |
| 19.103 | 210716.5266 | 133963.0667 |
| 19.582 | 4281858.3218 | 3130092.8024 |
| Sum | 4946658.2932 | |

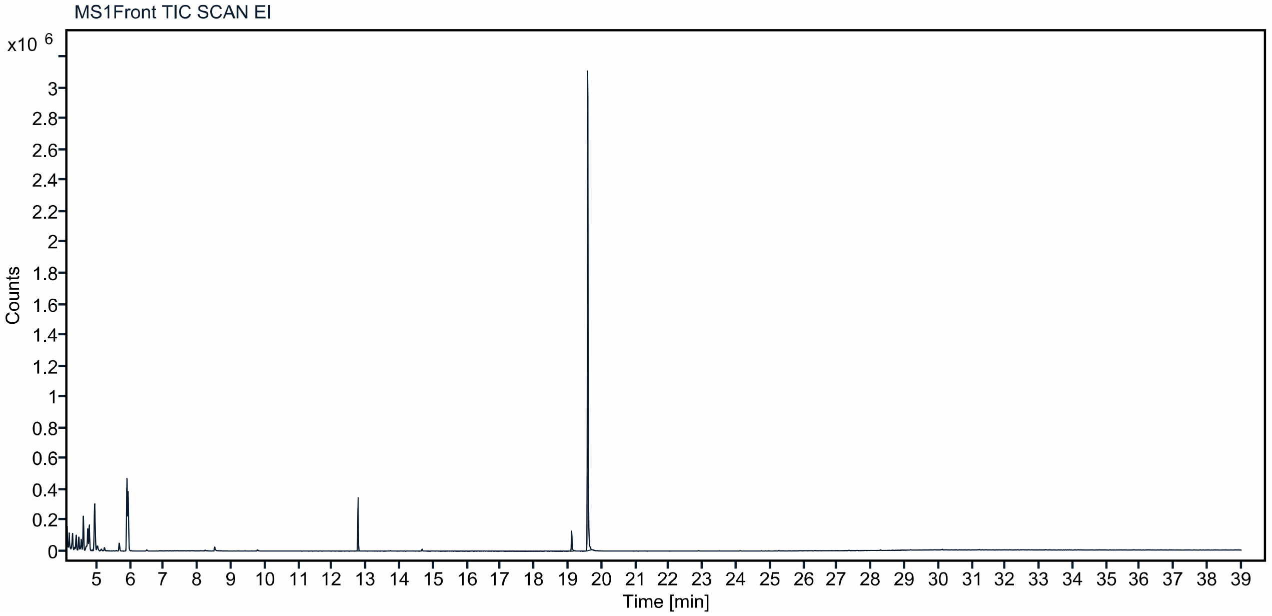


| RT | Compound Name | Score | Rev. Score | Prob. % | CAS # | Library Id |
|---|---|---|---|---|---|---|
| 12.753 | Dodecane | 912 | 918 | 19.57 | 112-40-3 | 26119 |

| RT | Compound Name | Score | Rev. Score | Prob. % | CAS # | Library Id |
|---|---|---|---|---|---|---|
| 19.103 | Benzenamine, N-(phenylmethylene)- | 888 | 891 | 67.62 | 538-51-2 | 185091 |

| RT | Compound Name | Score | Rev. Score | Prob. % | CAS # | Library Id |
|---|---|---|---|---|---|---|
| 19.582 | Benzenemethanamine, N-phenyl- | 919 | 921 | 88.48 | 103-32-2 | 69291 |

# Single Injection Report

Agilent Technologies

**Data file:** D:\CDSProjects\GCMS Liliana\Results\Wenjun\WJ206-29-100PPM-ipa-120oC-initial.sirslt\WJ206-29-100PPM-ipa-120oC-initial.dx
**Sample name:** WJ206-29-100PPM-ipa-120oC-initial
**Description:**
**Sample amount:** 0.000

**Instrument:** GCMS
**Injection date:** 2020-01-17 02:07:22+01:00
**Acq. method:** DB5MS 60-300_WY.amx
**Analysis method:** Bob_FID+MS.pmx
**Last changed:** 2019-08-30 14:05:20+02:00

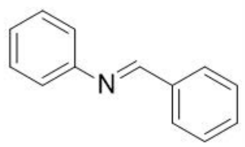


8v

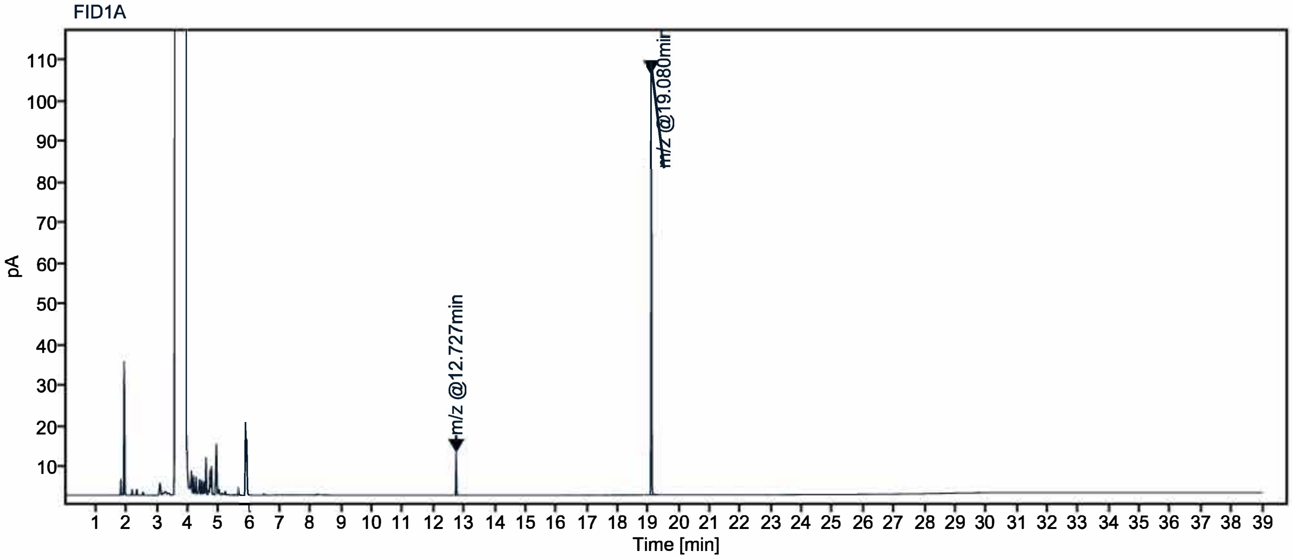


**Signal:** FID1A

| RT [min] | Area | Height |
|---|---|---|
| 12.727 | 14.3881 | 10.5391 |
| 19.080 | 150.1179 | 103.7216 |
| Sum | 164.5061 | |

**Signal:** MS1Front TIC SCAN EI

| RT [min] | Area | Height |
|---|---|---|
| 12.750 | 358512.4046 | 276410.3939 |
| 19.106 | 3922853.0552 | 3104474.1070 |
| Sum | 4281365.4598 | |

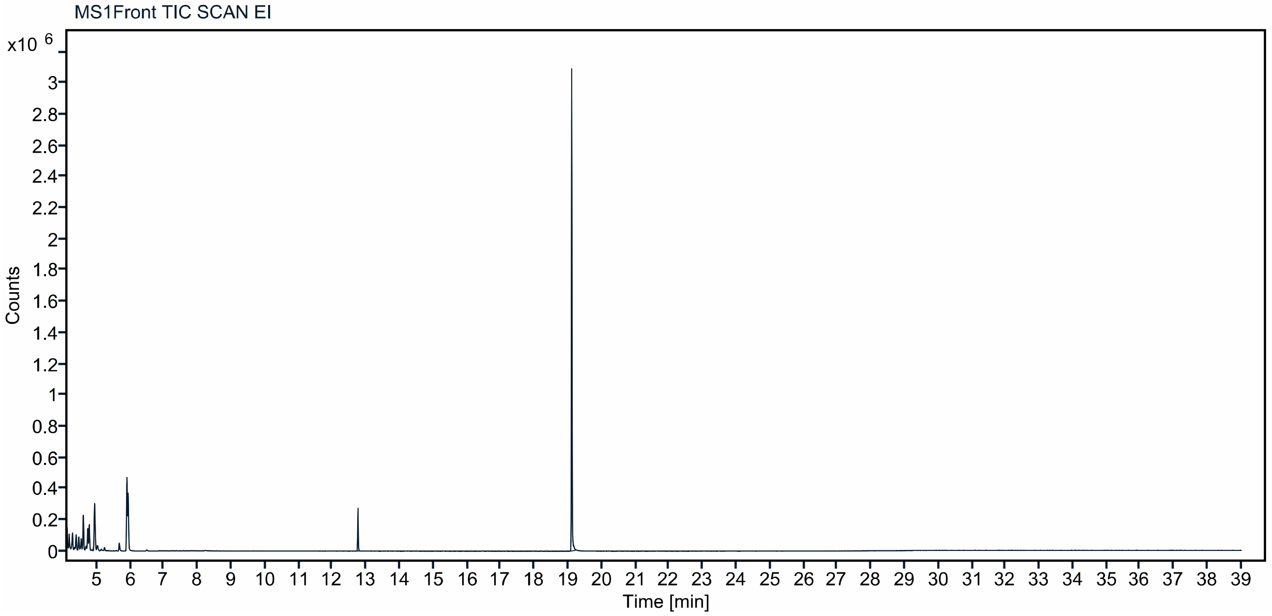


| RT | Compound Name | Score | Rev. Score | Prob. % | CAS # | Library Id |
|---|---|---|---|---|---|---|
| 12.75 | Dodecane | 900 | 908 | 18.51 | 112-40-3 | 26119 |

| RT | Compound Name | Score | Rev. Score | Prob. % | CAS # | Library Id |
|---|---|---|---|---|---|---|
| 19.106 | Benzenamine, N-(phenylmethylene)- | 933 | 933 | 75.08 | 538-51-2 | 185091 |

# Single Injection Report

Agilent Technologies

**Data file:** D:\CDSProjects\GCMS Liliana\Results\Wenjun\WJ206-30-100PPM-ipa-120oC-initial.sirslt\WJ206-30-100PPM-ipa-120oC-initial.dx

**Sample name:** WJ206-30-100PPM-ipa-120oC-initial

**Description:**

**Sample amount:** 0.000

**Instrument:** GCMS

**Injection date:** 2020-01-17 02:51:12+01:00

**Acq. method:** DB5MS 60-300_WY.amx

**Analysis method:** Bob_FID+MS.pmx

**Last changed:** 2019-08-30 14:05:20+02:00

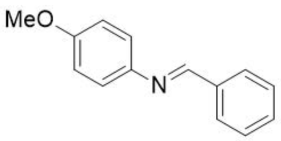


8x

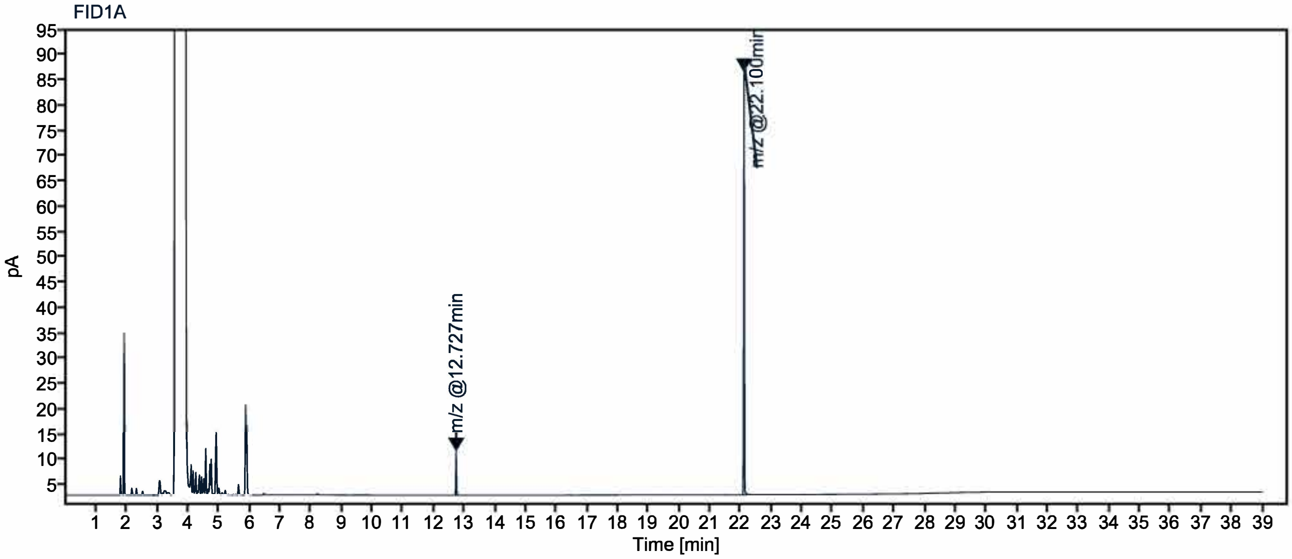


**Signal:** FID1A

| RT [min] | Area | Height |
|---|---|---|
| 12.727 | 12.0311 | 8.7298 |
| 22.100 | 126.4244 | 83.5077 |
| Sum | 138.4555 | |

**Signal:** MS1Front TIC SCAN EI

| RT [min] | Area | Height |
|---|---|---|
| 12.750 | 288782.4053 | 218269.2970 |
| 22.129 | 2250215.65 90 | 1577531.70 18 |
| Sum | 2538998.06 44 | |

# Single Injection Report

Agilent Technologies

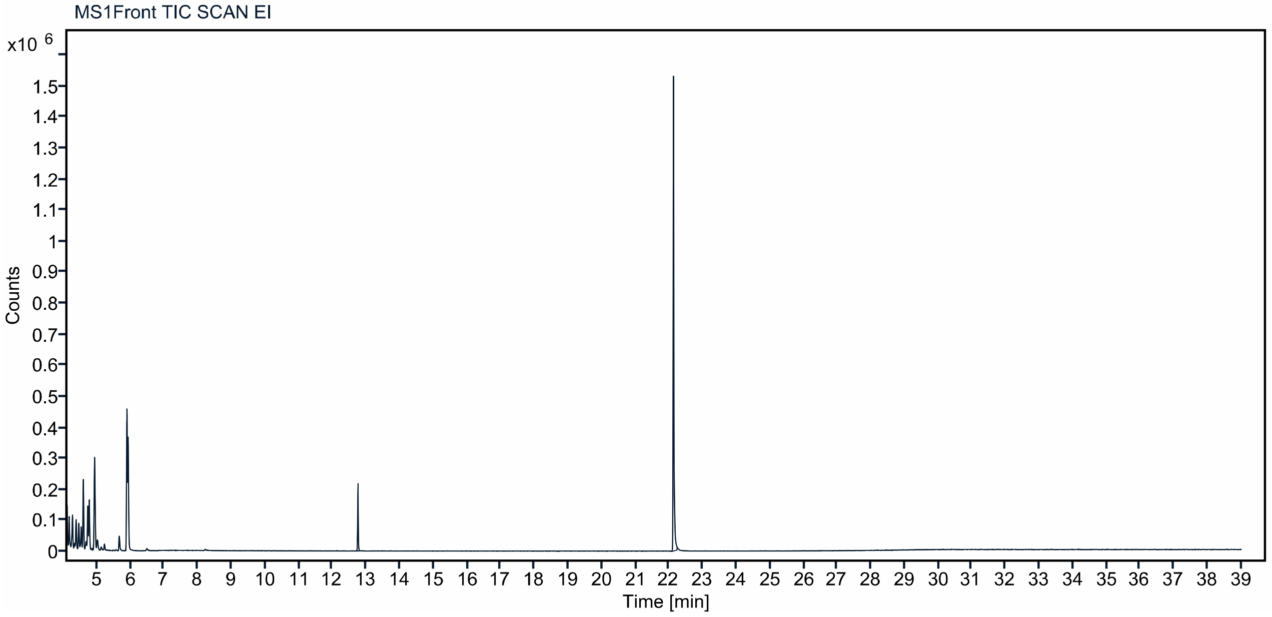


| RT | Compound Name | Score | Rev. Score | Prob. % | CAS # | Library Id |
|---|---|---|---|---|---|---|
| 12.75 | Dodecane | 905 | 911 | 16.19 | 112-40-3 | 26119 |

| RT | Compound Name | Score | Rev. Score | Prob. % | CAS # | Library Id |
|---|---|---|---|---|---|---|
| 22.129 | Benzenamine, 4-methoxy-N-(phenylmethylene)- | 896 | 898 | 91.59 | 783-08-4 | 210290 |

# Single Injection Report

Agilent Technologies

**Data file:** D:\CDSProjects\GCMS Liliana\Results\Wenjun\WJ206-30-100PPM-ipa-120oC.sirslt\WJ206-30-100PPM-ipa-120oC.dx
**Sample name:** WJ206-30-100PPM-ipa-120oC
**Description:**
**Sample amount:** 0.000

**Instrument:** GCMS
**Injection date:** 2020-01-17 00:39:47+01:00
**Acq. method:** DB5MS 60-300_WY.amx
**Analysis method:** Bob_FID+MS.pmx
**Last changed:** 2019-08-30 14:05:20+02:00

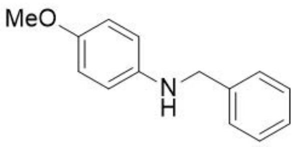


**9x**, >99%

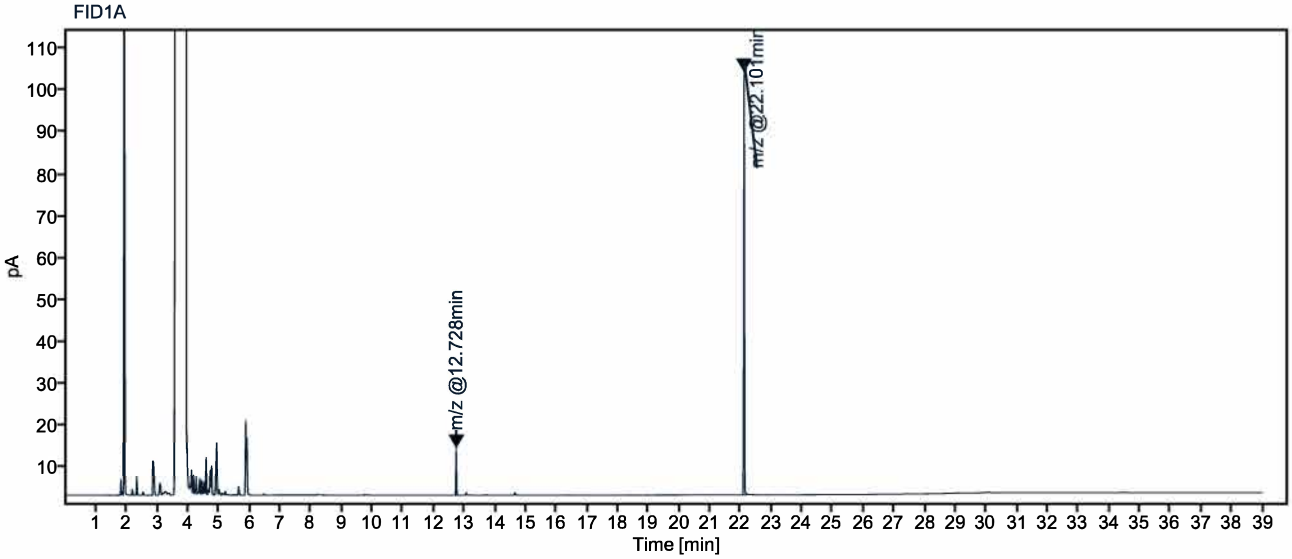


**Signal:** FID1A

| RT [min] | Area | Height |
|---|---|---|
| 12.728 | 15.3570 | 11.3225 |
| 22.101 | 159.4166 | 101.2988 |
| Sum | 174.7736 | |

**Signal:** MS1Front TIC SCAN EI

| RT [min] | Area | Height |
|---|---|---|
| 12.751 | 397969.1540 | 303685.5015 |
| 22.130 | 3216436.3879 | 2245359.7998 |
| Sum | 3614405.5418 | |

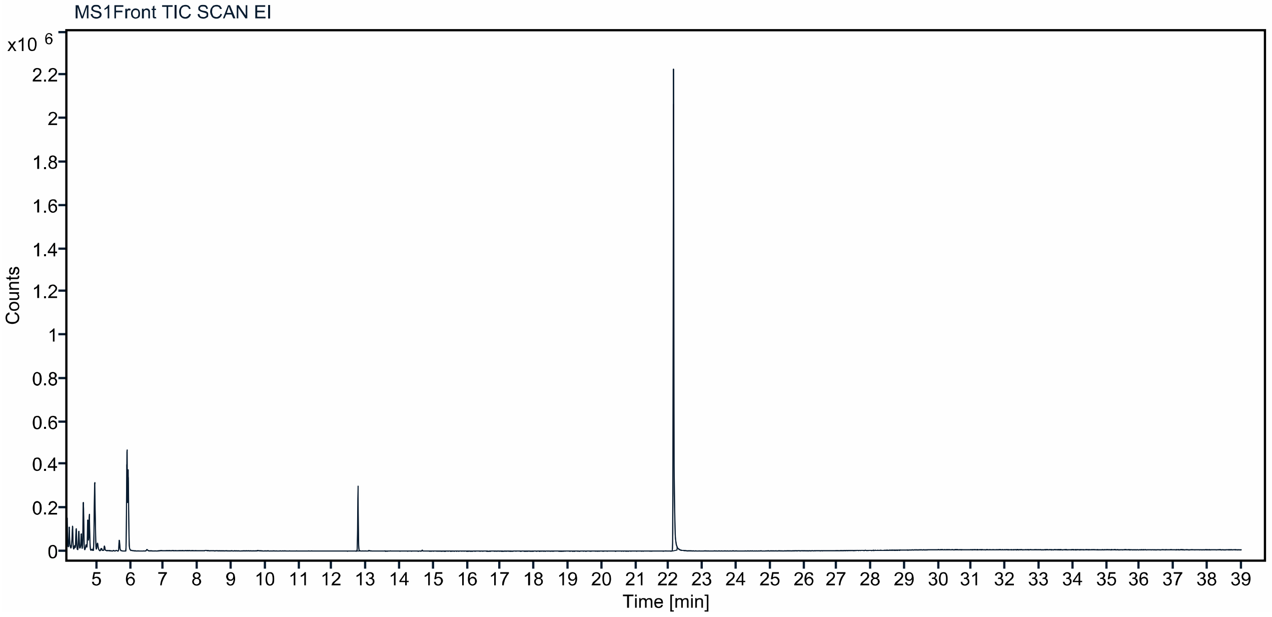


| RT | Compound Name | Score | Rev. Score | Prob. % | CAS # | Library Id |
|---|---|---|---|---|---|---|
| 12.751 | Dodecane | 911 | 918 | 20.51 | 112-40-3 | 26119 |

| RT | Compound Name | Score | Rev. Score | Prob. % | CAS # | Library Id |
|---|---|---|---|---|---|---|
| 22.13 | N-Benzyl-p-anisidine | 893 | 899 | 97.71 | 17377-95-6 | 67604 |
